# Tris(pentafluorophenyl)borane‐Catalyzed Carbenium Ion Generation and Autocatalytic Pyrazole Synthesis—A Computational and Experimental Study

**DOI:** 10.1002/anie.202109744

**Published:** 2021-10-12

**Authors:** Ayan Dasgupta, Rasool Babaahmadi, Sanjukta Pahar, Katarina Stefkova, Lukas Gierlichs, Brian F. Yates, Alireza Ariafard, Rebecca L. Melen

**Affiliations:** ^1^ Cardiff Catalysis Institute School of Chemistry Cardiff University Main Building, Park Place Cardiff CF10 3AT Cymru/Wales United Kingdom; ^2^ School of Physical Sciences University of Tasmania Private Bag 75 Hobart Tasmania 7001 Australia

**Keywords:** aryl ester, autocatalysis, carbenium, diazoester, pyrazole

## Abstract

In recent years, metal‐free organic synthesis using triarylboranes as catalysts has become a prevalent research area. Herein we report a comprehensive computational and experimental study for the highly selective synthesis of N‐substituted pyrazoles through the generation of carbenium species from the reaction between aryl esters and vinyl diazoacetates in the presence of catalytic tris(pentafluorophenyl)borane [B(C_6_F_5_)_3_]. DFT studies were undertaken to illuminate the reaction mechanism revealing that the in situ generation of a carbenium species acts as an autocatalyst to prompt the regiospecific formation of N‐substituted pyrazoles in good to excellent yields (up to 81 %).

## Graphical abstract

Tris(pentafluorophenyl)borane‐catalyzed reactions between vinyl diazoacetates and aryl esters afforded highly regioselective N‐alkylated pyrazoles. These reactions involve an autocatalytic process in which in situ formed carbenium species act as a catalyst to regioselectively afford pyrazoles. Detailed DFT studies were carried out to explain the reaction mechanism.

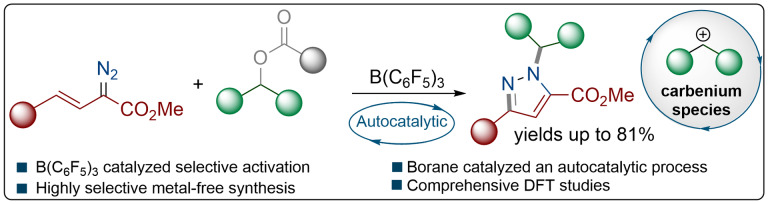

An autocatalytic process is one wherein the products of a reaction act to catalyze the chemical reaction in which they themselves are formed.^[1]^ The fascinating nature of autocatalytic processes has resulted in many investigations to establish their mode and mechanism of action. Indeed, one area which has been thoroughly investigated for autocatalysis is organocatalysis,^[2]^ where such autocatalytic process can often amplify the selectivity of reactions.^[3]^ Uses of boranes in an autocatalytic system remain underexplored. The usage of Lewis acidic boranes, such as tris(pentafluorophenyl)borane, B(C_6_F_5_)_3_, as catalysts in organic synthesis has gained unprecedent attention as an alternative to many common transition metals.^[4]^ Recently ourselves,^[5]^ and others^[6a]^ have explored the catalytic activity of B(C_6_F_5_)_3_ in the activation of diazoesters leading to the formation of carbene intermediates through the elimination of N_2_. The carbene intermediate can subsequently be used as building block for the synthesis of novel organic molecules through a range of different reactions such as O−H/N−H/C−H insertion,[^5a^, ^6b^, ^6d^, ^6g^, ^6h^] azide/carbonate transfer,[^6e^, ^6f^] cyclopropanation/cyclopropenation,[^5a^, ^6c^] and the ring‐opening of heterocyclic compounds.^[5a]^ In this study, we were interested in the B(C_6_F_5_)_3_‐catalyzed synthesis of pyrazoles from vinyl diazoacetates in which the N_2_ functionality of the diazo starting material is not released. The metal‐free synthesis of nitrogen‐containing heterocycles is an important area of research as most of these heterocycles are of biological importance.^[7]^ Pyrazoles are an important class of nitrogen‐based heterocycles that are omnipresent in natural products and therefore have a broad impact in medicinal chemistry.^[8]^ Thus, a metal‐free synthesis of functionalized pyrazole compounds is desirable as synthetic routes need to avoid trace impurities of toxic metals in the final compounds. Herein we report the reactions between aryl esters and vinyl diazoacetates in the presence of catalytic B(C_6_F_5_)_3_ to afford N‐alkylated pyrazoles in a selective manner (Scheme 1 C). The only report of a similar reaction is with gold‐based catalysts where selective decomposition of a diazoester in the presence of a second diazoester generates a pyrazole product (Scheme 1 B).^[9]^

**Scheme 1:**
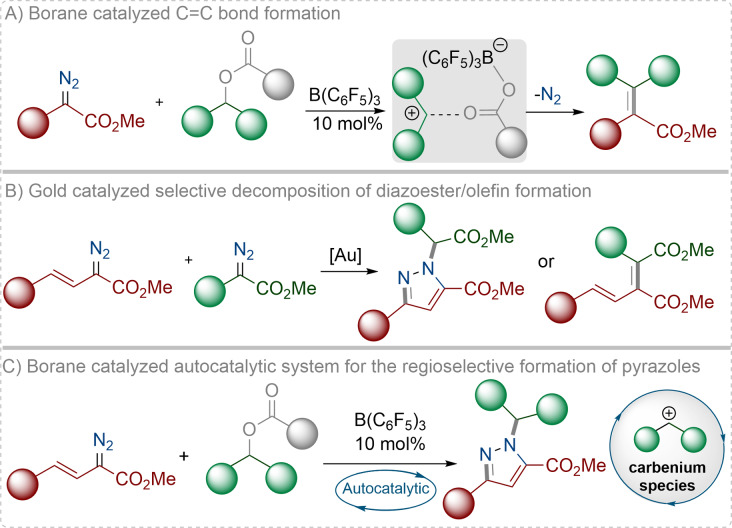
Previous and current work. A) General representation for boranecatalyzed alkenylation. B) Gold‐catalyzed pyrazole synthesis. C) This work on borane‐catalyzed autocatalytic system for pyrazole synthesis.

In our previous work, we demonstrated that, in the presence of catalytic amounts of B(C_6_F_5_)_3_, α‐aryl α‐diazoesters readily react with aryl esters to afford C=C cross‐coupled products with the elimination of N_2_ through an alkenylation reaction (Scheme 1 A).^[10]^ Our initial interest was to develop this previous work further to make 1,3‐diene ester compounds^[11]^ using vinyl diazoacetates rather than α‐aryl α‐diazoesters. To this end we synthesized the vinyl diazoacetate compound (**1 a**, Scheme 2) and treated it with an aryl ester (**2 a**), with catalytic amounts of B(C_6_F_5_)_3_ (10 mol %). Following the reaction at 50 °C in 1,2‐C_2_H_4_Cl_2_ for 20 h, the resulting product was purified via preparative thin layer chromatography to yield a white solid. The solid however could not be attributed to the product from the alkenylation reaction when observing the multinuclear NMR spectra and high‐resolution mass spectrometric data. Slow evaporation of the solid from CH_2_Cl_2_ gave a crop of colorless crystals of the product. Single‐crystal X‐ray diffraction analysis unequivocally confirmed the product to be a substituted pyrazole ring compound **3 a** that was formed in 76 % yield (Figure 1).

**Figure 1:**
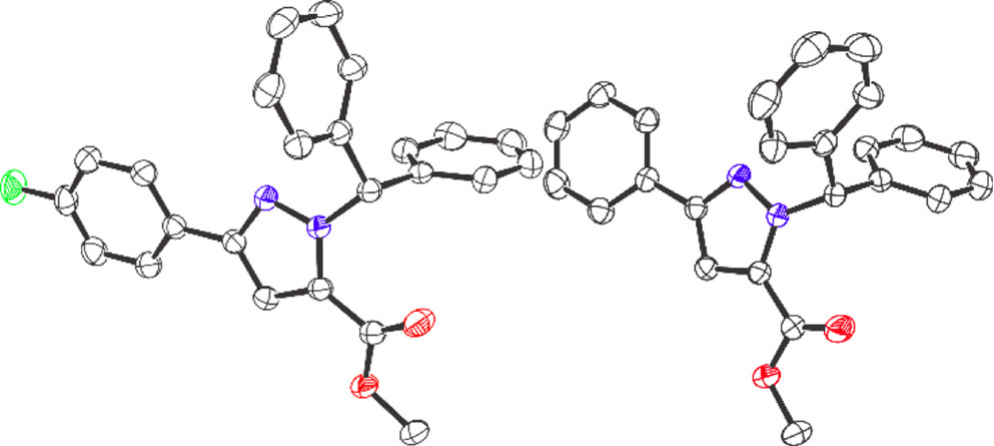
Crystal structure of **3 a** (left) and **3 d** (right). Thermal ellipsoids shown at 50 %. H atoms omitted for clarity. Carbon: black; Oxygen: red; Nitrogen: blue; Fluorine: green.

**Scheme 2:**
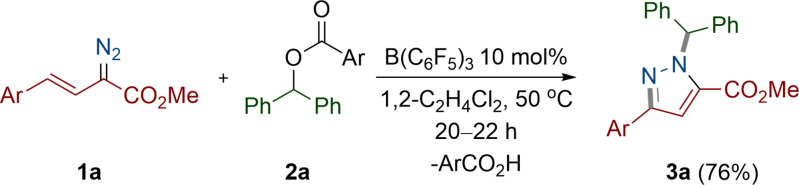
Reaction between vinyl diazoacetate (**1 a**) and aryl ester (**2 a**). Ar=*p*‐FC_6_H_4_.

In an attempt to explain the product formation, we hypothesized that the B(C_6_F_5_)_3_ catalyst could either (i) activate the diazoester compound through the ester (**1**⋅B(C_6_F_5_)_3_) or diazo (**1′**⋅B(C_6_F_5_)_3_) functionalities, or (ii) activate the aryl ester (**2**⋅B(C_6_F_5_)_3_) in the first step of the reaction (Scheme 3 A). In the scenario that B(C_6_F_5_)_3_ activates diazo compound **1**, one plausible pathway for the reaction could be initial heterocycle **4** formation (formed from the intramolecular attack of the nitrogen atom onto the alkene) followed by B(C_6_F_5_)_3_ catalyzed N‐alkylation. To investigate the mechanism for the reaction, we undertook DFT calculations at the SMD/M06‐2X‐D3/def2‐TZVP//SMD/M06‐2X/6‐31G(d) level of theory. Using the vinyl diazoacetate compound **1 b** as an example, the activation barrier to afford the pyrazole product **4** was found to be 34.7 kcal mol^−1^ in the absence of a catalyst (Scheme 3 B).

**Scheme 3:**
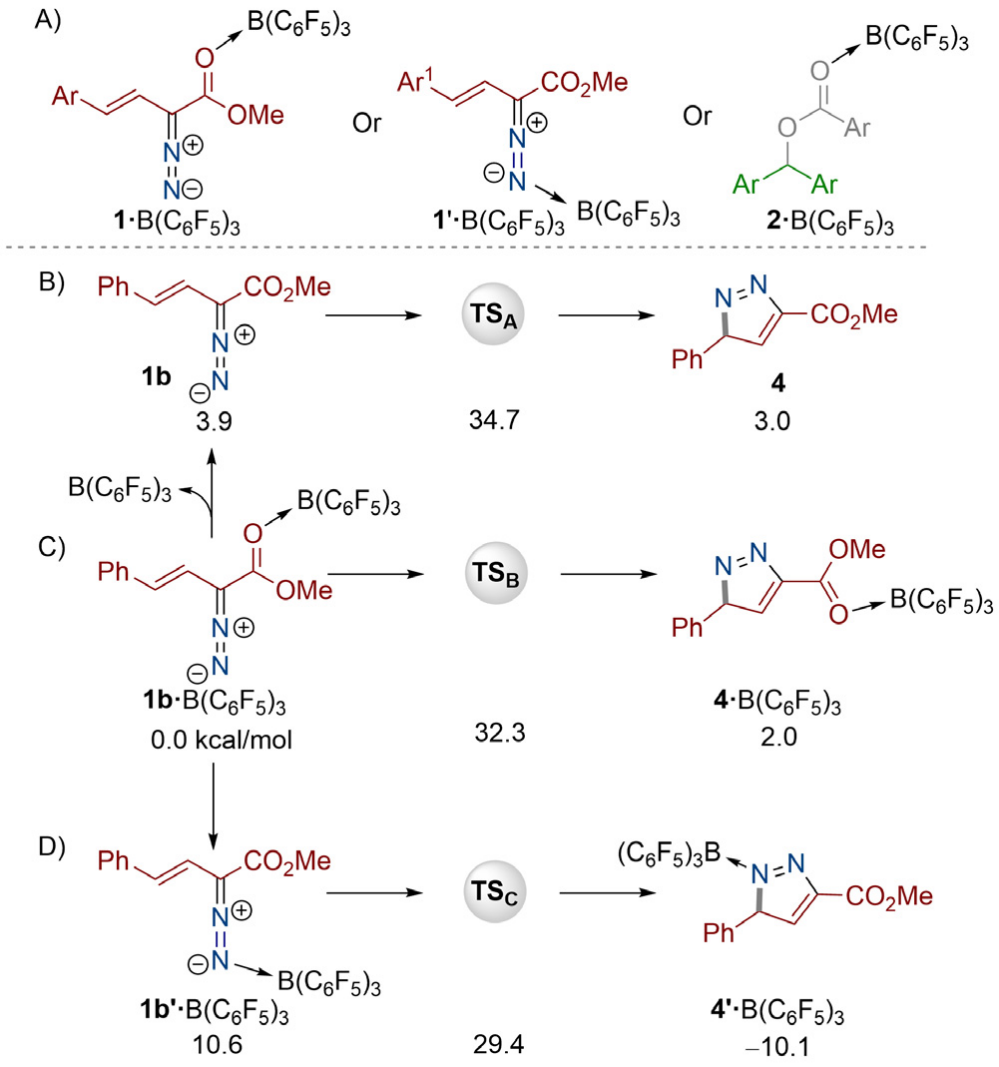
B(C_6_F_5_)_3_ activation modes (A). Calculated energy barriers (kcal mol^−1^) for the cyclization of the diazo compound (B–D). DFT calculations at the SMD/M06‐2X‐D3/def2‐TZVP//SMD/M06‐2X/6‐31G(d) level of theory using CH_2_Cl_2_.

Interestingly, addition of B(C_6_F_5_)_3_ to activate the ester functionality (**1 b**⋅B(C_6_F_5_)_3_) does not significantly change the activation barrier (32.3 kcal mol^−1^) to generate the pyrazole as an ester O→B(C_6_F_5_)_3_ adduct **4**⋅B(C_6_F_5_)_3_ (Scheme 3 C). In addition, experimental data reveals that the 1:1 stoichiometric reaction between **1 b** and B(C_6_F_5_)_3_ led to the formation of an allylic substituted product.^[12]^ Alternatively, activation of the terminal nitrogen atom of the diazo functionality lowered the activation barrier slightly to 29.4 kcal mol^−1^ to yield the N→B(C_6_F_5_)_3_ adduct **4′**⋅B(C_6_F_5_)_3_ (Scheme 3 D). Given the high activation barrier for this reaction in all scenarios, we next investigated B(C_6_F_5_)_3_ activation of the ester **2** as the initial step of the reaction (Scheme 4 and Figure 2) as observed in our previous work.^[13]^ Initial coordination of the borane to the ester **2 b** was found to be favorable by 5.6 kcal mol^−1^ yielding **2 b**⋅B(C_6_F_5_)_3_ (Figure 2). Indeed, the ^11^B NMR spectrum of the 1:1 stoichiometric reaction of **2 b** and B(C_6_F_5_)_3_ showed initial adduct formation indicated by a broad peak at 4.87 ppm. This promotes the formation of carbenium ion **7** through **TS_1_** of 15.3 kcal mol^−1^. Reaction of the carbenium ion with the vinyl diazoacetate **1 b** through the terminal nitrogen atom generates the high energy intermediate **8** through **TS_2_** (13.9 kcal mol^−1^), which in turn rapidly cyclizes through a low energy transition state (**TS_3_** =4.7 kcal mol^−1^) generating the heterocycle **9** in which the pyrazole core has been formed. Alkylated vinyl diazoacetate **8** is much more reactive than vinyl diazoacetate **1 b** towards cyclization, supported by a small energy difference between **TS_3_** and **8** (4.7 kcal mol^−1^). Indeed, the C_arylester_−N_diazoester_ bond strength in compound **9** is calculated to be 26.7 kcal mol^−1^ whereas the same bond in **8** is much weaker with Δ*H*_rxn_=1.3 kcal mol^−1^ (Figure 2, insert). As such, the relatively low activation barrier to the cyclization via transformation **8**→**9** can be explained by strengthening of the C_arylester_−N_diazoester_ bond upon moving from **8** to **9** acting as a driving force to facilitate the process. Heterocycle **9** then undergoes a deprotonation step aided by borate anion **6** to yield the pyrazole compound **10**. Notably, pyrazole **10** is the isomer of the obtained product in which the bis(aryl)methyl group is bonded to the nitrogen atom adjacent to the phenyl functionality. The overall energy barrier to generate the pyrazole isomer was calculated to be 21.3 kcal mol^−1^.

**Figure 2:**
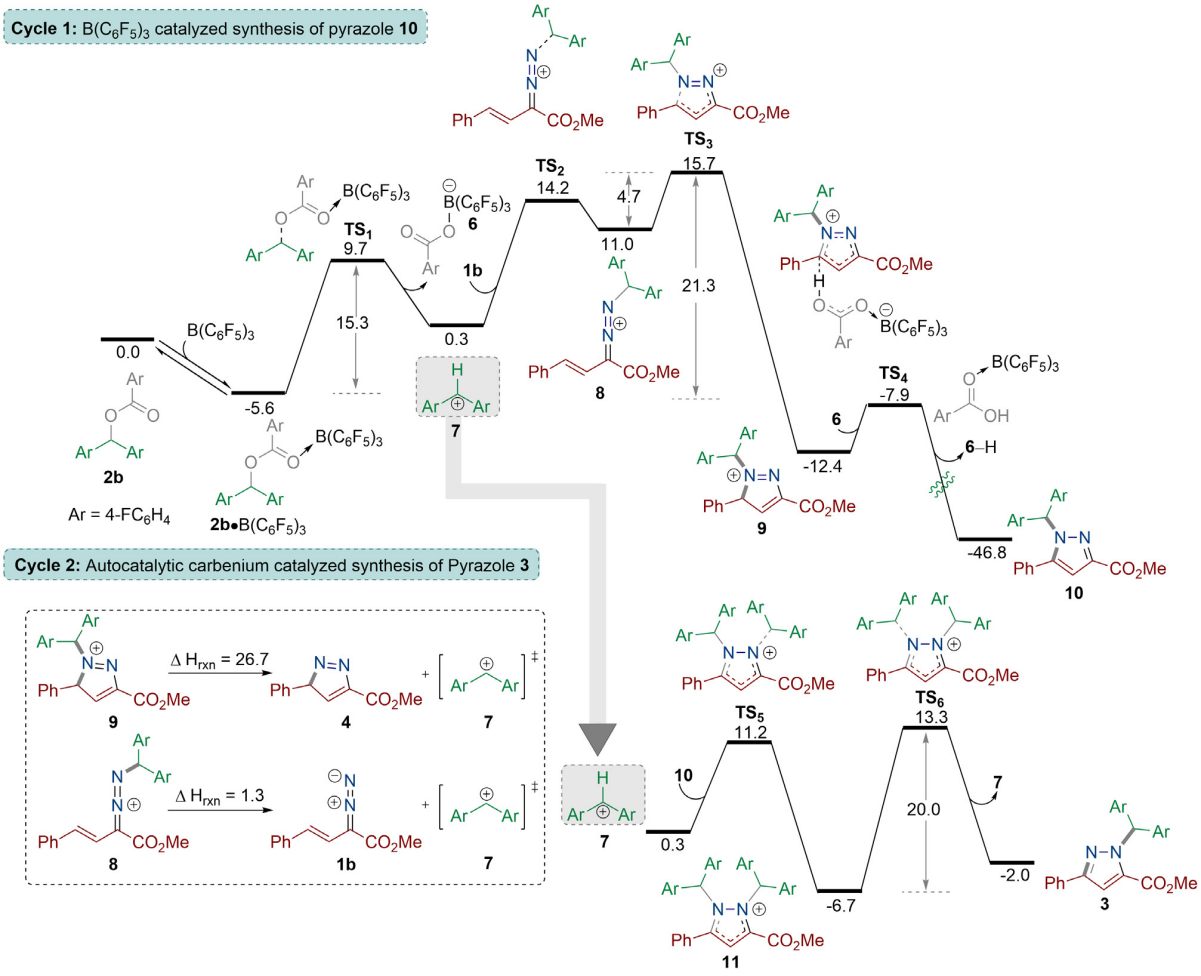
DFT‐computed reaction pathways for the formation of pyrazole‐alkylated compound from the reaction of methyl (*E*)‐2‐diazo‐4‐phenylbut‐3‐enoate (**1 b**) with aryl ester (**2 b**) calculated using the SMD/M06‐2X‐D3/def2‐TZVP//SMD/ M06‐2X/ 6‐31G(d) level of theory in CH_2_Cl_2_. Energies given in kcal mol^−1^. Ar=*p*‐FC_6_H_4_.

**Scheme 4:**
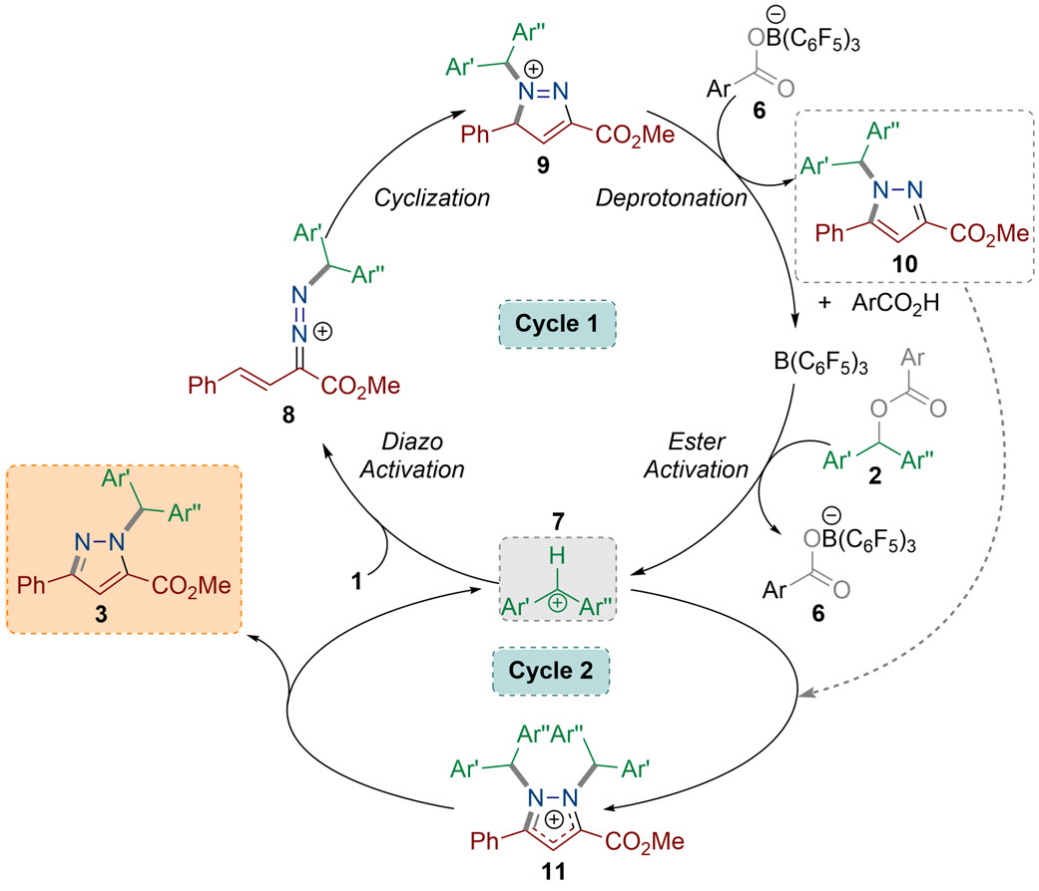
DFT‐based proposed reaction mechanism for the formation of pyrazole‐alkylated compound.

In cycle 2, the carbenium ion **7** acts as an autocatalyst to convert the kinetic pyrazole product **10** into the thermodynamic pyrazole isomer **3** with a barrier of 20.0 kcal mol^−1^.

Finally, we investigated the scope of the reaction to examine if this process was general to other aryl esters and vinyl diazoacetates (Scheme 5). Initially, we synthesized three vinyl diazoacetates according to literature report^[14]^ bearing electron withdrawing (*p*‐F: **1 a**), electron‐neutral (*p*‐H: **1 b**), and electron‐donating (*p*‐OMe: **1 c**) groups on the aryl ring. Likewise, several symmetrical diaryl esters were prepared bearing electron‐neutral (*p*‐H, **2 a**), electron‐withdrawing (*p*‐F, *p*‐CF_3_: **2 b** and **2 c**, respectively), and electron‐donating (*p*‐OMe, **2 d**) groups. The unsymmetrical esters containing naphthyl/methyl (**2 e**), phenyl/cyclohexyl (**2 f**), and 2‐isopropyl‐5‐methylcyclohexyl (**2 g**) were also synthesized. Each of the vinyl diazoacetates was reacted with all ester compounds in 1,2‐C_2_H_4_Cl_2_ for 20–22 h at 50 °C with catalytic B(C_6_F_5_)_3_ (10 mol %) generating **3 a**–**3 j** in good to excellent isolated yields (70–81 %). For all reaction products in Scheme 5, the ^1^H NMR spectra of the crude reaction mixture clearly showed the formation of only one regioisomer of the pyrazole being formed. Slow evaporation of a solution of **3 d** in CH_2_Cl_2_ afforded crystals suitable for single‐crystal X‐ray diffraction measurement (Figure 1).

**Scheme 5:**
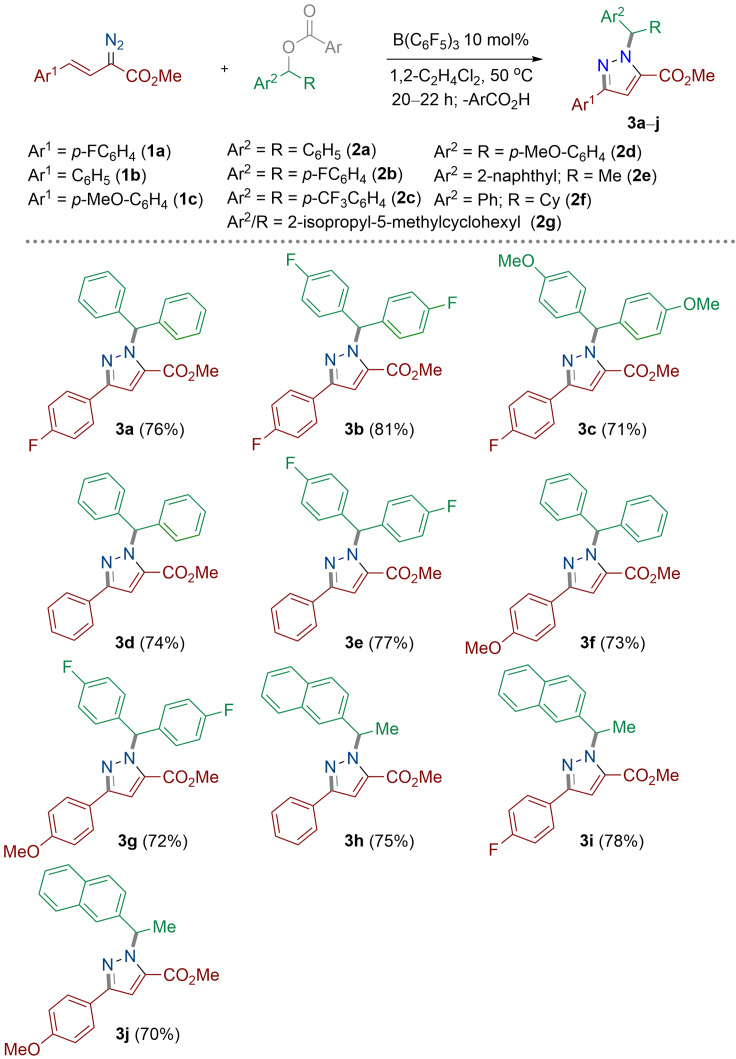
Substrate scope for the reaction between vinyl diazoacetates (**1**) and aryl esters (**2**). All the reactions were carried out on a 0.1 mmol scale. Yields reported are isolated yields. Ar=*p*‐FC_6_H_4_.

Highly electron‐deficient ester (**2 c**) was unsuccessful in the reaction due to the reduced basicity of the ester and high activation barrier for the carbenium ion formation.^[13]^ Likewise, the non‐aromatic ester 2‐isopropyl‐5‐methylcyclohexyl 4‐fluoro benzoate (**2 g**) failed to react with vinyl diazoesters due to the instability of the carbenium ion formed following B(C_6_F_5_)_3_ activation. Strongly electron‐donating groups in both the vinyl diazoacetate (**1 c**) and diaryl ester (**2 d**) afforded complicated reaction mixtures and attempts to isolate any pure compound failed.

Given the low activation barrier (20.0 kcal mol^−1^) calculated for the conversion of pyrazole isomer **10** into **3**, we wondered if we could observe this isomerization experimentally. To examine this, we took product **3 e** and subjected it to the carbocation species **7** (Ar=C_6_H_5_) which was generated in situ from compound **2 a** (1 equiv) using 10 mol % B(C_6_F_5_)_3_ (Scheme 6). The reaction was carried out in 1,2‐C_2_H_4_Cl_2_ at 50 °C. By ^1^H NMR spectroscopy it was found that the starting material **3 e** and product **3 d** were formed in an approximate 1:1 ratio with a small amount of the minor isomer **10 d** being observed. After 22 h **3 e** was isolated in 17 % yield, and **3 d** was isolated as the major product in 49 % yield. The latter species was formed from the exchange between the two diarylmethylene groups through cycle 2 (Scheme 4, Figure 2). Interestingly, we also isolated the less thermodynamically stable isomer **10 d** in 16 % yield. We propose that compound **3 e** first reacts with in situ generated carbocation **7** (Ar=C_6_H_5_) to generate cationic intermediate **11** (Scheme 4xschr4). Loss of carbenium **7** (Ar=*p*‐FC_6_H_4_) leads to the kinetic isomer **10 d**. Reaction of **10 d** with carbenium ion **7** (Ar=C_6_H_5_) generates the thermodynamic isomer **3 d** as the major product. This observation supports our DFT‐based mechanism to account for the formation of pyrazole compounds.

**Scheme 6:**
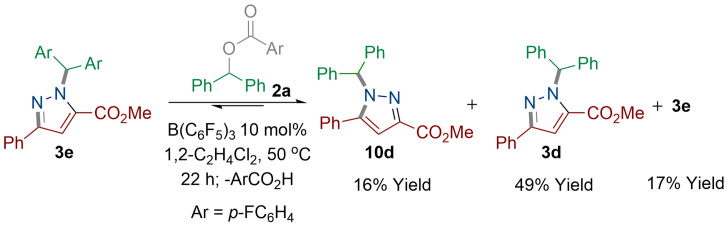
Reaction between **2 a** and **3 e** using 10 mol % B(C_6_F_5_)_3_. Yields reported are isolated yields.

In conclusion, we have demonstrated a new metal‐free synthetic approach for the preparation of regioselective N‐alkylated pyrazoles. A new reactivity pattern has been observed whereby catalytic B(C_6_F_5_)_3_ does not decompose the diazo compound allowing the N_2_ functionality to be exploited in the generation of N‐heterocycles. Detailed mechanistic studies were carried out to explain the mechanism for the reaction. DFT calculations revealed that catalytic amounts of B(C_6_F_5_)_3_ are required to activate the aryl ester generating a carbenium ion. Interestingly, this in situ generated carbenium species subsequently acts as an autocatalyst to promote the regioselective formation of N‐alkylated pyrazoles in good to excellent yields. This new reactivity pattern and metal‐free synthetic approach for the preparation of novel pyrazoles will have a broad impact in future applications towards the synthesis of biologically important molecules.

## Conflict of interest

The authors declare no conflict of interest.

## Supporting information

As a service to our authors and readers, this journal provides supporting information supplied by the authors. Such materials are peer reviewed and may be re‐organized for online delivery, but are not copy‐edited or typeset. Technical support issues arising from supporting information (other than missing files) should be addressed to the authors.

Supporting Information

Supporting Information

## Data Availability

Deposition numbers 2068715 (**3 a**) and 2095847 (**3 d**) contain the supplementary crystallographic data for this paper. These data are provided free of charge by the joint Cambridge Crystallographic Data Centre and Fachinformationszentrum Karlsruhe Access Structures service. Information about the data that underpins the results presented in this article, including how to access them, can be found in the Cardiff University data catalogue at https://doi.org/10.17035/d.2021.0140641037.
