# Supplementary material for: Tris(pentafluorophenyl)borane‐Catalyzed Carbenium Ion Generation and Autocatalytic Pyrazole Synthesis—A Computational and Experimental Study
Source: Angew Chem Int Ed Engl. 2021 Oct 12;60(46):24395–9. doi: 10.1002/anie.202109744 (PMC8596400; doi:10.1002/anie.202109744)
Supplement: Supplementary file 2 — Supporting Information [file ANIE-60-24395-s001.pdf]

Angewandte Chemie
Eine Zeitschrift der Gesellschaft Deutscher Chemiker

Supporting Information

# Tris(pentafluorophenyl)borane-Catalyzed Carbenium Ion Generation and Autocatalytic Pyrazole Synthesis—A Computational and Experimental Study

*Ayan Dasgupta[+], Rasool Babaahmadi[+], Sanjukta Pahar, Katarina Stefkova, Lukas Gierlichs, Brian F. Yates, Alireza Ariafard,* and Rebecca L. Melen**

anie_202109744_sm_miscellaneous_information.pdf
anie_202109744_sm_cif.zip

# Table of Content


| | | | |
|---|---|---|---|
| 1 | Experimental | | 3 |
| | 1.1 | General experimental | 3 |
| 1.2 | | Synthesis of vinyl diazoesters | 4 |
| | 1.2.1 | General procedure a | 4 |
| | 1.2.2 | Synthesis and spectral characterization of vinyl diazoesters | 4 |
| 1.3 | | Synthesis of aryl esters | 6 |
| | 1.3.1 | General procedure b | 6 |
| | 1.3.2 | Synthesis and spectral characterization of aryl esters | 6 |
| 2 | Product Characterization | | 10 |
| | 2.1 | General procedure c | 10 |
| | 2.2 | Synthesis and spectral characterization of products | 10 |
| 3 | NMR Spectra | | 17 |
| 4 | Crystallographic Data | | 82 |
| | 4.1 | Single crystal X-ray diffraction experimental | 82 |
| | 4.2 | Solid-state structure | 83 |
| | 4.3 | X-ray refinement data | 85 |
| 5 | Computational Data | | 87 |
| | 5.1 | Computational details | 87 |
| | 5.2 | Cartesian coordinates and total energies for the calculated structures | 88 |
| 6 | References | | 120 |

# 1. Experimental

## 1.1 General experimental:

Except for the starting materials, all reactions and manipulations were carried out under an atmosphere of dry, $O_2$-free nitrogen using standard double-manifold techniques with a rotary oil pump. A nitrogen-filled glove box (MBraun) was used to manipulate solids including the storage of starting materials, ambient temperature reactions, product recovery and sample preparation for analysis. All solvents (toluene, tetrahydrofuran, dichloromethane, hexane, diethyl ether, acetonitrile) were dried by employing a Grubbs-type column system (Innovative Technology) or a solvent purification system MB SPS-800 and stored under a nitrogen atmosphere. Anhydrous (with Sure/Seal) 1,2-dichloroethane (1,2-$C_2H_4Cl_2$) was purchased from Merck and dried over molecular sieves before use. Deuterated solvents were distilled and/or dried over molecular sieves before use. Chemicals were purchased from commercial suppliers and used as received. All the triarylfluoroboranes were prepared as per the standard literature report.[1] Thin-layer chromatography (TLC) was performed on pre-coated aluminium sheets of Merck silica gel 60 F254 (0.20 mm). $^{1}H$, $^{13}C$, and $^{19}F$ NMR spectra were recorded on a Bruker Avance II 400 or Bruker Avance 500 spectrometer. All coupling constants are absolute values and are expressed in Hertz (Hz). $^{13}C$ NMR was measured as $^{1}H$ decoupled. Yields are given as isolated yields. Chemical shifts are expressed as parts per million (ppm, δ) downfield of tetramethylsilane (TMS) and are referenced to $CDCl_3$ (7.26/77.16 ppm) as internal standard. NMR spectra were referenced to $CFCl_3$ ($^{19}F$).[2] The description of signals includes s = singlet, d = doublet, t = triplet, q = quartet, and m = multiplet, br. = broad. All coupling constants are absolute values and are expressed in Hertz (Hz). $^{13}C$ NMR was measured as $^{1}H$ decoupled. Yields are given as isolated yields. All spectra were analyzed assuming a first order approximation. IR-Spectra were measured on a Shimadzu IRAffinity-1 photo-spectrometer. Mass spectra were measured on a Waters LCT Premier/XE or a Waters GCT Premier spectrometer. Ions were generated by the Atmospheric Solids, Analysis Probe (ASAP), Electrospray (ES) or Electron Ionisation (EI). The molecular ion peaks values quoted for either molecular ion ($M^+$), molecular ion plus or minus hydrogen ($M+H^+$, $M-H^-$), molecular ion plus sodium ($M+Na^+$).

## 1.2 Synthesis of vinyl diazoesters:

**1.2.1 General Procedure a**: In an oven dried 500 mL Schlenk flask 2-carboxyethyltriphenylphosphonium chloride (1.2 equiv) was placed and the flask was evacuated, back filled with dry nitrogen gas three times. Dry THF (100 mL) was added to the flask followed by the addition of aryl aldehyde (1 equiv) at room temperature. The flask was cooled to 0 °C using an ice-water bath and the reaction mixture was stirred vigorously. A THF solution (50 mL) of potassium *tert*butoxide (2.5 equiv) was added dropwise to the reaction mixture at 0 °C. After one hour of stirring at 0 °C under an $N_2$ atmosphere, the reaction mixture was stirred at room temperature for additional 30 min. Dimethyl sulfate (2.0 equiv) was added to the reaction mixture with vigorous stirring. After 3 h, the reaction mixture was cooled to 0 °C and *para*-acetamidobenzenesulfonyl azide (*p*-ABSA, 1.3 equiv) was added followed by rapid addition of 1,8-diazabicyclo[5.4.0]undec-7-ene (DBU, 1.5 equiv). After 4 h of stirring at 0 °C, the reaction mixture was stirred at room temperature for an additional hour. Subsequently the THF was removed under vacuum. The organic compounds were extracted with diethyl ether (3 × 100 mL), the combined organic fractions were washed with brine solution (1 × 100 mL), dried over $MgSO_4$ and concentrated using vacuum. The crude compound was purified *via* a quick column chromatography using silica gel (Merck, 60 Å, 230–400 mesh particle size) and hexane//diethyl ether as eluent. The pure compounds were stored at -30 °C.

### 1.2.2 Synthesis and spectral characterization of vinyl diazoesters:

*Synthesis of methyl (E)-2-diazo-4-(4-fluorophenyl)but-3-enoate* (**1a**)[3]

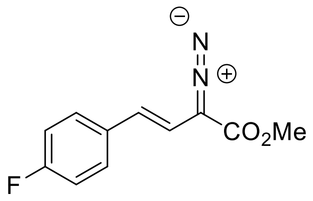


Synthesized in accordance with *General Procedure a* using 2-carboxyethyltriphenylphosphonium chloride (7.1 g, 19.3 mmol), 4-fluorobenzaldehyde (2 g, 16.1 mmol), potassium *tert*butoxide (4.5 g, 40.2 mmol), dimethyl sulfate (3.1 mL, 32.2 mmol), *p*-ABSA (5 g, 20.9 mmol), and DBU (3.1 mL, 24.2 mmol). All volatiles were removed *in vacuo* and the crude compound was purified *via* column chromatography using silica gel and hexane/diethyl ether (95:5 v/v) as eluent: The desired product **1a** was obtained as a red solid. Yield: 2.6 g, 11.8 mmol, 73%.

$^1$H NMR (400 MHz, $CDCl_3$, 298 K) δ: 7.32–7.28 (m, 2H, Ar–CH), 7.02–6.97 (m, 2H, Ar–CH), 6.37 (d, $J$ = 16.1 Hz, 1H, $CH_2$), 6.16 (d, $J$ = 16.3 Hz, 1H, $CH_2$), 3.84 (s, 3H, $CO_2$Me); $^{13}$C NMR (101 MHz, $CDCl_3$, 298 K) δ: 165.6 (C=O), 162.1 (d, $J_{C–F}$ = 246.9 Hz), 133.2 (d, $J_{C–F}$ = 3.4 Hz), 127.4 (d, $J_{C–F}$ = 7.9 Hz), 122.1, 115.7 (d, $J_{C–F}$ = 21.7 Hz), 111.2 (d, $J_{C–F}$ = 2.4 Hz), 52.4 ($CO_2$Me); $^{19}$F NMR (376 MHz, $CDCl_3$, 298 K) δ: -114.91 (s, 1F, Ar–F).

*Synthesis of methyl (E)-2-diazo-4-phenylbut-3-enoate* (**1b**)[3]

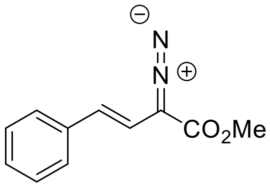


In an oven dried Schlenk flask, methyl (*E*)-4-phenylbut-3-enoate (2.5 g, 14.2 mmol, 1 equiv) was dissolved in anhydrous acetonitrile (120 mL. *p*-ABSA (5.1 g, 21.3 mmol, 1.5 equiv) was added to the reaction mixture at 0 °C under an $N_2$-atmosphere followed by the dropwise addition of DBU (3.2 mL, 21.3 mmol, 1.5 equiv). The mixture was slowly warmed up to room temperature (23 °C) and stirred overnight at ambient temperature under a $N_2$ atmosphere. The reaction mixture was quenched with saturated $NH_4Cl$ (aq) solution (100 mL). The aqueous layer was washed with diethyl ether (3 × 150 mL) and dried over anhydrous $MgSO_4$. All volatiles were removed *in vacuo* and the crude compound was purified *via* column chromatography using silica gel (Merck, 60 Å, 230‒400 mesh particle size) and hexane/diethyl ether (98:2 v/v) as eluent. The desired product **1b** was obtained as a red oil. Yield: 2.3 g, 11.2 mmol, 79%.

$^1$H NMR (400 MHz, $CDCl_3$, 298 K) δ: 7.25–7.17 (m, 4H, Ar–CH), 7.10–7.08 (m, 1H, Ar–CH), 6.37 (d, *J* = 16.3 Hz, 1H, $CH_2$), 6.08 (d, *J* = 16.3 Hz, 1H, $CH_2$), 3.72 (s, 3H, $CO_2Me$); $^{13}$C NMR (101 MHz, $CDCl_3$, 298 K) δ 165.6 (C=O), 136.8, 128.7, 127.1, 125.9, 123.1, 111.3, 52.3 ($CO_2Me$).

*Synthesis of methyl (E)-2-diazo-4-(4-methoxyphenyl)but-3-enoate* (**1c**)[3]

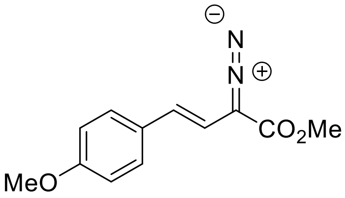


Synthesized in accordance with *General Procedure a* using 2-carboxyethyltriphenylphosphonium chloride (6.5 g, 17.6 mmol), 4-methoxybenzaldehyde (2 g, 14.7 mmol), potassium *tert*butoxide (4.1 g, 36.7 mmol), dimethyl sulfate (2.8 mL, 29.4 mmol), *p*-ABSA (4.6 g, 19.1 mmol) and DBU (2.8 mL, 22 mmol). All volatiles were removed *in vacuo* and the crude compound was purified *via* column chromatography using silica gel and hexane/diethyl ether (93:7 v/v) as eluent: The desired product **1c** was obtained as a red solid. Yield: 2.4 g, 10.4 mmol, 70%.

$^1$H NMR (400 MHz, $CDCl_3$, 298 K) δ: 7.22 (dd, *J* = 8.7, 1.8 Hz, 2H, Ar–CH), 6.81–6.77 (m, 2H, Ar–CH), 6.22 (d, *J* = 16.3 Hz, 1H, $CH_2$), 6.07 (d, *J* = 16.6 Hz, 1H, $CH_2$), 3.77 (s, 3H, OMe), 3.73 (s, 3H, $CO_2Me$); $^{13}$C NMR (101 MHz, $CDCl_3$, 298 K) δ: 166.0 (C=O), 160.2, 159.1, 129.9, 127.2, 123.0, 114.5, 114.3, 108.8, 55.4 (OMe), 52.4 ($CO_2Me$).

**1.3 Synthesis of aryl esters:**

**1.3.1 General Procedure b:** Acyl chloride (1.2 equiv) and alcohol (1 equiv) were dissolved in pyridine at 0 °C. The mixture was stirred at ambient temperature overnight. The reaction was quenched with water and extracted with ethyl acetate (3 × 25 mL). The combined organic fractions were washed with brine solution (1 × 25 mL) and dried over $MgSO_4$. All volatiles were removed *in vacuo* and the crude compound was purified *via* column chromatography using silica gel (Merck, 60 Å, 230−400 mesh particle size) and hexane/ethyl acetate as eluent.

**1.3.2 Synthesis and spectral characterization of aryl esters:**

*Synthesis of benzhydryl 4-fluorobenzoate* (**2a**)[4]

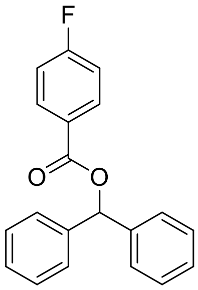


Synthesized in accordance with *General Procedure b* using 4-fluorobenzoyl chloride (2.7 mL, 23.2 mmol), diphenylmethanol (3.68 g, 20.0 mmol), and pyridine (25 mL). All volatiles were removed *in vacuo* and the crude product was purified *via* column chromatography using silica gel and hexane/ethyl acetate (95:5 v/v) as eluent. The desired product **2a** was obtained as a white solid. Yield: 5.49 g, 17.8 mmol, 89%.

$^1$H NMR (500 MHz, $CDCl_3$, 298 K) δ: 8.18–8.15 (m, 2H, Ar–CH), 7.44–7.42 (m, 4H, Ar–CH), 7.38–7.34 (m, 4H, Ar–CH), 7.32–7.29 (m, 2H, Ar–CH), 7.15–7.11 (m, 3H, Ar–CH and CH); $^{13}$C NMR (126 MHz, $CDCl_3$, 298 K) δ: 166.0 (d, $J_{C–F}$ = 254.3 Hz), 164.7 (C=O), 143.9, 140.2, 132.5 (d, $J_{C–F}$ = 9.3 Hz), 128.7, 128.6, 127.7, 127.2, 126.6, 126.6, 126.5, 115.8 (d, $J_{C–F}$ = 22.0 Hz), 77.7 (CH); $^{19}$F NMR (471 MHz, $CDCl_3$, 298 K) δ: -105.19 (s, 1F, Ar–F); IR $\nu_{max}$ (cm$^{-1}$): 3026, 3030, 1716 (C=O), 1598, 1504, 1454, 1411, 1361, 1294, 1184, 1105, 1089, 1014; HRMS (EI+) $[M]^+$ calculated for $[C_{20}H_{15}O_2F]^+$: 306.1056, found: 306.1056.

*Synthesis of bis(4-fluorophenyl) methyl 4-fluorobenzoate* (**2b**)[4]

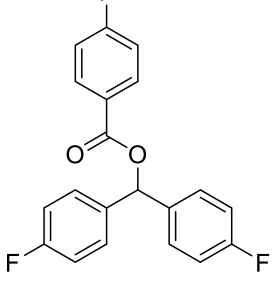


Synthesized in accordance with *General Procedure b* using 4-fluorobenzoyl chloride (2.7 mL, 23.2 mmol.), bis(4-fluorophenyl) methanol (4.4 g, 20.0 mmol), and pyridine (25 mL). All volatiles were removed *in vacuo* and the crude compound was purified *via* column chromatography using silica gel and hexane/ethyl acetate (95:5 v/v) as eluent: The desired product **2b** was obtained as a white solid. Yield: 5.81 g, 17.0 mmol, 85%.

$^{1}$H NMR (400 MHz, $CDCl_3$, 298 K) δ: 8.15–8.12 (m, 2H, Ar–CH), 7.39–7.36 (m, 4H, Ar–CH), 7.16–7.12 (m, 2H, Ar–CH), 7.08–7.04 (m, 5H, Ar–CH and CH); $^{13}$C NMR (101 MHz, $CDCl_3$, 298 K) δ: 166.1 (d, $J_{C–F}$ = 254.7 Hz), 164.8 (C=O), 162.6 (d, $J_{C–F}$ = 247.3 Hz), 135.8 (d, $J_{C–F}$ = 3.3 Hz), 132.4 (d, $J_{C–F}$ = 9.4 Hz), 129.0 (d, $J_{C–F}$ = 8.3 Hz), 126.2 (d, $J_{C–F}$ = 3.0 Hz), 115.8 (d, $J_{C–F}$ = 22.2 Hz), 115.7 (d, $J_{C–F}$ = 21.6 Hz), 76.4 (CH); $^{19}$F NMR (376 MHz, $CDCl_3$, 298 K) δ: -104.80 (s, 1F, Ar–F), -113.67 (s, 2F, Ar–F); IR $\nu_{max}$ (cm$^{-1}$): 3116, 3074, 1724 (C=O), 1602, 1504, 1413, 1340, 1301, 1265, 1186, 1099; HRMS (EI+) $[M]^+$ calculated for $[C_{20}H_{13}O_2F_3]^+$: 342.0868, found: 342.0871.

*Synthesis of bis(4-(trifluoromethyl)phenyl)methyl 4-fluorobenzoate* (**2c**)[4]

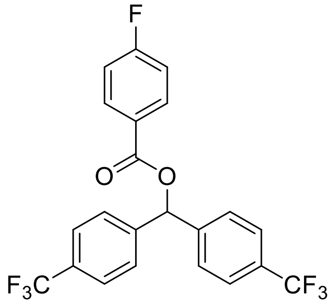


Synthesized in accordance with *General Procedure b* using 4-fluorobenzoyl chloride (1.4 mL, 13.1 mmol), bis(4-(trifluoromethyl)phenyl)methanol (3.5 g, 10.9 mmol), and pyridine (20 mL). All volatiles were removed *in vacuo* and the crude compound was purified *via* column chromatography using silica gel and hexane/ethyl acetate (20:1 v/v) as eluent: The desired product **2c** was obtained as a white solid. Yield: 4.24 g, 9.6 mmol, 88%.

$^{1}$H NMR (500 MHz, $CDCl_3$, 298 K) δ: 8.15 (dd, $J$ = 8.8, 5.4 Hz, 2H, Ar–CH), 7.65 (d, $J$ = 8.2 Hz, 4H, Ar–CH), 7.54 (d, $J$ = 8.0 Hz, 4H, Ar–CH), 7.18–7.14 (m, 3H, Ar–CH and CH); $^{13}$C NMR (126 MHz, $CDCl_3$, 298 K) δ: 166.2 (d, $J_{C–F}$ = 255.3 Hz), 164.4 (C=O), 143.2, 132.5 (d, $J_{C–F}$ = 9.4 Hz), 130.7 (q, $J_{C–F}$ = 32.7 Hz), 127.5, 126.0 (q, $J_{C–F}$ = 3.7 Hz), 125.7 (d, $J_{C–F}$ = 3.0 Hz), 123.9 (q, $J_{C–F}$ = 272.3 Hz), 116.0 (d, $J_{C–F}$ = 22.0 Hz), 76.3 (CH); $^{19}$F NMR (471 MHz, $CDCl_3$, 298 K) δ: -62.73 (s, 6F, $CF_3$), -104.17 (s, 1F, Ar–F); IR $\nu_{max}$ (cm$^{-1}$): 1724 (C=O), 1620, 1605, 1508, 1413, 1323, 1259, 1239, 1168, 1154, 1128, 1112, 1089, 1066, 1018; HRMS (ASAP-) $[M-H]^-$ calculated for $[C_{22}H_{12}O_2F_7]^-$: 441.0726, found: 441.0724.

*Synthesis of bis(4-methoxyphenyl) methyl 4-fluorobenzoate* (**2d**)[4]

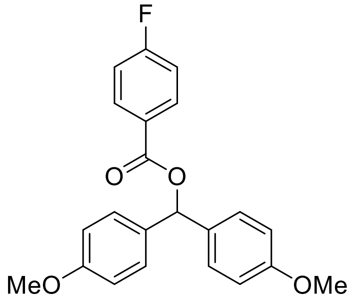


Synthesized in accordance with *General Procedure b* using 4-fluorobenzoyl chloride (2.7 mL, 23.2 mmol), bis(4-methoxyphenyl)methanol (4.86 g, 20.0 mmol), and pyridine (25 mL). All volatiles were removed *in vacuo* and the crude product was purified *via* column chromatography using silica gel and hexane/ethyl acetate (90:10 v/v) as eluent. The desired product **2d** was obtained as a colorless oil. Yield: 5.46 g, 14.9 mmol, 76%.

$^1$H NMR (500 MHz, $CDCl_3$, 298 K) δ: 8.13 (dd, $J$ = 8.9, 5.4 Hz, 2H, Ar–CH), 7.39–7.29 (m, 4H, Ar–CH), 7.12 (td, $J$ = 8.5, 1.4 Hz, 2H, Ar–CH), 7.04 (s, 1H, CH), 6.95–6.86 (m, 4H, Ar–CH), 3.80 (s, 6H, OMe); $^{13}$C NMR (126 MHz, $CDCl_3$, 298 K) δ: 165.9 (d, $J_{C–F}$ = 254.0 Hz), 164.8 (C=O), 159.4, 132.6, 132.4 (d, $J_{C–F}$ = 9.3 Hz), 128.6, 126.7 (d, $J_{C–F}$ = 3.0 Hz), 115.6 (d, $J_{C–F}$ = 22.0 Hz), 114.0, 76.9 (CH), 55.4 (OMe); $^{19}$F NMR (471 MHz, $CDCl_3$, 298 K) δ: -105.46 (s, 1F, Ar–F); IR $\nu_{max}$ ($cm^{-1}$): 3116, 3074, 1724 (C=O), 1602, 1413, 1340, 1301, 1263, 1186, 1099, 1014; HRMS (ES+) $[M+Na]^+$ calculated for $[C_{22}H_{19}O_4FNa]^+$: 389.1165, found: 389.1166.

*Synthesis of 1-(naphthalen-2-yl)ethyl 4-fluorobenzoate* (**2e**)[4]

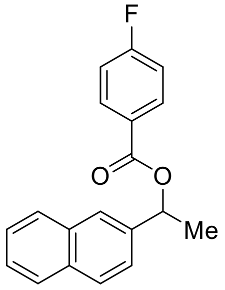


Synthesized in accordance with *General Procedure b* using 4-fluorobenzoyl chloride (2.3 mL, 20.2 mmol), 1-(naphthalen-2-yl)ethan-1-ol (3.17 g, 18.4 mmol), and pyridine (15 mL). All volatiles were removed *in vacuo* and the crude compound was purified *via* column chromatography using silica gel and hexane/ ethyl acetate (95:5 v/v) as eluent. The desired product **2e** was obtained as a while solid. Yield: 2 g, 6.8 mmol, 37%.

$^1$H NMR (500 MHz, $CDCl_3$, 298 K) δ: 8.16–8.10 (m, 2H, Ar–CH), 7.92–7.81 (m, 4H, Ar–CH), 7.58 (dd, $J$ = 8.5, 1.8 Hz, 1H, Ar–CH), 7.53–7.46 (m, 2H, Ar–CH), 7.16–7.09 (m, 2H, Ar–CH), 6.30 (q, $J$ = 6.6 Hz, 1H, CH), 1.77 (d, $J$ = 6.6 Hz, 3H, Me); $^{13}$C NMR (126 MHz, $CDCl_3$, 298 K) δ: 165.9 (d, $J_{C–F}$ = 254.5 Hz), 165.0 (C=O), 139.0, 133.2 (d, $J_{C–F}$ = 13.8 Hz), 132.3 (d, $J_{C–F}$ = 8.8 Hz), 128.6, 128.1, 127.8, 126.3 (d, $J_{C–F}$ = 18.9 Hz), 125.2, 124.1, 115.6 (d, $J_{C–F}$ = 22.0 Hz), 73.4 (CH), 22.4 (Me); $^{19}$F NMR (471 MHz, $CDCl_3$, 298 K) δ: -105.69 (s, 1F, Ar–F); HRMS ($EI^+$) $[M^+]$ $[C_{19}H_{15}O_2F]^+$: calculated 294.1056, found: 294.1061.

*Synthesis of cyclohexyl(phenyl)methyl 4-fluorobenzoate* (**2f**)[4]

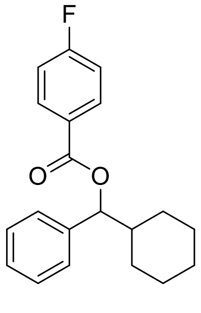


Synthesized in accordance with *General Procedure b* using 4-fluorobenzoyl chloride (3.4 mL, 28.9 mmol), cyclohexyl(phenyl)methanol (5 g, 26.3 mmol), and pyridine (18 mL). All volatiles were removed *in vacuo* and the crude compound was purified *via* column chromatography using silica gel and hexane/ethyl acetate (95:5 v/v) as eluent. The desired product **2f** was obtained as a white solid. Yield: 5.4 g, 17.4 mmol, 66%.

$^1$H NMR (500 MHz, $CDCl_3$, 298 K) δ: 8.12–8.08 (m, 2H, Ar–CH), 7.37–7.32 (m, 4H, Ar–CH), 7.29–7.26 (m, 1H, Ar–CH), 7.13–7.10 (m, 2H, Ar–CH), 5.73 (d, $J$ = 7.5 Hz, 1H, CH), 1.95–1.88 (m, 2H, $CH_2$), 1.78–1.65 (m, 3H, $CH_2$), 1.50–1.48 (m, 1H, CH), 1.29–1.12 (m, 4H, $CH_2$),

1.02 (qd, $J$ = 12.3, 3.8 Hz, 1H, CH); $^{13}$C NMR (126 MHz, $CDCl_3$, 298 K) δ: 165.8 (d, $J_{C–F}$ = 254.5 Hz), 165.0 (C=O), 139.7, 132.2 (d, $J_{C–F}$ = 10.0 Hz), 128.3, 127.9, 127.1, 126.9 (d, $J_{C–F}$ = 3.0 Hz), 115.6 (d, $J_{C–F}$ = 22.6 Hz), 81.1 (CH), 43.3 (CH), 29.2 ($CH_2$), 29.1 ($CH_2$), 26.4 ($CH_2$), 26.05 ($CH_2$), 26.00 ($CH_2$); $^{19}$F NMR (471 MHz, $CDCl_3$, 298 K) δ: -105.88 (s, 1F, Ar–F); IR $\nu_{max}$ ($cm^{-1}$): 2939, 2848, 1720 (C=O), 1600, 1504, 1448, 1292, 1253, 1238, 1219, 1153, 1107, 1087, 1053, 1012; HRMS (EI+) $[M]^+$ $[C_{20}H_{21}O_2F]^+$: calculated 312.1526, found 312.1519.

*Synthesis of 2-isopropyl-5-methylcyclohexyl 4-fluorobenzoate* (**2g**)

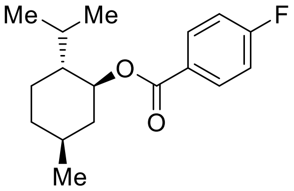


Synthesized in accordance with *General Procedure b* using 4-fluorobenzoyl chloride (4.5 mL, 38.4 mmol), L(-)-menthol (5 g, 32.0 mmol), and pyridine (18 mL). All volatiles were removed *in vacuo* and the crude compound was purified *via* column chromatography using silica gel and hexane/ethyl acetate (95:5 v/v) as eluent. The desired product **2g** was obtained as a white solid. Yield: 7.1 g, 25.9 mmol, 81%.

$^{1}$H NMR (500 MHz, $CDCl_3$, 298 K) δ: 8.45–7.69 (m, 2H, Ar–CH), 7.19–6.90 (m, 2H, Ar–CH), 4.92 (td, $J$ = 10.9, 4.4 Hz, 1H, CH), 2.11 (m, 1H, CH), 1.94 (m, 1H, CH), 1.81–1.66 (m, 2H, $CH_2$), 1.60–1.47 (m, 2H, $CH_2$), 1.20–1.08 (m, 2H, $CH_2$), 0.92 (dd, $J$ = 6.8, 5.5 Hz, 6H, $CH_3$), 0.79 (d, $J$ = 7.0 Hz, 3H, Me); $^{13}$C NMR (126 MHz, $CDCl_3$, 298 K) δ: 165.7 (d, $J_{C–F}$ = 254.5 Hz), 165.3 (C=O), 164.8, 132.2 (d, $J_{C–F}$ = 9.2 Hz), 127.2, 115.5 (d, $J_{C–F}$ = 21.9 Hz), 75.15, 47.4, 41.1, 34.4, 31.6, 26.7, 23.7, 22.2, 20.9, 16.6; $^{19}$F NMR (471 MHz, $CDCl_3$, 298 K) δ: -106.28 (s, 1F, Ar–F); HRMS (EI+) $[M]^+$ $[C_{17}H_{23}FO_2]^+$: calculated 278.1682, found 278.1686.

## 2. Product Characterization

**2.1 General Procedure c:** Tris(pentafluorophenyl)borane [$B(C_6F_5)_3$] (10 mol%) was dissolved in 1,2 dichloroethane/1,2-$C_2H_4Cl_2$ (0.5 mL) and added to a 1,2- $C_2H_4Cl_2$ solution (0.5 mL) of the aryl ester (1.3 equiv). The vinyl diazoester (1 equiv) was also dissolved in 1,2- $C_2H_4Cl_2$ (0.5 mL) separately and added dropwise to the reaction mixture. The reaction tube was sealed under a nitrogen atmosphere and heated at 50 °C for 20–22 h. All volatiles were removed *in vacuo* and the crude compound was purified *via* preparative thin layer chromatography using hexane/ethyl acetate as eluent.

**2.2 Synthesis and spectral characterization of products:**

*Synthesis of methyl 1-benzhydryl-3-(4-fluorophenyl)-1H-pyrazole-5-carboxylate* (**3a**)

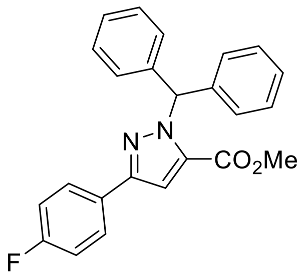


Synthesized in accordance with *General Procedure c* using $B(C_6F_5)_3$ (5 mg, 0.01 mmol), vinyl diazoester **1a** (22 mg, 0.10 mmol), and aryl ester **2a** (40 mg, 0.13 mmol) in 1,2-$C_2H_4Cl_2$ to afford **3a**. The crude reaction mixture was purified *via* preparative thin layer chromatography using hexane/ethyl acetate (95:5 v/v) as eluent. The desired compound **3a** was obtained as a white solid. Yield: 29 mg, 0.08 mmol, 76%.

$^1$H NMR (500 MHz, $CDCl_3$, 298 K) δ: 7.88 (s, 1H, CH), 7.76–7.73 (m, 2H, Ar–CH), 7.34–7.28 (m, 10H, Ar–CH), 7.16 (s, 1H, CH), 7.07–7.04 (m, 2H, Ar–CH), 3.90 (s, 3H, $CO_2Me$); $^{13}$C NMR (126 MHz, $CDCl_3$, 298 K) δ: 162.8 (d, $J_{C–F}$ = 246.8 Hz), 160.4 (C=O), 149.5, 140.1, 134.0, 128.9, 128.4, 127.8, 127.5 (d, $J_{C–F}$ = 8.1 Hz), 115.6 (d, $J_{C–F}$ = 21.7 Hz), 108.2, 66.5 (CH), 52.2 ($CO_2Me$); $^{19}$F NMR (471 MHz, $CDCl_3$, 298 K) δ: -114.02 (s, 1F, Ar–F); IR $\nu_{max}$ ($cm^{-1}$): 3031, 2984, 1721 (C=O), 1606, 1544, 1510, 1257, 1221, 1156, 1088; HRMS (ES+) $[M+H]^+$ $[C_{24}H_{20}FN_2O_2]^+$: calculated 387.1509, found 387.1496.

*Synthesis of methyl 1-(bis(4-fluorophenyl)methyl)-3-(4-fluorophenyl)-1H-pyrazole-5-carboxylate* (**3b**)

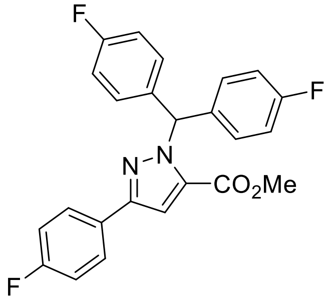


Synthesized in accordance with *General Procedure c* using $B(C_6F_5)_3$ (5 mg, 0.01 mmol), vinyl diazoester **1a** (22 mg, 0.10 mmol), and aryl ester **2b** (45 mg, 0.13 mmol) in 1,2-$C_2H_4Cl_2$ to afford **3b**. The crude reaction mixture was purified *via* preparative thin layer chromatography using hexane/ethyl acetate (95:5 v/v) as

eluent. The desired compound **3b** was obtained as a white solid. Yield: 34 mg, 0.08 mmol, 81%.

$^1$H NMR (500 MHz, $CDCl_3$, 298 K) δ: 7.93 (s, 1H, CH), 7.84–7.81 (m, 2H, Ar–CH), 7.38–7.34 (m, 4H, Ar–CH), 7.19–7.10 (m, 7H, Ar–CH, CH), 4.01 (s, 3H, $CO_2Me$); $^{13}$C NMR (126 MHz, $CDCl_3$, 298 K) δ: 162.9 (d, $J_{C–F}$ = 247.2 Hz), 162.4 (d, $J_{C–F}$ = 246.9 Hz), 160.4 (C=O), 149.8, 135.8 (d, $J_{C–F}$ = 3.3 Hz), 133.9, 130.5 (d, $J_{C–F}$ = 8.2 Hz), 128.8 (d, $J_{C–F}$ = 3.2 Hz), 127.5 (d, $J_{C–F}$ = 8.1 Hz), 115.7 (d, $J_{C–F}$ = 21.7 Hz), 115.4 (d, $J_{C–F}$ = 21.5 Hz), 108.3, 65.2 (CH), 52.3 ($CO_2Me$); $^{19}$F NMR (471 MHz, $CDCl_3$, 298 K) δ: -113.68 (s, 1F, Ar–F); -114.46 (s, 2F, Ar–F); IR $\nu_{max}$ (cm$^{-1}$): 3031, 2922, 1723 (C=O), 1633, 1656, 1515, 1444, 1356, 1257, 1123, 1088; HRMS (ES+) $[M+H]^+$ $[C_{24}H_{18}F_3N_2O_2]^+$: calculated 423.1320, found 423.1315.

*Synthesis of methyl 1-(bis(4-methoxyphenyl)methyl)-3-(4-fluorophenyl)-1H-pyrazole-5-carboxylate* (**3c**)

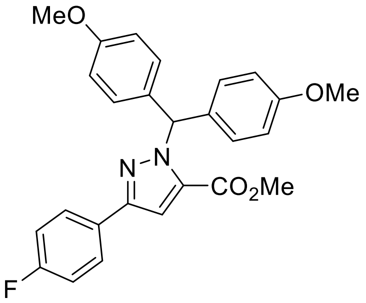


Synthesized in accordance with *General Procedure c* using $B(C_6F_5)_3$ (5 mg, 0.01 mmol), vinyl diazoester **1a** (22 mg, 0.10 mmol), and aryl ester **2d** (48 mg, 0.13 mmol) in 1,2-$C_2H_4Cl_2$ to afford **3c**. The crude reaction mixture was purified *via* preparative thin layer chromatography using hexane/ethyl acetate (92:8 v/v) as eluent. The desired compound **3c** was obtained as a white solid. Yield: 32 mg, 0.07 mmol, 71%.

$^1$H NMR (500 MHz, $CDCl_3$, 298 K) δ: 7.76–7.72 (m, 3H, Ar–CH, CH), 7.25–7.21 (m, 4H, Ar–CH), 7.13 (s, 1H, CH), 7.09–7.03 (m, 2H, Ar–CH), 6.86–6.83 (m, 4H, Ar–CH), 3.89 (s, 3H, $CO_2Me$), 3.78 (s, 6H, OMe); $^{13}$C NMR (126 MHz, $CDCl_3$, 298 K) δ: 162.8 (d, $J_{C–F}$ = 246.7 Hz), 160.5 (C=O), 159.1, 149.4, 133.8, 132.7, 130, 127.5 (d, $J_{C–F}$ = 8.1 Hz), 115.6 (d, $J_{C–F}$ = 21.6 Hz), 113.7, 108.2, 65.7 (CH), 55.4 (OMe), 52.2 ($CO_2Me$); $^{19}$F NMR (471 MHz, $CDCl_3$, 298 K) δ: -114.15 (s, 1F, Ar–F); IR $\nu_{max}$ (cm$^{-1}$): 3021, 2899, 1717 (C=O), 1654, 1509, 1444, 1275, 1248, 1220, 1156, 1089; HRMS (ES+) $[M+Na]^+$ $[C_{26}H_{23}FN_2O_4Na]^+$: calculated 469.1540, found 469.1537.

*Synthesis of methyl 1-benzhydryl-3-phenyl-1H-pyrazole-5-carboxylate* (**3d**)

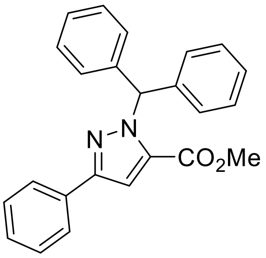


Synthesized in accordance with *General Procedure c* using $B(C_6F_5)_3$ (5 mg, 0.01 mmol), vinyl diazoester **1b** (20 mg, 0.10 mmol), and aryl ester **2a** (40 mg, 0.13 mmol) in 1,2-$C_2H_4Cl_2$ to afford **3d**. The crude reaction mixture was purified *via* preparative thin layer chromatography using hexane/ethyl acetate (95:5 v/v) as eluent. The desired compound **3d** was obtained as a white solid. Yield: 27 mg, 0.07 mmol, 74%.

$^1$H NMR (500 MHz, $CDCl_3$, 298 K) δ: 7.88 (s, 1H, CH), 7.79–7.77 (m, 2H, Ar–CH), 7.39–7.35 (m, 2H, Ar–CH), 7.33–7.28 (m, 9H, Ar–CH), 7.27–7.25 (m, 2H, Ar–CH), 7.21 (s, 1H, CH), 3.90 (s, 3H, $CO_2$Me); $^{13}$C NMR (126 MHz, $CDCl_3$, 298 K) δ: 160.6 (C=O), 140.2, 133.9, 132.9, 128.9, 128.7, 128.4, 128.1, 127.7, 125.8, 108.5, 66.6 (CH), 52.2 ($CO_2$Me); IR $\nu_{max}$ (cm$^{-1}$): 3022, 2956, 1722 (C=O), 1643, 1544, 1515, 1447, 1340, 1225, 1262, 1171, 1089; HRMS (ES+) $[M+H]^+$ $[C_{24}H_{21}N_2O_2]^+$: calculated 369.1603, found 369.1609.

*Synthesis of methyl 1-(bis(4-fluorophenyl)methyl)-3-phenyl-1H-pyrazole-5-carboxylate* (**3e**)

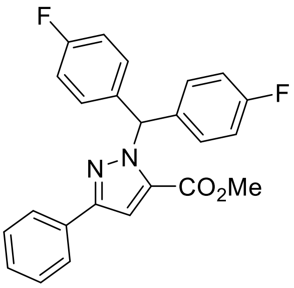


Synthesized in accordance with *General Procedure c* using $B(C_6F_5)_3$ (5 mg, 0.01 mmol), vinyl diazoester **1b** (20 mg, 0.10 mmol), and aryl ester **2b** (44 mg, 0.13 mmol) in 1,2-$C_2H_4Cl_2$ to afford **3e**. The crude reaction mixture was purified *via* preparative thin layer chromatography using hexane/ethyl acetate (95:5 v/v) as eluent. The desired compound **3e** was obtained as a white solid. Yield: 31 mg, 0.08 mmol, 77%.

$^1$H NMR (500 MHz, $CDCl_3$, 298 K) δ: 7.83 (s, 1H, CH), 7.77–7.75 (m, 2H, Ar–CH), 7.40–7.36 (m, 2H, Ar–CH), 7.33–7.31 (m, 1H, Ar–CH), 7.28–7.25 (m, 5H, Ar–CH), 7.21 (s, 1H, Ar–CH), 7.03–6.98 (m, 3H, Ar–CH), 3.91 (s, 3H, $CO_2$Me); $^{13}$C NMR (126 MHz, $CDCl_3$, 298 K) δ: 162.41 (d, $J_{C–F}$ = 246.7 Hz), 160.5 (C=O), 150.6, 135.9 (d, $J_{C–F}$ = 3.3 Hz), 133.8, 132.6, 130.5 (d, $J_{C–F}$ = 8.1 Hz), 129.2, 128.8, 128.3, 125.8, 115.4 (d, $J_{C–F}$ = 21.5 Hz), 108.6, 65.2 (CH), 52.3 ($CO_2$Me); $^{19}$F NMR (471 MHz, $CDCl_3$, 298 K) δ: -114.60 (s, 2F, Ar–F); IR $\nu_{max}$ (cm$^{-1}$): 3030, 2888, 1719 (C=O), 1604, 1509, 1445, 1379, 1258, 1224, 1159, 1089; HRMS (ES+) $[M+H]^+$ $[C_{24}H_{19}F_2N_2O_2]^+$: calculated 405.1415, found 405.1412.

*Synthesis of methyl 1-benzhydryl-3-(4-methoxyphenyl)-1H-pyrazole-5-carboxylate* (**3f**)

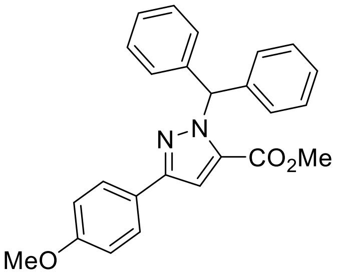


Synthesized in accordance with *General Procedure c* using $B(C_6F_5)_3$ (5 mg, 0.01 mmol), vinyl diazoester **1c** (23 mg, 0.10 mmol), and aryl ester **2a** (40 mg, 0.13 mmol) in 1,2-$C_2H_4Cl_2$ to afford **3f**. The crude reaction mixture was purified *via* preparative thin layer chromatography using hexane/ethyl acetate (95:5 v/v) as eluent. The desired compound **3f** was obtained as a white solid. Yield: 29 mg, 0.07 mmol, 73%.

$^1$H NMR (500 MHz, $CDCl_3$, 298 K) δ: 7.86 (s, 1H, CH), 7.72–7.69 (m, 2H, Ar–CH), 7.32–7.31 (m, 7H, Ar–CH), 7.29–7.27 (m, 2H, Ar–CH), 7.13 (s, 1H, CH), 6.91–6.89 (m, 2H, Ar–CH), 3.89 (s, 3H, OMe), 3.82 (s, 3H, $CO_2Me$); $^{13}$C NMR (126 MHz, $CDCl_3$, 298 K) δ: 160.6 (C=O), 159.7, 150.2, 140.3, 133.8, 128.9, 128.4, 127.7, 127.1, 125.7, 114.1, 107.9, 66.5 (CH), 55.5 (OMe), 52.1 ($CO_2Me$); IR $\nu_{max}$ (cm$^{-1}$): 3012, 2922, 1723 (C=O), 1645, 1565, 1523, 1432, 1411, 1354, 1312, 1262, 1223, 1175, 1089; HRMS (ES+) $[M+H]^+$ $[C_{25}H_{22}N_2O_3]^+$: calculated 398.1630, found 398.1633.

*Synthesis of methyl 1-(bis(4-fluorophenyl)methyl)-3-(4-methoxyphenyl)-1H-pyrazole-5-carboxylate* (**3g**)

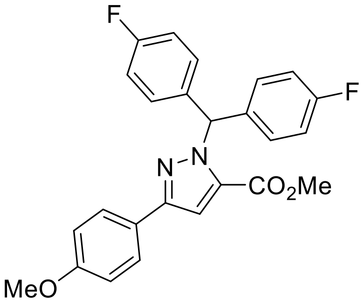


Synthesized in accordance with *General Procedure c* using $B(C_6F_5)_3$ (5 mg, 0.01 mmol), vinyl diazoester **1c** (23 mg, 0.10 mmol), and aryl ester **2b** (44 mg, 0.13 mmol) in 1,2-$C_2H_4Cl_2$ to afford **3g**. The crude reaction mixture was purified *via* preparative thin layer chromatography using hexane/ethyl acetate (95:5 v/v) as eluent. The desired compound **3g** was obtained as a white solid. Yield: 31 mg, 0.07 mmol, 72%.

$^1$H NMR (500 MHz, $CDCl_3$, 298 K) δ: 7.81 (s, 1H, Ar–CH), 7.70–7.67 (m, 2H, Ar–CH), 7.28–7.25 (m, 5H, Ar–CH), 7.13 (s, 1H, CH), 7.02–6.98 (m, 3H, Ar–CH), 6.93–6.90 (m, 2H, Ar–CH), 3.90 (s, 3H, OMe), 3.83 (s, 3H, $CO_2Me$); $^{13}$C NMR (126 MHz, $CDCl_3$, 298 K) δ: 162.4 (d, $J_{C–F}$ = 246.6 Hz), 160.6 (C=O), 159.8, 150.5, 136 (d, $J_{C–F}$ = 3.2 Hz), 133.7, 130.5 (d, $J_{C–F}$ = 8.2 Hz), 127.1, 125.5, 115.4, 115.3, 114.2, 108.0, 65.1 (CH), 55.5 (OMe), 52.2 ($CO_2Me$); $^{19}$F NMR (471 MHz, $CDCl_3$, 298 K) δ: -114.69 (s, 2F, Ar–F); IR $\nu_{max}$ (cm$^{-1}$): 3015, 2856, 1717 (C=O), 1634, 1509, 1449, 1365, 1320, 1222, 1254, 1174, 1087; HRMS (ES+) $[M+H]^+$ $[C_{25}H_{21}F_2N_2O_3]^+$: calculated 435.1520, found 435.1513.

*Synthesis of methyl 1-(1-(naphthalen-2-yl)ethyl)-3-phenyl-1H-pyrazole-5-carboxylate* (**3h**)

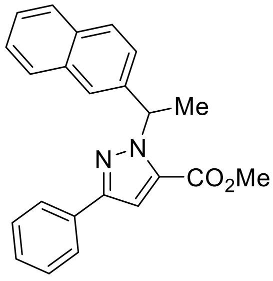


Synthesized in accordance with *General Procedure c* using $B(C_6F_5)_3$ (5 mg, 0.01 mmol), vinyl diazoester **1b** (20 mg, 0.10 mmol), and aryl ester **2e** (38 mg, 0.13 mmol) in 1,2-$C_2H_4Cl_2$ to afford **3h**. The crude reaction mixture was purified *via* preparative thin layer chromatography using hexane/ethyl acetate (95:5 v/v) as eluent. The desired compound **3h** was obtained as a white solid. Yield: 27 mg, 0.07 mmol, 75%.

$^1$H NMR (500 MHz, $CDCl_3$, 298 K) δ: 7.91–7.88 (m, 2H, Ar–CH), 7.81–7.77 (m, 3H, Ar–CH), 7.75 (s, 1H, Ar–CH), 7.55 (dd, $J$ = 8.5, 1.8 Hz, 1H, Ar–CH), 7.45–7.42 (m, 4H, Ar–CH), 7.36–7.33 (m, 1H, Ar–CH), 7.18 (s, 1H, CH), 6.75 (q, $J$ = 7.0 Hz, 1H, CH), 3.85 (s, 3H, $CO_2Me$), 2.06 (d, $J$ = 7.1 Hz, 3H, Me); $^{13}$C NMR (126 MHz, $CDCl_3$, 298 K) δ: 160.4 (C=O), 149.9, 140.2, 133.4, 133.2, 133.0, 132.9, 128.8, 128.4, 128.2, 128.1, 127.7, 126.2, 126.0, 125.8, 125.5, 124.9, 108.8, 59.5 (CH), 52.0 ($CO_2Me$), 22.2 (Me); IR $\nu_{max}$ ($cm^{-1}$): 3031, 2921, 1721 (C=O), 1677, 1535, 1477, 1392, 1320, 1231, 1254, 1171, 1089; HRMS (ES+) $[M+H]^+$ $[C_{23}H_{21}N_2O_2]^+$: calculated 357.1603, found 357.1595.

*Synthesis of methyl 3-(4-fluorophenyl)-1-(1-(naphthalen-2-yl)ethyl)-1H-pyrazole-5-carboxylate* (**3i**)

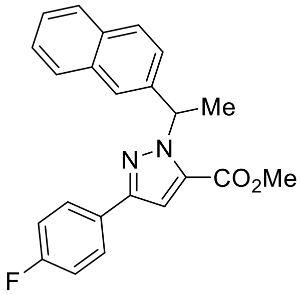


Synthesized in accordance with *General Procedure c* using $B(C_6F_5)_3$ (5 mg, 0.01 mmol), vinyl diazoester **1a** (22 mg, 0.10 mmol), and aryl ester **2e** (38 mg, 0.13 mmol) in 1,2-$C_2H_4Cl_2$ to afford **3i**. The crude reaction mixture was purified *via* preparative thin layer chromatography using hexane/ethyl acetate (95:5 v/v) as eluent. The desired compound **3i** was obtained as a white solid. Yield: 29 mg, 0.08 mmol, 78%.

$^1$H NMR (500 MHz, $CDCl_3$, 298 K) δ: 7.86–7.83 (m, 2H, Ar–CH), 7.81–7.77 (m, 3H, Ar–CH), 7.74 (s, 1H, CH), 7.54 (dd, $J$ = 8.6, 1.8 Hz, 1H, Ar–CH), 7.46–7.42 (m, 2H, Ar–CH), 7.14–7.09 (m, 3H, Ar–CH), 6.74 (q, $J$ = 7.0 Hz, 1H, CH), 3.85 (s, 3H, $CO_2Me$), 2.05 (d, $J$ = 7.1 Hz, 3H, Me); $^{13}$C NMR (126 MHz, $CDCl_3$, 298 K) δ: 162.8 (d, $J_{C–F}$ = 246.8 Hz), 160.3 (C=O), 149.1, 140.1, 133.3 (d, $J_{C–F}$ = 10.7 Hz), 132.9, 129.3 (d, $J_{C–F}$ = 3.2 Hz), 128.4, 128.2, 127.7, 127.5 (d, $J_{C–F}$ = 8.1 Hz), 126.2, 126.1, 125.5, 124.9, 115.7 (d, $J_{C–F}$ = 21.6 Hz), 108.5, 59.5 (CH), 52.1 ($CO_2Me$), 22.1 (Me); $^{19}$F NMR (471 MHz, $CDCl_3$, 298 K) δ: -114.18 (s, 1F, Ar–F); IR $\nu_{max}$ ($cm^{-1}$): 3033, 2902, 1719 (C=O), 1633, 1504, 1455, 1411, 1303, 1251, 1232, 1171, 1081; HRMS (ES+) $[M+H]^+$ $[C_{23}H_{20}FN_2O_2]^+$: calculated 375.1509, found 375.1508.

*Synthesis of methyl 3-(4-methoxyphenyl)-1-(1-(naphthalen-2-yl)ethyl)-1H-pyrazole-5-carboxylate* (**3j**)

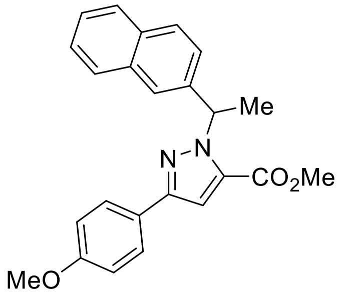


Synthesized in accordance with *General Procedure c* using $B(C_6F_5)_3$ (5 mg, 0.01 mmol), vinyl diazoester **1c** (23 mg, 0.10 mmol), and aryl ester **2e** (38 mg, 0.13 mmol) in 1,2-$C_2H_4Cl_2$ to afford **3j**. The crude reaction mixture was purified *via* preparative thin layer chromatography using hexane/ethyl acetate (95:5 v/v) as eluent. The desired compound **3j** was obtained as a white solid. Yield: 27 mg, 0.07 mmol, 70%.

$^1$H NMR (500 MHz, $CDCl_3$, 298 K) δ: 7.83–7.76 (m, 5H, Ar–CH), 7.74 (s, 1H, Ar–CH), 7.54 (dd, $J$ = 8.6, 1.8 Hz, 1H, Ar–CH), 7.44–7.42 (m, 2H, Ar–CH), 7.09 (s, 1H, CH), 6.96 (d, $J$ = 8.7 Hz, 2H, Ar–CH), 6.73 (q, $J$ = 7.0 Hz, 1H, CH), 3.85 (s, 3H, OMe), 3.84 (s, 3H, $CO_2Me$), 2.05 (d, $J$ = 7.0 Hz, 3H, Me); $^{13}$C NMR (126 MHz, $CDCl_3$, 298 K) δ: 160.4 (C=O), 159.7, 149.8, 140.3, 133.4, 133.1, 132.9, 128.3, 128.2, 127.7, 127.1, 126.1, 126.0, 125.9, 125.4, 124.9, 114.2, 108.2, 59.4 (CH), 55.5 (OMe), 52.0 ($CO_2Me$), 22.2 (Me);.IR $\nu_{max}$ ($cm^{-1}$): 3021, 2932, 1722 (C=O), 1654, 1532, 1501, 1475, 1421, 1322, 1245, 1221, 1171, 1087; HRMS (ES+) $[M+H]^+$ $[C_{24}H_{23}N_2O_3]^+$: calculated 387.1709, found 387.1707.

*Control experiment between experiment between* ***2a*** *and* ***3e*** *using 10%* $B(C_6F_5)_3$.

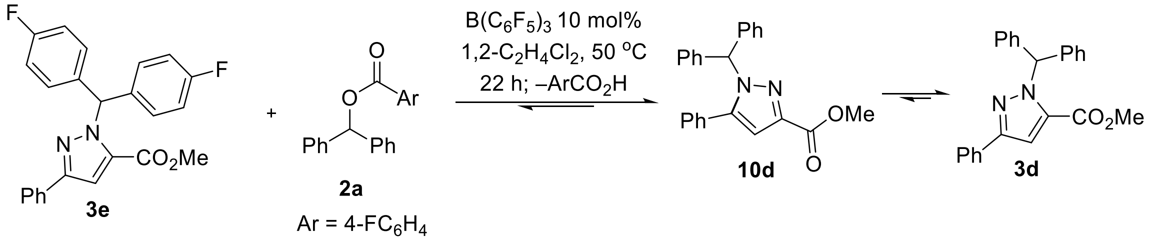


In a stirred 1,2-$C_2H_4Cl_2$ solution (0.5 mL) of **2a** (41mg, 0.1 mmol), 1,2-$C_2H_4Cl_2$ solution (0.5 mL) of $B(C_6F_5)_3$ (5 mg, 0.01 mmol, 10 mol%) was added dropwise. Another separate 1,2-$C_2H_4Cl_2$ solution (0.5 mL) of compound **3e** (37 mg, 0.12 mmol) was added slowly into the reaction mixture. The reaction tube was sealed under nitrogen atmosphere and heated at 50 $^o$C for 22 h. All volatiles were removed *in vacuo.* A mixture of compounds were obtained and purified *via* preparative thin layer chromatography using hexane/ethyl acetate (95:5 v/v) as eluent. Compound **3d** was isolated as white solid (18 mg, 0.049 mmol, 49%) and compound **10d** was isolated as a minor product (6 mg, 0.016 mmol, 16%). **3e** was isolated as unreacted starting material (7 mg, 0.017 mmol, 17%).

*Characterization of methyl 1-benzhydryl-5-phenyl-1H-pyrazole-3-carboxylate* (**10d**)

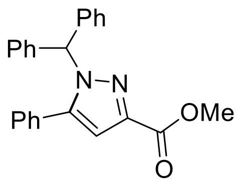


$^{1}H$ NMR (600 MHz, $CDCl_3$, 298 K) δ: 7.47–7.41 (m, 3H, Ar–CH), 7.36–7.27 (m, 9H, Ar–CH), 7.20–7.15 (m, 4H, Ar–CH), 6.88 (s, 1H, CH), 3.89 (s, 3H, $CO_2Me$); $^{13}C$ NMR (151 MHz, $CDCl_3$, 298 K) δ: 163.2 (C=O), 146.3, 143.5, 139.6, 130.7, 130.0, 129.5, 129.4, 129.4, 129.0, 128.6, 128.0, 109.2, 66.3 (CH), 52.1($CO_2Me$); IR $\nu_{max}$ ($cm^{-1}$): 3011, 2917, 1715 (C=O), 1622, 1577, 1487, 1433, 1365, 1242, 1215, 1168, 1087; HRMS (ES+) $[M+H]^+$ $[C_{24}H_{20}N_2O_2]^+$: calculated 368.1525, found 368.1527.

*Reaction between stoichiometric* ***1b*** *with* $B(C_6F_5)_3$

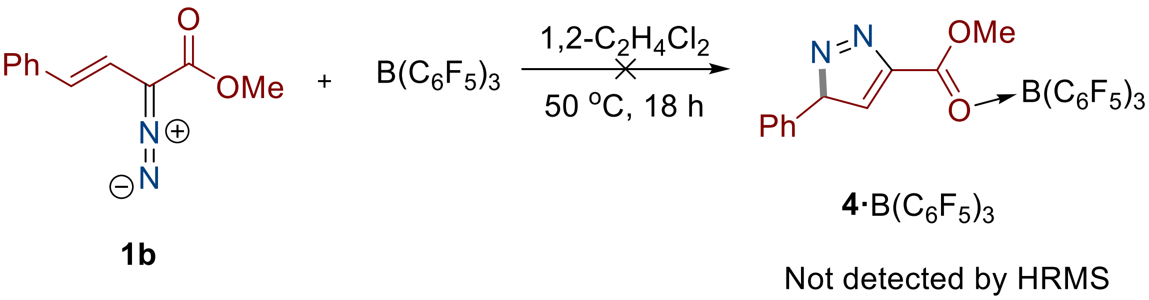


**1b**

**4**·$B(C_6F_5)_3$

Not detected by HRMS

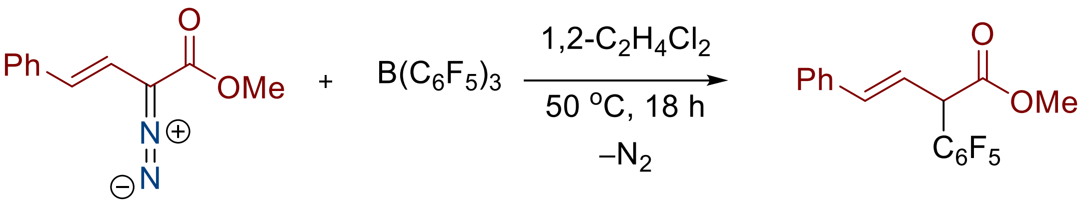


**1b**

## 3. NMR Spectra

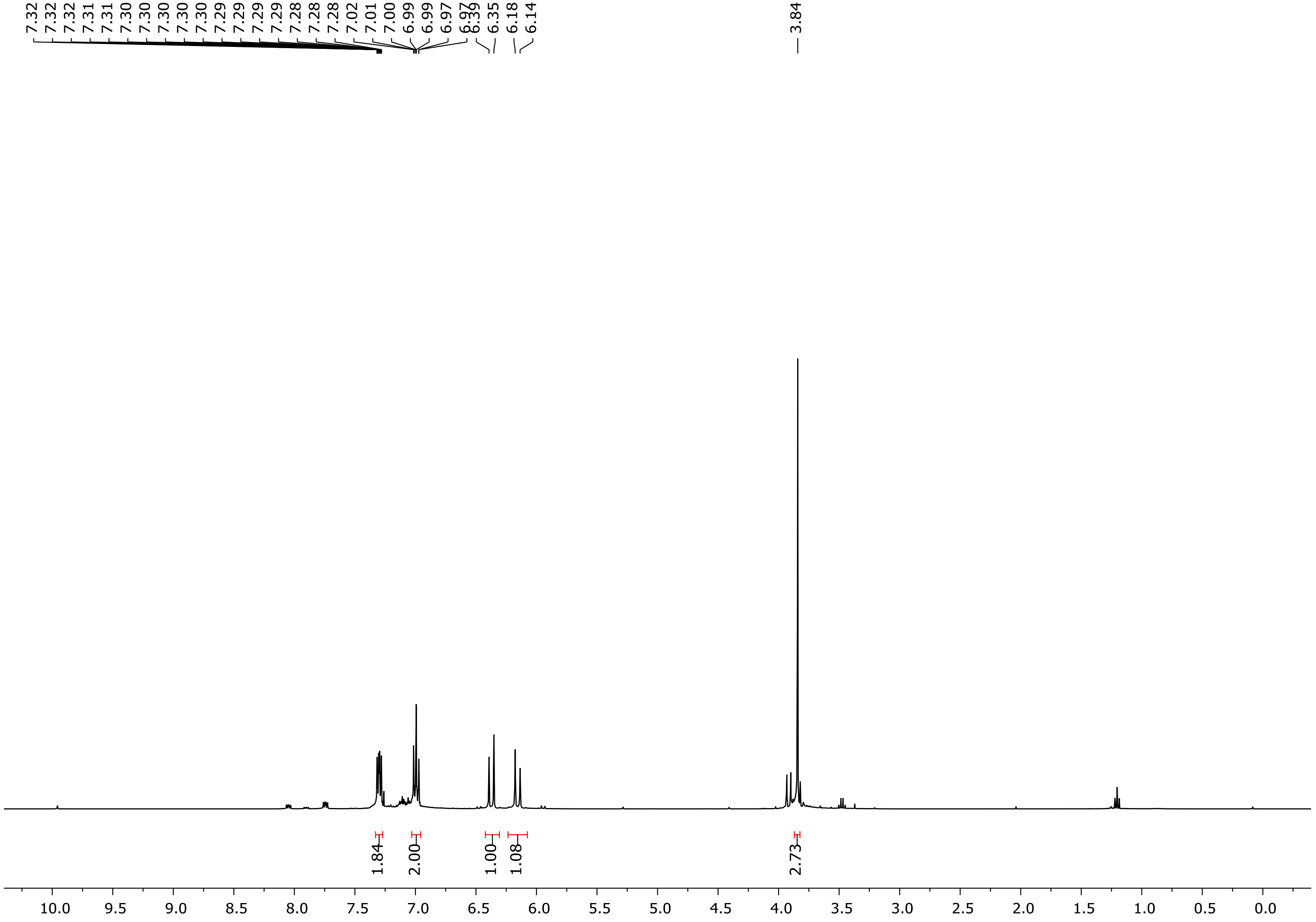


Figure S1: **$^1$H NMR** (400 MHz, $CDCl_3$, 298 K) spectrum of **1a**.

Figure S2: **$^{13}$C NMR** (101 MHz, $CDCl_3$, 298 K) spectrum of **1a**.

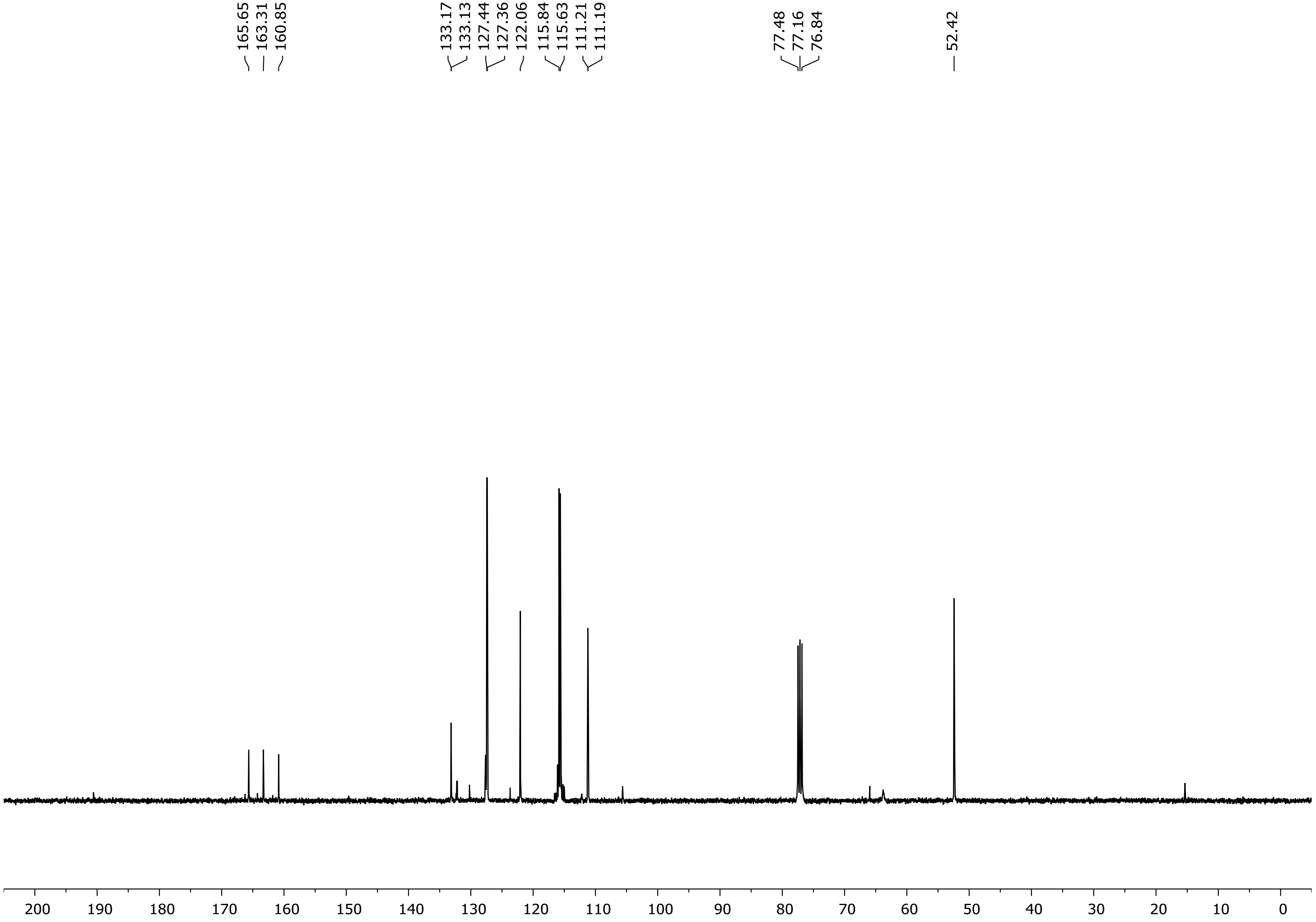

Figure S3: **$^{19}$F NMR** (376 MHz, $CDCl_3$, 298 K) spectrum of **1a**.

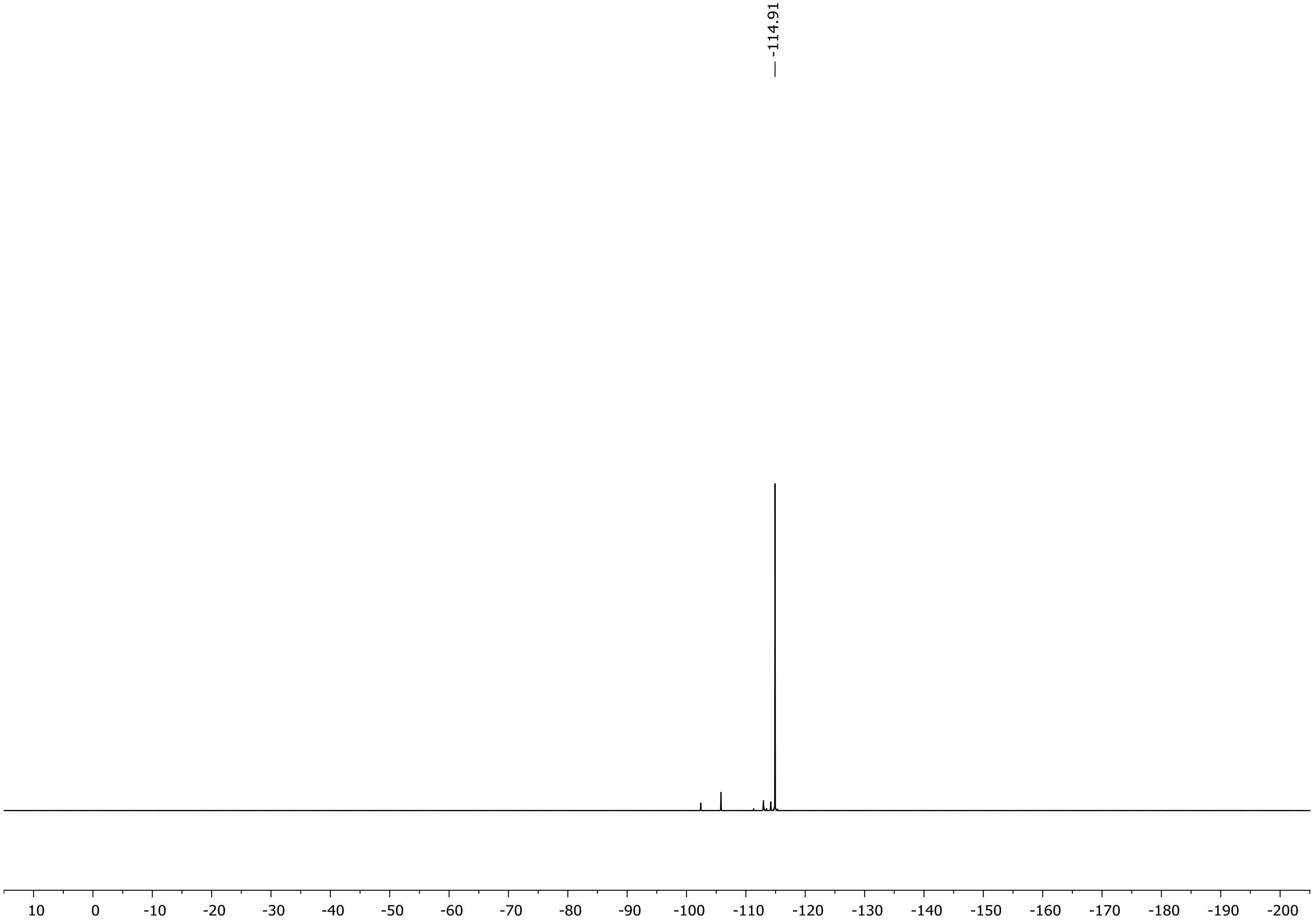

Figure S4: **$^{1}$H NMR** (400 MHz, $CDCl_3$, 298 K) spectrum of **1b**.

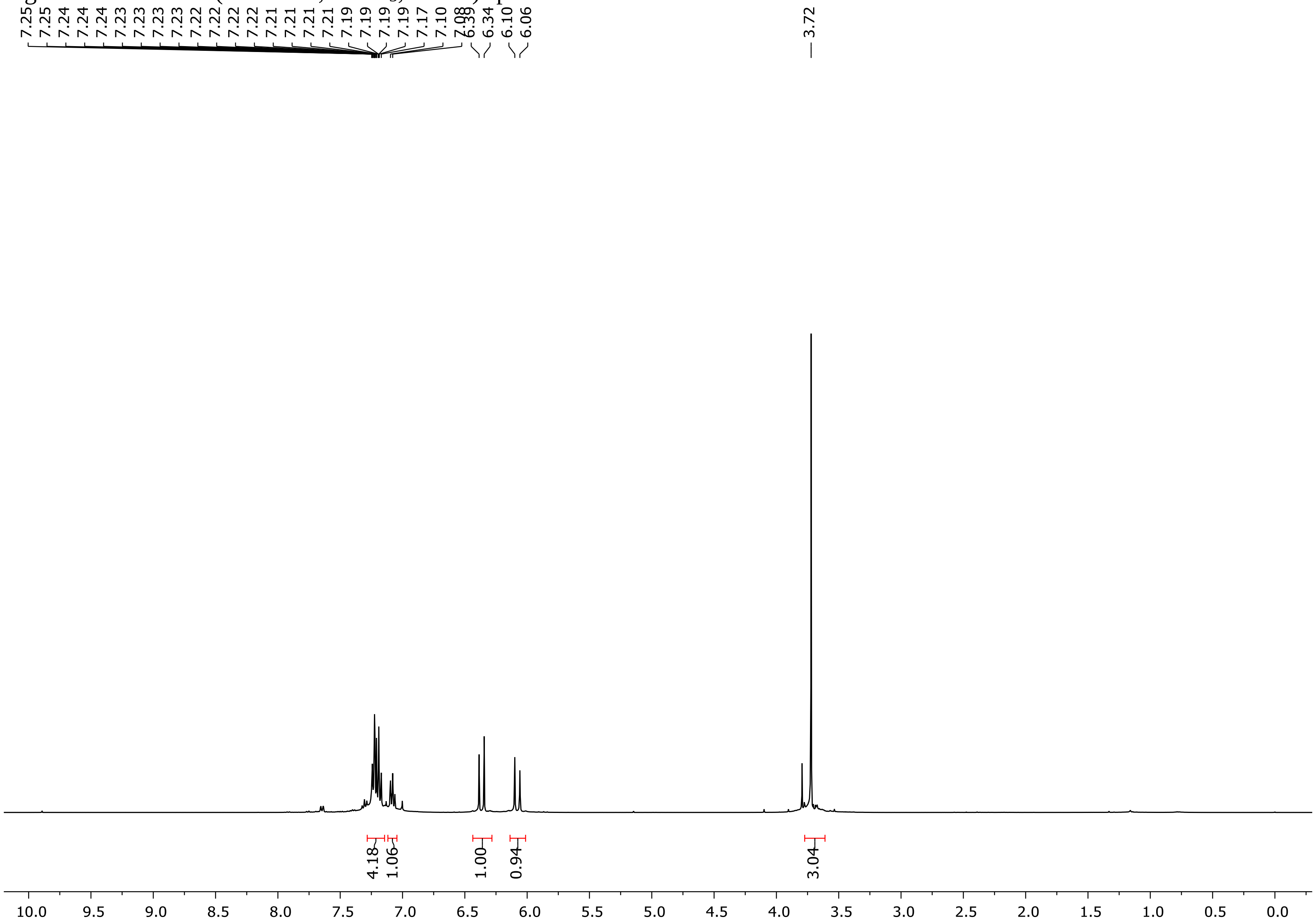

Figure S5: **$^{13}$C NMR** (101 MHz, $CDCl_3$, 298 K) spectrum of **1b**.

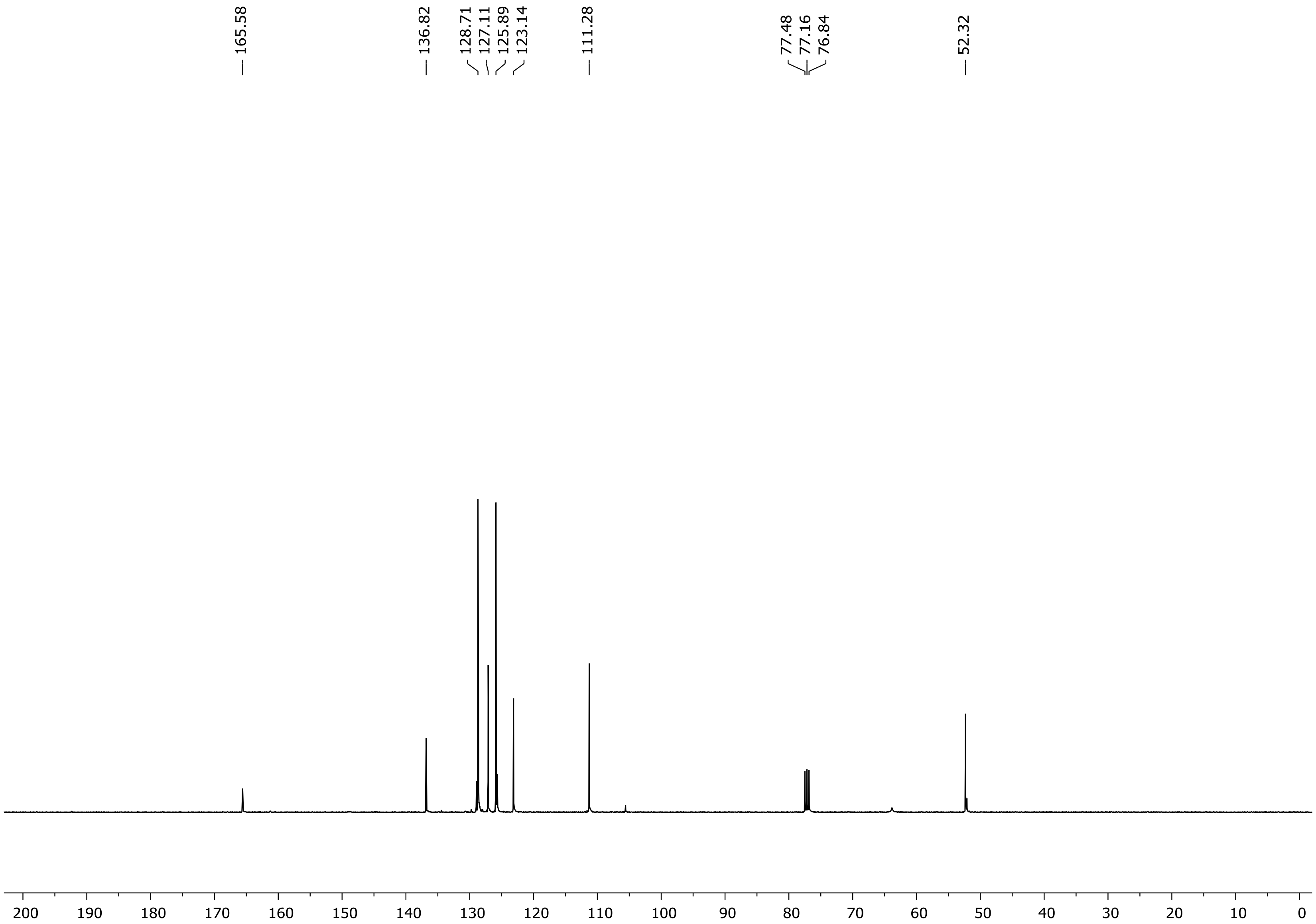

Figure S6: **$^{1}$H NMR** (400 MHz, $CDCl_3$, 298 K) spectrum of **1c**.

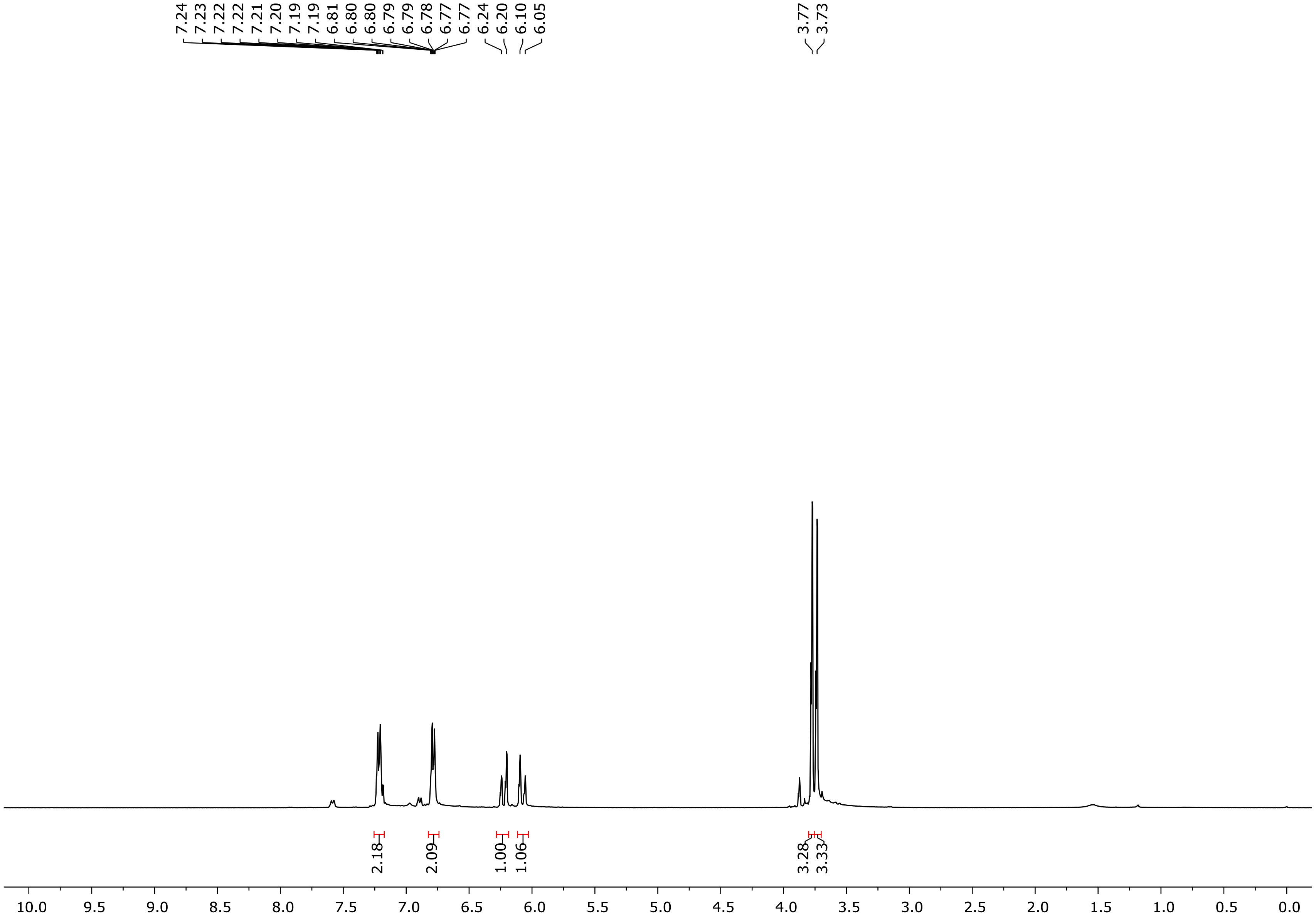

Figure S7: **$^{13}$C NMR** (101 MHz, $CDCl_3$, 298 K) spectrum of **1c**.

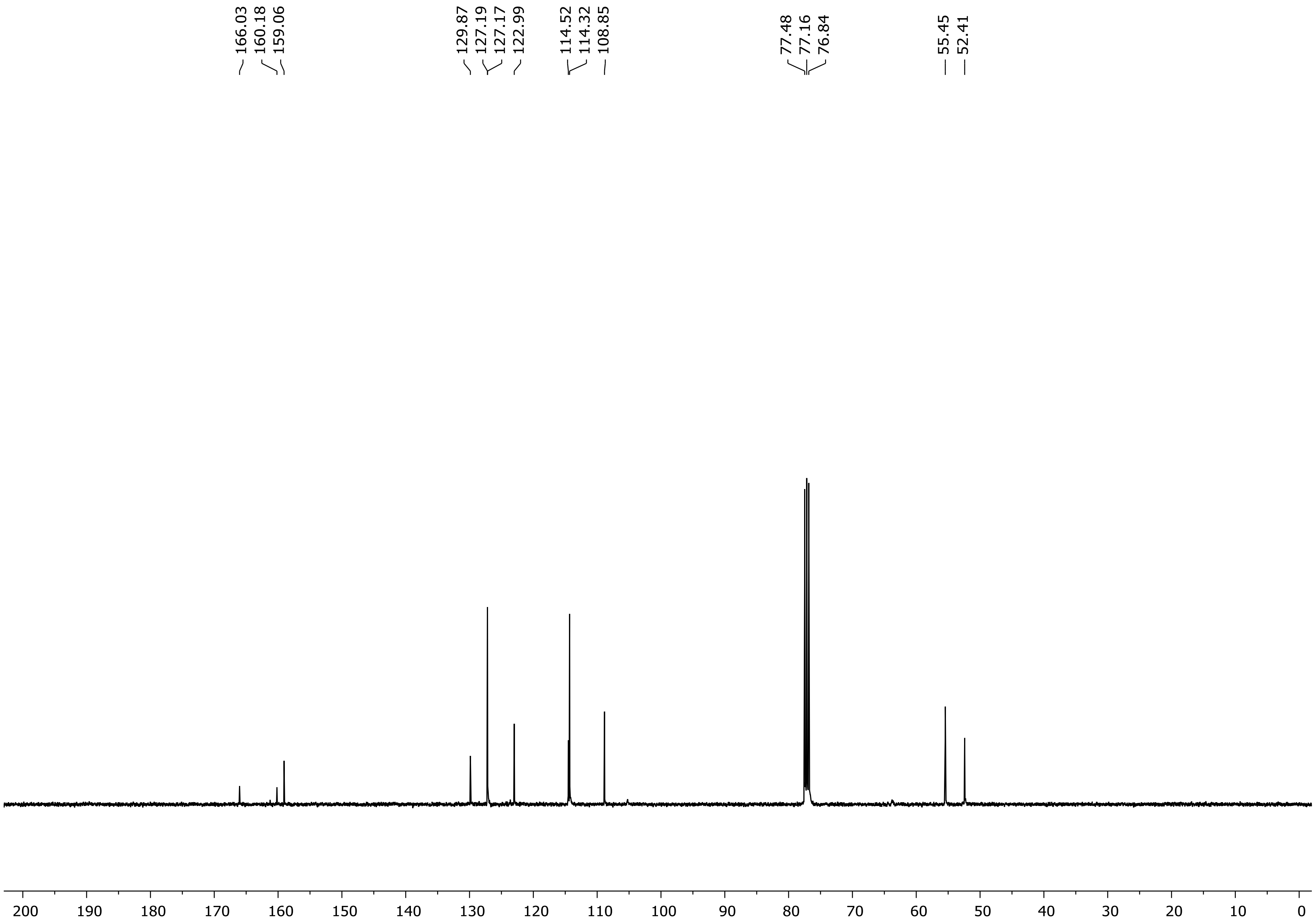

Figure S8: **$^{1}$H NMR** (500 MHz, $CDCl_3$, 298 K) spectrum of **2a**.

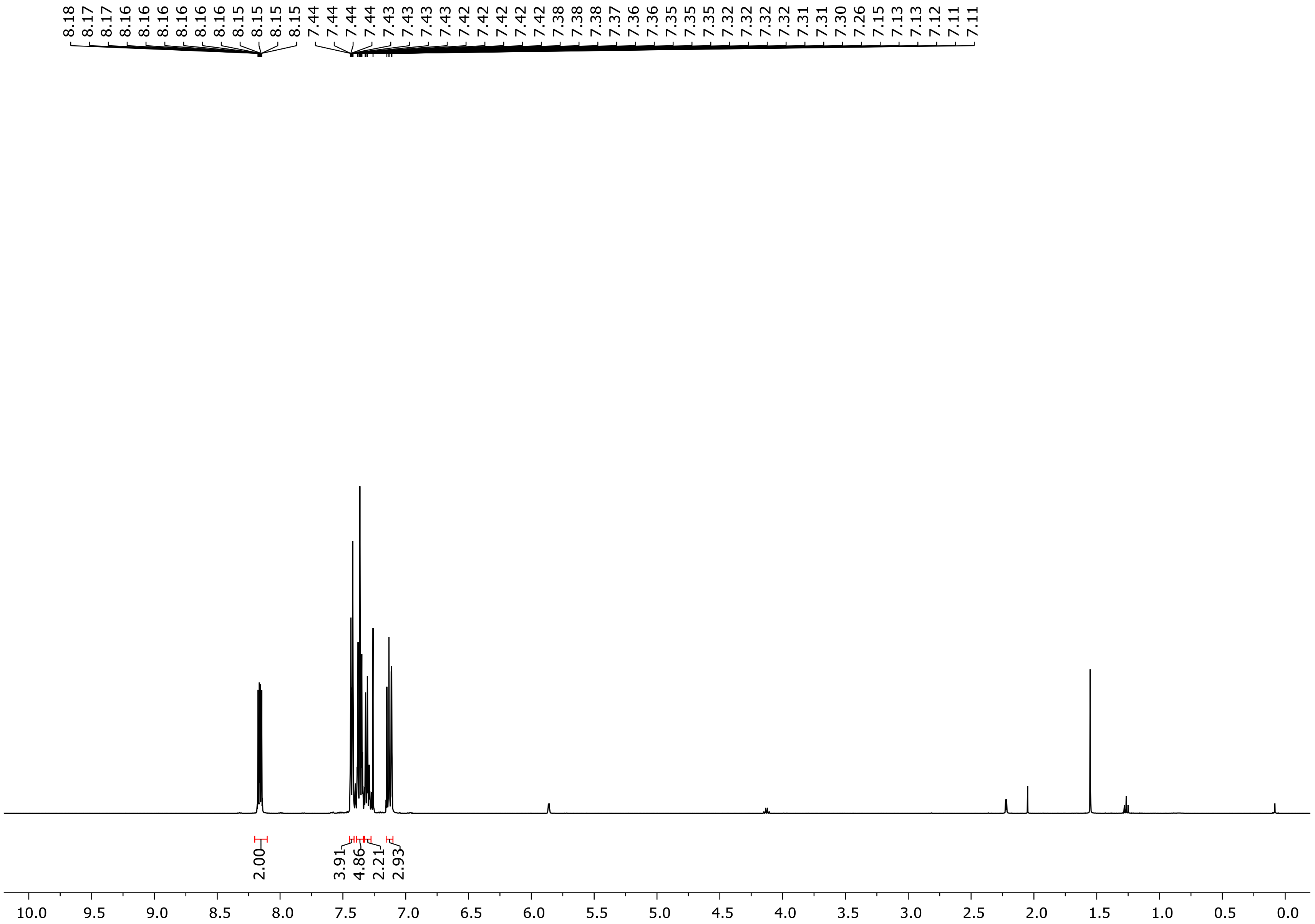

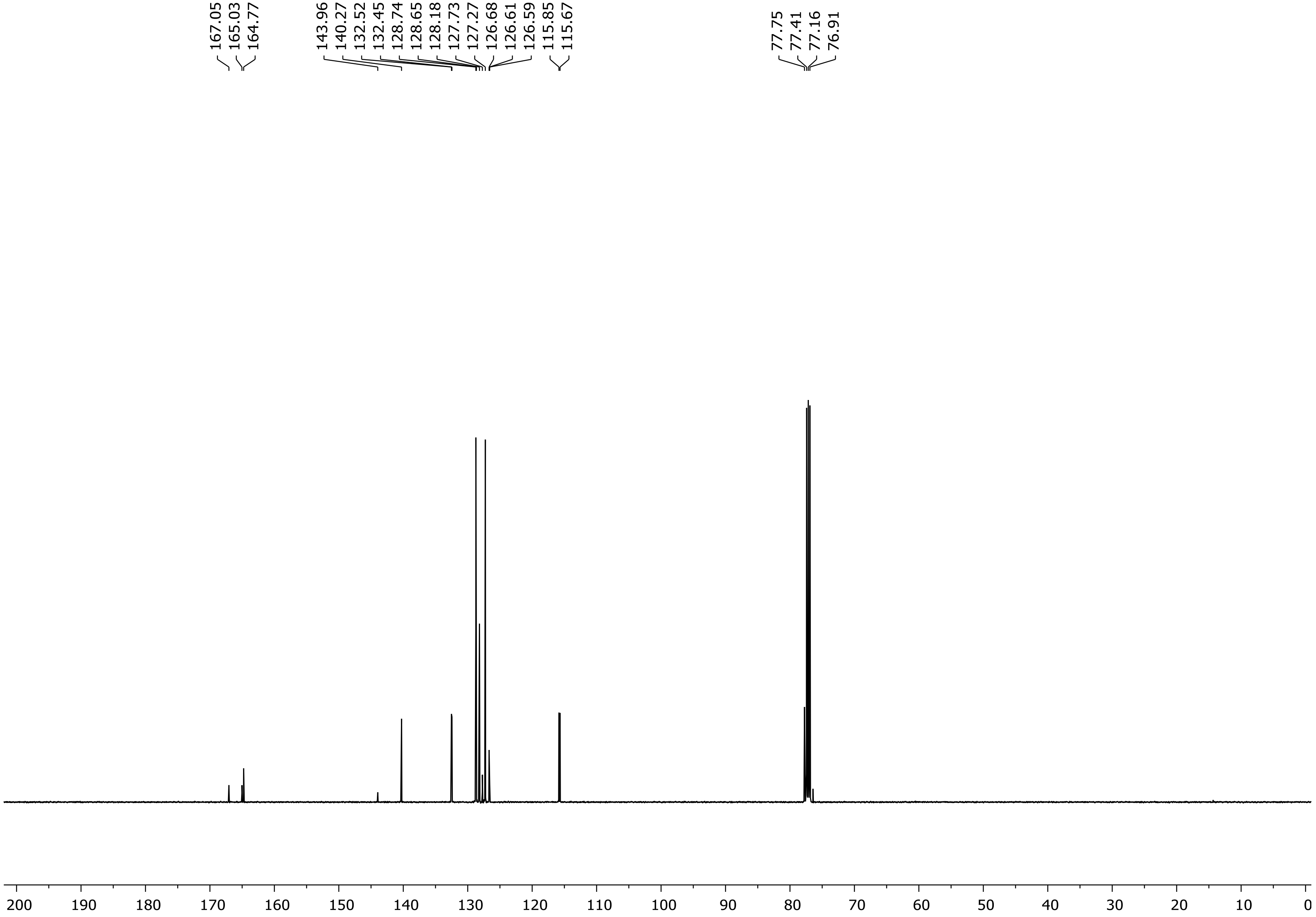


Figure S9: **$^{13}$C NMR** (126 MHz, $CDCl_3$, 298 K) spectrum of **2a**.

Figure S10: $^{19}$**F NMR** (471 MHz, $CDCl_3$, 298 K) spectrum of **2a**.

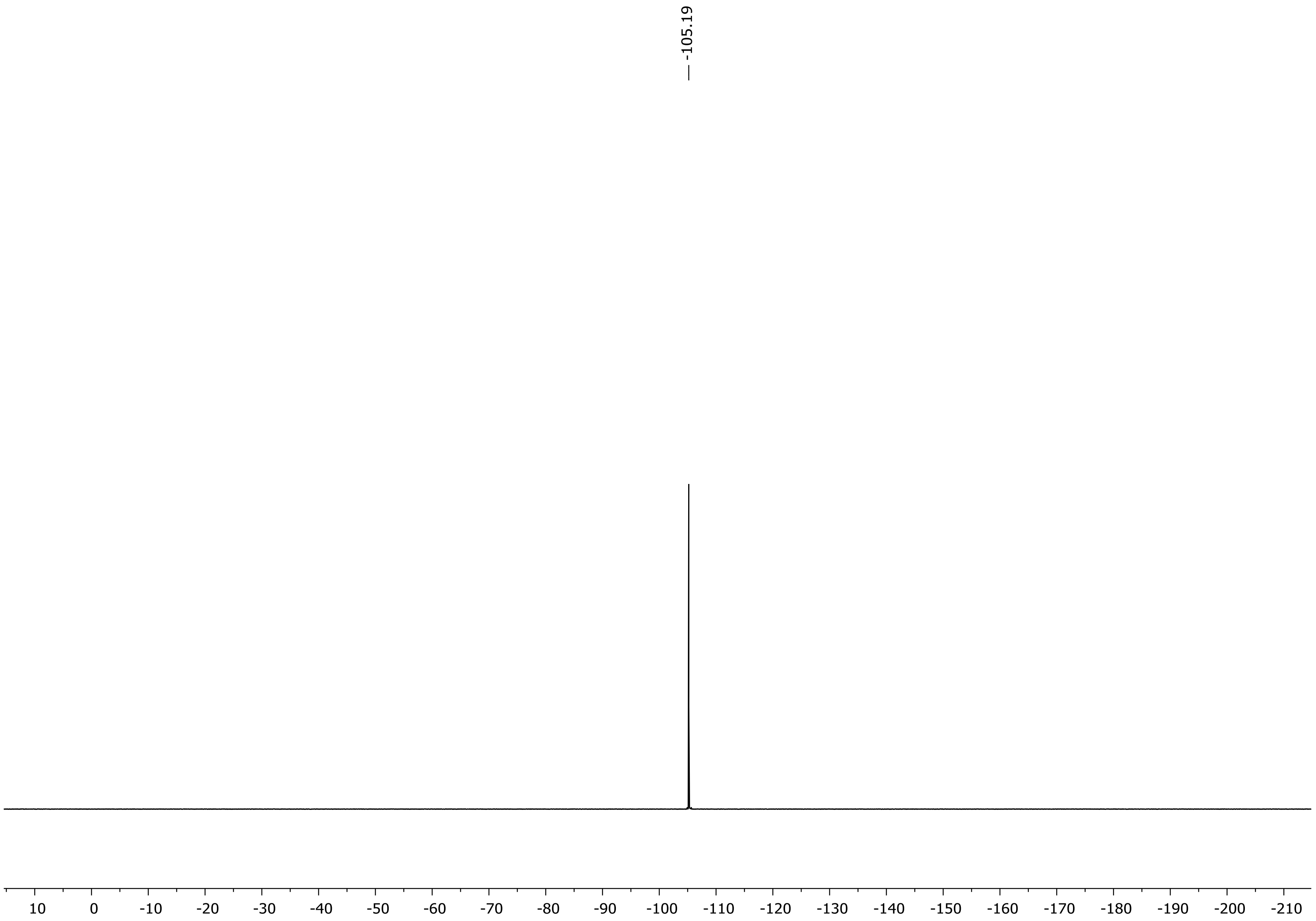

Figure S11: **$^{1}$H NMR** (400 MHz, $CDCl_3$, 298 K) spectrum of **2b**.

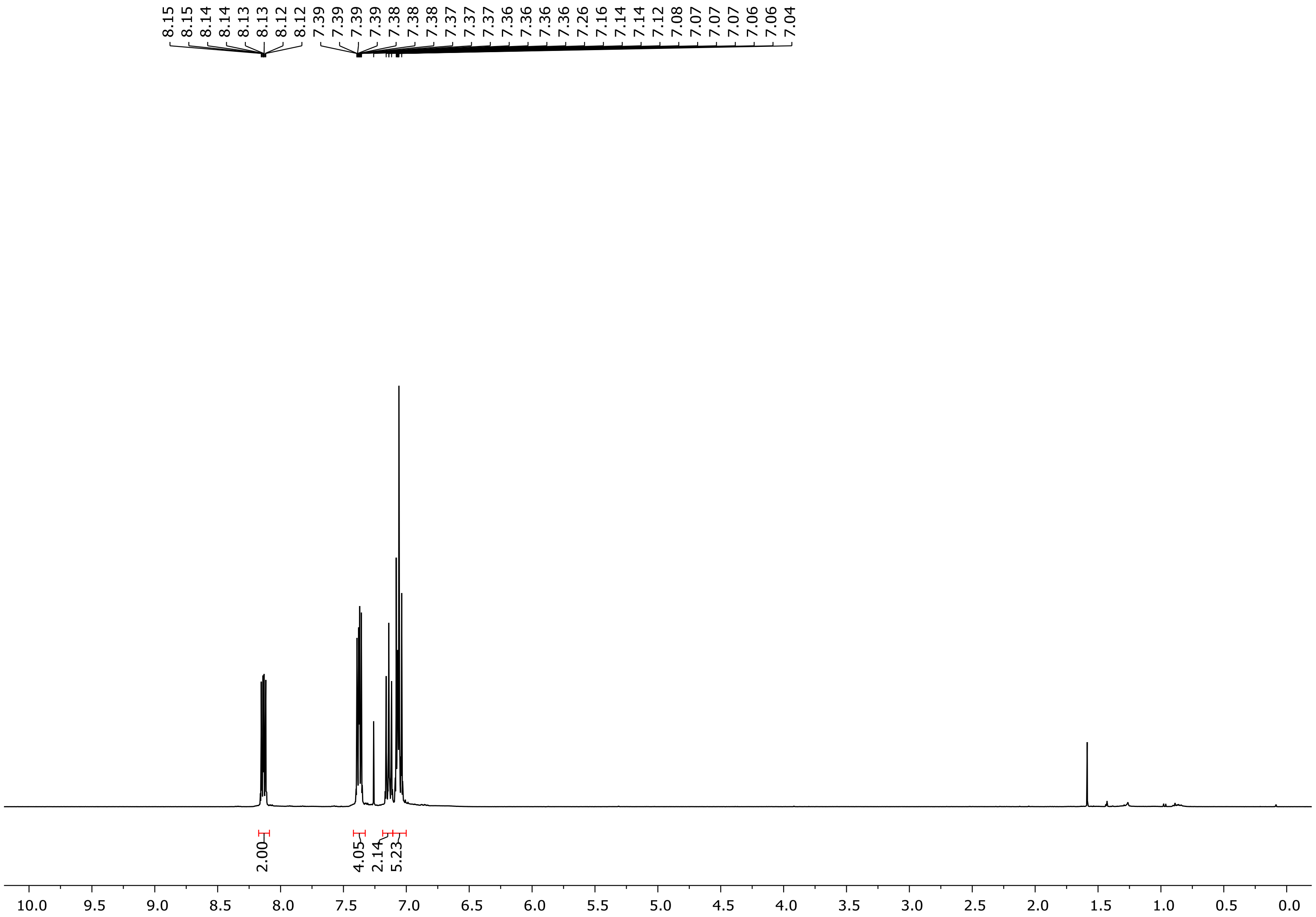

Figure S12: **$^{13}$C NMR** (101 MHz, $CDCl_3$, 298 K) spectrum of **2b**.

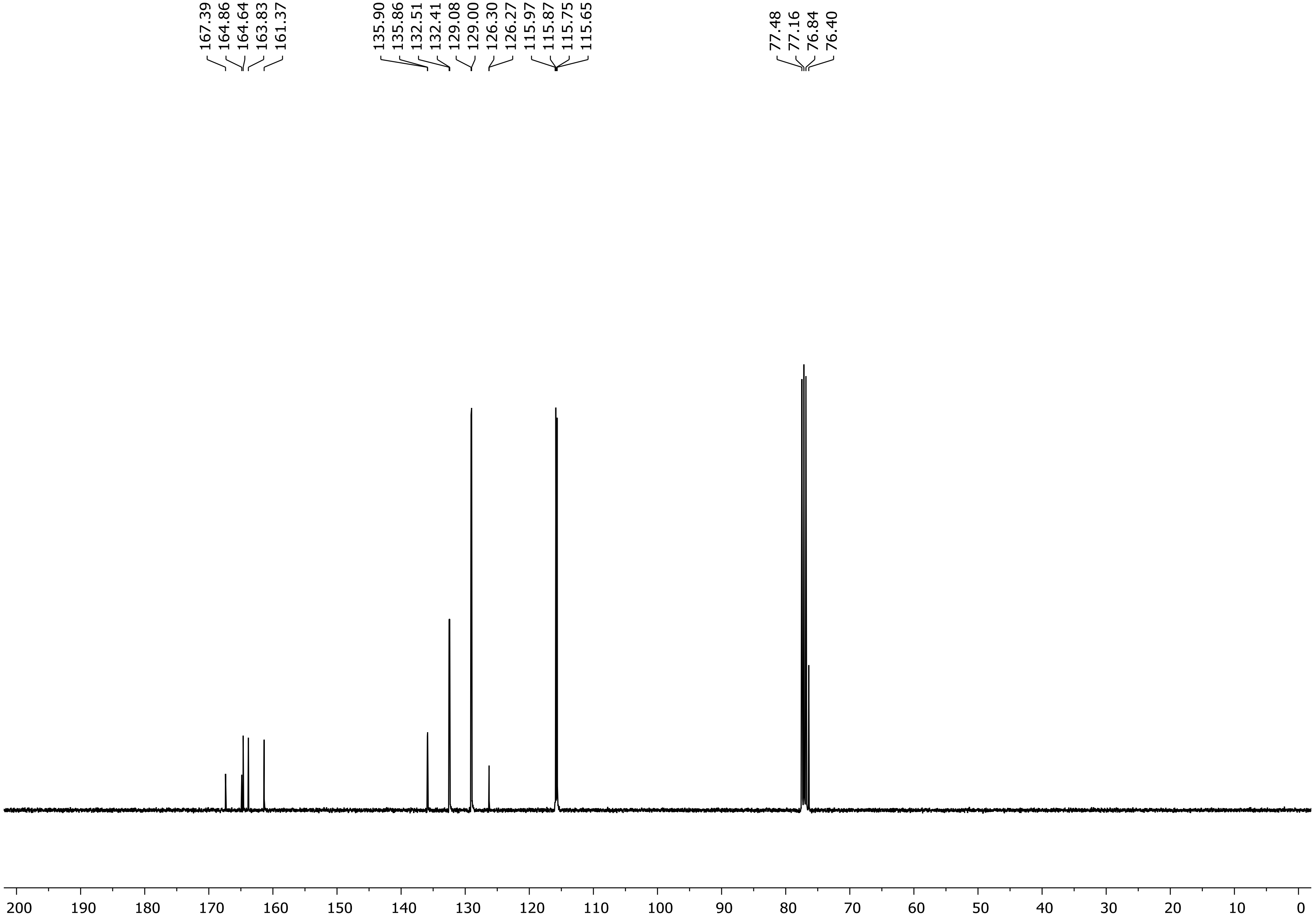

Figure S13: $^{19}$**F NMR** (376 MHz, $CDCl_3$, 298 K) spectrum of **2b**.

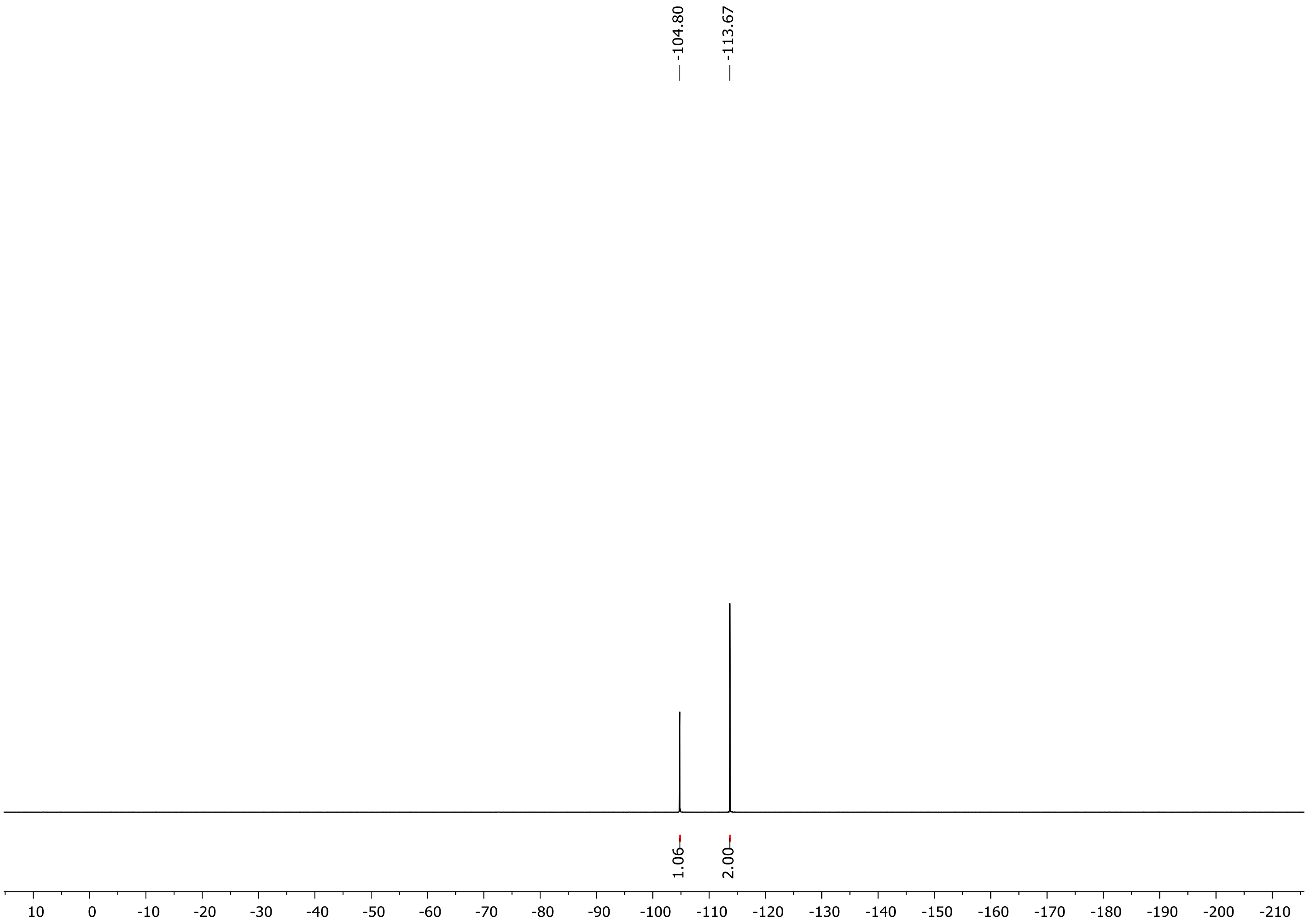

Figure S14: **$^1$H NMR** (500 MHz, $CDCl_3$, 298 K) spectrum of **2c**.

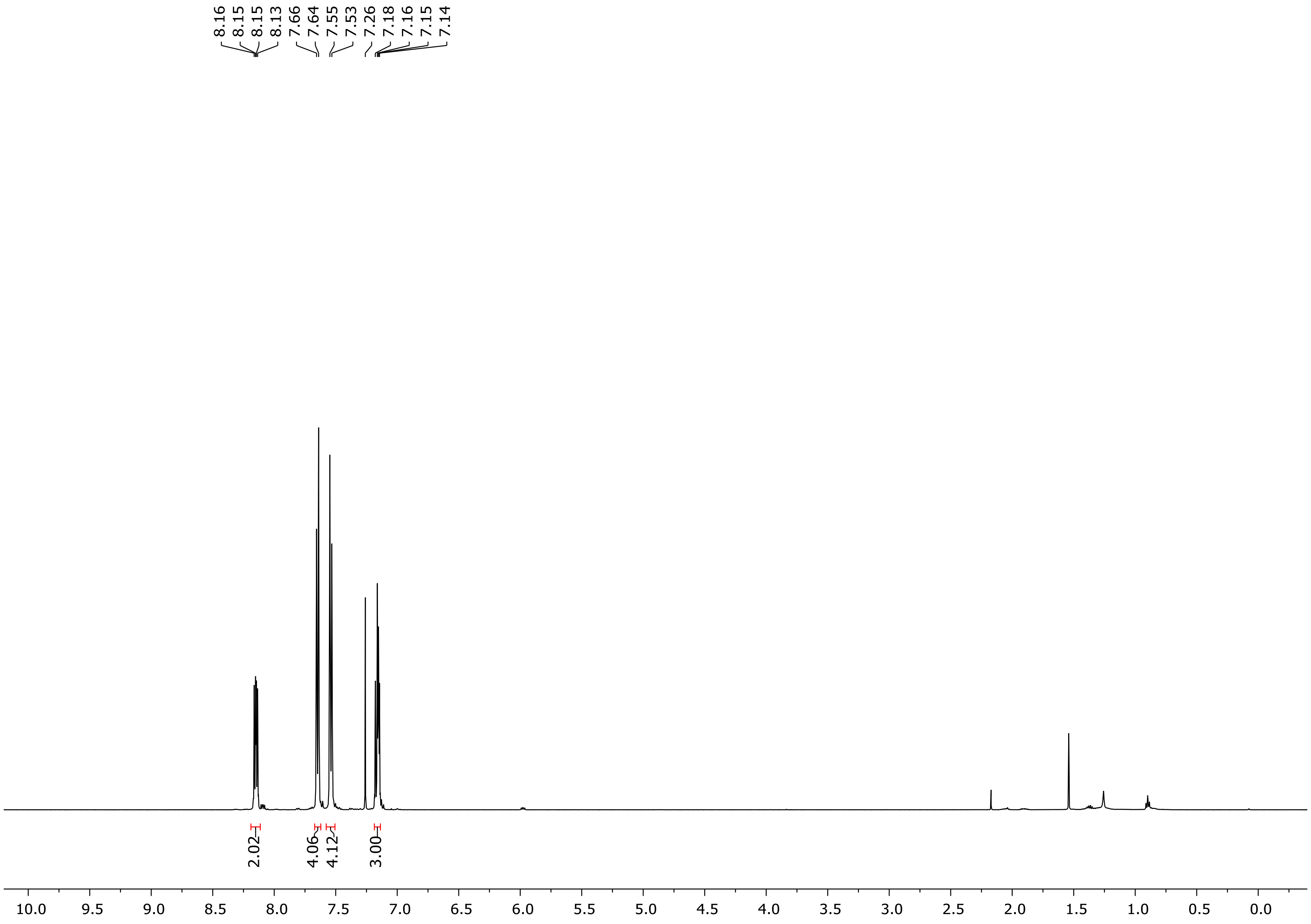

Figure S15: **$^{13}$C NMR** (126 MHz, $CDCl_3$, 298 K) spectrum of **2c**.

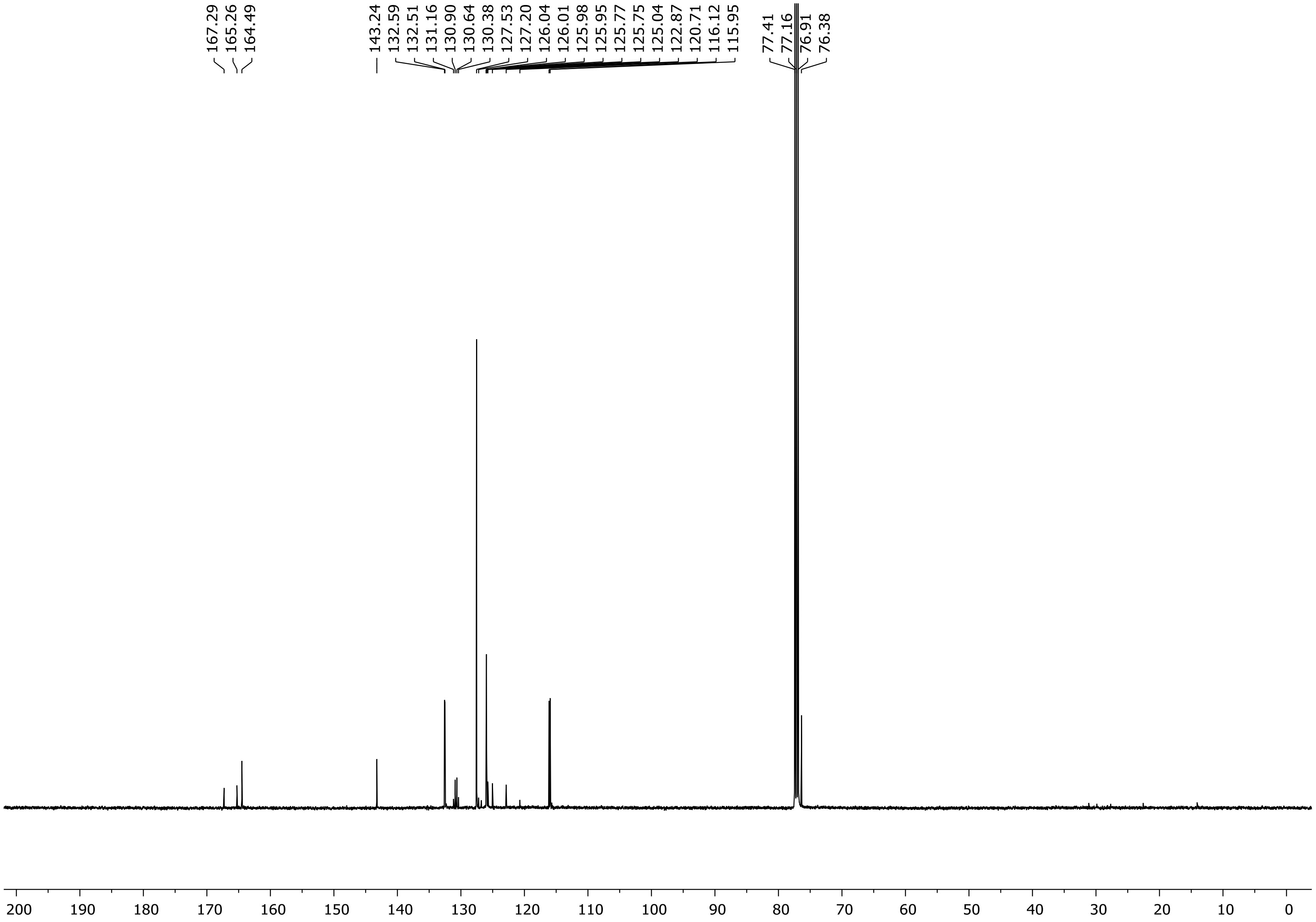

Figure S16: **$^{19}$F NMR** (471 MHz, $CDCl_3$, 298 K) spectrum of **2c**.

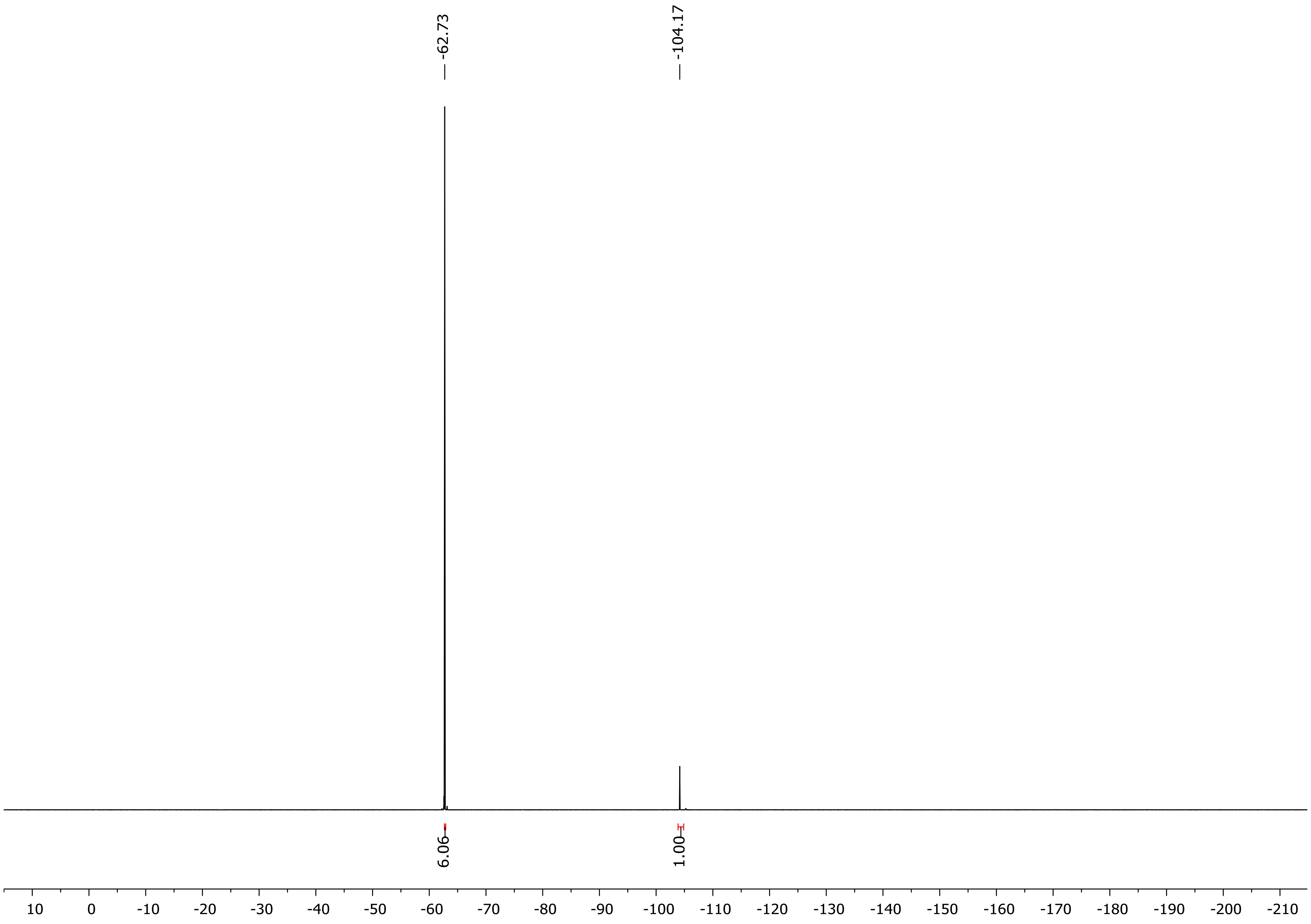

Figure S17: [1]**H NMR** (500 MHz, $CDCl_3$, 298 K) spectrum of **2d**.

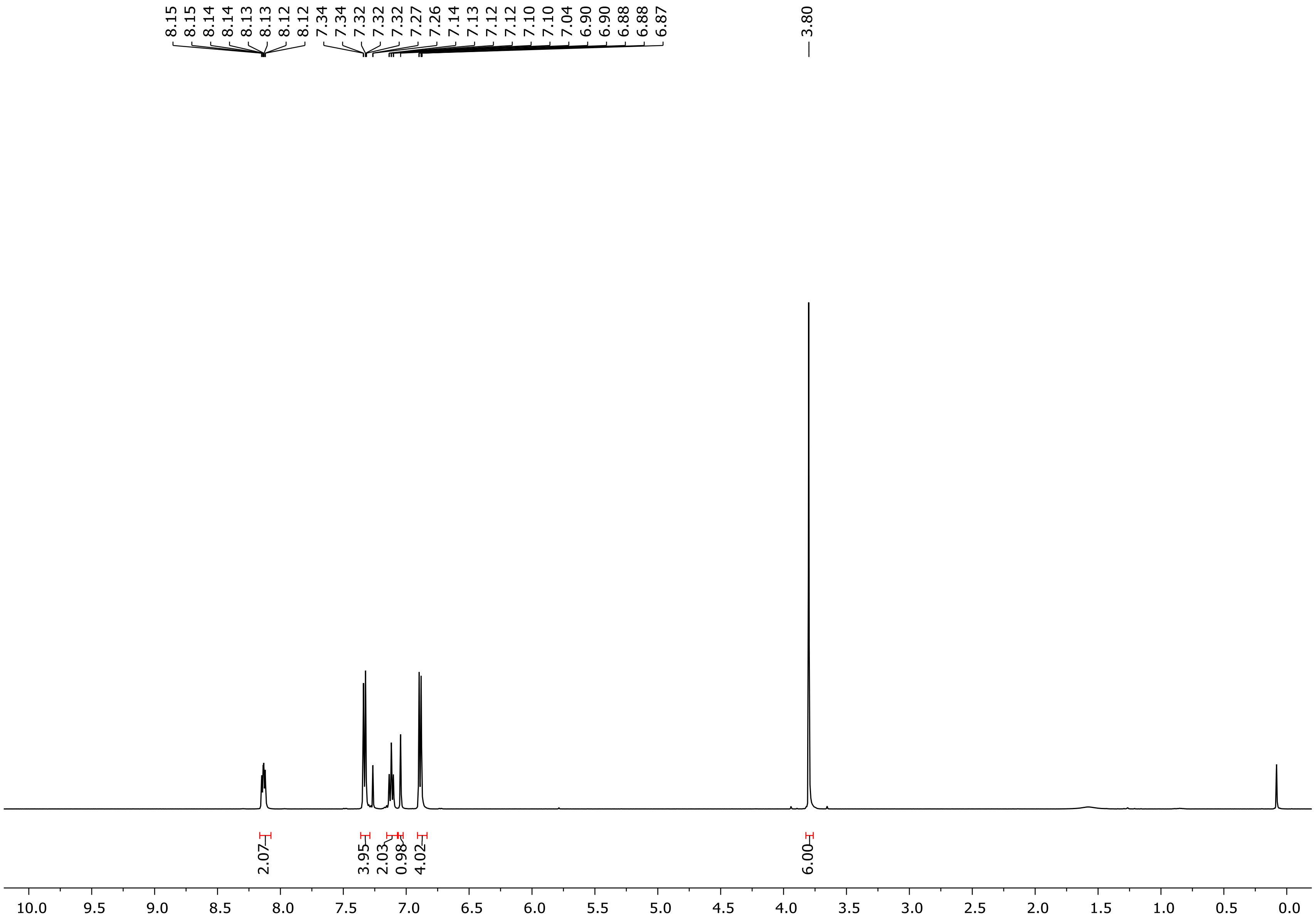

Figure S18: **$^{13}$C NMR** (126 MHz, $CDCl_3$, 298 K) spectrum of **2d**.

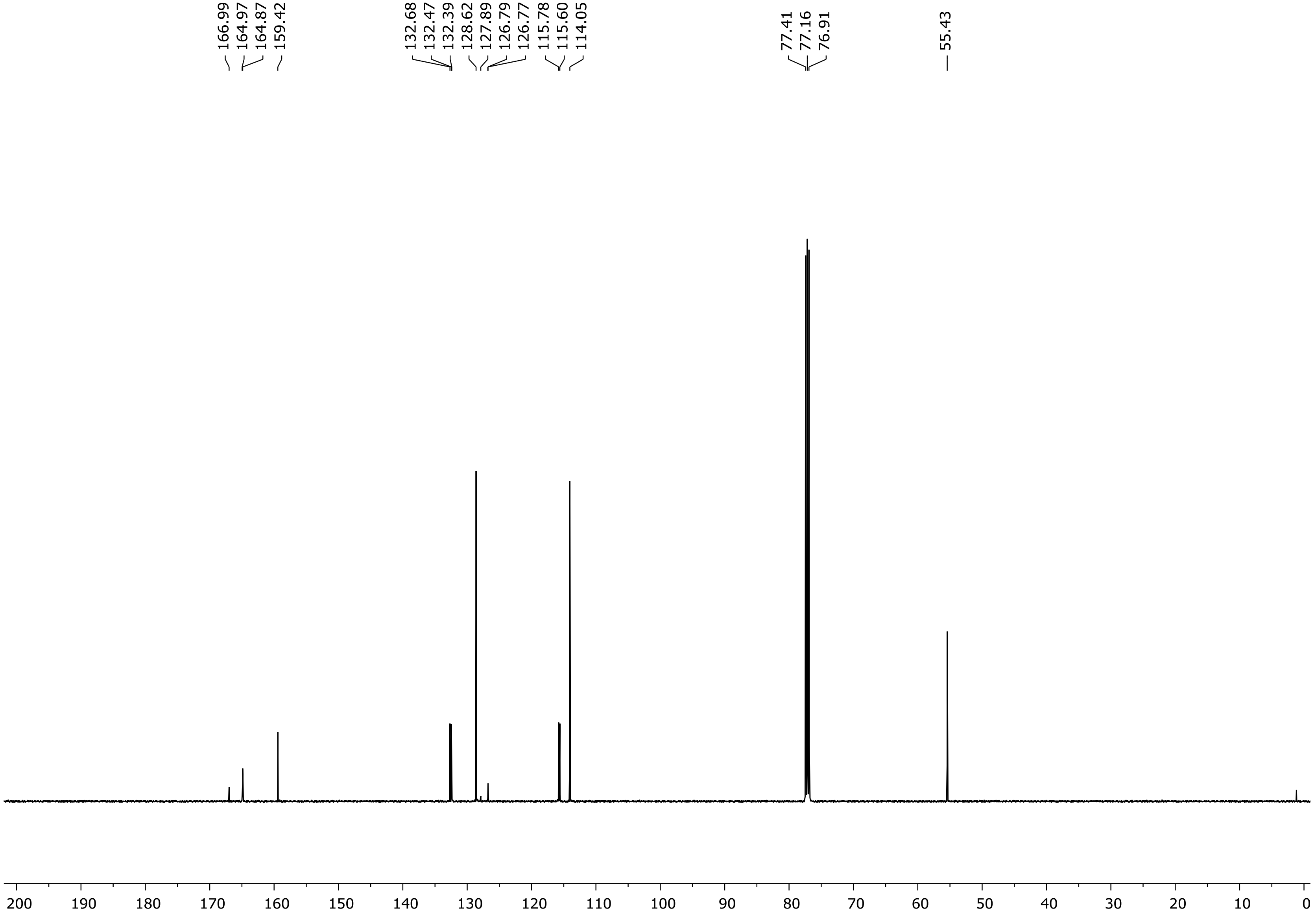

Figure S19: **$^{19}$F NMR** (471 MHz, $CDCl_3$, 298 K) spectrum of **2d**.

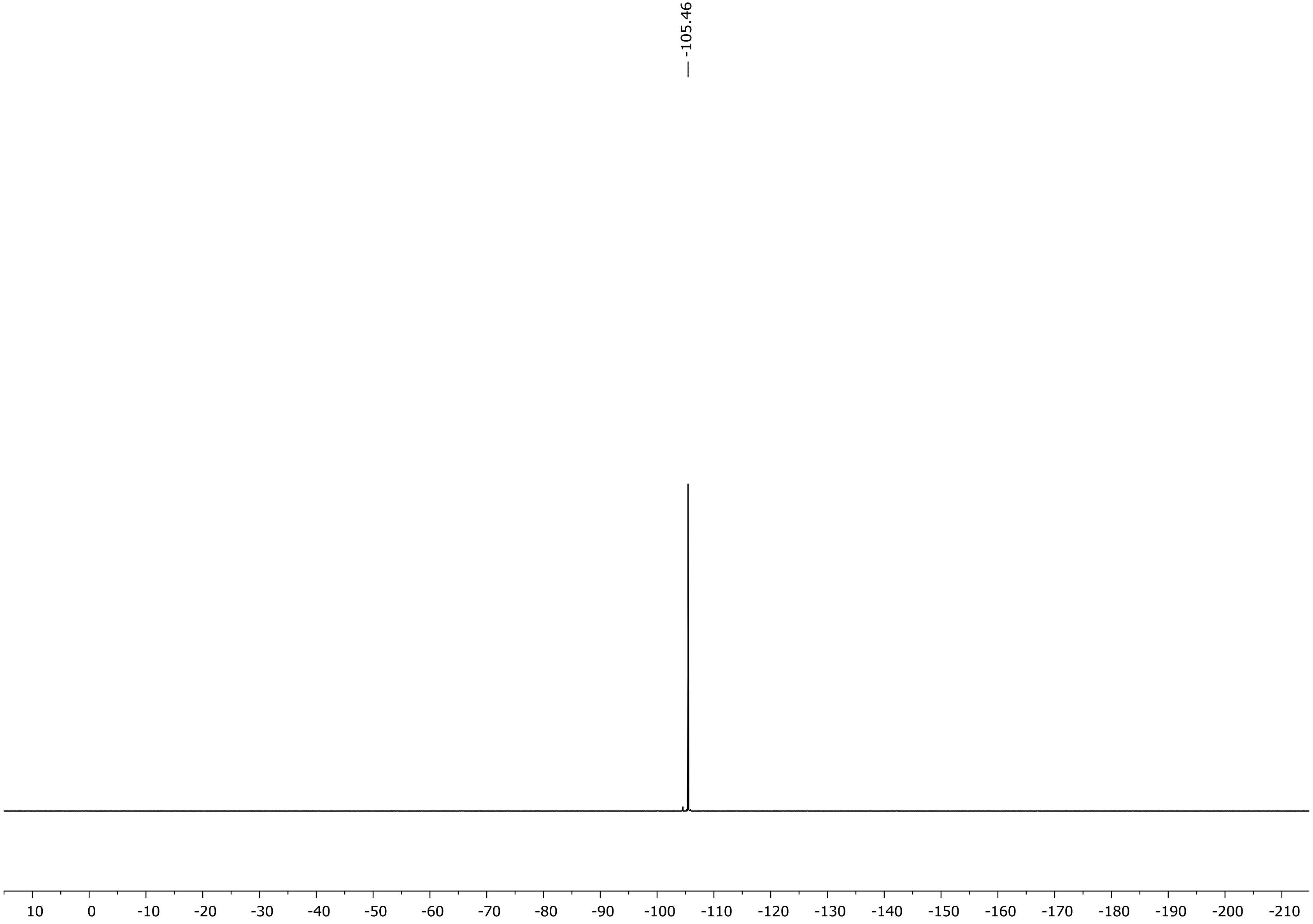

Figure S20: **¹H NMR** (500 MHz, $CDCl_3$, 298 K) spectrum of **2e**.

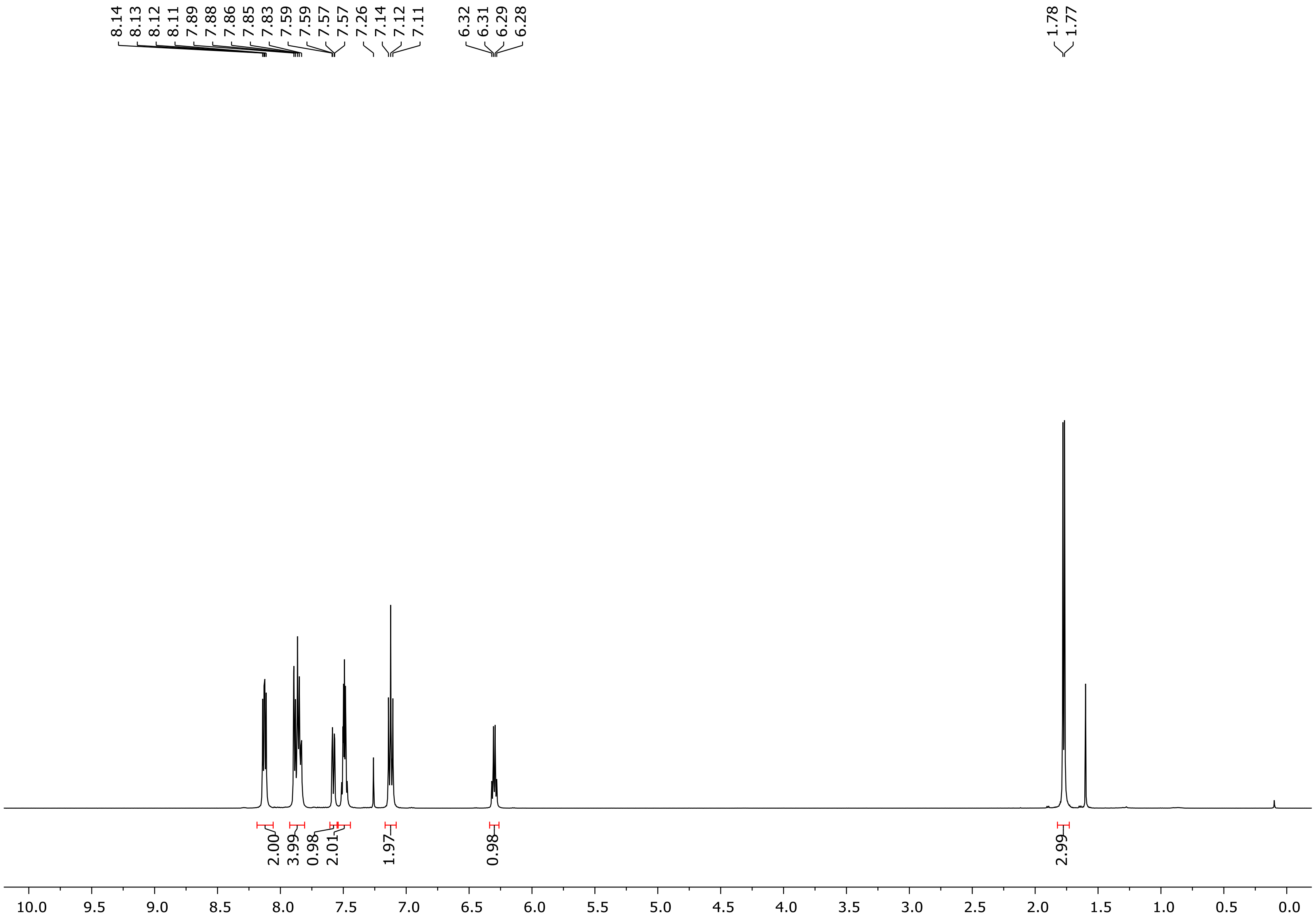

Figure S21: **$^{13}$C NMR** (126 MHz, $CDCl_3$, 298 K) spectrum of **2e**.

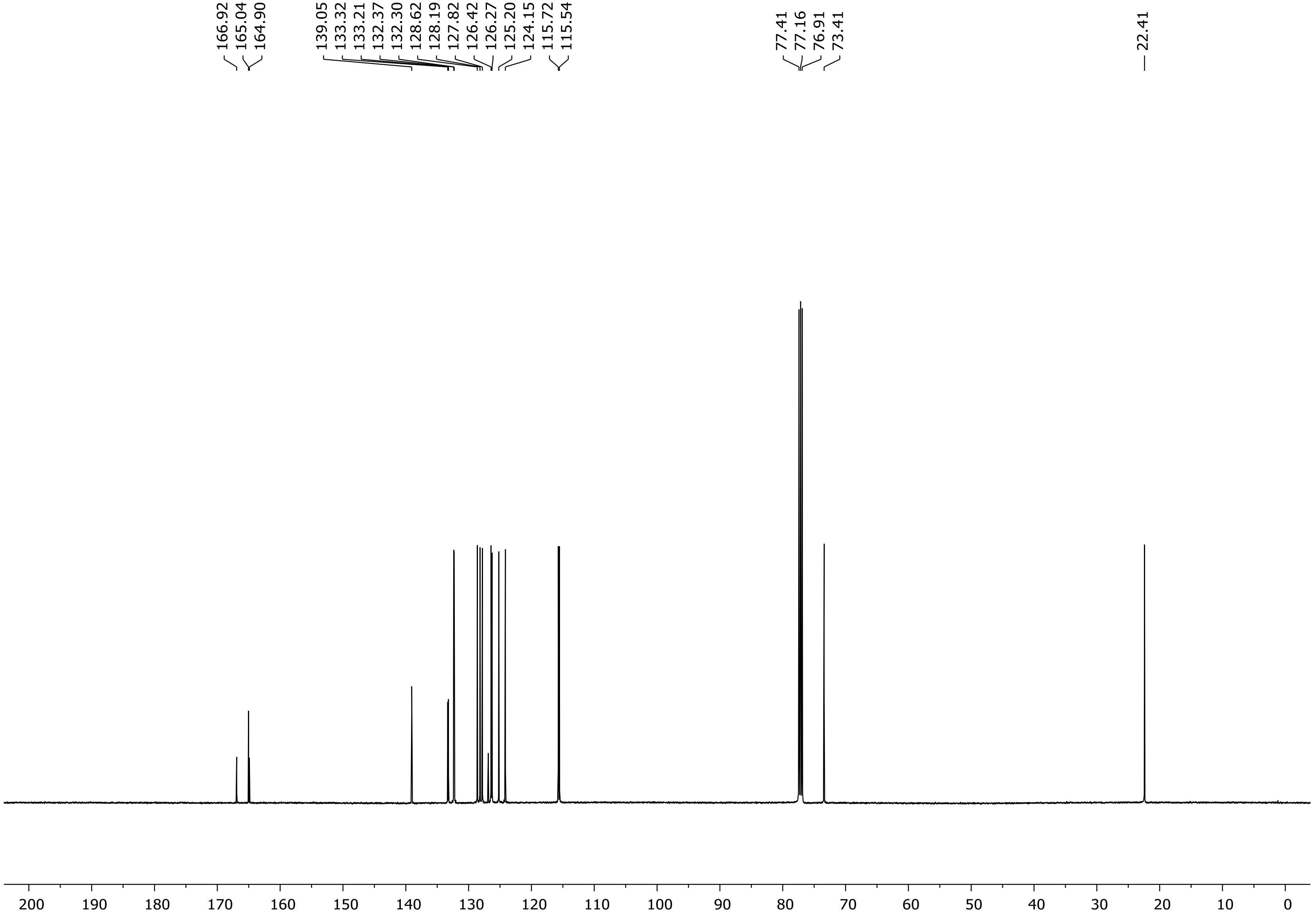

Figure S22: $^{19}$**F NMR** (471 MHz, $CDCl_3$, 298 K) spectrum of **2e**.

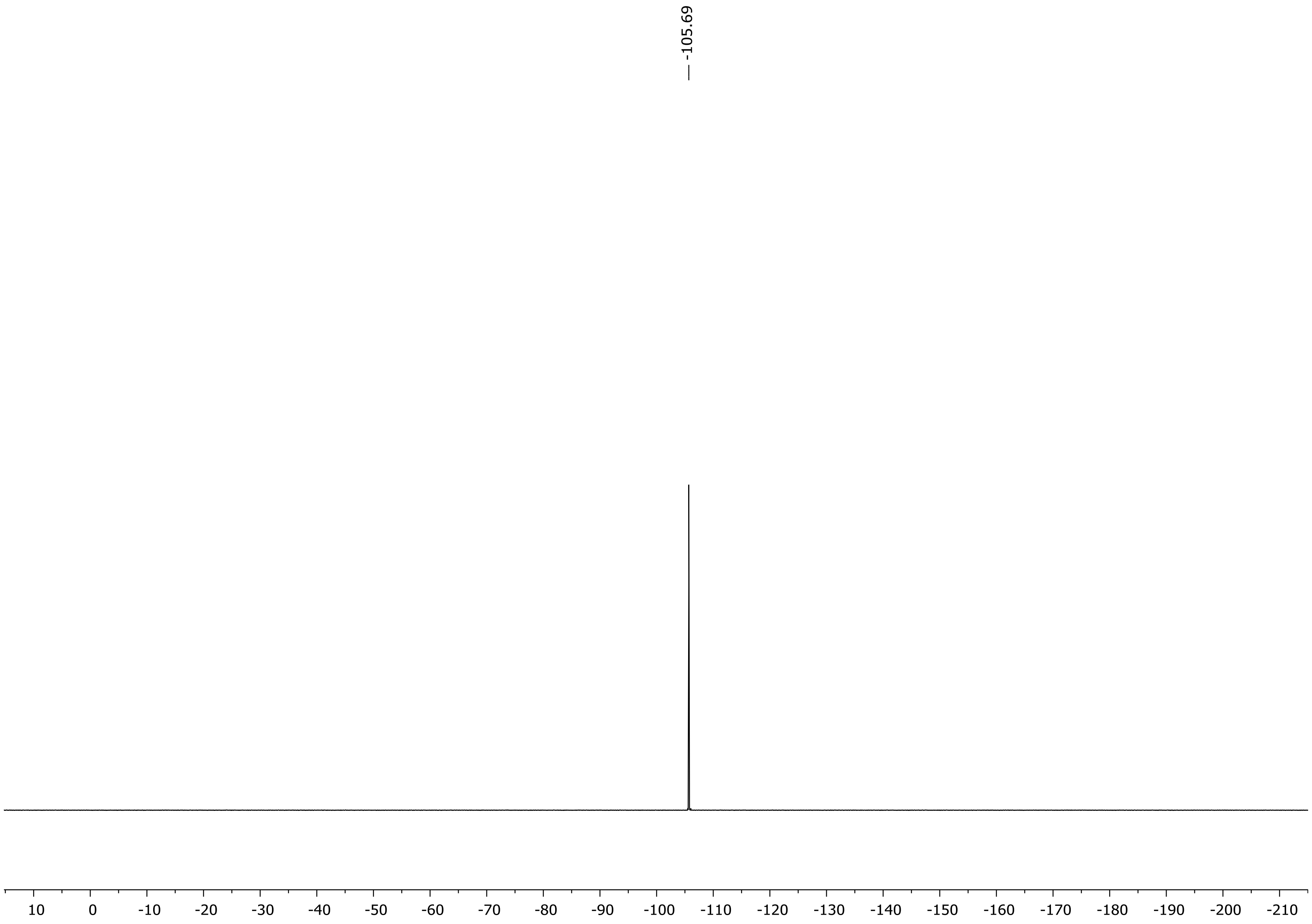

Figure S23: **$^1$H NMR** (500 MHz, $CDCl_3$, 298 K) spectrum of **2f.**

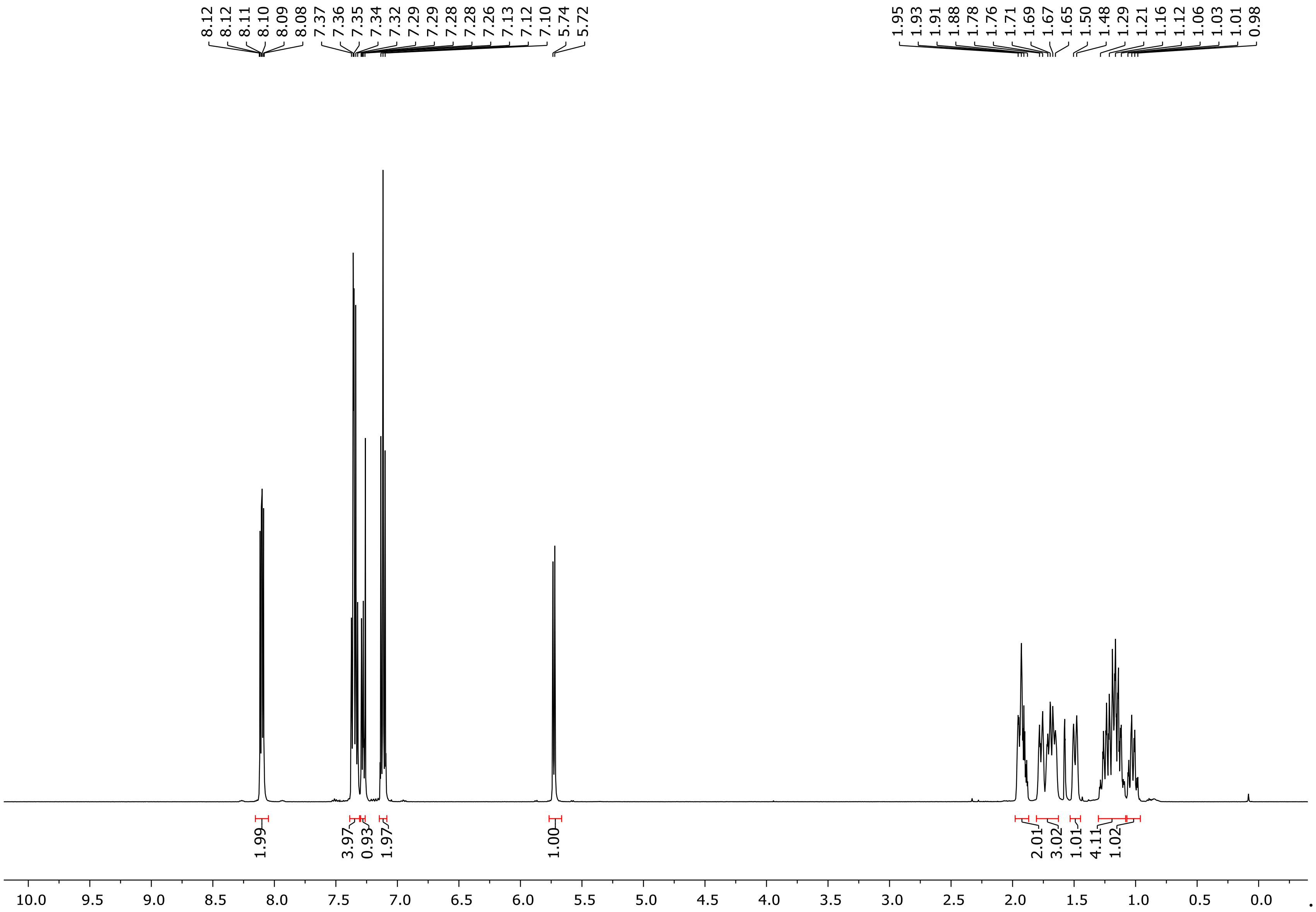

Figure S24: **$^{13}$C NMR** (126 MHz, $CDCl_3$, 298 K) spectrum of **2f**.

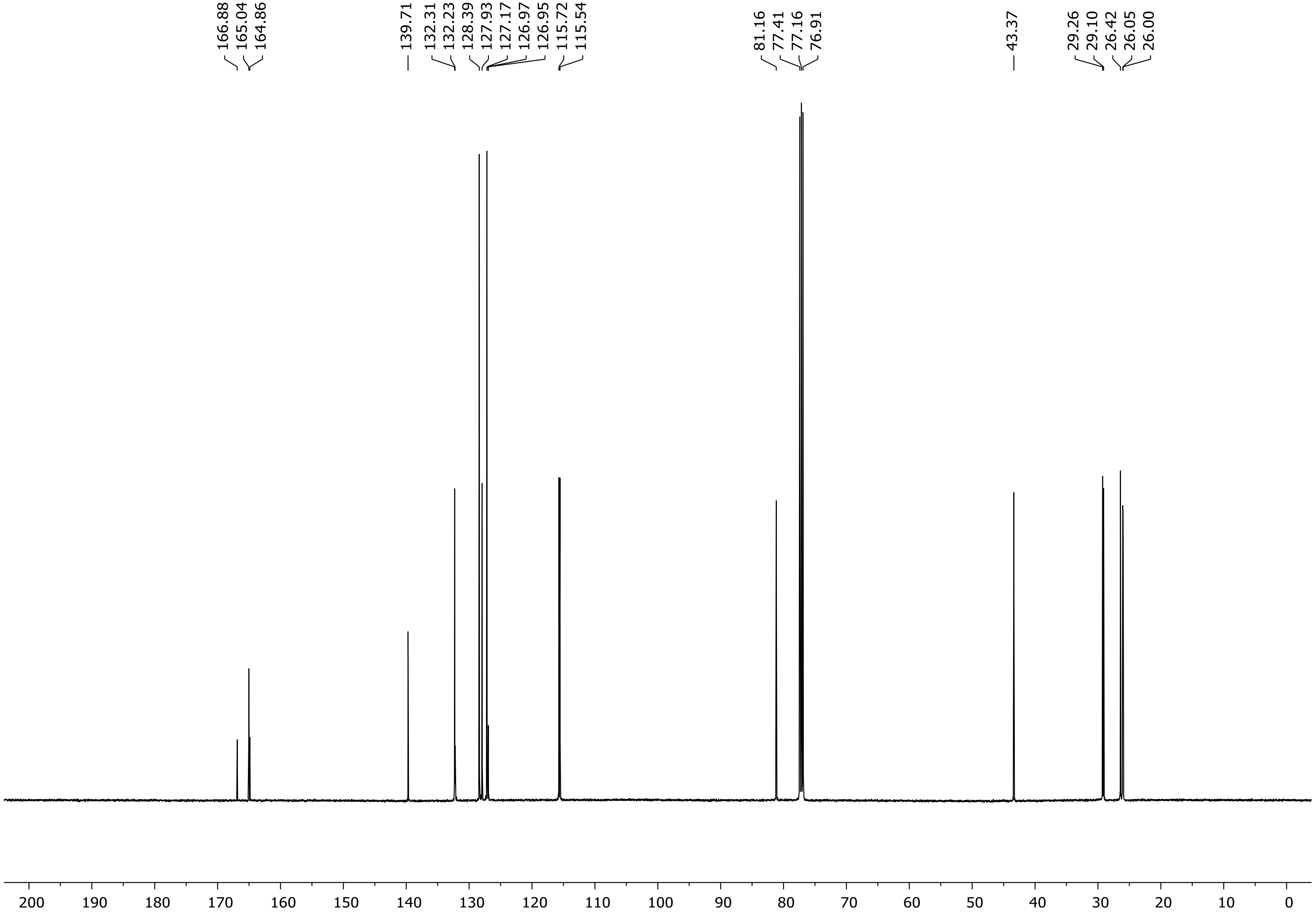

Figure S25: $^{19}$**F NMR** (471 MHz, $CDCl_3$, 298 K) spectrum of **2f**.

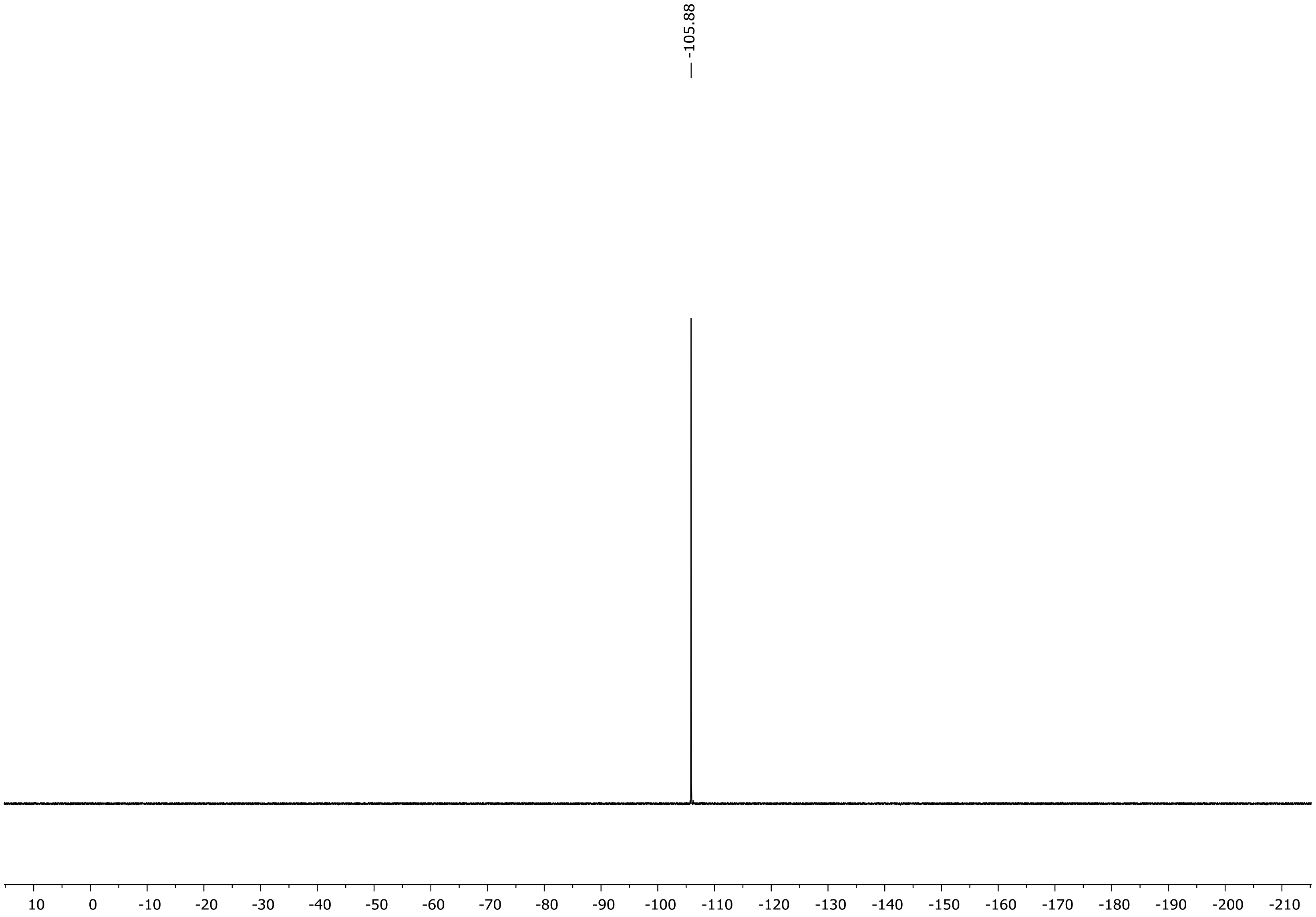

Figure S26: **$^1$H NMR** (500 MHz, $CDCl_3$, 298 K) spectrum of **2g.**

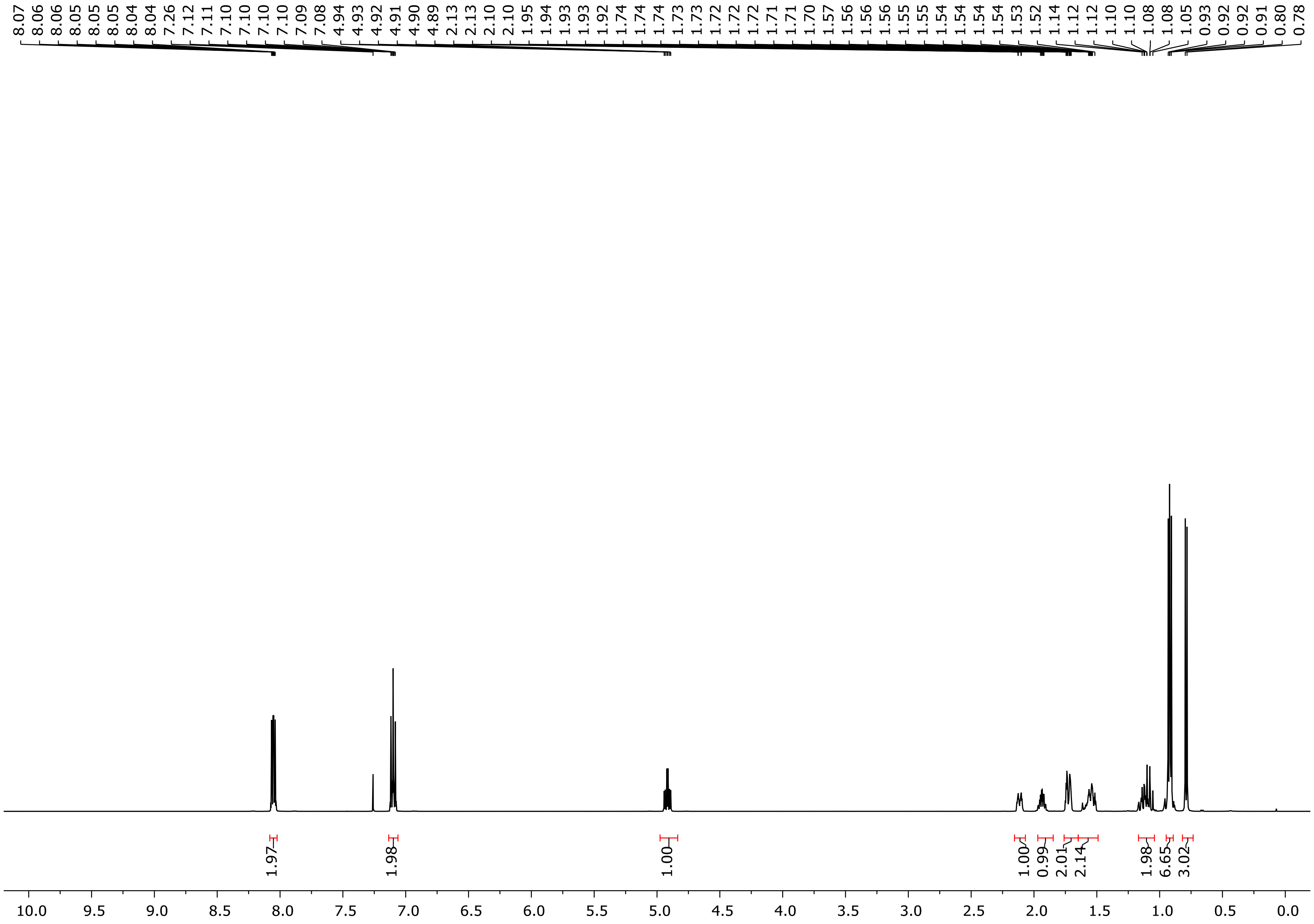

Figure S27: **$^{13}$C NMR** (126 MHz, $CDCl_3$, 298 K) spectrum of **2g**.

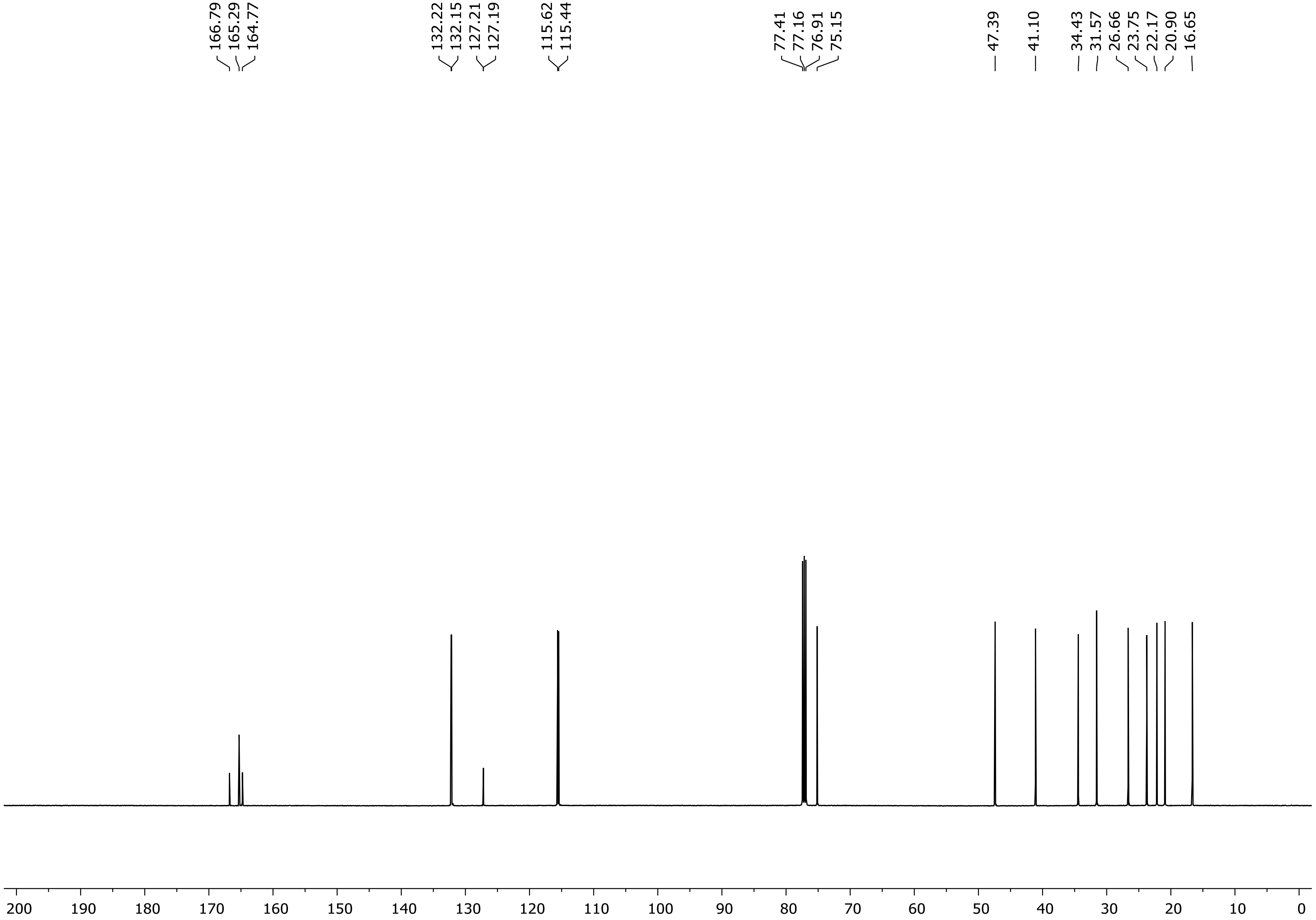

Figure S28: **$^{19}$F NMR** (471 MHz, $CDCl_3$, 298 K) spectrum of **2g**.

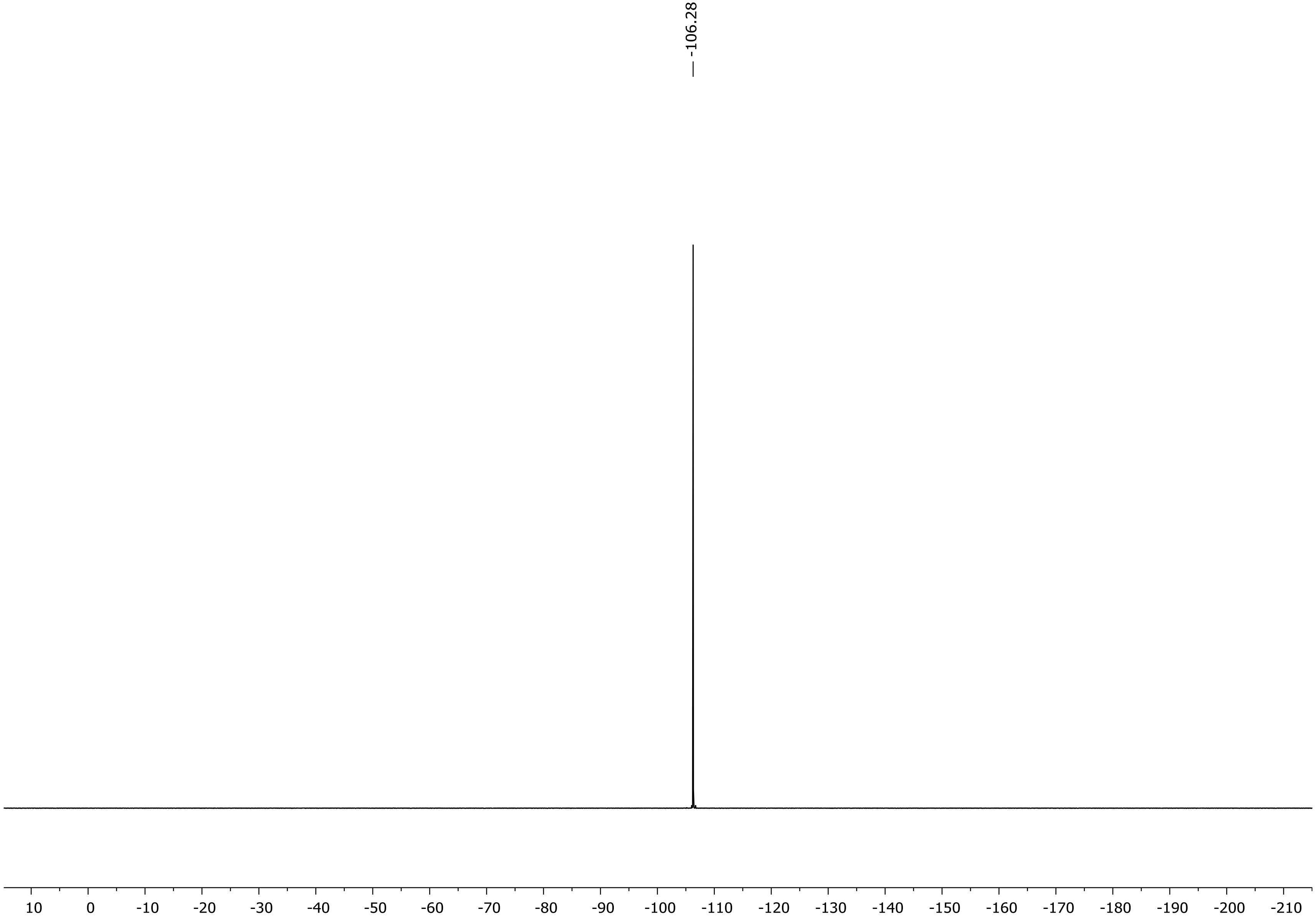

Figure S29: **$^{1}$H NMR** (500 MHz, $CDCl_3$, 298 K) spectrum of **3a**.

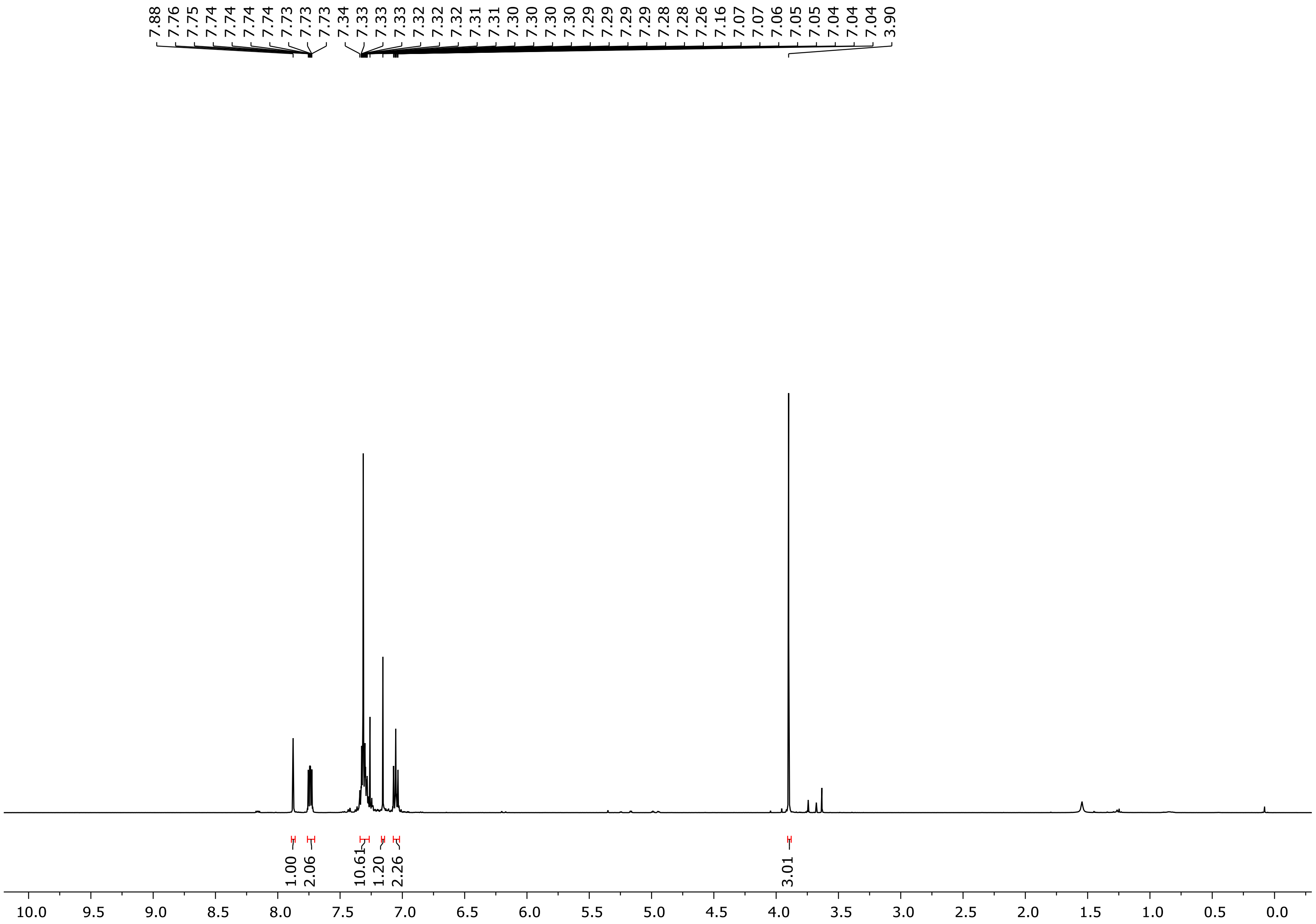

Figure S30: **$^{13}$C NMR** (126 MHz, $CDCl_3$, 298 K) spectrum of **3a**.

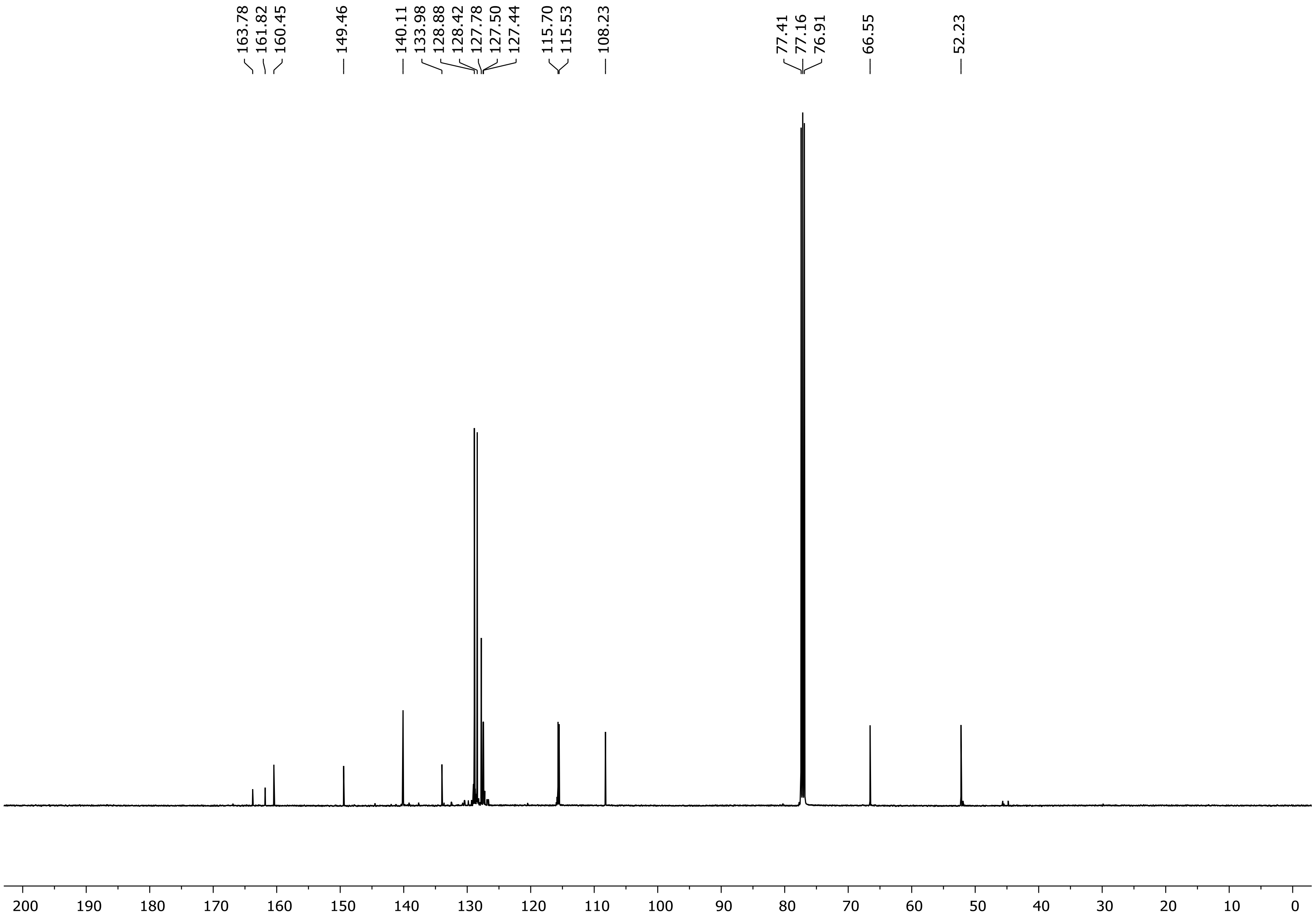

Figure S31: **$^{19}$F NMR** (471 MHz, $CDCl_3$, 298 K) spectrum of **3a**.

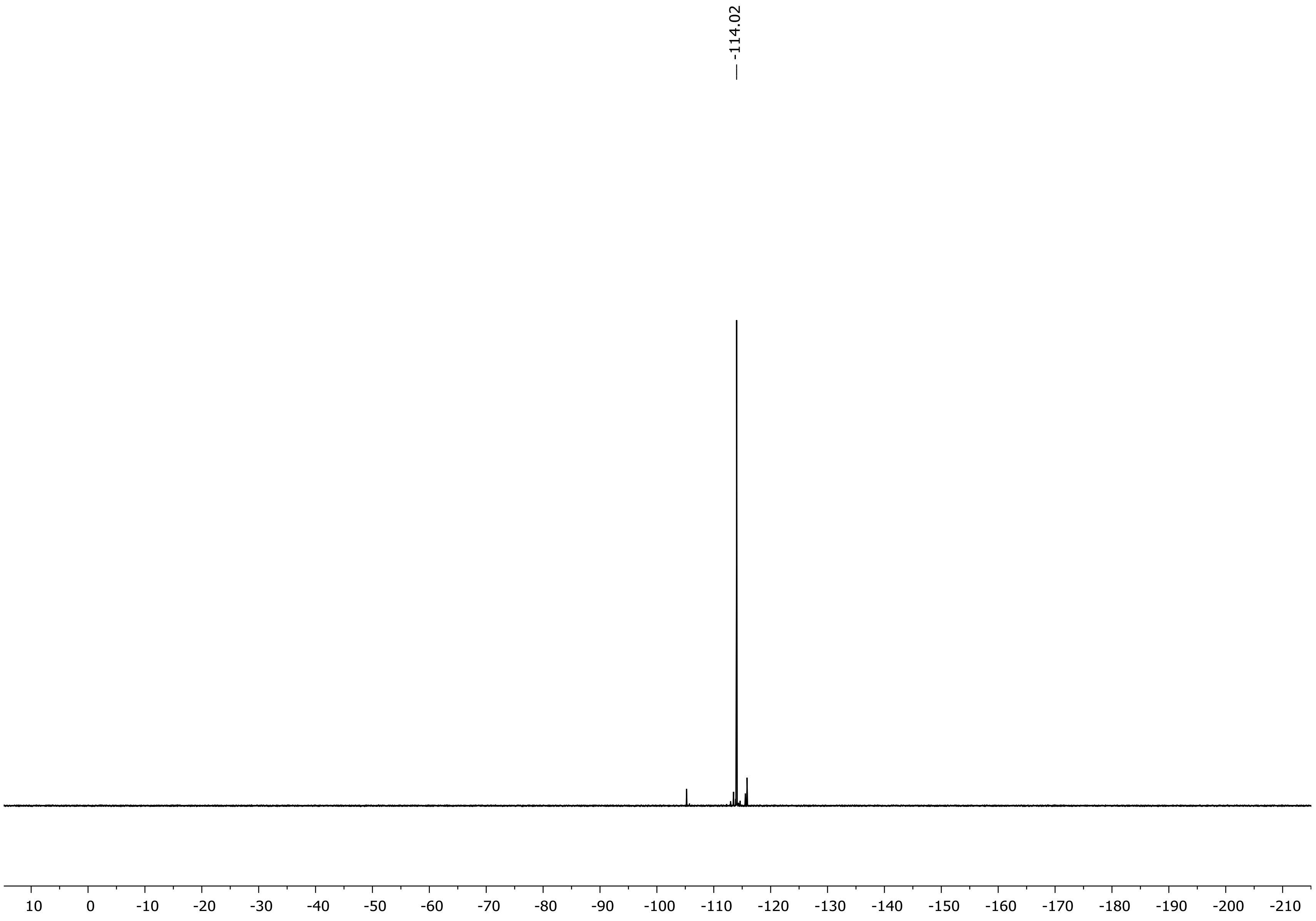

Figure S32: **$^{1}$H NMR** (500 MHz, $CDCl_3$, 298 K) spectrum of **3b**.

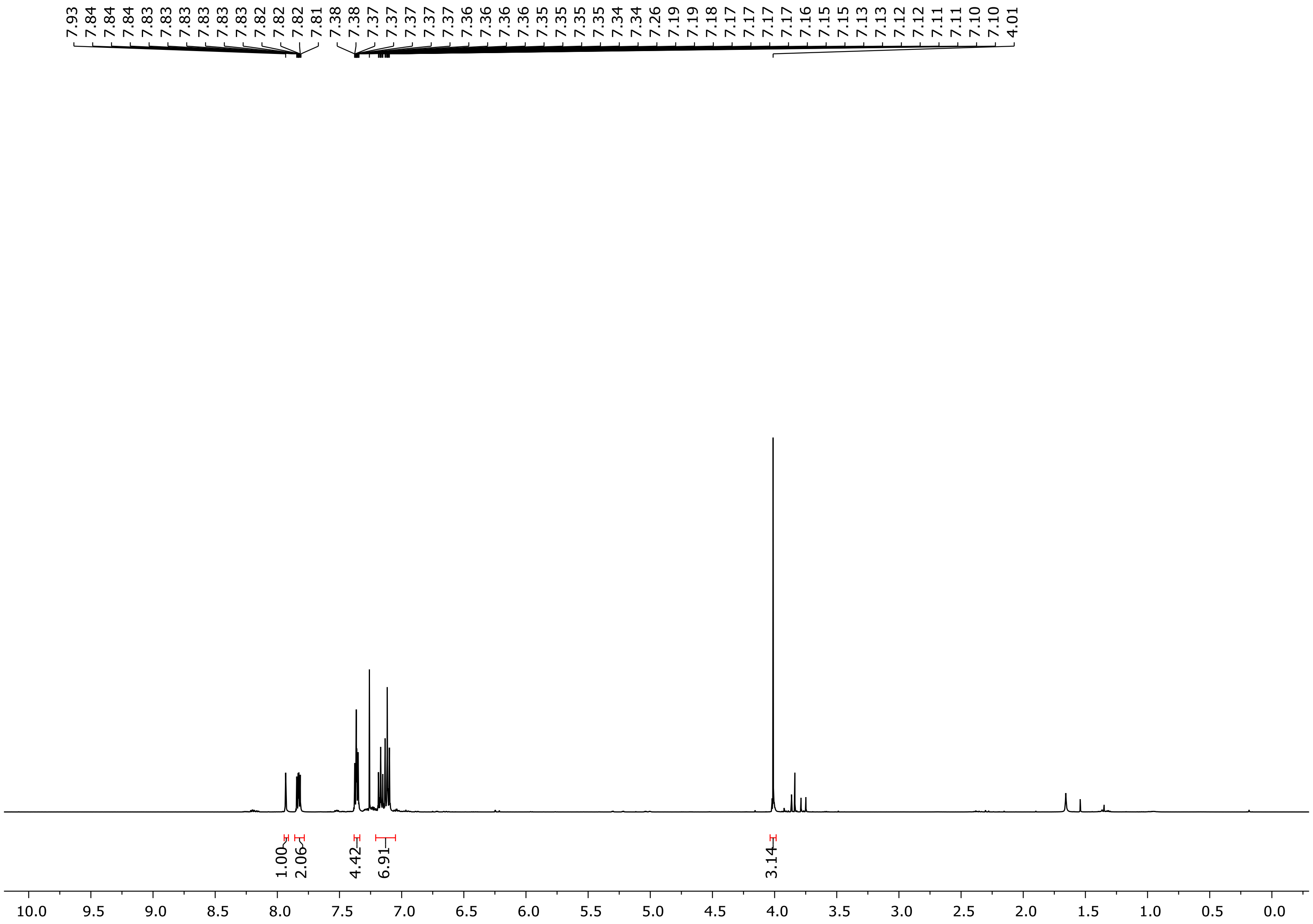

Figure S33: **$^{13}$C NMR** (126 MHz, $CDCl_3$, 298 K) spectrum of **3b**.

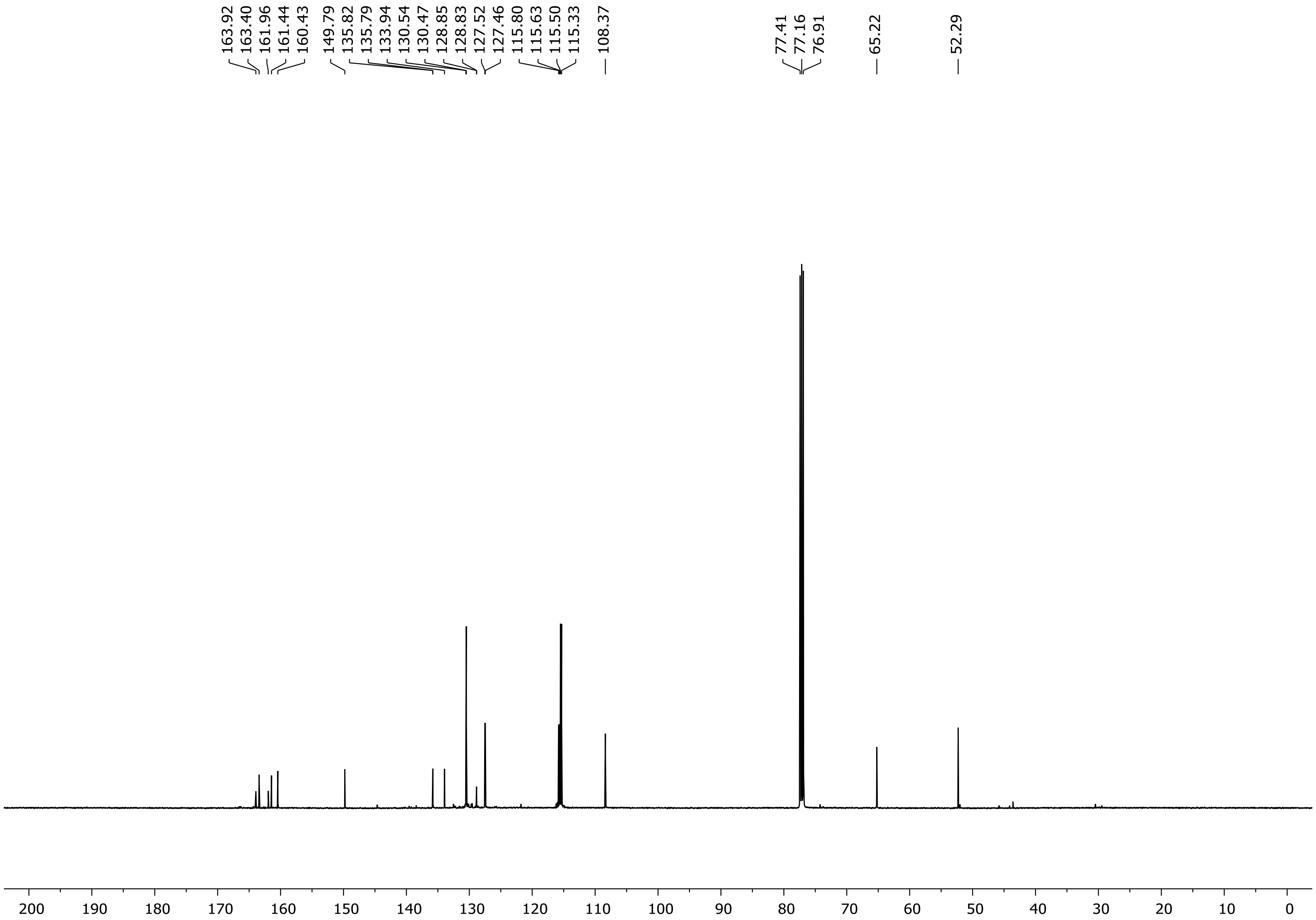

Figure S34: **$^{19}$F NMR** (471 MHz, $CDCl_3$, 298 K) spectrum of **3b**.

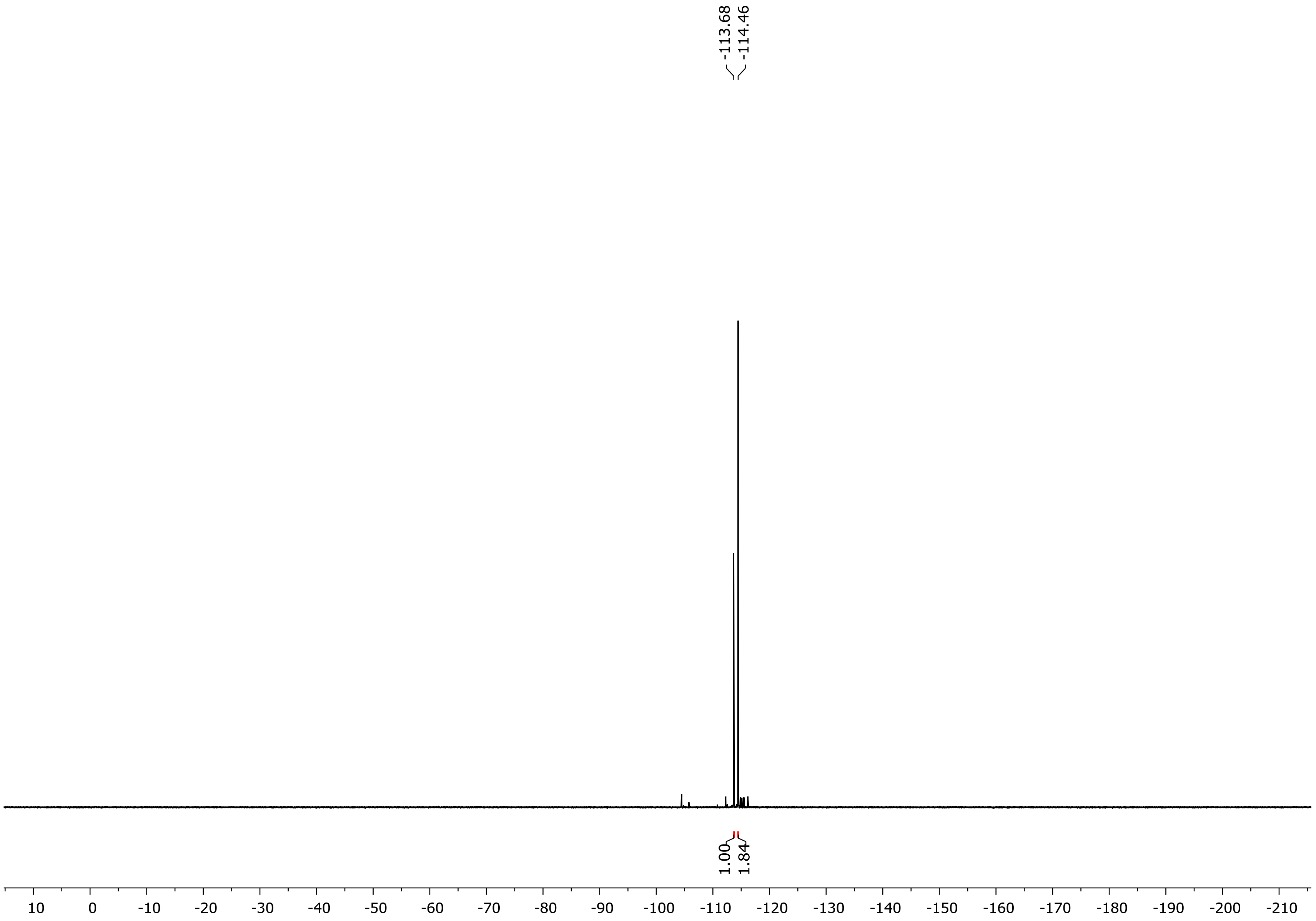

Figure S35: **$^{1}$H NMR** (500 MHz, $CDCl_3$, 298 K) spectrum of **3c**.

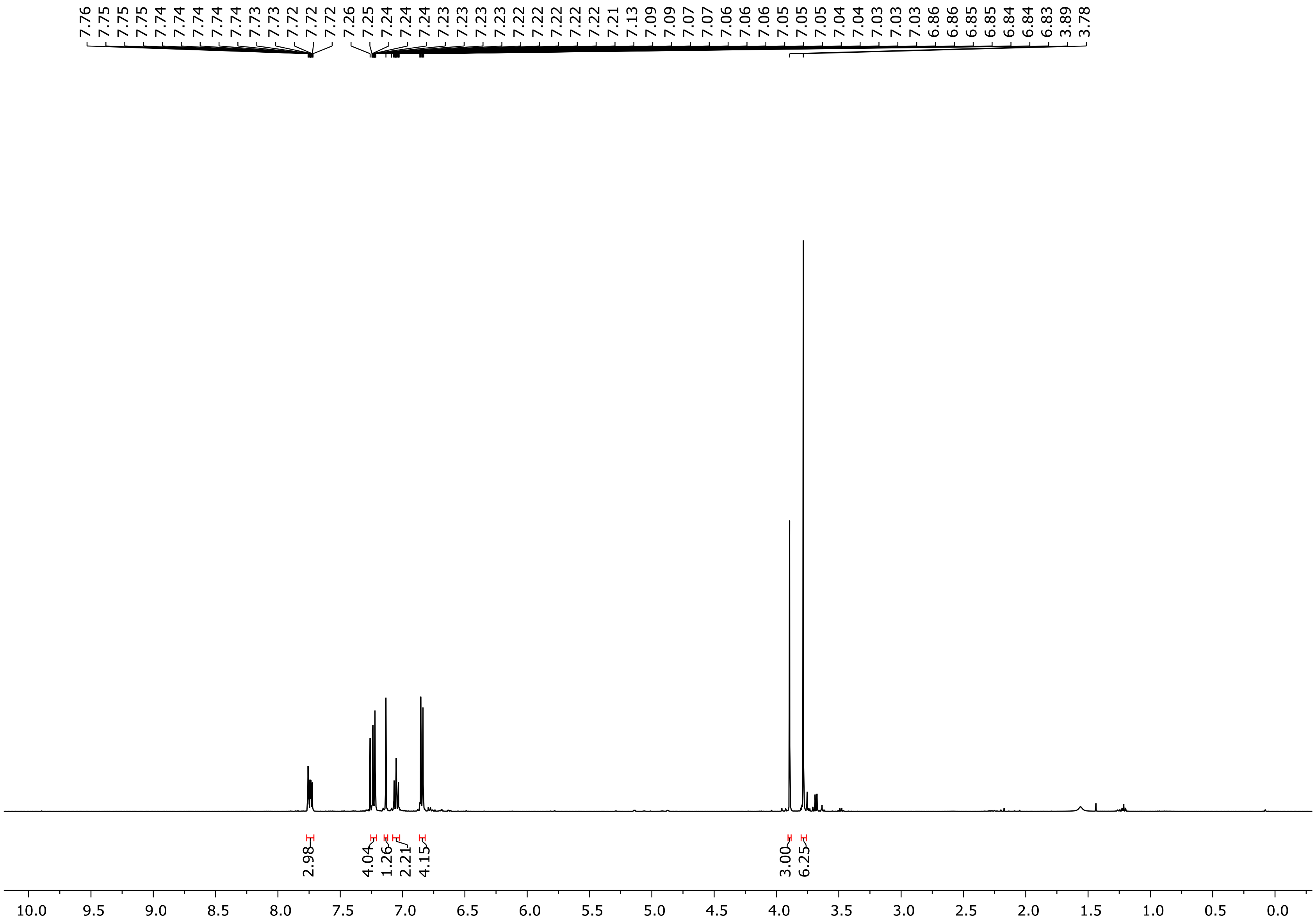

Figure S36: **$^{13}$C NMR** (126 MHz, $CDCl_3$, 298 K) spectrum of **3c**.

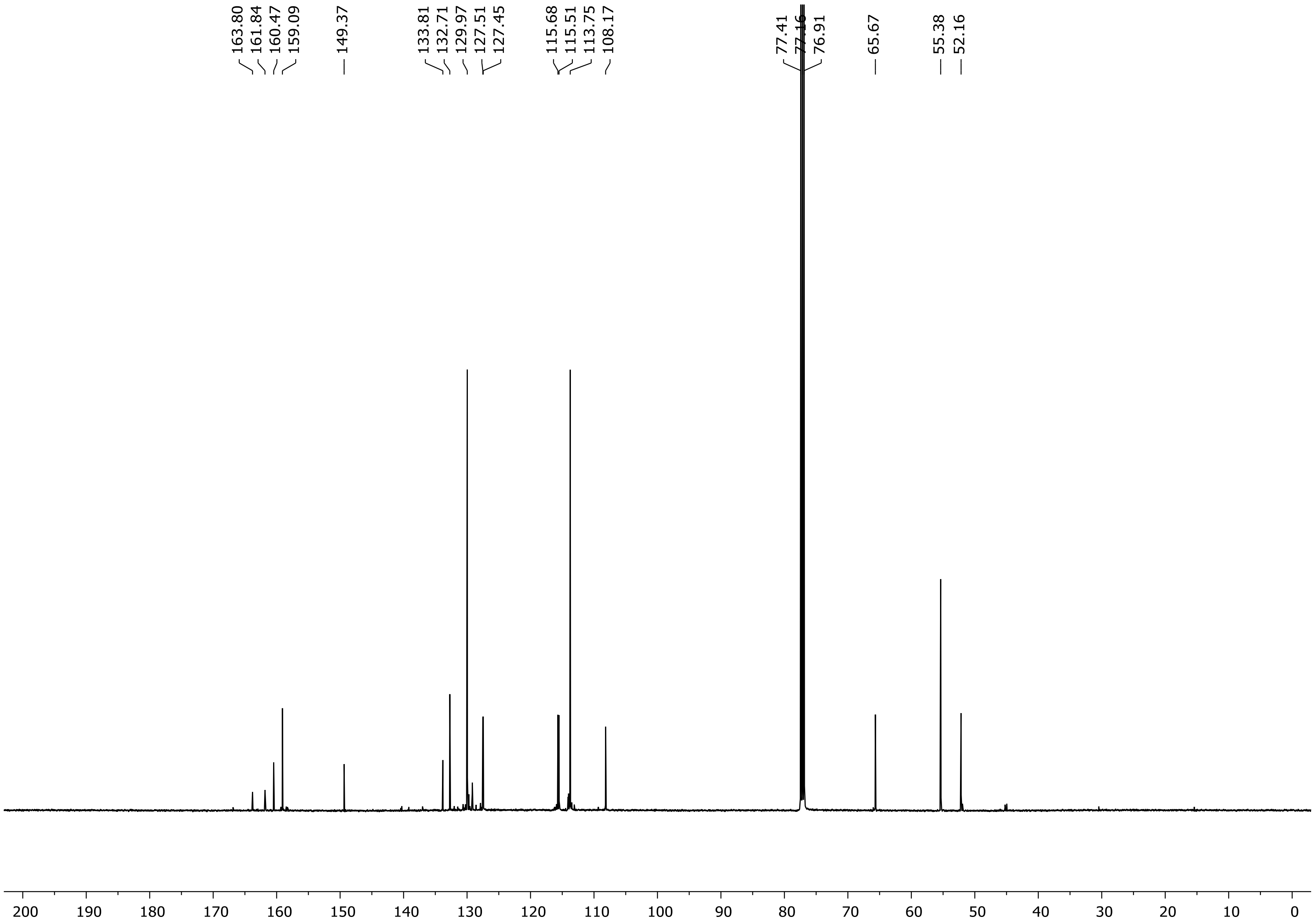

Figure S37: $^{19}$**F NMR** (471 MHz, $CDCl_3$, 298 K) spectrum of **3c**.

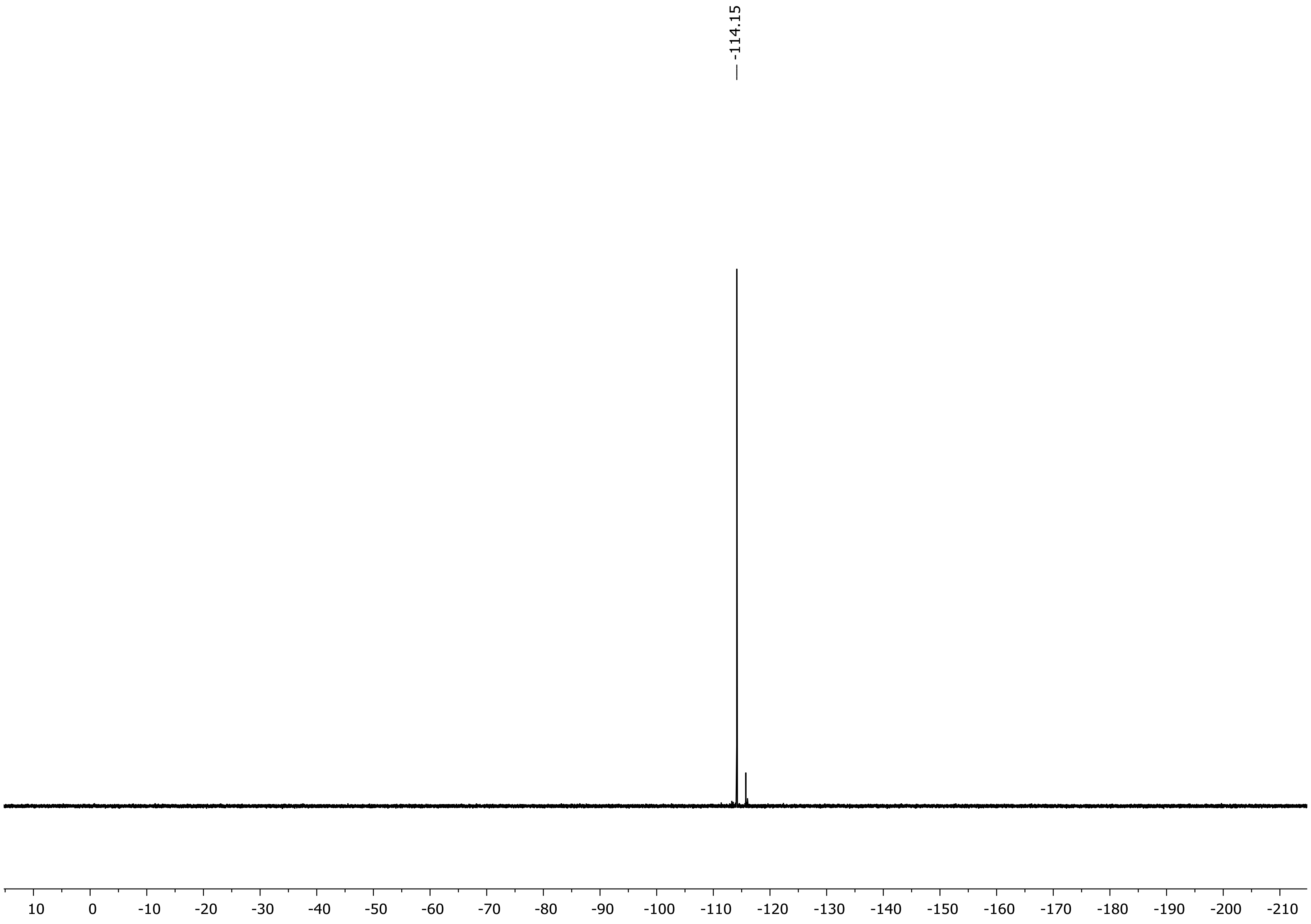

Figure S38: **$^{1}$H NMR** (500 MHz, $CDCl_3$, 298 K) spectrum of **3d**.

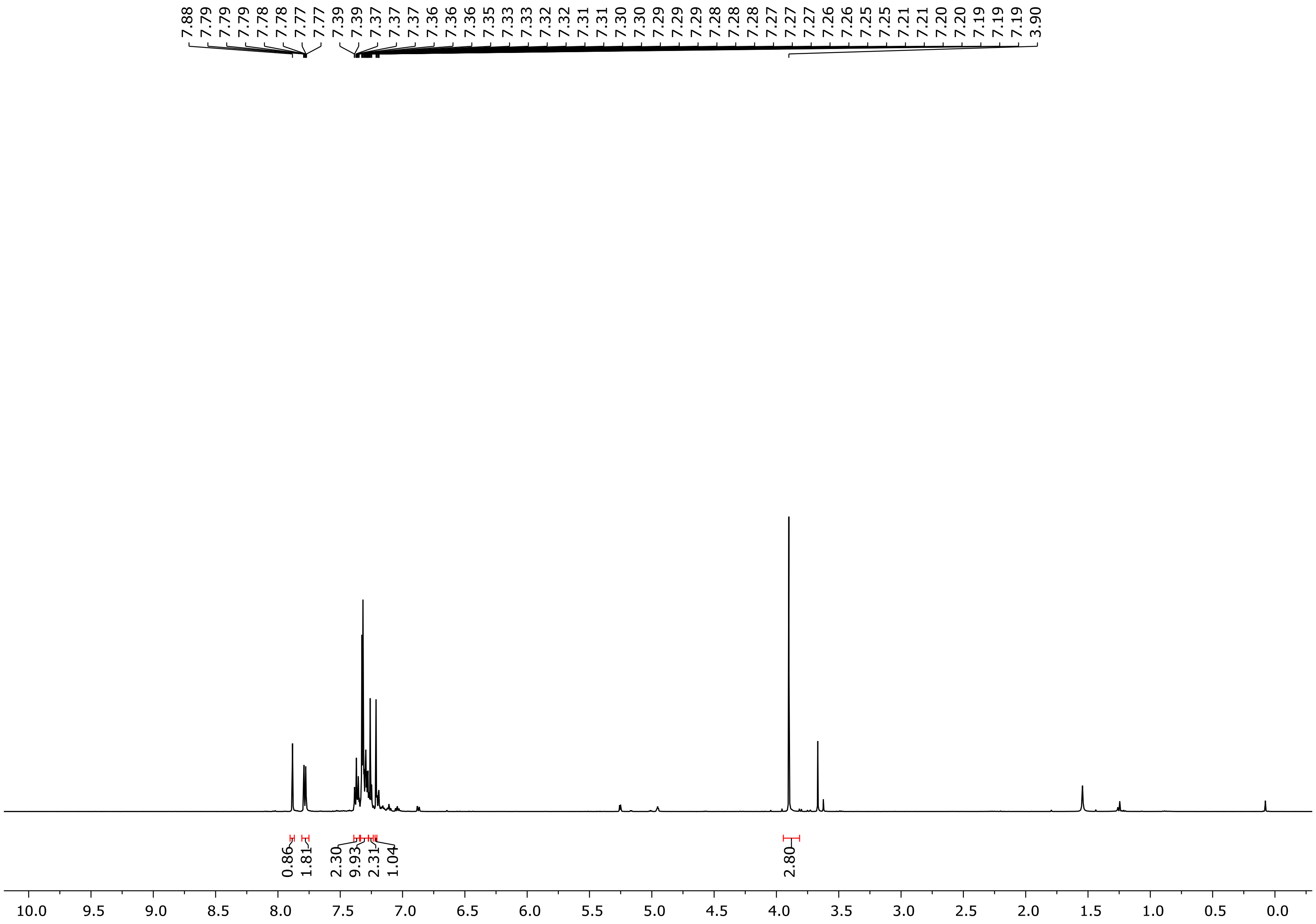

Figure S39: **$^{13}$C NMR** (126 MHz, $CDCl_3$, 298 K) spectrum of **3d**.

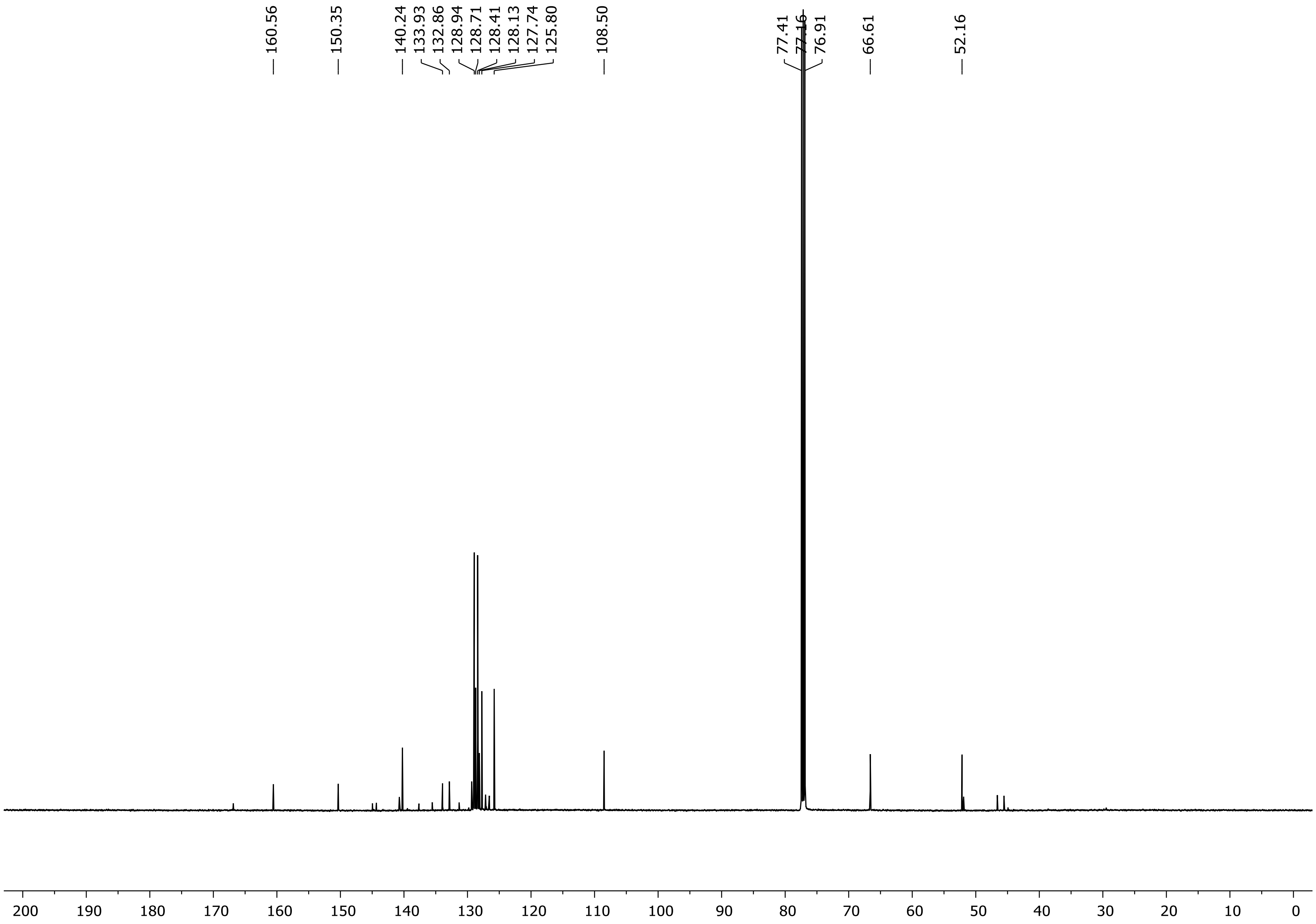

Figure S40: $^{1}$**H NMR** (500 MHz, $CDCl_3$, 298 K) spectrum of **3e**.

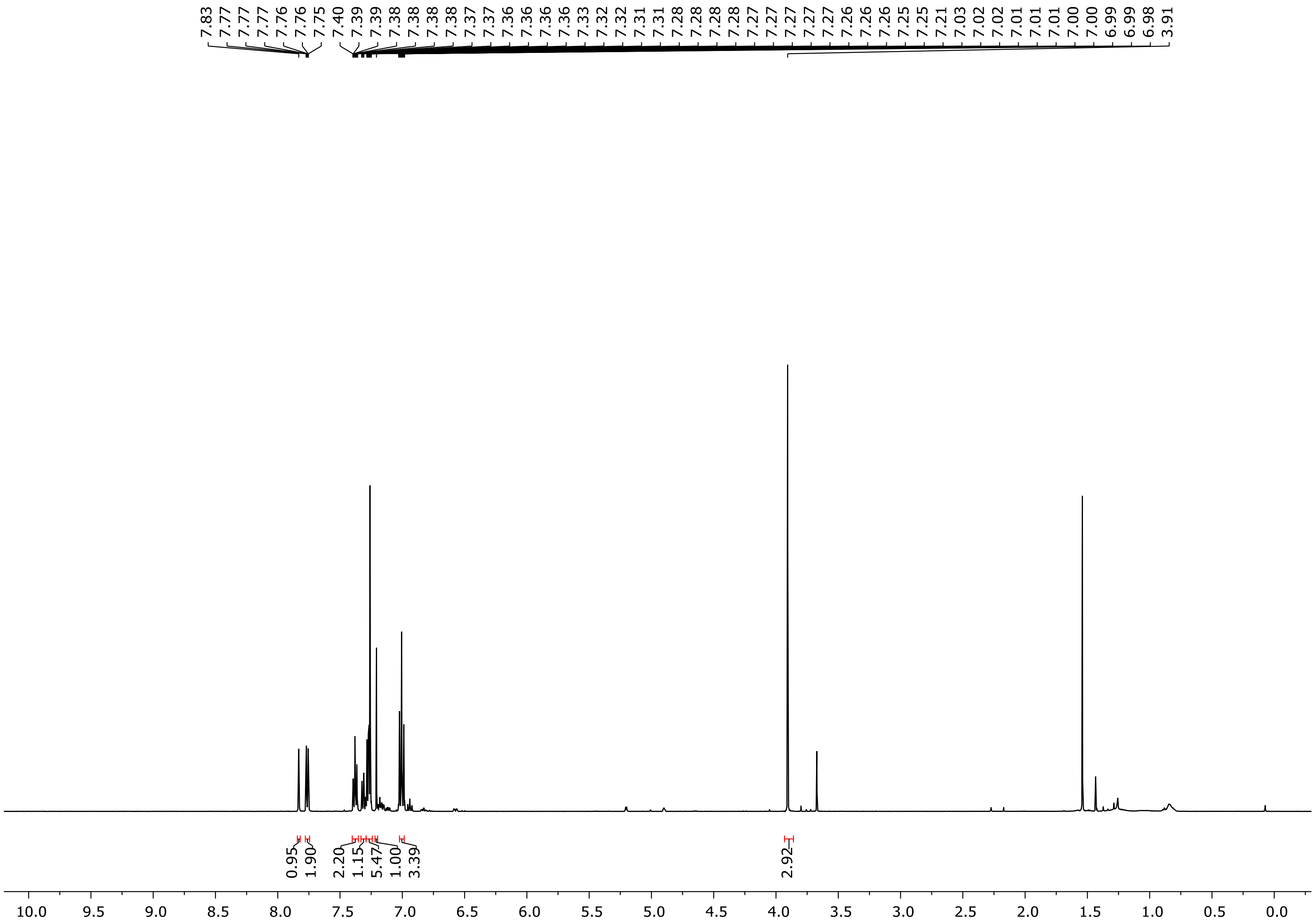

Figure S41: **$^{13}$C NMR** (126 MHz, $CDCl_3$, 298 K) spectrum of **3e**.

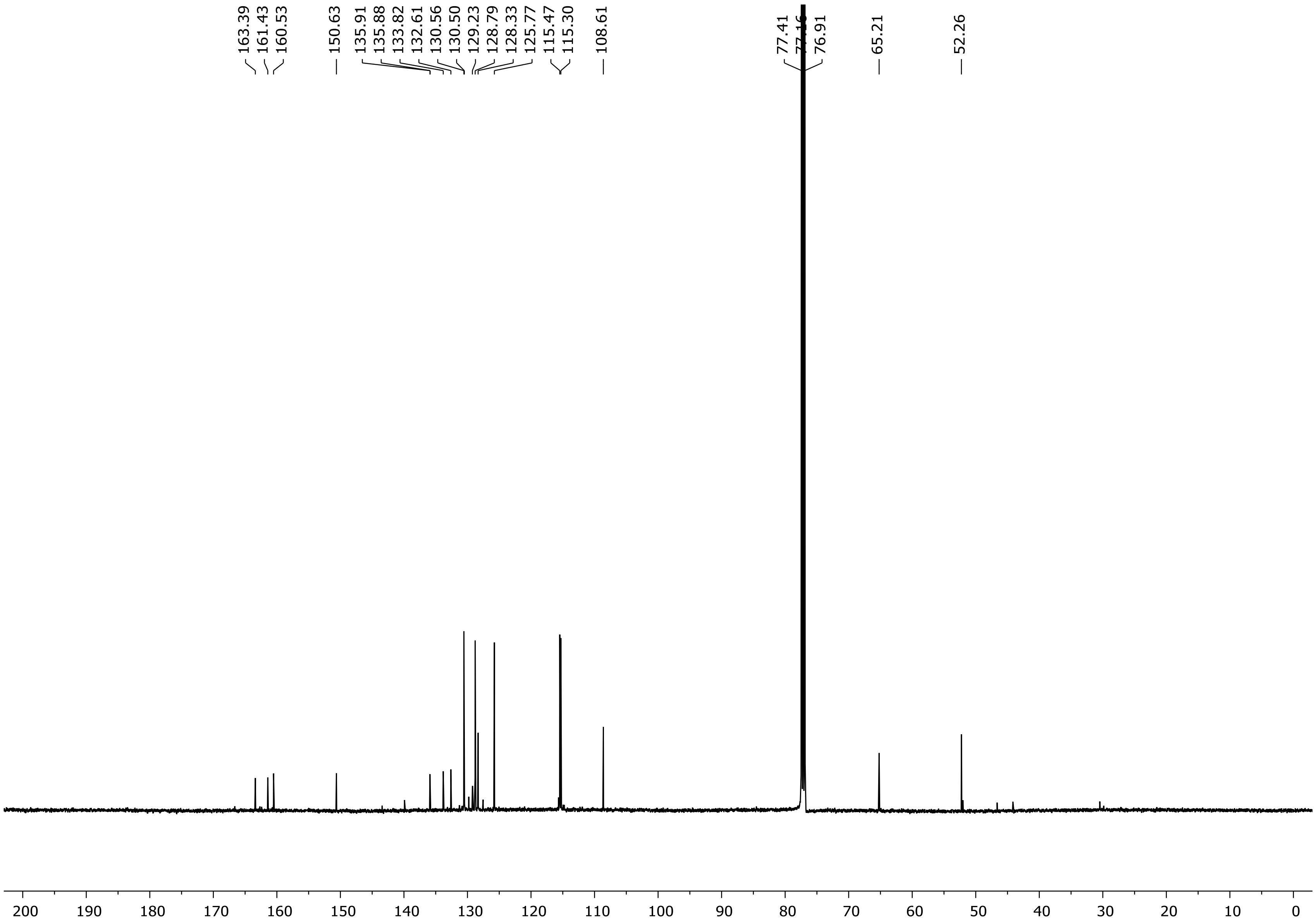

Figure S42: **$^{19}$F NMR** (471 MHz, $CDCl_3$, 298 K) spectrum of **3e**.

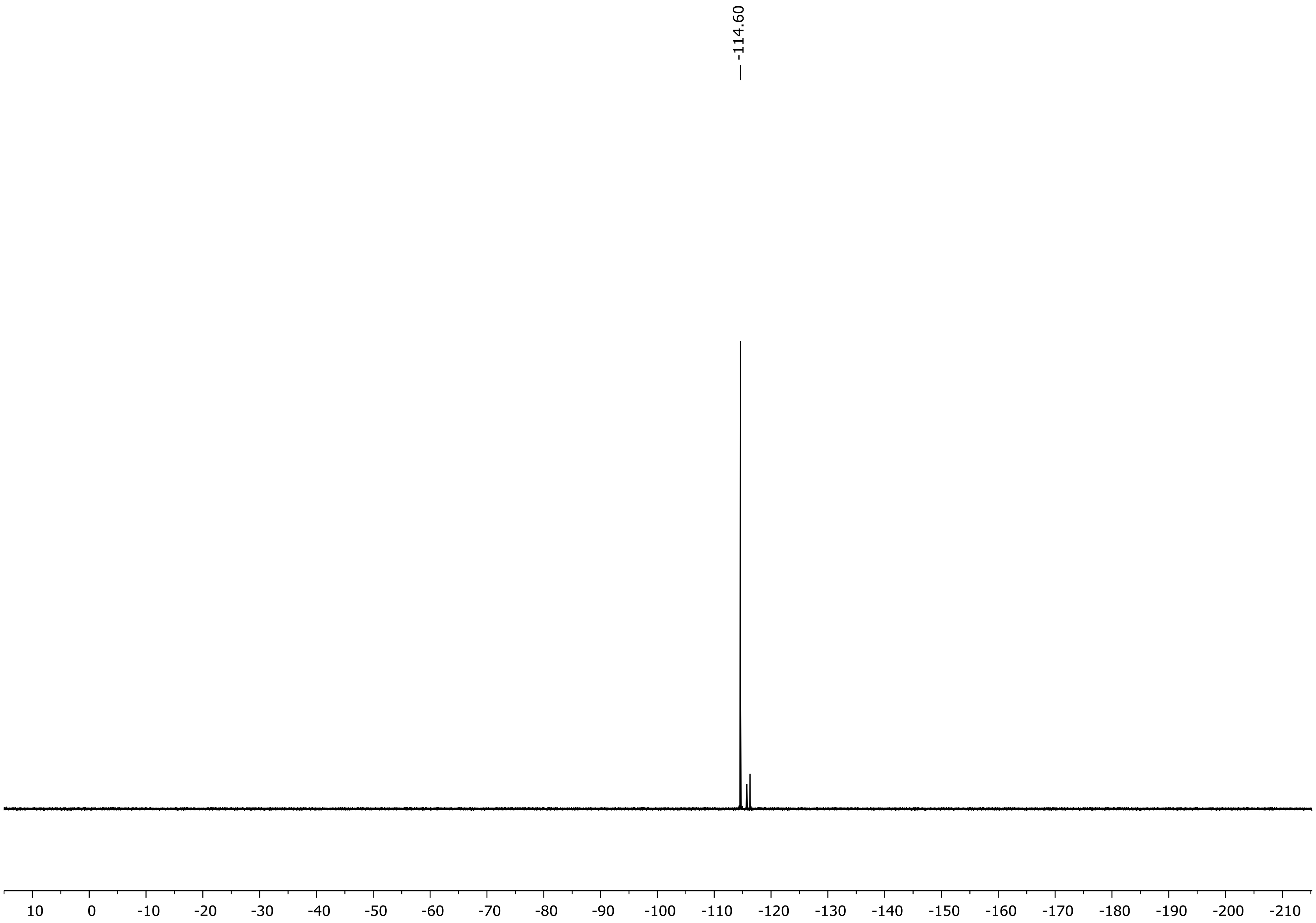

Figure S43: $^{1}$**H NMR** (500 MHz, $CDCl_3$, 298 K) spectrum of **3f**.

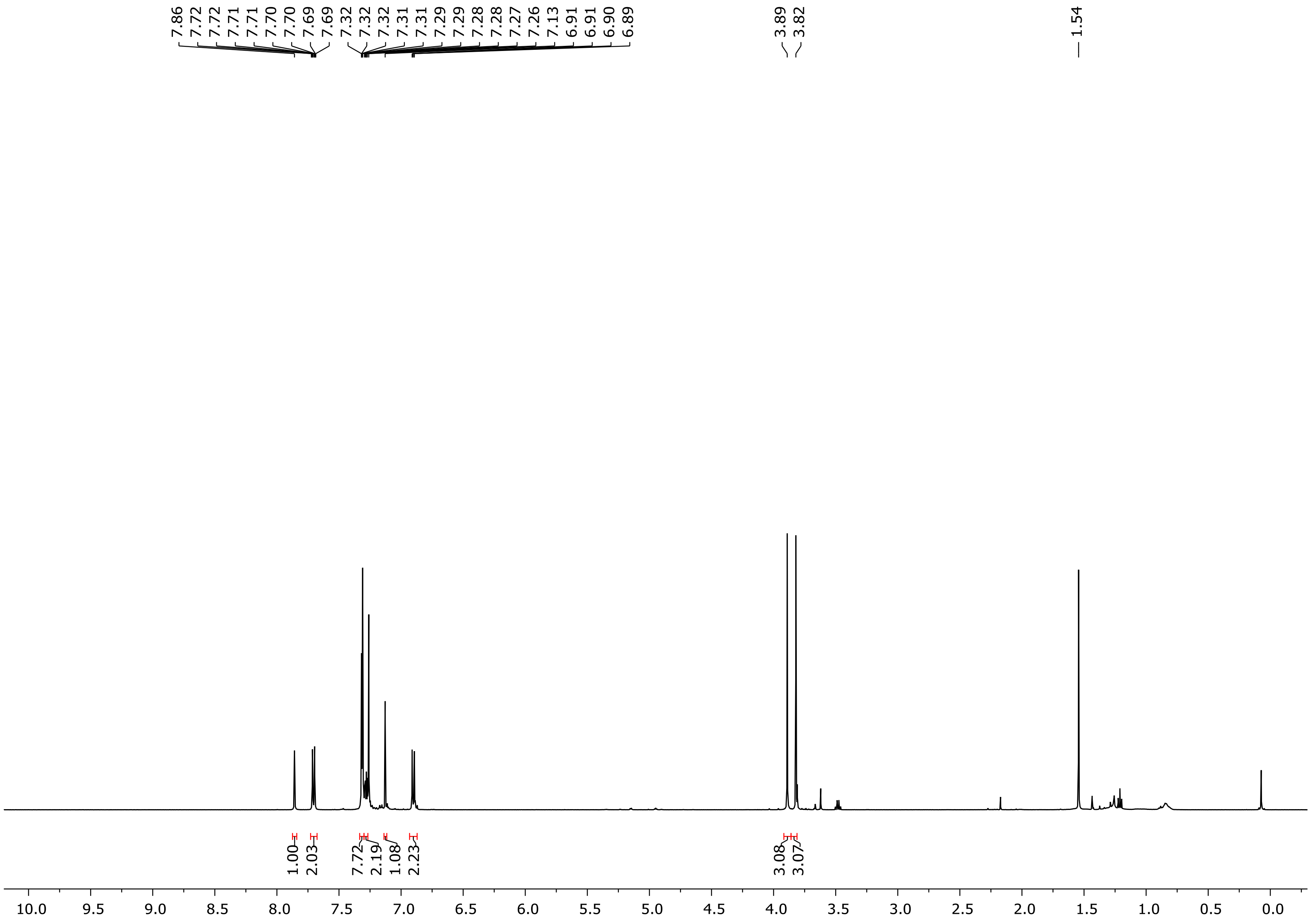

Figure S44: **$^{13}$C NMR** (126 MHz, $CDCl_3$, 298 K) spectrum of **3f**.

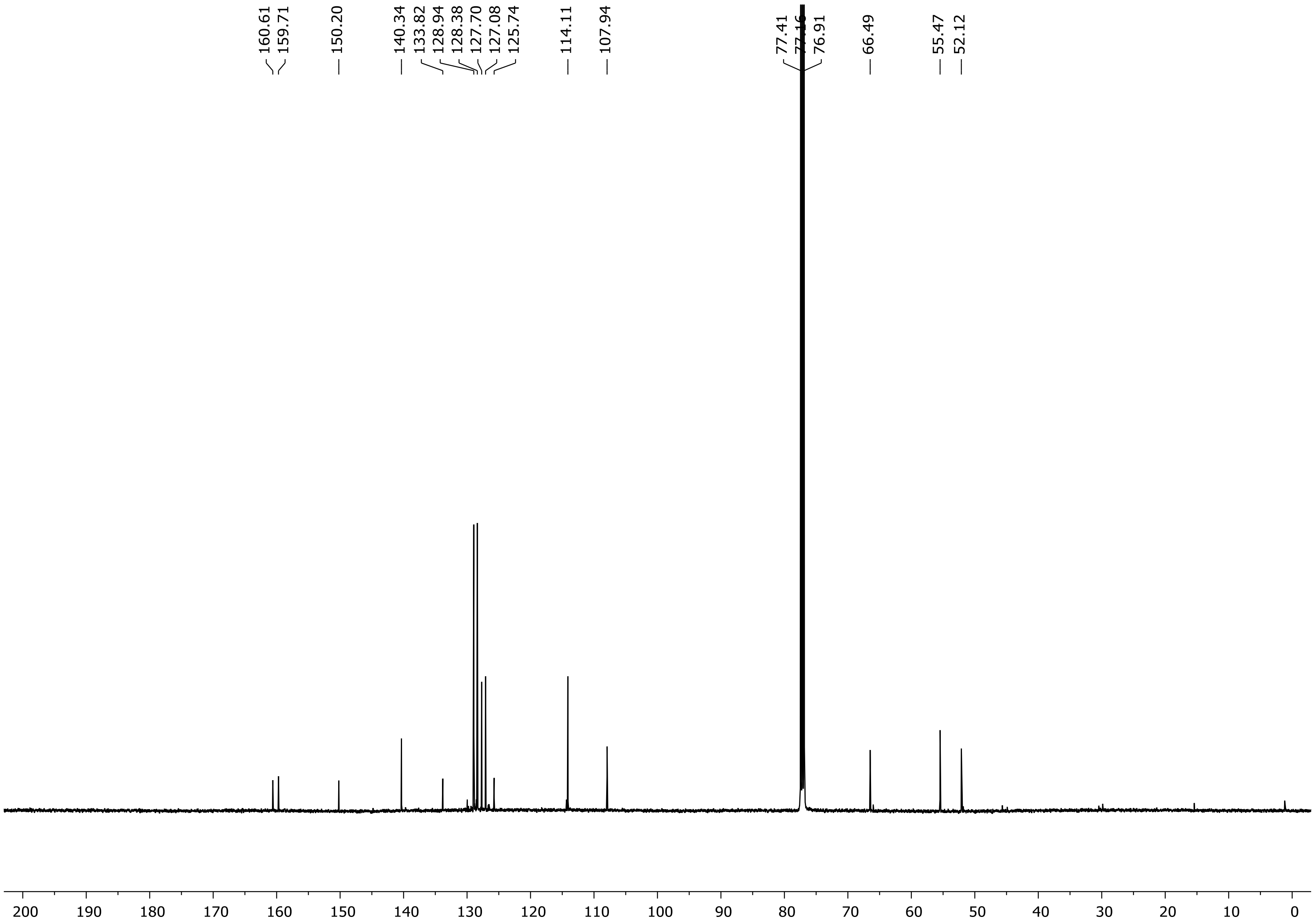

Figure S45: **$^{1}$H NMR** (500 MHz, $CDCl_3$, 298 K) spectrum of **3g**.

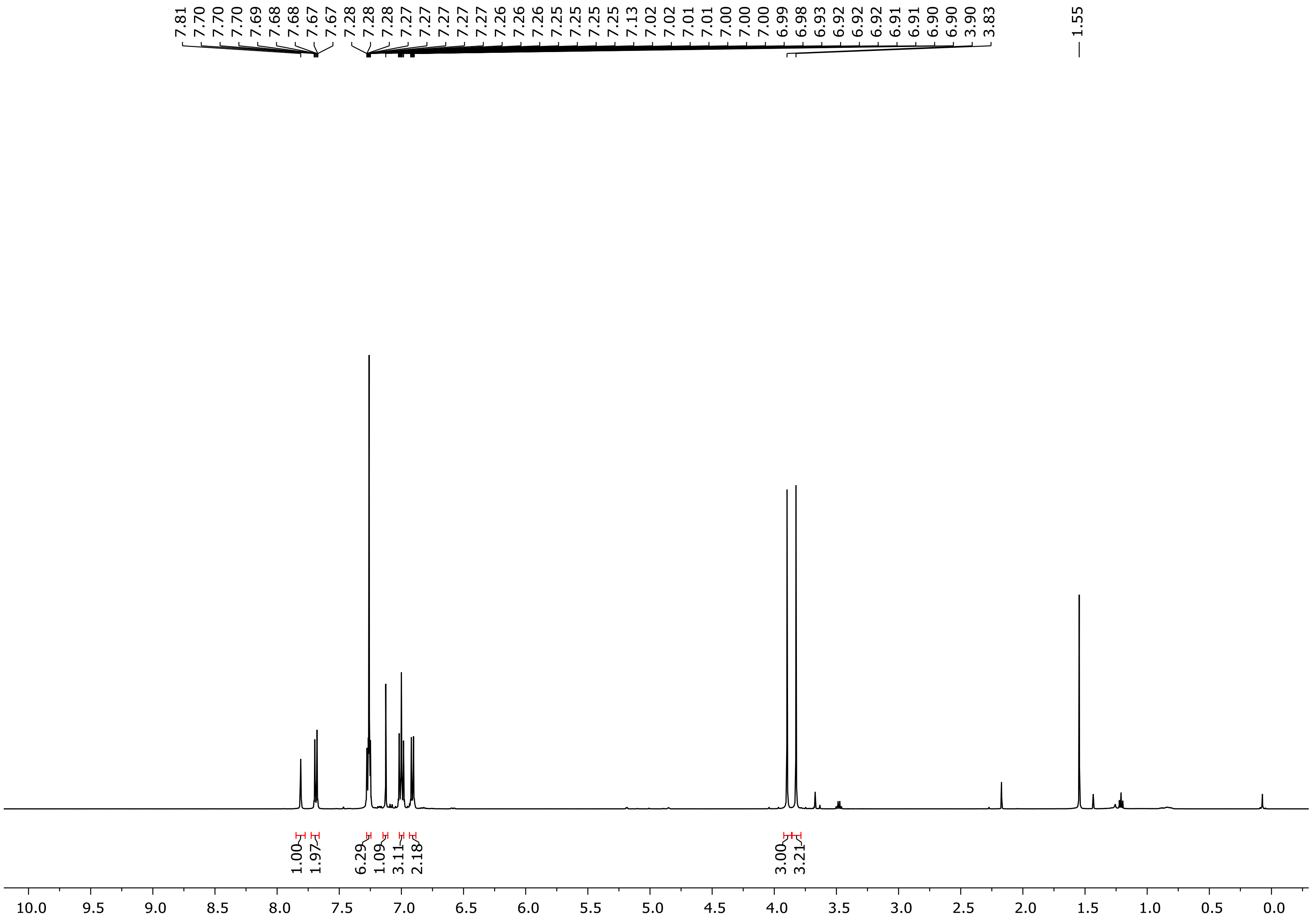

Figure S46: **$^{13}$C NMR** (126 MHz, $CDCl_3$, 298 K) spectrum of **3g**.

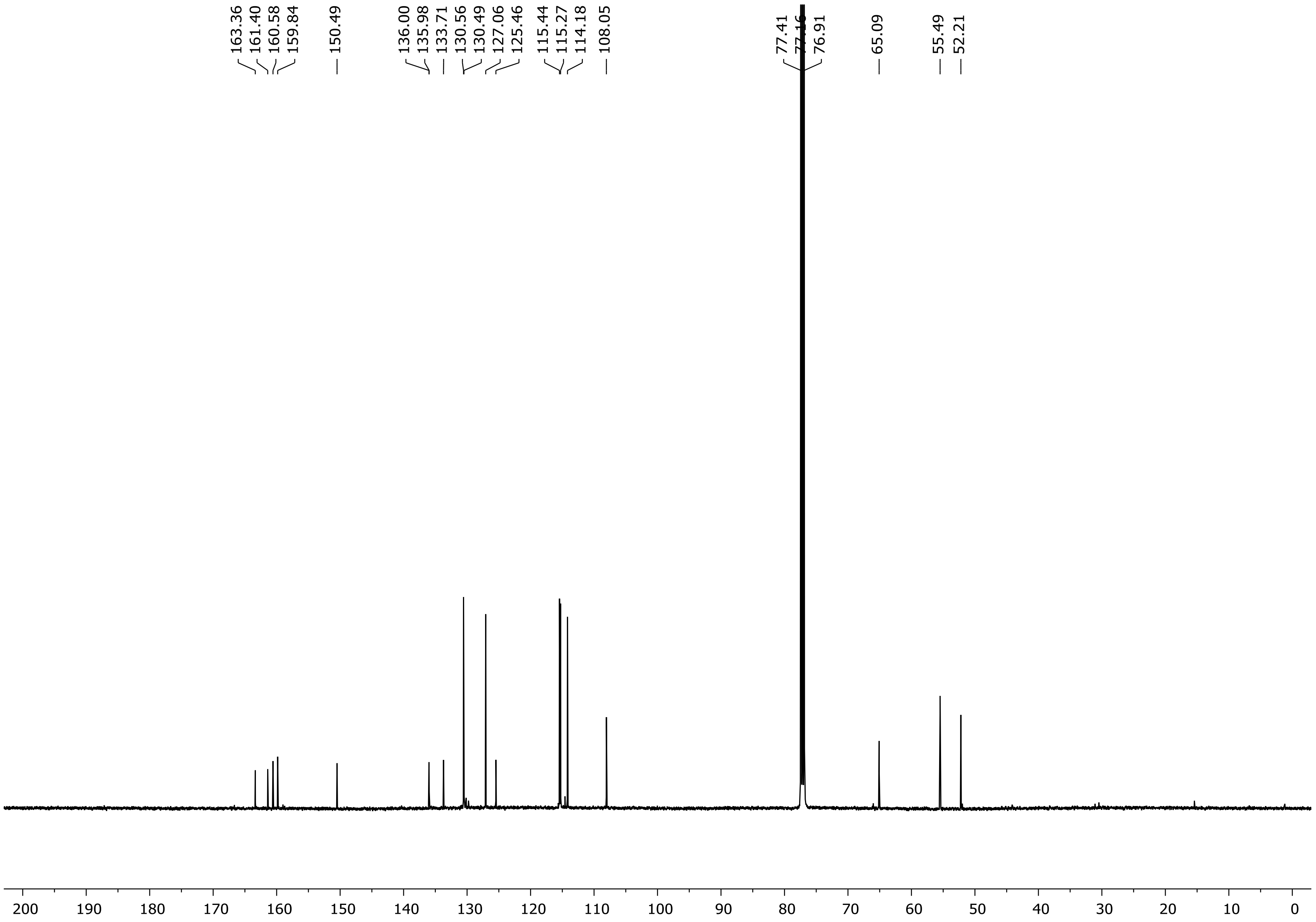

Figure S47: **$^{19}$F NMR** (471 MHz, $CDCl_3$, 298 K) spectrum of **3g**.

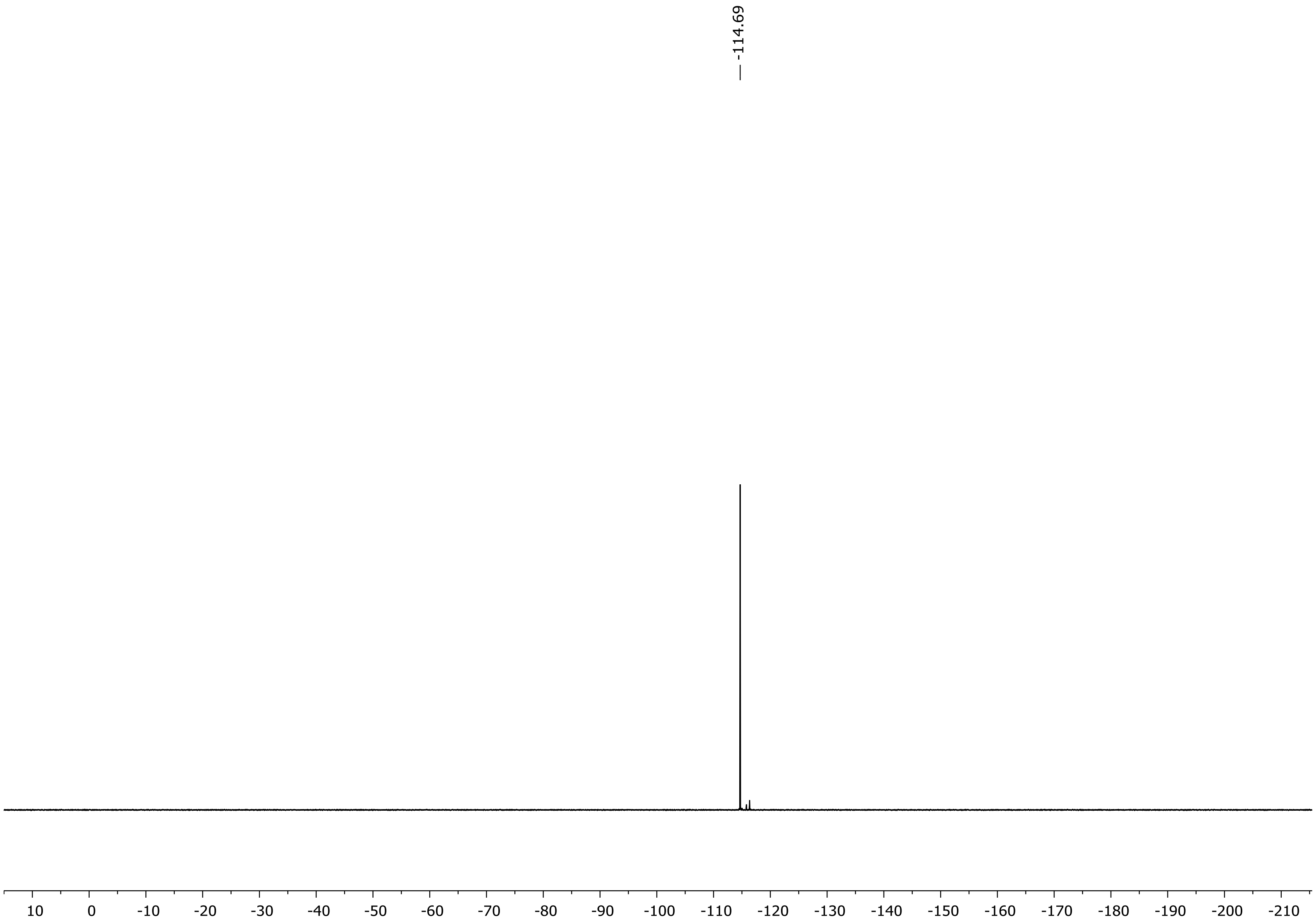

Figure S48: **$^1$H NMR** (500 MHz, $CDCl_3$, 298 K) spectrum of **3h**.

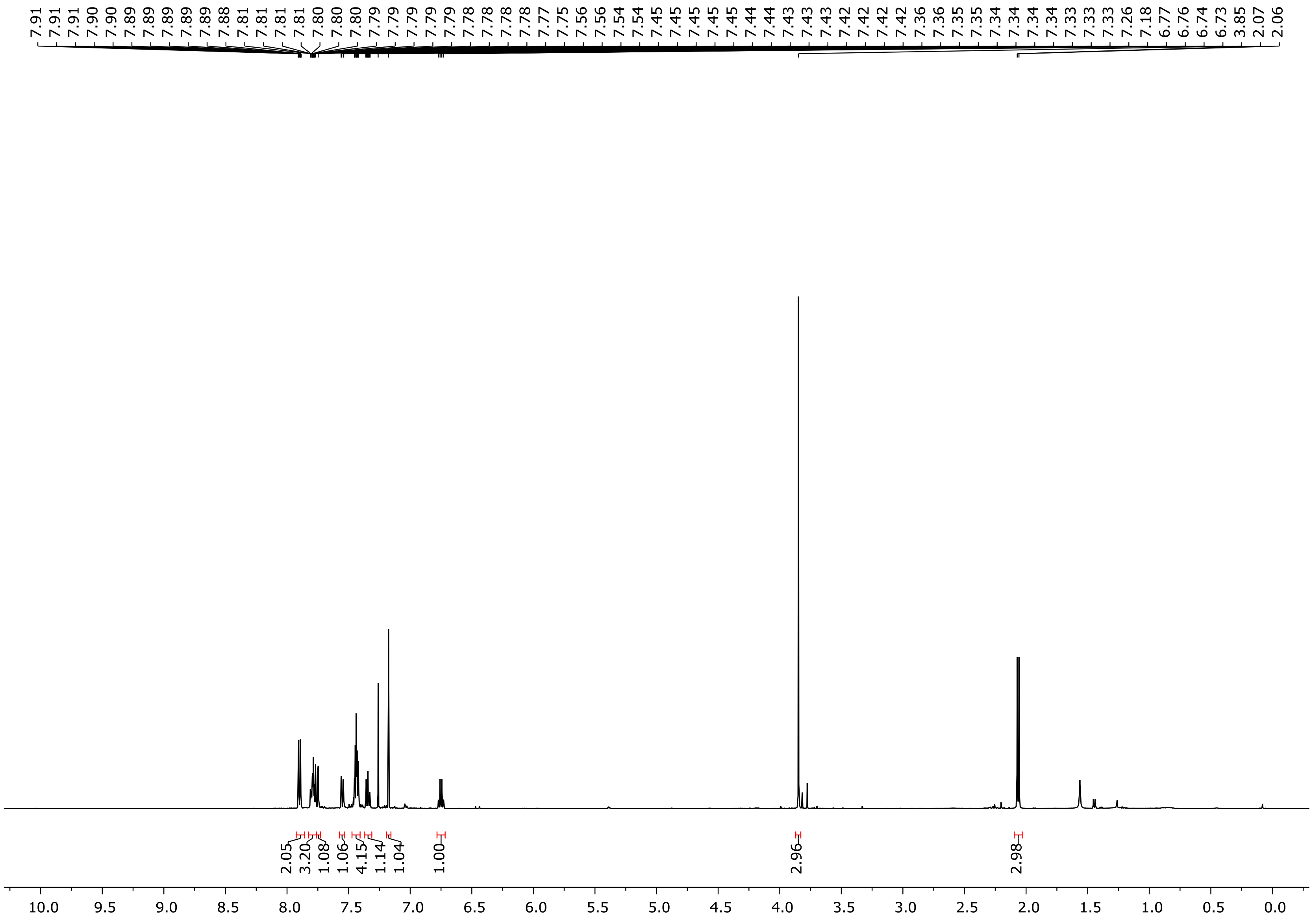

Figure S49: $^{13}$**C NMR** (126 MHz, $CDCl_3$, 298 K) spectrum of **3h**.

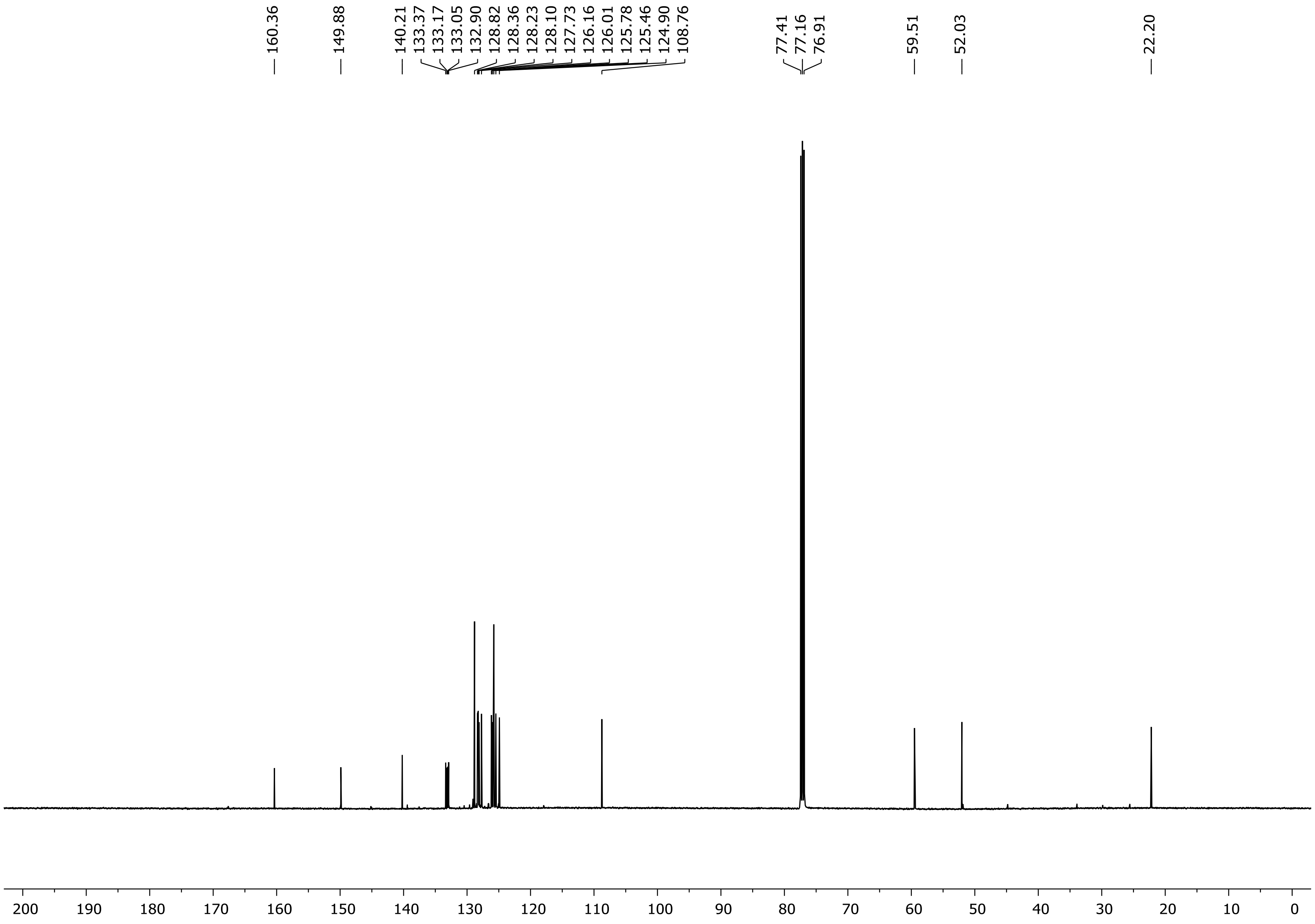

Figure S50: **$^1$H NMR** (500 MHz, $CDCl_3$, 298 K) spectrum of **3i**.

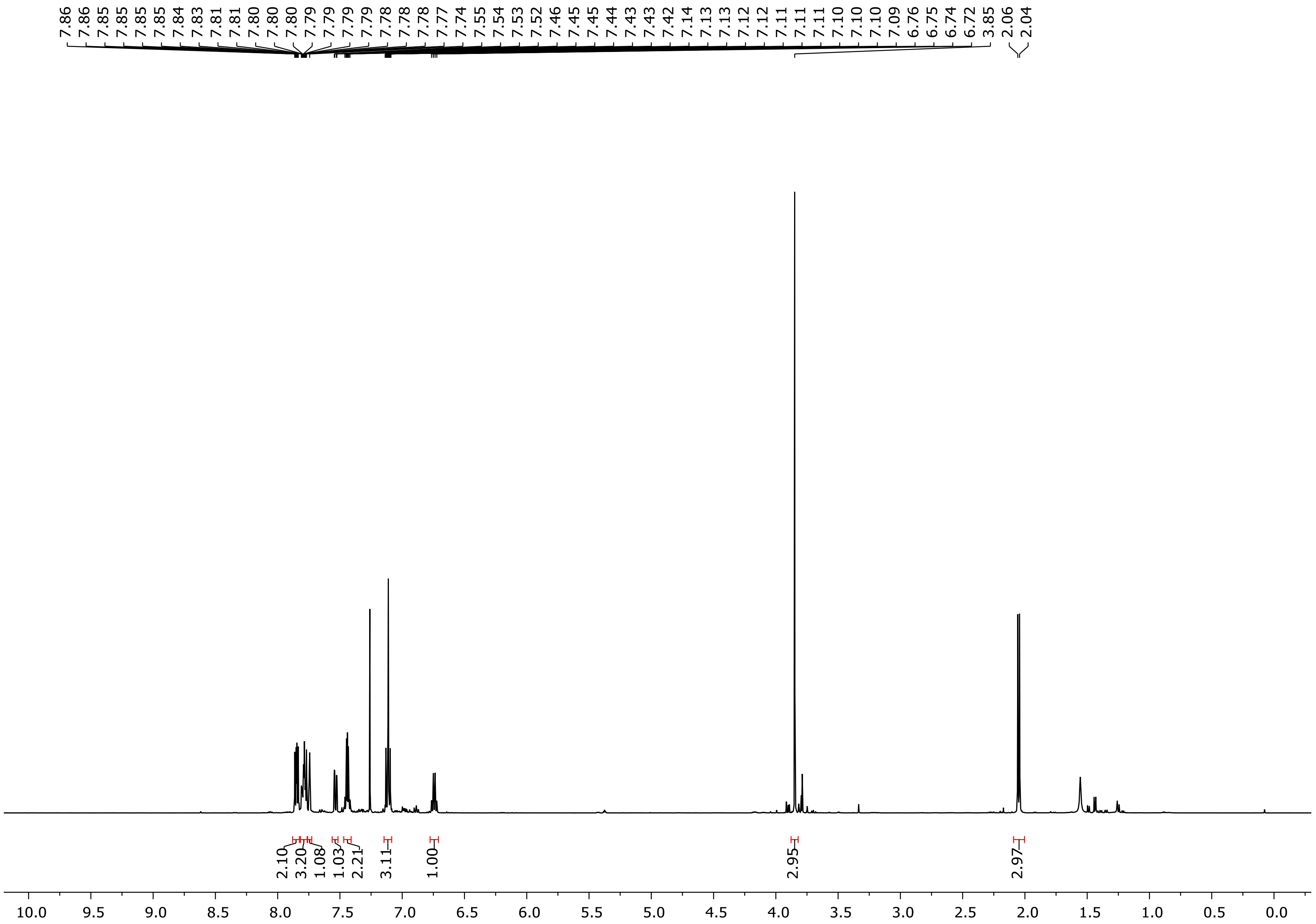

Figure S51: $^{13}$**C NMR** (126 MHz, $CDCl_3$, 298 K) spectrum of **3i**.

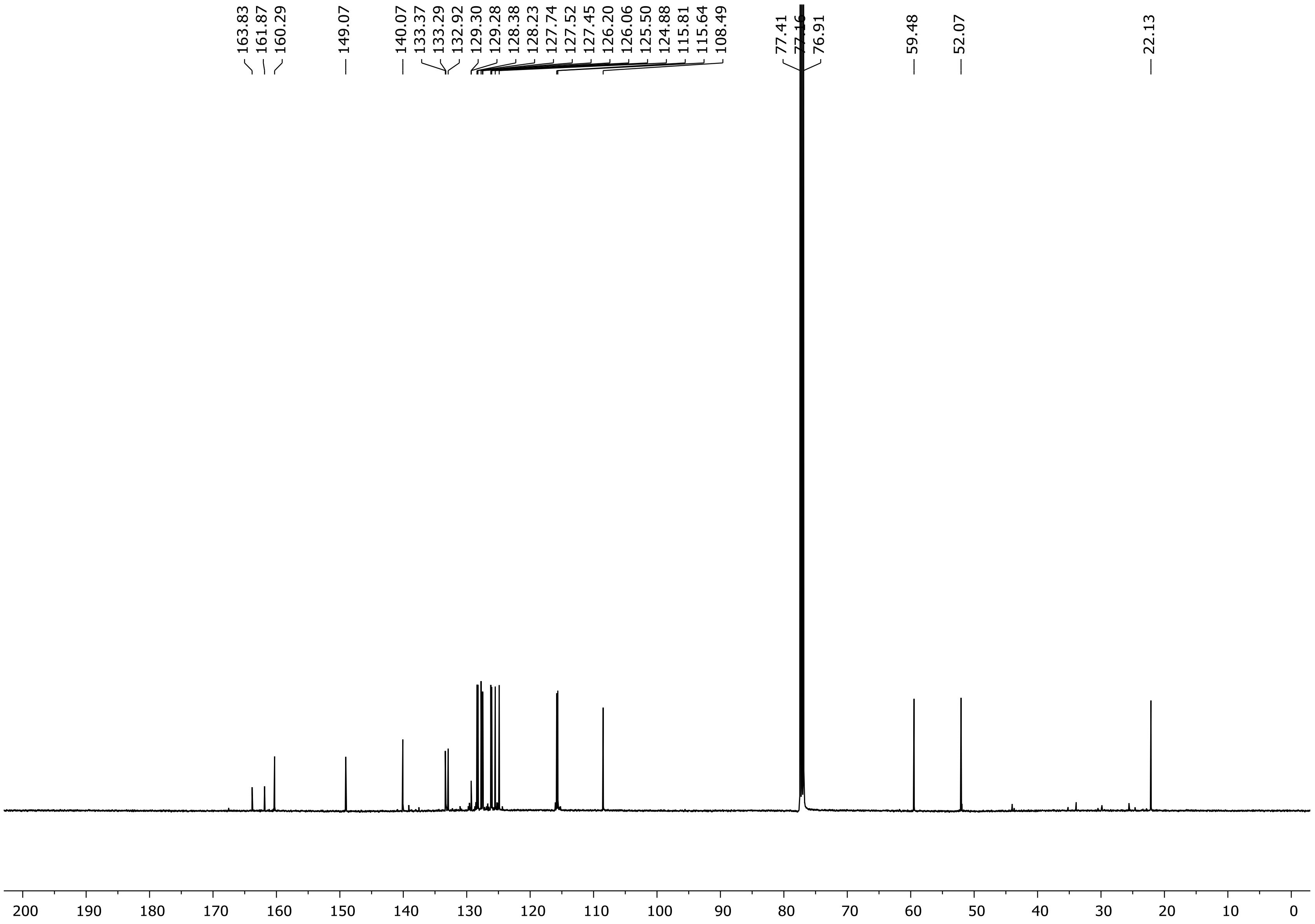

Figure S52: **$^{19}$F NMR** (471 MHz, $CDCl_3$, 298 K) spectrum of **3i**.

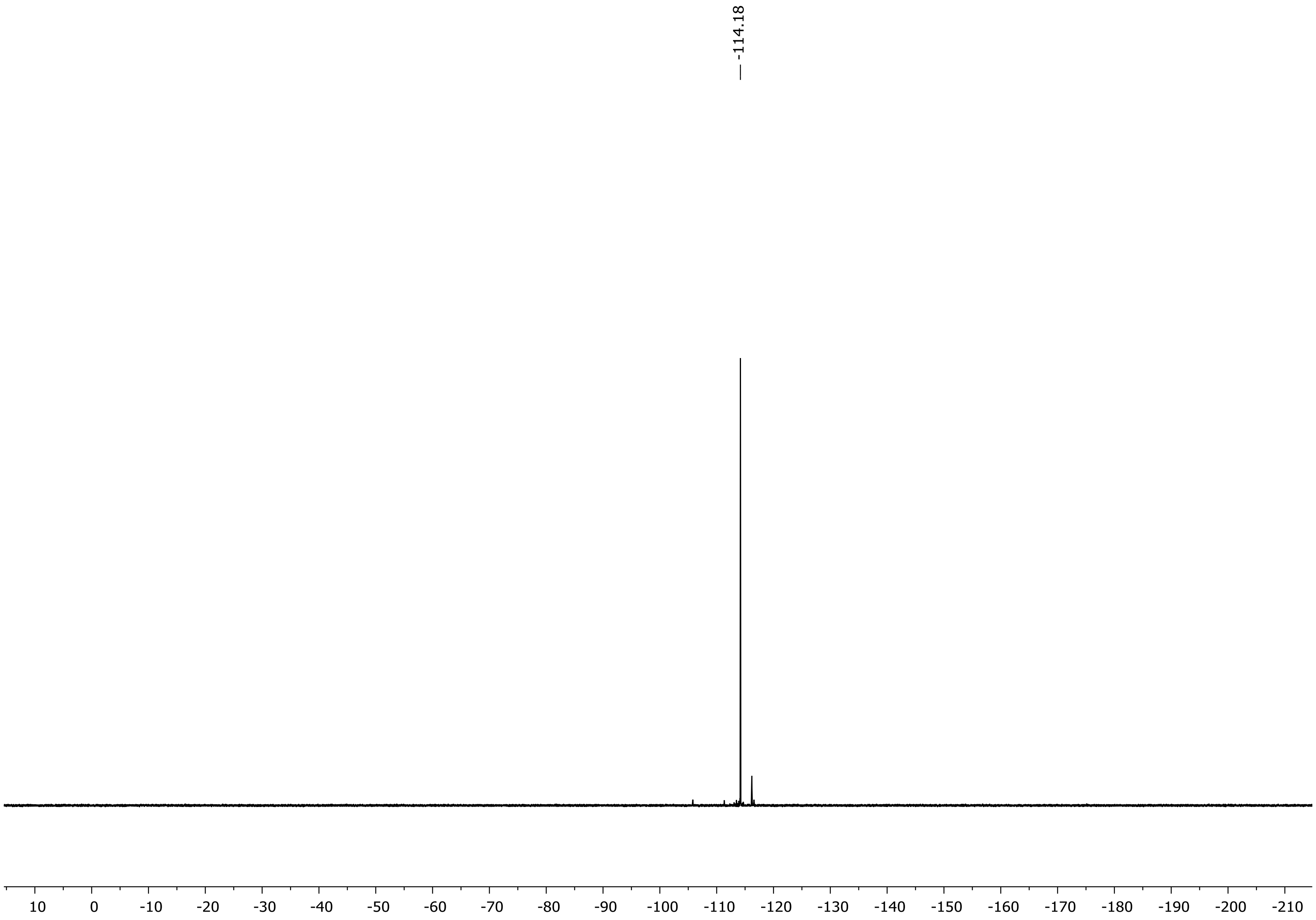

Figure S53: $^{1}$**H NMR** (500 MHz, $CDCl_3$, 298 K) spectrum of **3j**.

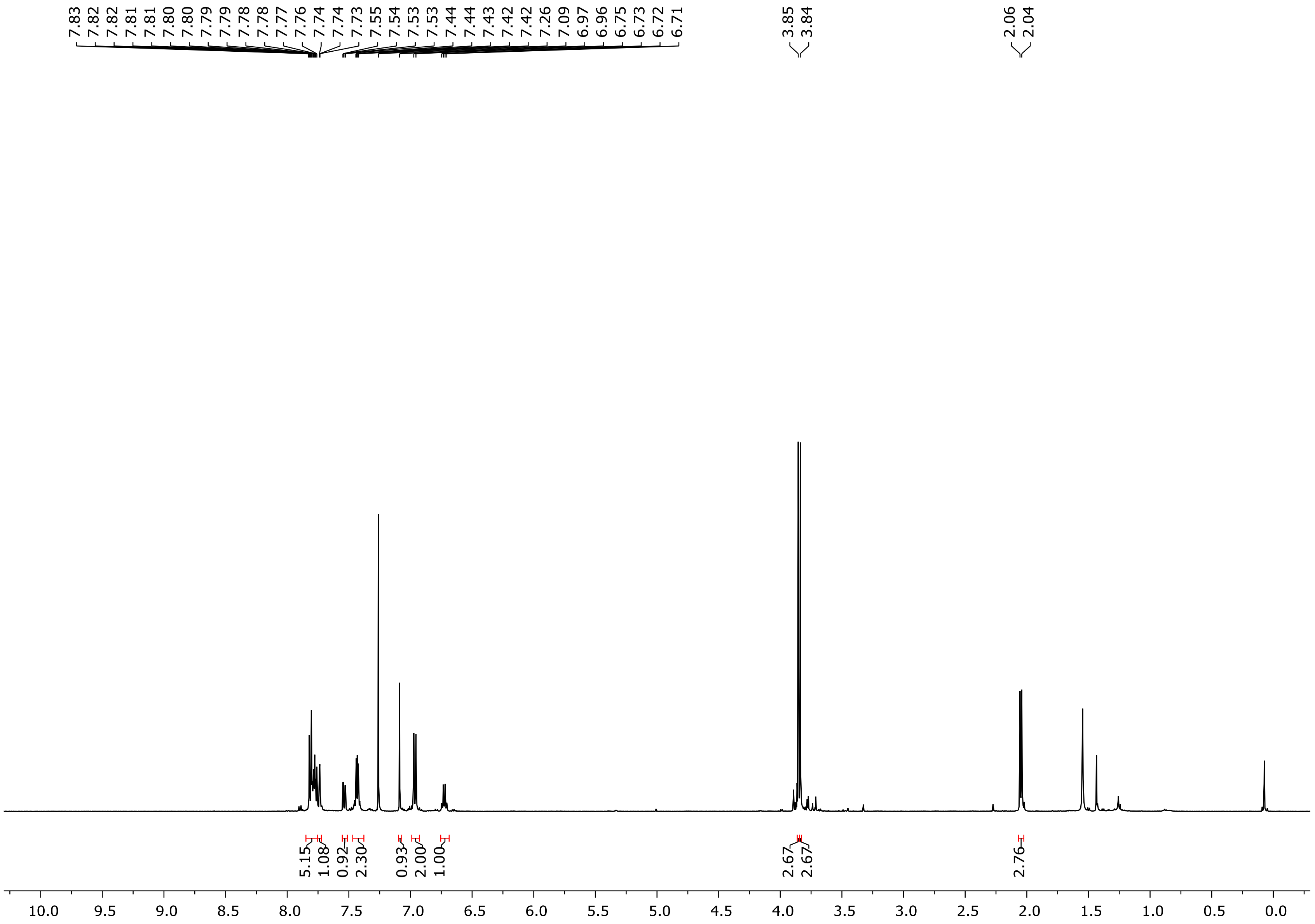

Figure S54: **$^{13}$C NMR** (126 MHz, $CDCl_3$, 298 K) spectrum of **3j**.

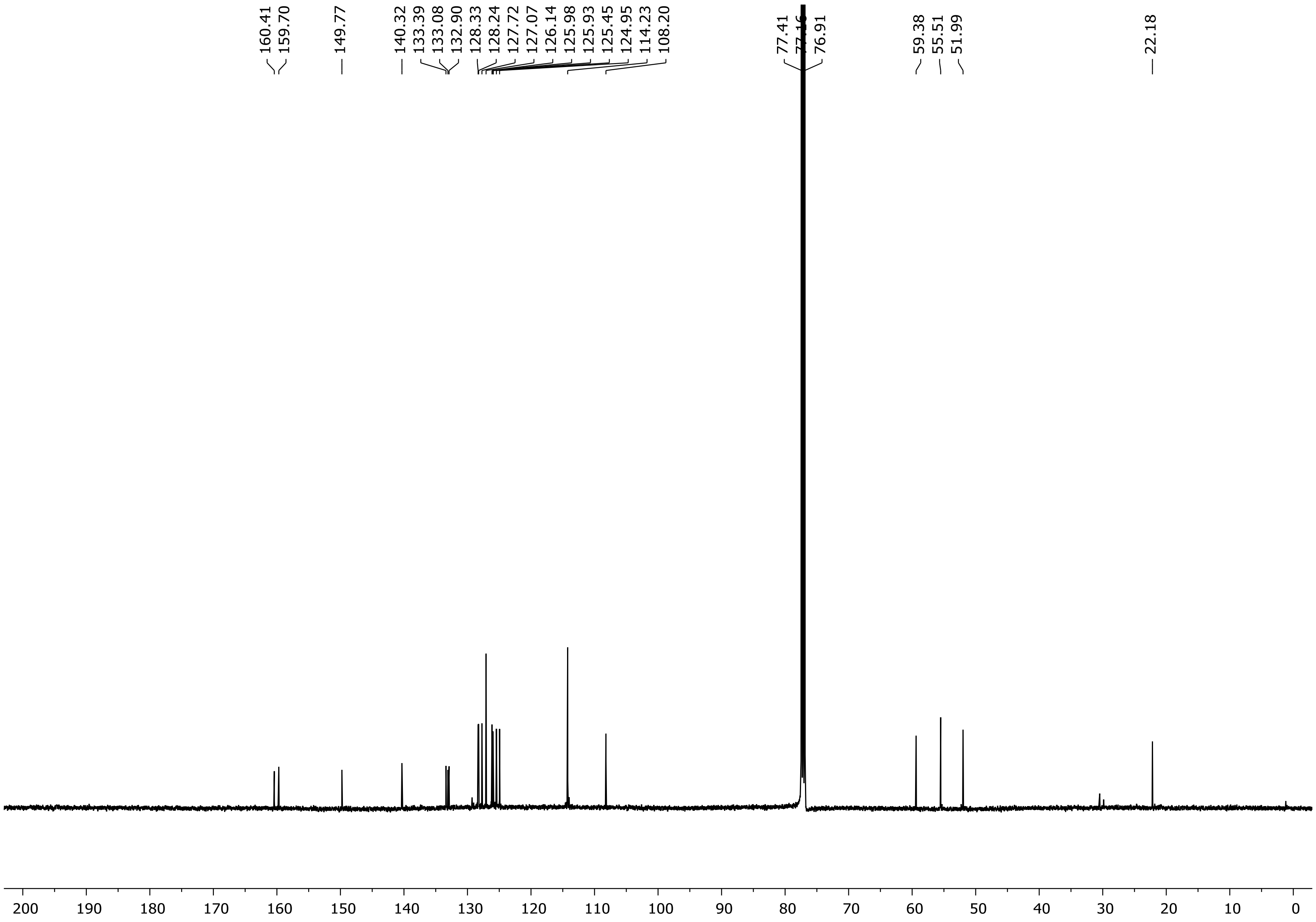

Figure S55: **¹H NMR** (600 MHz, $CDCl_3$, 298 K) spectrum of **10d**.

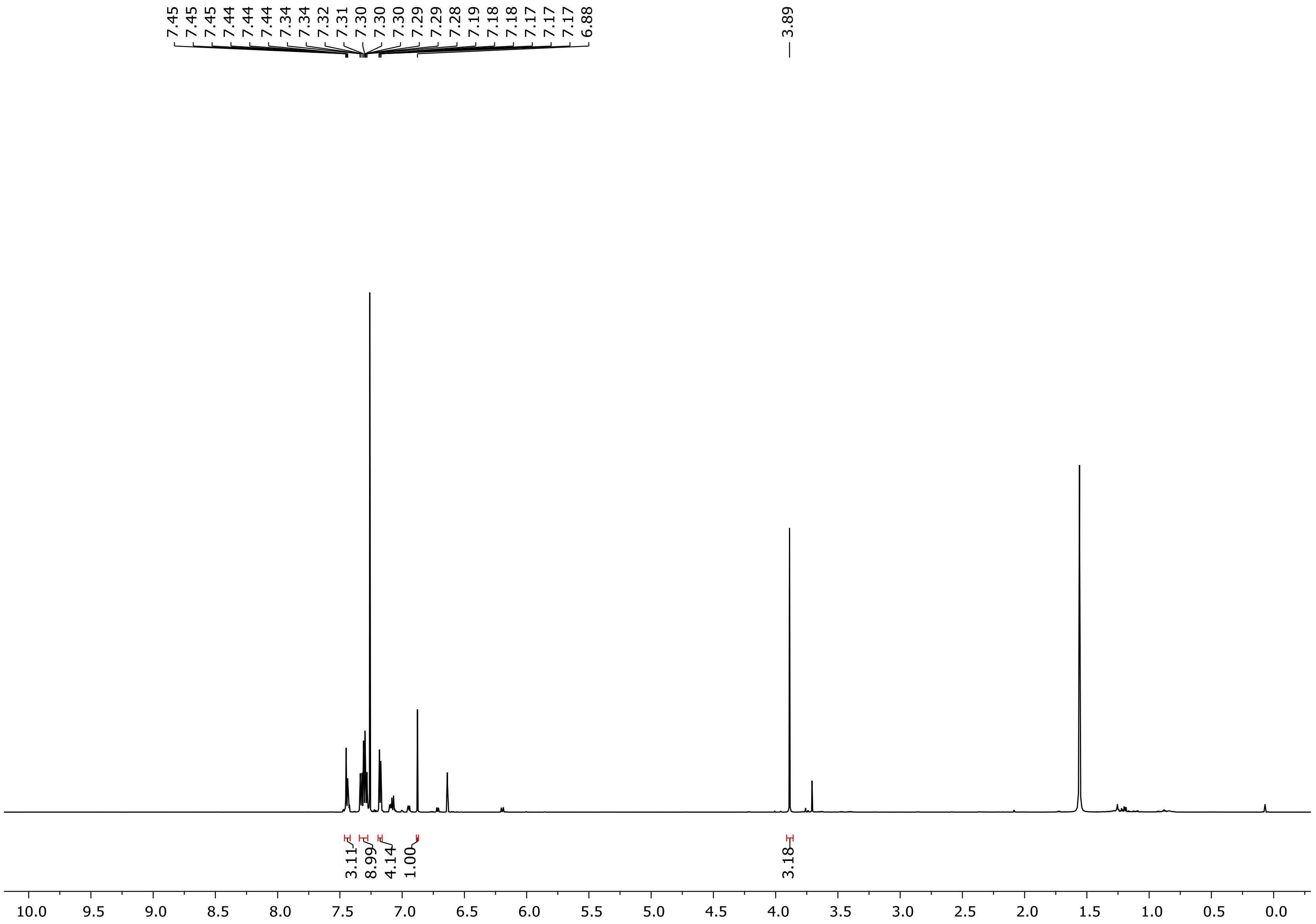

Figure S56: **$^{13}$C NMR** (151 MHz, $CDCl_3$, 298 K) spectrum of **10d**.

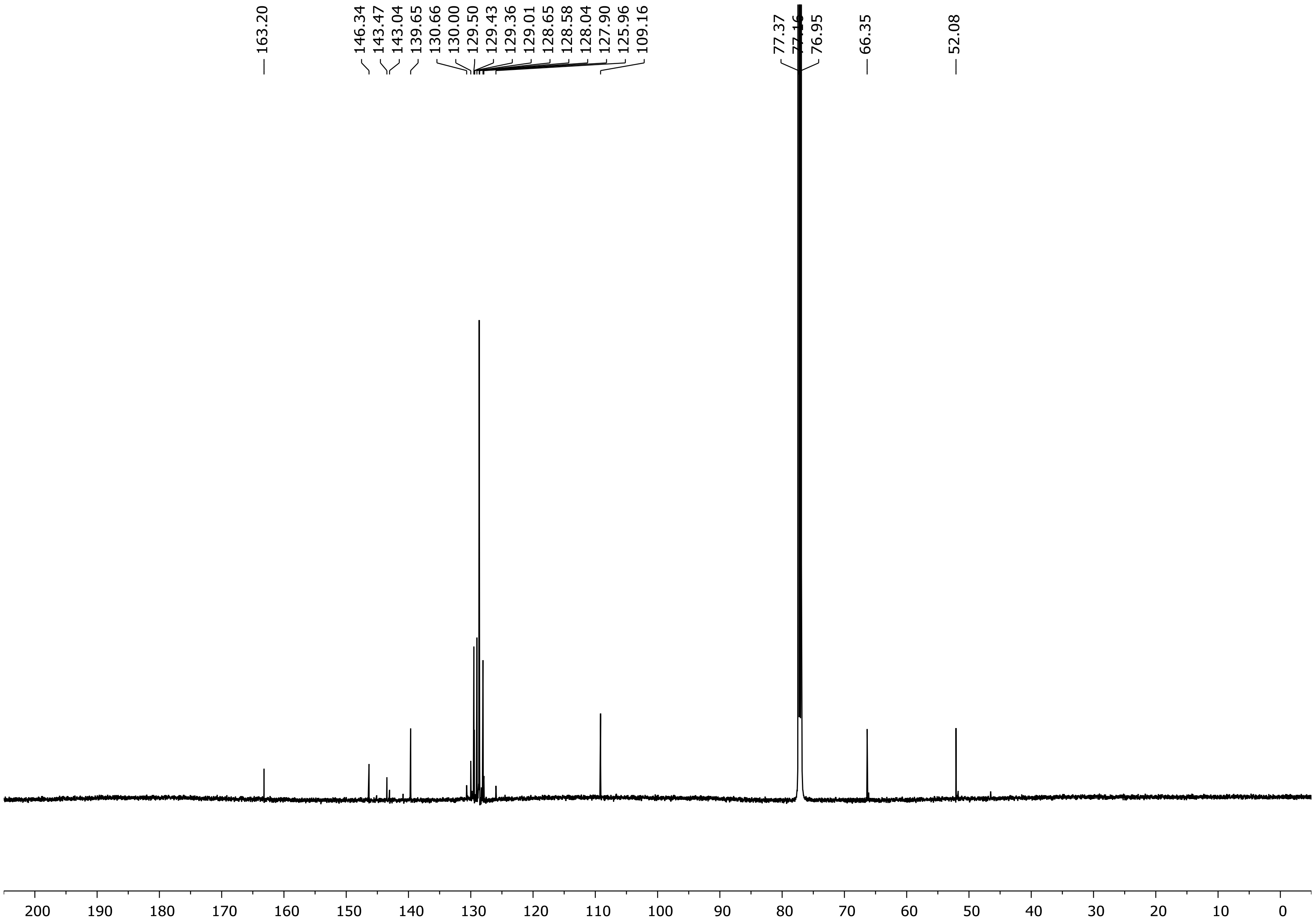

Figure S57: **$^{1}$H NMR** (500 MHz, $CDCl_3$, 298 K) spectrum of a reaction between stoichiometric **1b** and $B(C_6F_5)_3$.

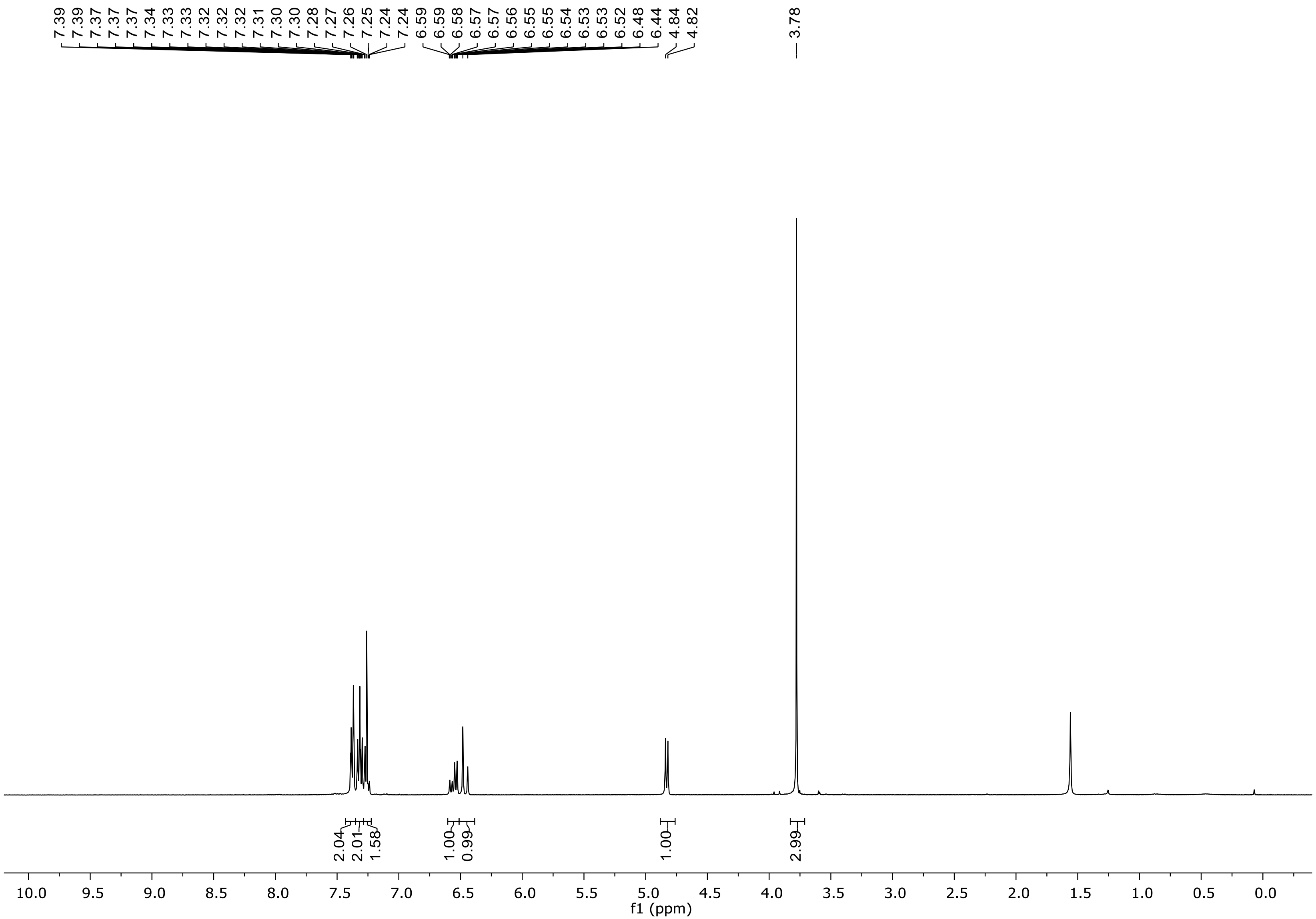

Figure S58: **$^{13}$C NMR** (126 MHz, $CDCl_3$, 298 K) spectrum of a reaction between stoichiometric **1b** and $B(C_6F_5)_3$.

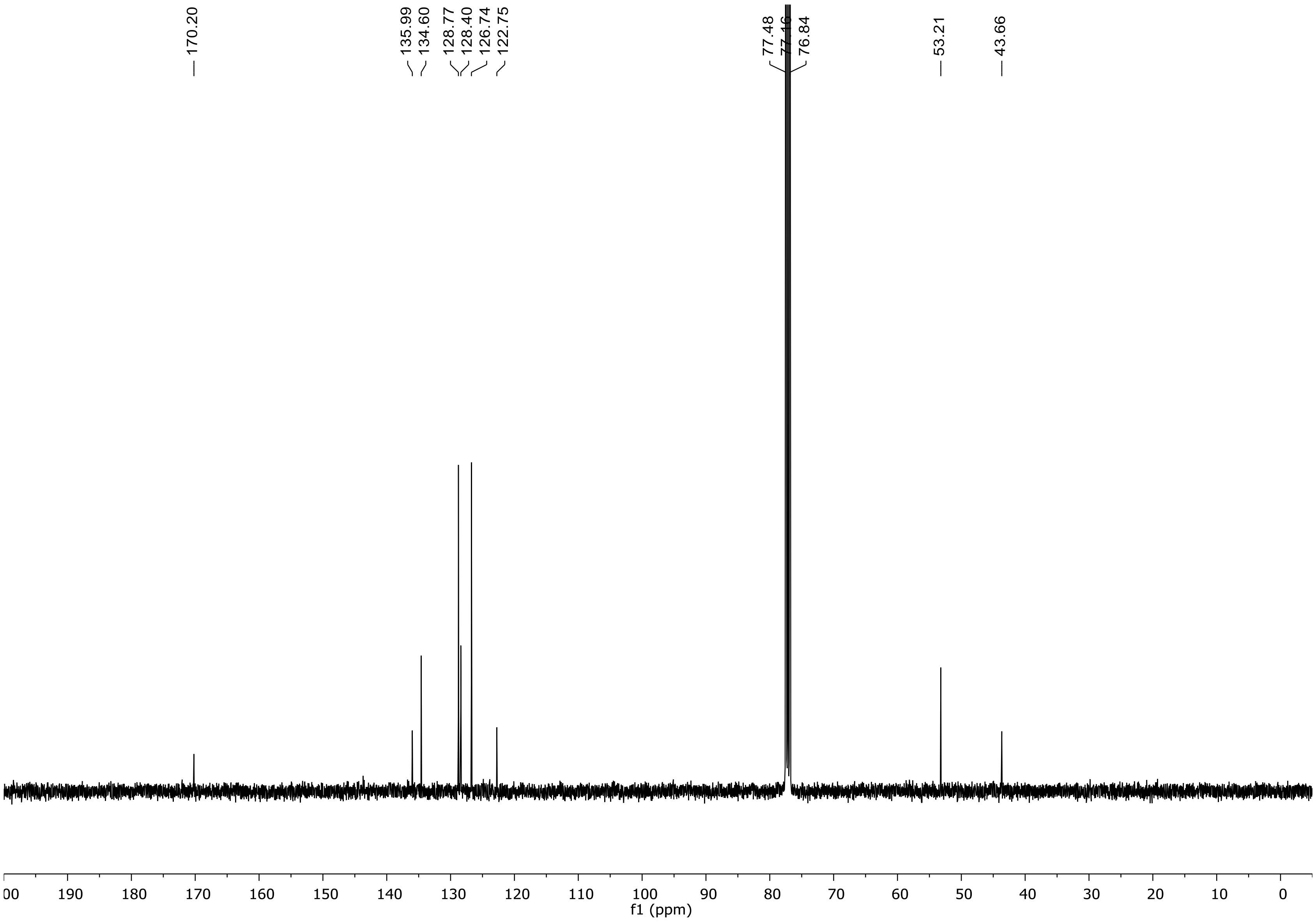

Figure S59: **$^{19}$F NMR** (471 MHz, $CDCl_3$, 298 K) spectrum of a stoichiometric reaction between **1b** and $B(C_6F_5)_3$.

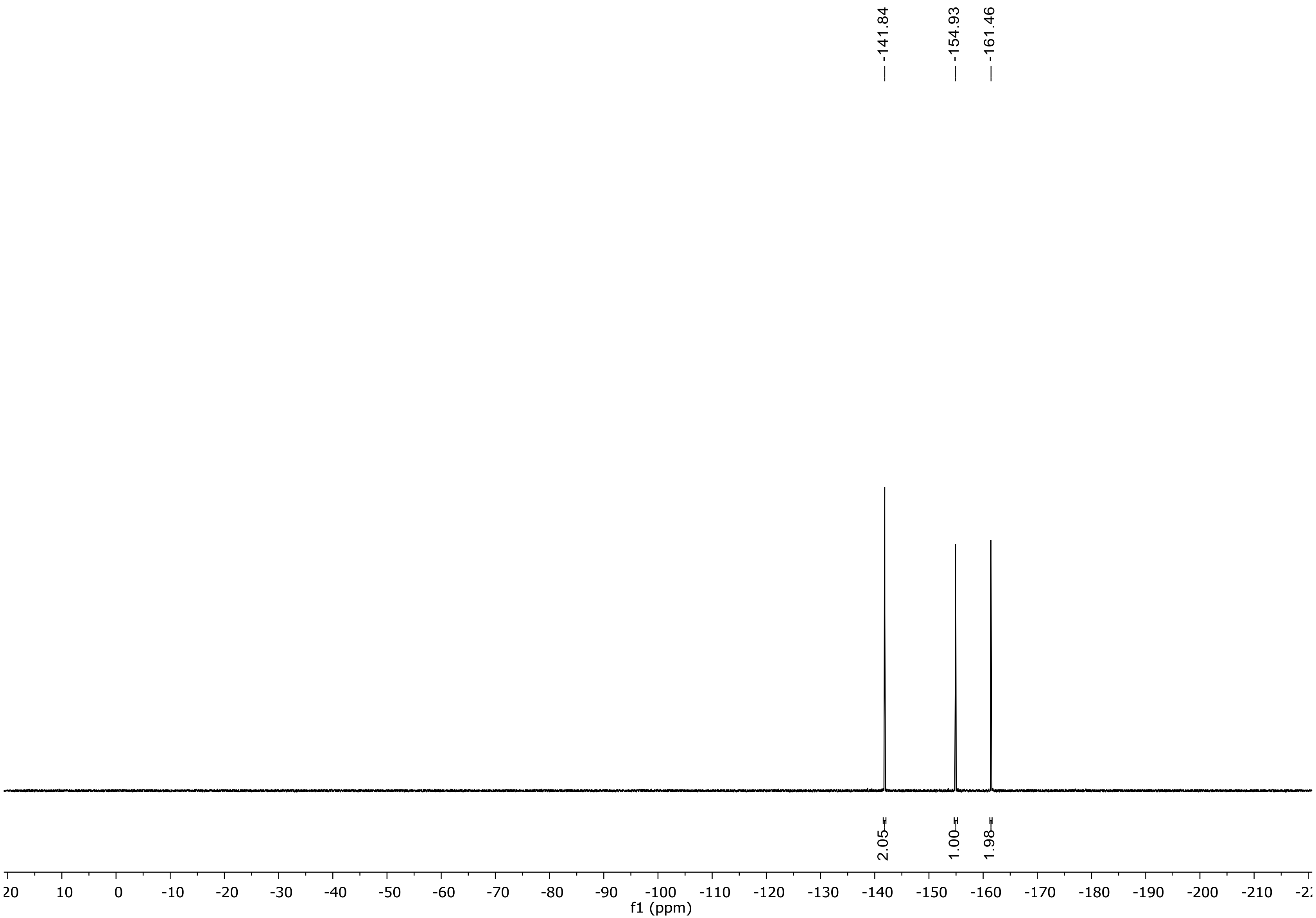

Figure S60: Crude **¹H NMR** (500 MHz, $CDCl_3$, 298 K) spectrum of **3a**.

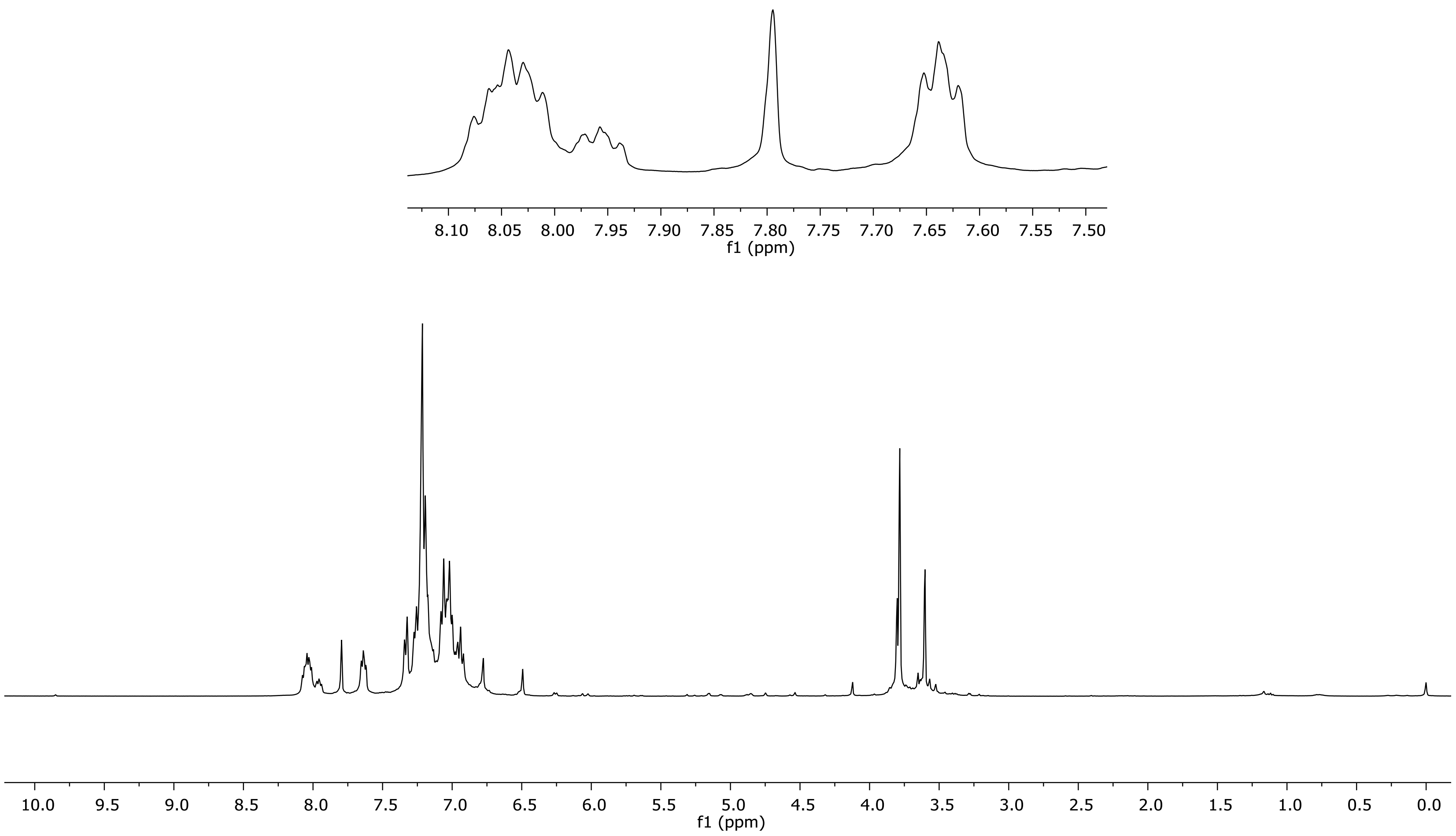

Figure S61: Crude **$^{1}$H NMR** (500 MHz, $CDCl_3$, 298 K) spectrum of **3d**.

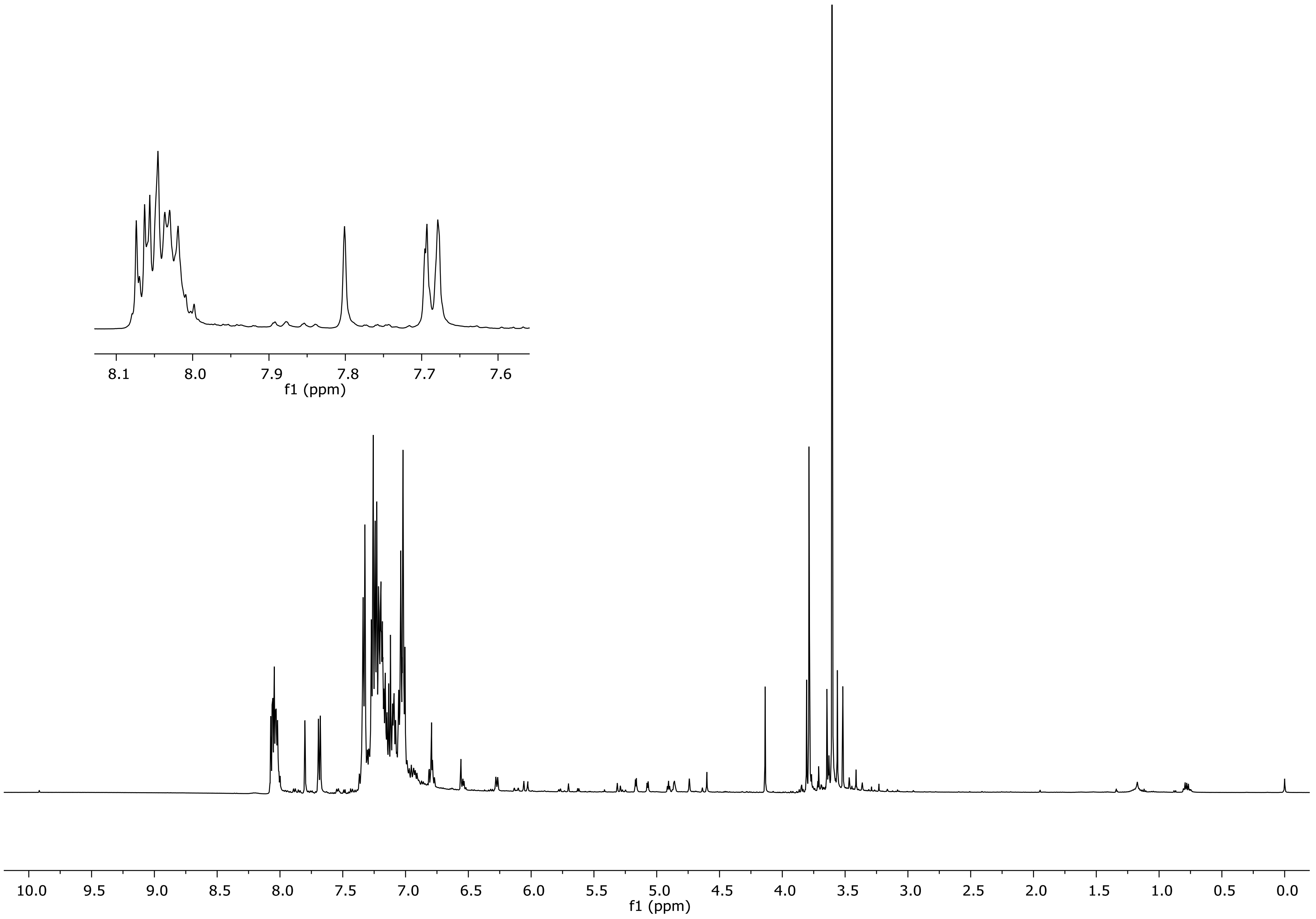

Figure S62: Crude **$^{1}$H NMR** (500 MHz, $CDCl_3$, 298 K) spectrum of **3e**.

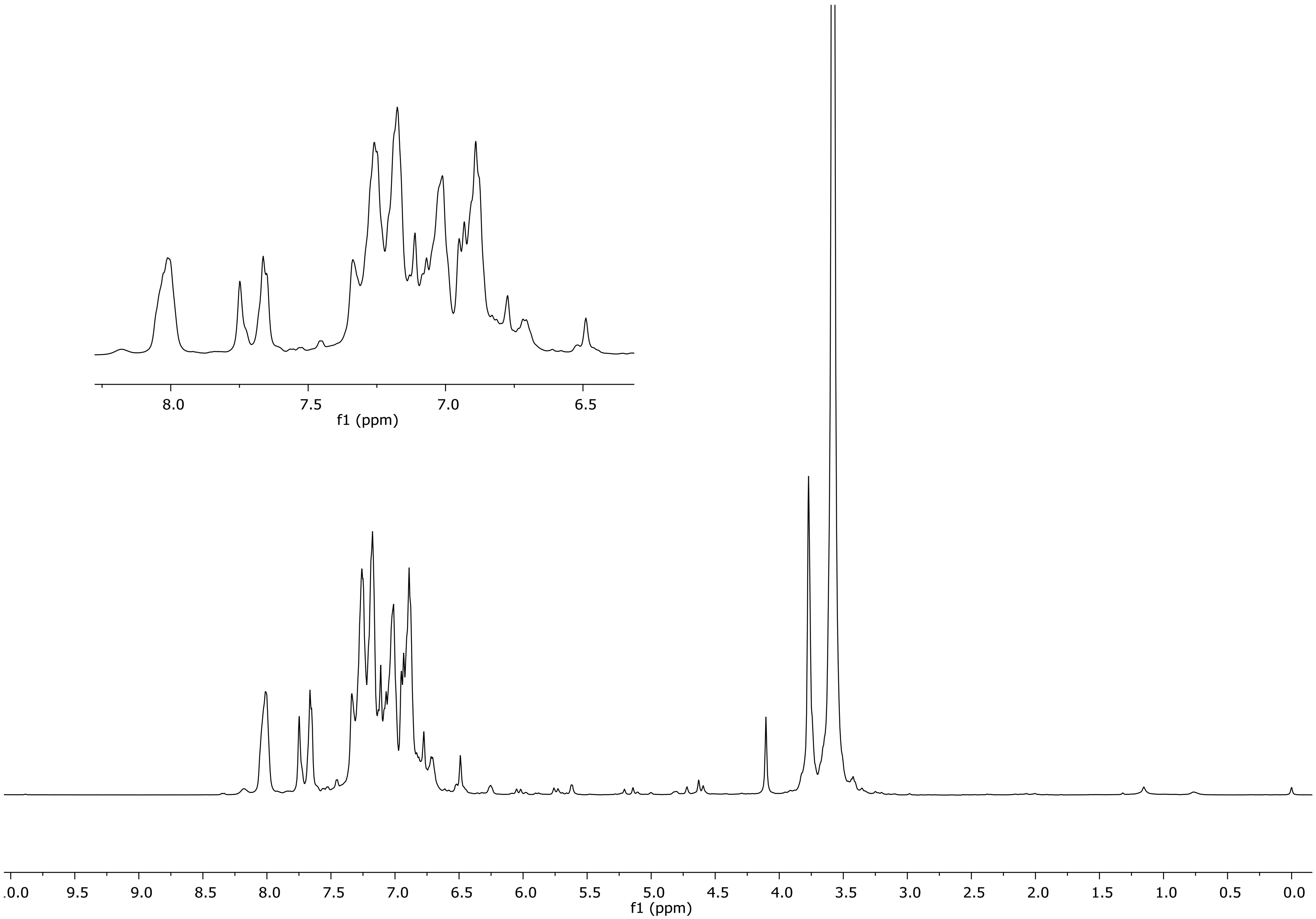

Figure S63: Crude **$^1$H NMR** (500 MHz, $CDCl_3$, 298 K) spectrum of **3g**.

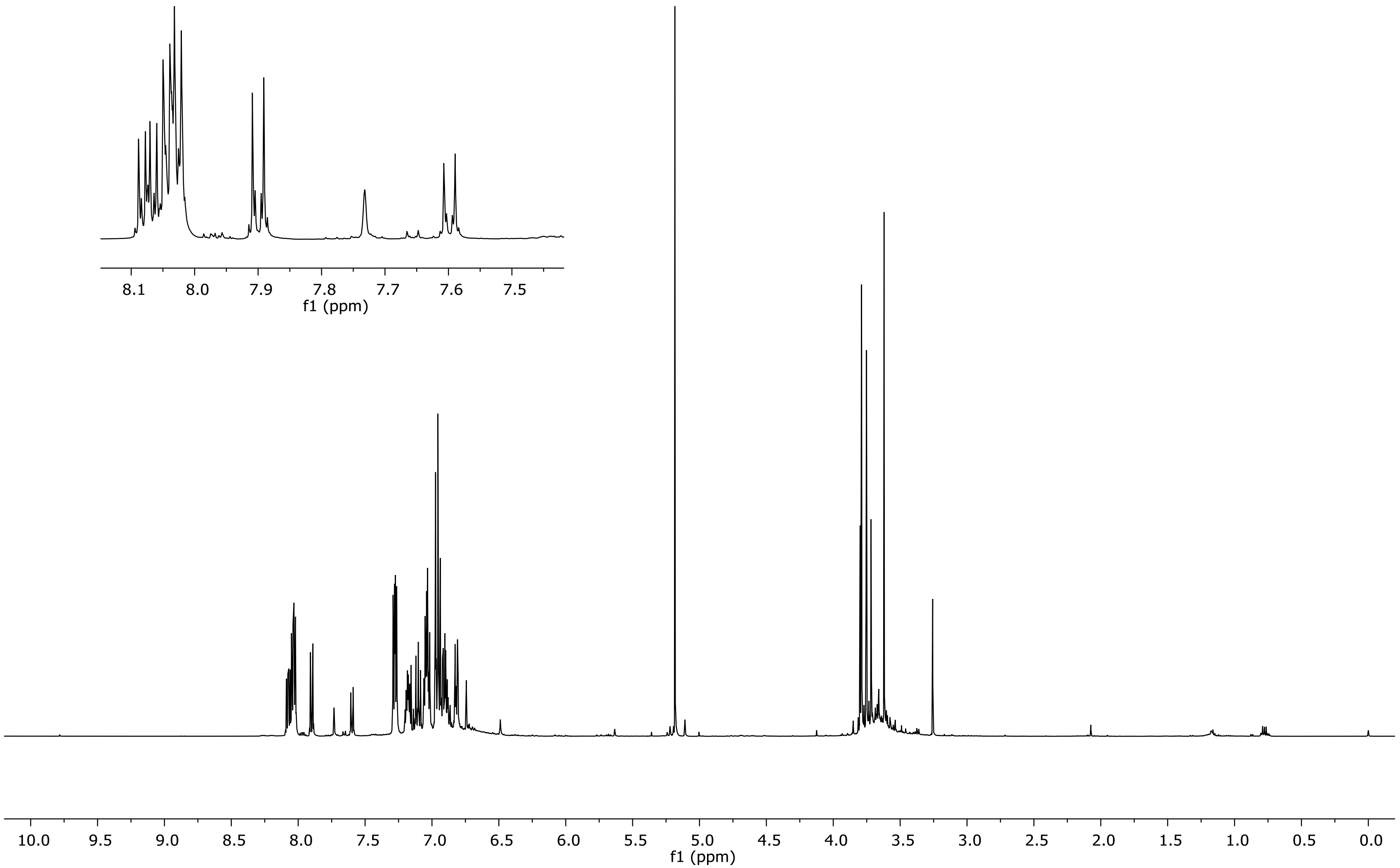

Figure S64: **$^{11}$B NMR** (128 MHz, $CDCl_3$, 298 K) spectrum of a stoichiometric reaction between **2a** and $B(C_6F_5)_3$.

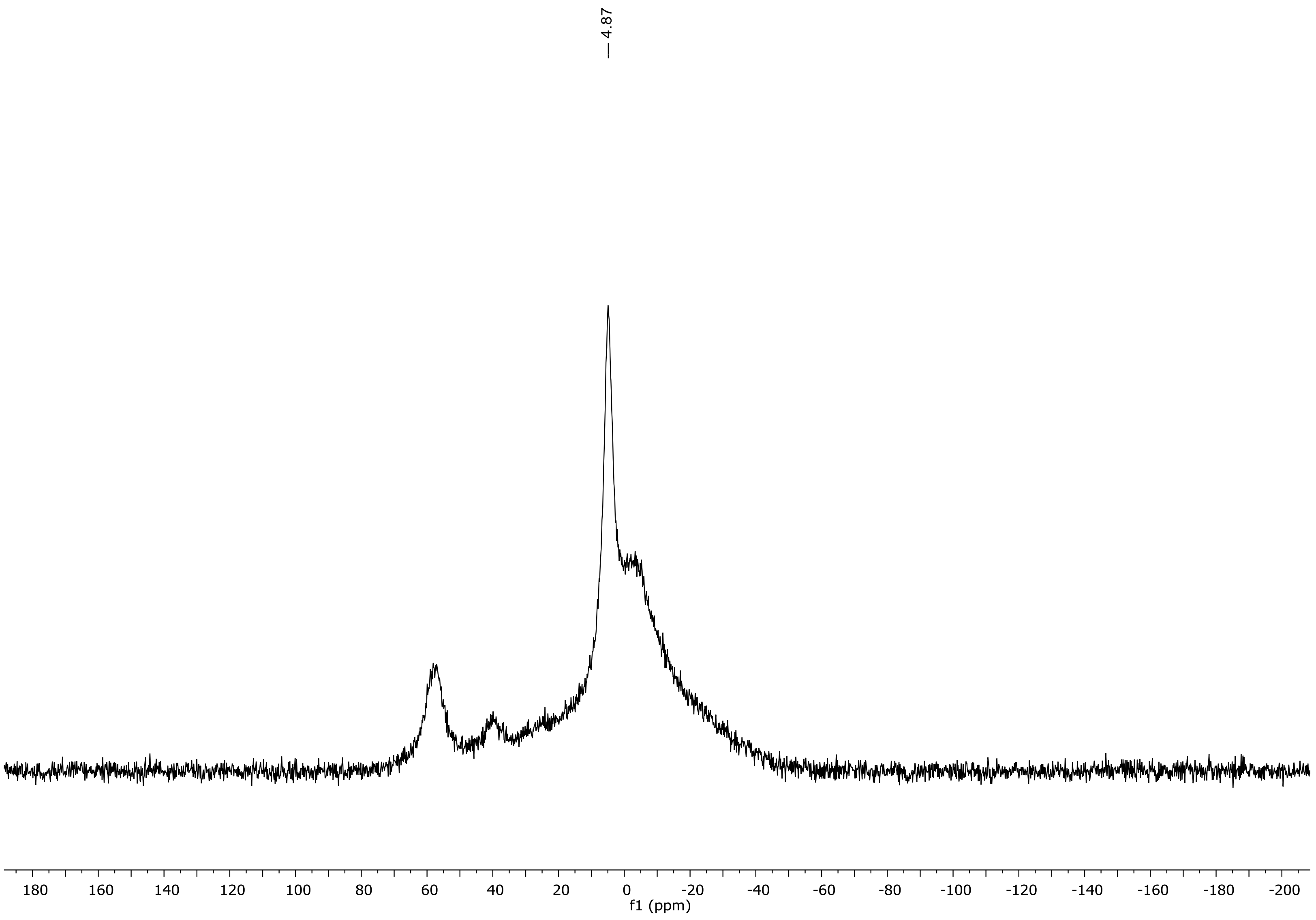

Figure S65: Crude **$^1$H NMR** (500 MHz, $CDCl_3$, 298 K) spectrum of reaction between **2a** and **3e** using 10 mol% $B(C_6F_5)_3$.

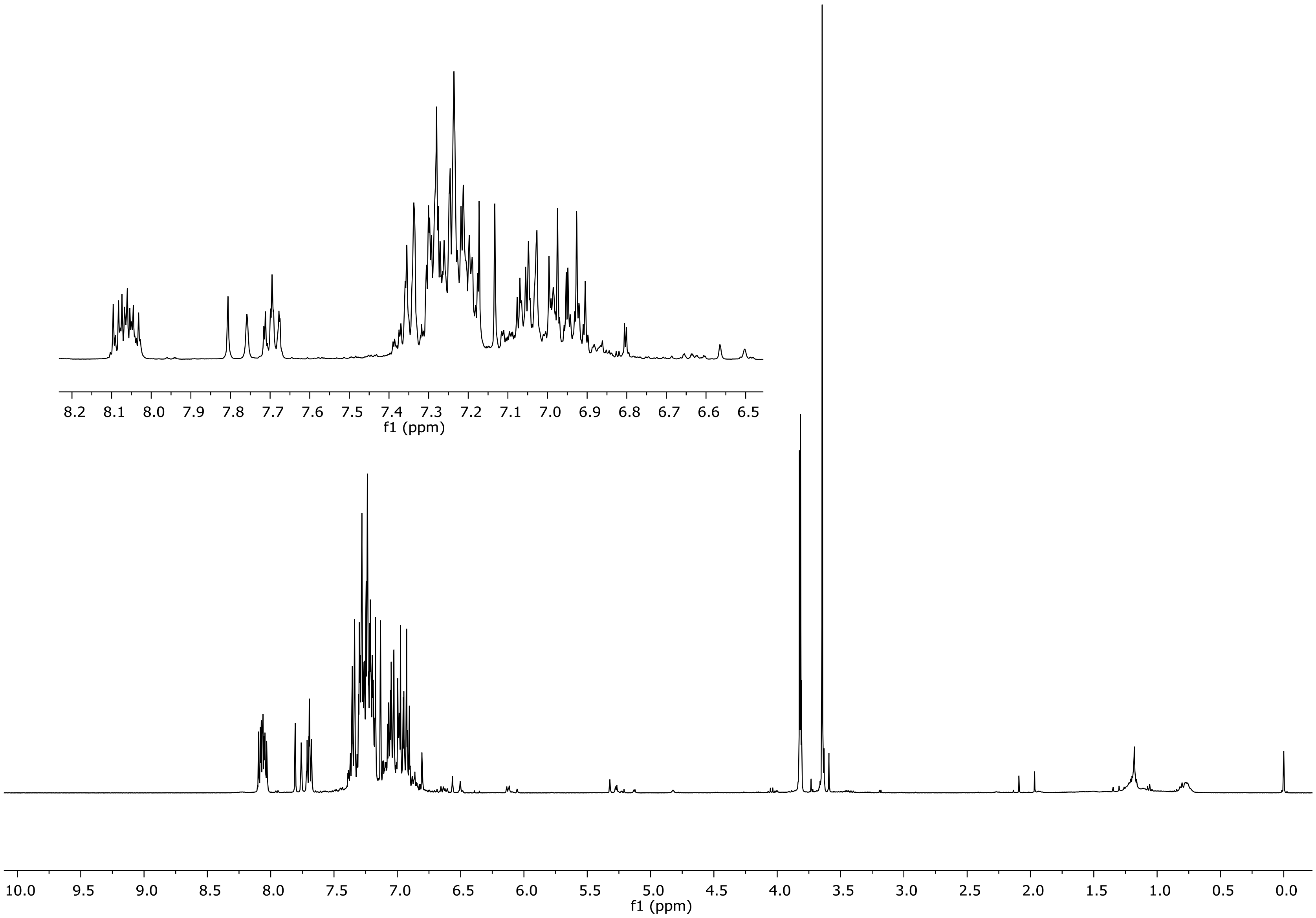

# 4. Crystallographic Data

## 4.1 Single crystal X-ray diffraction experimental:

Single crystals of **3a** and **3d** were grown in a fume hood by slow evaporation or vapor diffusion. Crystallographic studies were undertaken on single crystal mounted in paratone and studied on an Agilent SuperNova Dual Atlas three-circle diffractometer using Mo- or Cu-Kα radiation and a CCD detector. Measurements were taken at 206(9) K (**3a)** and 180(10) K (**3d**) with temperatures maintained using an Oxford cryostream. Data were collected and integrated and data corrected for absorption using a numerical absorption correction based on Gaussian integration over a multifaceted crystal model within CrysAlisPro.[5] The structure was solved by direct methods and refined against F2 within SHELXL-2013.[6] The structure has been deposited with the Cambridge Structural Database [CCDC deposition numbers **2068715** (**3a**) and **2095847** (**3d**)]. This can be obtained free of charge from the Cambridge Crystallographic Data Centre via www.ccdc.cam.ac.uk/data_request/cif

**4.2 Solid-state structure:**

Figure 66. Solid-state structure of compound **3a**, Thermal ellipsoids drawn at 50% probability. Carbon: black; oxygen: red; fluorine: light green; nitrogen: blue.

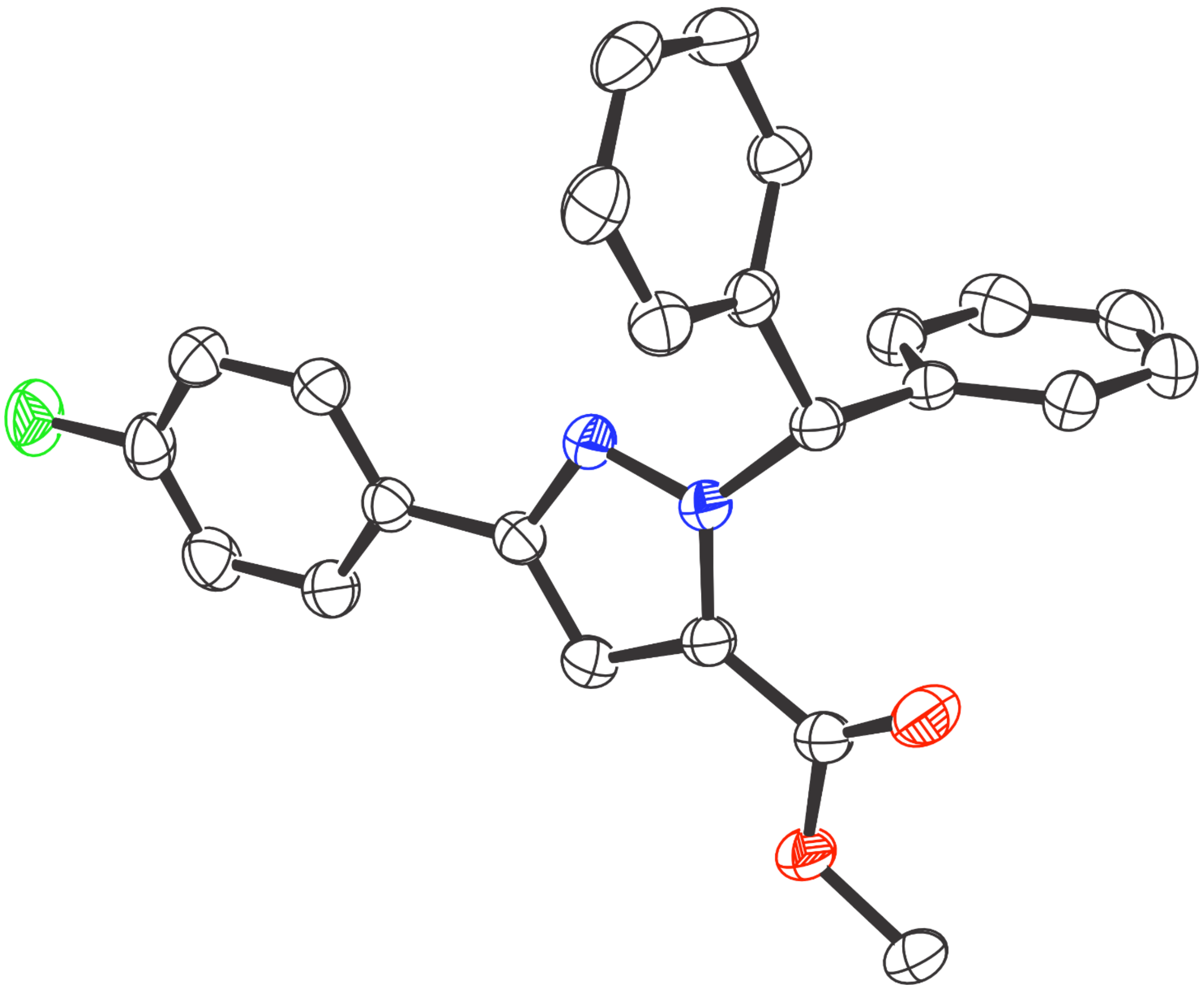

Figure 67. Solid-state structure of compound **3d**, Thermal ellipsoids drawn at 50% probability. Carbon: black; oxygen: red; nitrogen: blue.

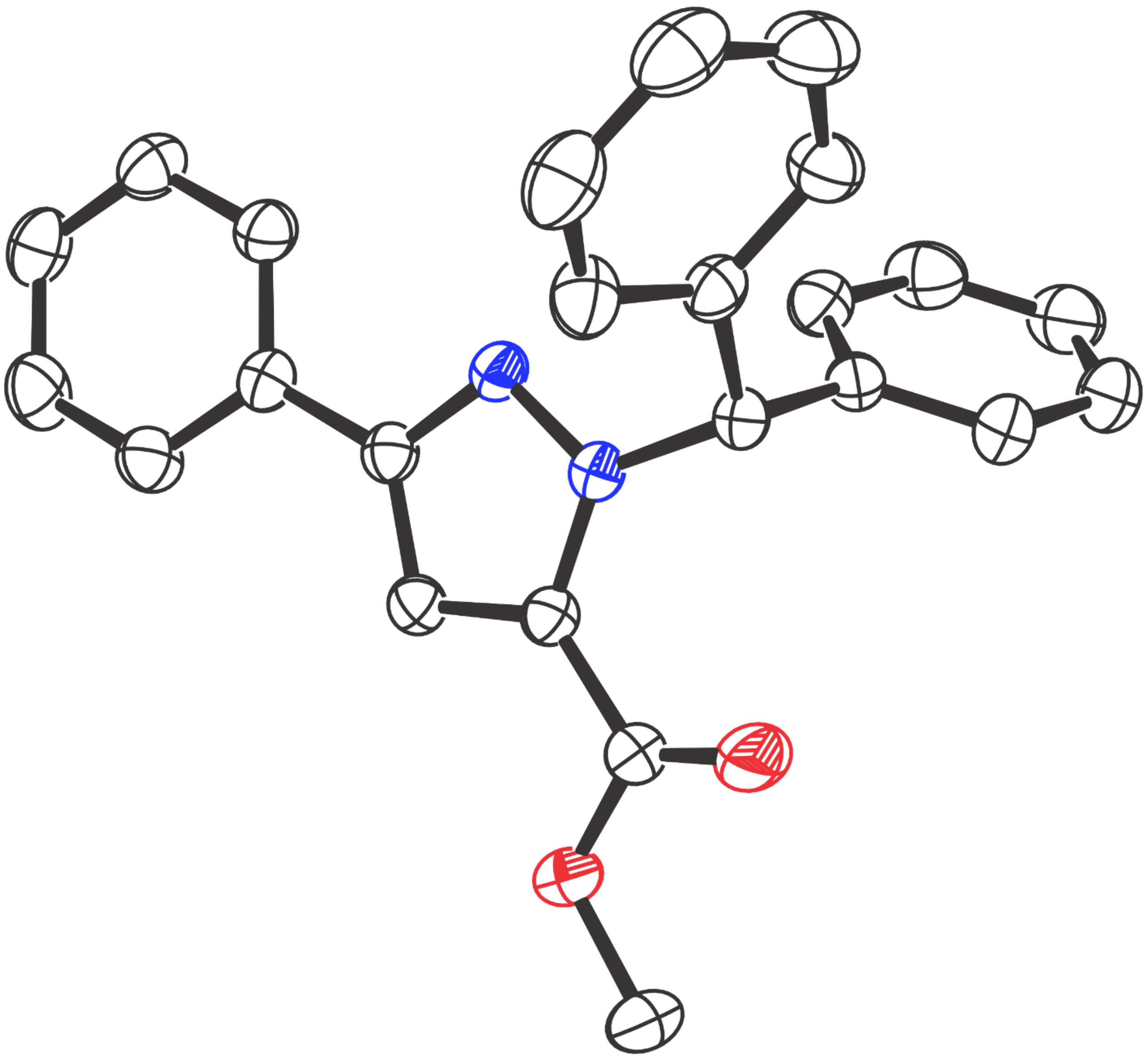

## 4.3 X-ray refinement data:

Table S1. Crystal data and structure refinement for compound **3a**. [CCDC: 2068715]

| | |
|---|---|
| Empirical formula | $C_{24}H_{19}FN_2O_2$ |
| Formula weight | 386.41 |
| Temperature/K | 206 (9) |
| Crystal system | Triclinic |
| Space group | *P-1* |
| a/Å | 9.3699 (7) |
| b/Å | 10.2228 (9) |
| c/Å | 10.3816 (8) |
| α/° | 89.381 (7) |
| β/° | 85.744 (6) |
| γ/° | 77.947 (7) |
| Volume/Å$^3$ | 969.81 (14) |
| Z | 2 |
| Density ($\rho_{calc}$g/cm$^3$) | 1.323 |
| Absorption coefficient (μ/mm$^{-1}$) | 0.091 |
| F(000) | 404 |
| Crystal size/mm$^3$ | 0.436 × 0.310 × 0.196 |
| Radiation | MoKα (λ = 0.71073) |
| 2θ range for data collection/° | 7.8220 to 58.6920 |
| Index ranges | $-12 \leq h \leq 12$, $-13 \leq k \leq 14$, $-13 \leq l \leq 14$ |
| Reflections collected | 8055 |
| Independent reflections | 4580 [$R_{int}$ = 0.0214, $R_{sigma}$ = 0.0394] |
| Data/restraints/parameters | 4580/0/263 |
| Goodness-of-fit on $F^2$ | 1.079 |
| Final R indexes [I>=2σ (I)] | $R_1$ = 0.047, $wR_2$ = 0.103 |
| Final R indexes [all data] | $R_1$ = 0.066, $wR_2$ = 0.116 |
| Largest diff. peak/hole / e Å$^{-3}$ | 0.22/-0.27 |

**Table S2**. Crystal data and structure refinement for compound **3d**. [CCDC: 2095847]

| | |
|---|---|
| Empirical formula | $C_{24}H_{20}N_2O_2$ |
| Formula weight | 368.42 |
| Temperature/K | 179.99(10) |
| Crystal system | Monoclinic |
| Space group | $P2_1/c$ |
| a/Å | 10.3717 (2) |
| b/Å | 7.82398 (19) |
| c/Å | 23.8439 (5) |
| α/° | 90 |
| β/° | 97.135 (2) |
| γ/° | 90 |
| Volume/Å$^3$ | 1919.91 (8) |
| Z | 4 |
| Density ($\rho_{calc}$g/cm$^3$) | 1.275 |
| Absorption coefficient (μ/mm$^{-1}$) | 0.651 |
| F(000) | 776 |
| Crystal size/mm$^3$ | 0.514 × 0.109 × 0.052 |
| Radiation | CuKα (λ = 1.54178) |
| 2θ range for data collection/° | 4.2590 to 72.6830 |
| Index ranges | -12 ≤ h ≤ 12, -9 ≤ k ≤ 9, -23 ≤ l ≤ 29 |
| Reflections collected | 7501 |
| Independent reflections | 3740 [$R_{int}$ = 0.0235, $R_{sigma}$ = 0.0288] |
| Data/restraints/parameters | 3740/0/254 |
| Goodness-of-fit on F$^2$ | 1.045 |
| Final R indexes [I>=2σ (I)] | $R_1$ = 0.040, w$R_2$ = 0.102 |
| Final R indexes [all data] | $R_1$ = 0.048, w$R_2$ = 0.109 |
| Largest diff. peak/hole / e Å$^{-3}$ | 0.19/-0.23 |

# 5. Computational Data

## 5.1 Computational Details:

We used Gaussian 16[7] to fully optimize all the structures reported in this paper at the M06-2X level[8] of density functional theory (DFT) using the SMD solvation model[9] in dichloromethane. The 6-31G(d) basis set[10] was chosen for all atoms. We carried out frequency calculations at the same level of theory as those for structural optimization. Transition structures were located using the Berny algorithm and intrinsic reaction coordinate (IRC) calculations[11] were used to confirm the connectivity between transition structures and minima. To further refine the energies obtained from the SMD/M06-2X/6-31G(d) calculations and consider dispersive interactions,[12] we carried out single-point energy calculations using the M06-2X-D3 functional method for all of the structures with a larger basis set def2-TZVP[13] and the SMD solvation model in dichloromethane. All thermodynamic data were calculated at the standard state (298.15 K and 1 atm). In this work, the free energy for each species optimized by SMD/M06-2X/6-31g(d) in solution was calculated using the formula:

$$G = E(SMD/M06\text{-}2X\text{-}D3/def2\text{-}TZVP) + G(SMD/M06\text{-}2X/6\text{-}31g(d)) - E(SMD/M06\text{-}2X/6\text{-}31g(d)) + \Delta G^{1atm\rightarrow 1M} \quad (1)$$

where $\Delta G^{1atm\rightarrow 1M}$ = 1.89 kcal/mol is the free energy change for compression of 1 mol of an ideal gas from 1 atm to 1 M solution phase standard state.[14] In a simple word, a correction of -1.89 (or 1.89) kcal/mol is employed to estimate relative free energies for a 2-to-1 (or a 1-to-2) transformation.

**Scheme S1**: Stability of 6-H and **2b**•$B(C_6F_5)_3$.

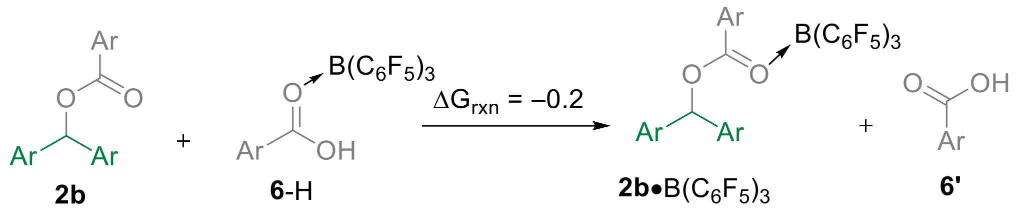

## 5.2 Cartesian coordinates and total energies for the calculated structures

**1b**

E(SMD/M06-2X/6-31g(d)) = -684.8129201 au
H(SMD/M06-2X/6-31g(d)) = -684.603643 au
G(SMD/M06-2X/6-31g(d)) = -684.660965 au
E(SMD/M06-2X-D3/def2-TZVP//SMD/M06-2X/6-31g(d)) = -685.084095 au

| | | | |
|---|---|---|---|
| C | 1.62964500 | 0.29501100 | -0.01216700 |
| N | 1.90048100 | 1.58150400 | -0.04822100 |
| C | 0.24962800 | -0.15533600 | -0.00505800 |
| C | 2.76650200 | -0.62498500 | 0.02056200 |
| O | 2.64641000 | -1.83035600 | 0.05513500 |
| O | 3.94089600 | 0.02070500 | 0.00768200 |
| H | 0.17246500 | -1.23924900 | 0.01586600 |
| C | -0.83547600 | 0.63586800 | -0.01749200 |
| H | -0.70377700 | 1.71856800 | -0.02508000 |
| C | -2.23200100 | 0.18195400 | -0.01142100 |
| C | -2.60189500 | -1.17081800 | -0.09044400 |
| C | -3.25027000 | 1.14251400 | 0.07639900 |
| C | -3.94056200 | -1.54254900 | -0.07333500 |
| H | -1.83930400 | -1.93996400 | -0.17148300 |
| C | -4.59131000 | 0.76989700 | 0.09287300 |
| H | -2.97986100 | 2.19406000 | 0.13435400 |
| C | -4.94323600 | -0.57559500 | 0.01939700 |
| H | -4.20464000 | -2.59421000 | -0.13586200 |
| H | -5.36117000 | 1.53273500 | 0.16327300 |
| H | -5.98796200 | -0.87072600 | 0.03121500 |
| N | 2.08903000 | 2.69097600 | -0.07972300 |
| C | 5.09935400 | -0.82063300 | 0.04020300 |
| H | 5.95284700 | -0.14463100 | 0.02379900 |
| H | 5.10716000 | -1.42142400 | 0.95202800 |
| H | 5.11693100 | -1.47728800 | -0.83214400 |

**4**

E(SMD/M06-2X/6-31g(d)) = -684.8214711 au
H(SMD/M06-2X/6-31g(d)) = -684.6118 au
G(SMD/M06-2X/6-31g(d)) = -684.667788 au
E(SMD/M06-2X-D3/def2-TZVP//SMD/M06-2X/6-31g(d)) = -685.0872703 au

| | | | |
|---|---|---|---|
| C | 1.50551800 | -0.52238600 | 0.05636300 |
| N | 1.01546300 | -1.65496700 | -0.67571800 |
| C | 0.56476200 | -0.07014300 | 0.89476100 |
| C | 2.88293200 | -0.04159900 | -0.19621400 |
| O | 3.64322100 | -0.54412000 | -0.98911500 |
| O | 3.17172200 | 1.01627000 | 0.56424700 |
| H | 0.61897700 | 0.76612500 | 1.57894200 |
| C | -0.61338200 | -0.96104600 | 0.70232700 |
| H | -0.79791600 | -1.56047100 | 1.60574500 |

| | | | |
|---|---|---|---|
| C | -1.89633000 | -0.26540400 | 0.28836000 |
| C | -1.95196300 | 0.39961500 | -0.93911900 |
| C | -3.01218100 | -0.27401100 | 1.12299900 |
| C | -3.11791000 | 1.05175200 | -1.32636400 |
| H | -1.08086100 | 0.40022000 | -1.59087600 |
| C | -4.17875400 | 0.38437400 | 0.73578000 |
| H | -2.96856600 | -0.79592700 | 2.07517700 |
| C | -4.23312800 | 1.04663600 | -0.48773500 |
| H | -3.15715900 | 1.56412900 | -2.28281900 |
| H | -5.04487500 | 0.37492700 | 1.39056400 |
| H | -5.14253600 | 1.55696000 | -0.79034600 |
| N | -0.15829700 | -1.91173500 | -0.33685000 |
| C | 4.48867100 | 1.55264000 | 0.38820700 |
| H | 4.55872200 | 2.39236200 | 1.07774100 |
| H | 5.24027000 | 0.79747800 | 0.62781300 |
| H | 4.62482200 | 1.89133700 | -0.64121500 |

**1b.B($C_6F_5$)$_3$**

E(SMD/M06-2X/6-31g(d)) = -2892.355713 au
H(SMD/M06-2X/6-31g(d)) = -2891.95734 au
G(SMD/M06-2X/6-31g(d)) = -2892.080505 au
E(SMD/M06-2X-D3/def2-TZVP//SMD/M06-2X/6-31g(d)) = -2893.594608 au

| | | | |
|---|---|---|---|
| C | -2.66789400 | -0.40554100 | -0.72268300 |
| N | -2.40457300 | -1.39547800 | -1.58424500 |
| C | -4.05662300 | -0.00330800 | -0.51056100 |
| C | -1.47071000 | 0.13722500 | -0.17708700 |
| O | -0.37440900 | -0.13074000 | -0.76189000 |
| O | -1.42562100 | 0.89914400 | 0.87563600 |
| H | -4.17674500 | 1.00207700 | -0.11929300 |
| C | -5.11798200 | -0.76232900 | -0.81998300 |
| H | -4.96046400 | -1.77468400 | -1.19288300 |
| C | -6.52448700 | -0.35786700 | -0.69313900 |
| C | -6.91816100 | 0.96232600 | -0.42454000 |
| C | -7.51849800 | -1.33435900 | -0.84661900 |
| C | -8.26324900 | 1.28627400 | -0.29669900 |
| H | -6.17144100 | 1.74512700 | -0.32821500 |
| C | -8.86575000 | -1.00873000 | -0.72015200 |
| H | -7.22634800 | -2.35899600 | -1.06208800 |
| C | -9.24305800 | 0.30287600 | -0.44242700 |
| H | -8.55030800 | 2.31307100 | -0.09081600 |
| H | -9.62016300 | -1.78035500 | -0.84036100 |
| H | -10.29318600 | 0.56114800 | -0.34635000 |
| N | -2.19865500 | -2.23714200 | -2.28714900 |
| C | -2.40682300 | 0.83690200 | 1.93608700 |
| H | -3.12433000 | 1.64759200 | 1.81165000 |
| H | -2.89899700 | -0.13596000 | 1.94256500 |
| H | -1.83152000 | 0.98010600 | 2.84999600 |

| | | | |
|---|---|---|---|
| B | 1.02214200 | 0.09189300 | -0.13185200 |
| C | 1.14079500 | -0.64018400 | 1.32533400 |
| C | 0.26002400 | -1.55515300 | 1.88298100 |
| C | 2.31271300 | -0.44797000 | 2.05195400 |
| C | 0.49180900 | -2.20829500 | 3.08875300 |
| C | 2.59046800 | -1.07576700 | 3.25405700 |
| C | 1.66654700 | -1.96900400 | 3.77891100 |
| C | 1.23945800 | 1.70829100 | -0.11161300 |
| C | 1.43849300 | 2.40524200 | -1.29980100 |
| C | 1.13368100 | 2.51012800 | 1.01968500 |
| C | 1.56757900 | 3.78388000 | -1.37537900 |
| C | 1.25630700 | 3.89375200 | 0.99167200 |
| C | 1.47740200 | 4.53603800 | -0.21476200 |
| C | 2.02152900 | -0.74277500 | -1.11637100 |
| C | 1.66728100 | -2.03050200 | -1.51215900 |
| C | 3.28225800 | -0.32964700 | -1.53100700 |
| C | 2.47063200 | -2.84204400 | -2.29966200 |
| C | 4.12027700 | -1.11153500 | -2.31622600 |
| C | 3.71050100 | -2.37556800 | -2.70681700 |
| F | 0.87955000 | 1.97736600 | 2.22521900 |
| F | 1.52029100 | 1.73786600 | -2.46202400 |
| F | 1.76860000 | 4.38987500 | -2.54802200 |
| F | 1.59269000 | 5.86207000 | -0.26108200 |
| F | 1.14594600 | 4.60834500 | 2.11476200 |
| F | -0.90767400 | -1.85937600 | 1.27654100 |
| F | 3.24170500 | 0.39851300 | 1.57729100 |
| F | 3.73022500 | -0.83922300 | 3.90572700 |
| F | 1.90942800 | -2.58776000 | 4.93313800 |
| F | -0.40554800 | -3.06792400 | 3.57605900 |
| F | 0.49768100 | -2.56397400 | -1.11197700 |
| F | 3.75972900 | 0.87578200 | -1.18879500 |
| F | 5.31704200 | -0.65623800 | -2.69388700 |
| F | 4.50248100 | -3.13768500 | -3.45944900 |
| F | 2.06991100 | -4.06559500 | -2.65301700 |

**4.$B(C_6F_5)_3$**

E(SMD/M06-2X/6-31g(d)) = -2892.359403 au
H(SMD/M06-2X/6-31g(d)) = -2891.960458 au
G(SMD/M06-2X/6-31g(d)) = -2892.081811 au
E(SMD/M06-2X-D3/def2-TZVP//SMD/M06-2X/6-31g(d)) = -2893.593888 au

| | | | |
|---|---|---|---|
| C | 2.73350200 | 0.85480700 | -0.16535700 |
| N | 2.91585600 | 0.83006400 | -1.59668300 |
| C | 3.82006600 | 1.37506400 | 0.42894300 |
| C | 1.43712400 | 0.40602400 | 0.34398900 |
| O | 0.48204900 | 0.37746900 | -0.46972700 |
| O | 1.20855400 | 0.03109400 | 1.55638600 |
| H | 4.02250300 | 1.56543100 | 1.47374400 |

| | | | |
|---|---|---|---|
| C | 4.76642400 | 1.69762400 | -0.67532800 |
| H | 4.90320800 | 2.78623700 | -0.75384400 |
| C | 6.11992200 | 1.01783800 | -0.56894900 |
| C | 6.18760800 | -0.37653600 | -0.61912100 |
| C | 7.28419200 | 1.76779200 | -0.41704800 |
| C | 7.41837000 | -1.01641800 | -0.51782200 |
| H | 5.27633400 | -0.95749800 | -0.74461400 |
| C | 8.51608900 | 1.12372900 | -0.31130200 |
| H | 7.22783900 | 2.85230000 | -0.38473700 |
| C | 8.58423000 | -0.26620200 | -0.36168400 |
| H | 7.46843500 | -2.09994200 | -0.56185200 |
| H | 9.42172500 | 1.71077600 | -0.19422500 |
| H | 9.54447600 | -0.76642400 | -0.28217200 |
| N | 4.03948800 | 1.27903900 | -1.88896500 |
| C | 2.20286800 | -0.01912900 | 2.60129900 |
| H | 3.10549900 | -0.51538900 | 2.24248700 |
| H | 2.40377000 | 0.99515900 | 2.94778100 |
| H | 1.73266500 | -0.60166900 | 3.39066800 |
| B | -0.96422000 | -0.08348300 | -0.08138000 |
| C | -1.57398000 | 0.87582300 | 1.08753500 |
| C | -1.05675100 | 2.08081100 | 1.54191800 |
| C | -2.82013800 | 0.54070700 | 1.60761000 |
| C | -1.70691400 | 2.88763500 | 2.46929400 |
| C | -3.50515100 | 1.31181000 | 2.53110400 |
| C | -2.93974900 | 2.50232700 | 2.96613500 |
| C | -0.79894700 | -1.65778800 | 0.30774300 |
| C | -0.50492600 | -2.59211300 | -0.68203700 |
| C | -0.82190100 | -2.16895900 | 1.60091400 |
| C | -0.28729800 | -3.93854300 | -0.43268800 |
| C | -0.60740700 | -3.50941700 | 1.89732100 |
| C | -0.34132000 | -4.40182000 | 0.87271600 |
| C | -1.85244500 | 0.22406600 | -1.41077500 |
| C | -1.74833400 | 1.47436500 | -2.01488000 |
| C | -2.82057900 | -0.61167900 | -1.95469700 |
| C | -2.50302200 | 1.86410800 | -3.11105700 |
| C | -3.59980600 | -0.26030700 | -3.04986700 |
| C | -3.43762700 | 0.98461500 | -3.63476000 |
| F | -1.03004300 | -1.36963200 | 2.65819000 |
| F | -0.42377400 | -2.20412100 | -1.96367000 |
| F | -0.01980800 | -4.78515700 | -1.42920200 |
| F | -0.12871100 | -5.68883000 | 1.13896300 |
| F | -0.63949700 | -3.93844500 | 3.16150900 |
| F | 0.13420000 | 2.53877300 | 1.10707000 |
| F | -3.40708700 | -0.59891900 | 1.20452900 |
| F | -4.69475600 | 0.92971500 | 2.99913900 |
| F | -3.57744300 | 3.26529300 | 3.85256900 |

| | | | |
|---|---|---|---|
| F | -1.15278000 | 4.03172400 | 2.87742300 |
| F | -0.89626900 | 2.38643500 | -1.51876200 |
| F | -3.05581400 | -1.82592700 | -1.43636500 |
| F | -4.50681600 | -1.10971900 | -3.53796100 |
| F | -4.17869900 | 1.33721800 | -4.68381600 |
| F | -2.35183500 | 3.07546000 | -3.65127700 |

**1b′.B($C_6F_5$)$_3$**

E(SMD/M06-2X/6-31g(d)) = -2892.342593 au
H(SMD/M06-2X/6-31g(d)) = -2891.944326 au
G(SMD/M06-2X/6-31g(d)) = -2892.066799 au
E(SMD/M06-2X-D3/def2-TZVP//SMD/M06-2X/6-31g(d)) = -2893.578327 au

| | | | |
|---|---|---|---|
| C | 1.55764300 | 1.87112500 | 1.48738300 |
| C | 1.10405100 | 3.30832500 | 1.45750800 |
| O | 1.88024100 | 4.22134300 | 1.36035700 |
| O | -0.21373200 | 3.38850900 | 1.53421800 |
| C | -0.77526200 | 4.71126300 | 1.47284400 |
| H | -1.84310800 | 4.57825300 | 1.63497800 |
| H | -0.34292400 | 5.33636300 | 2.25563100 |
| H | -0.58238700 | 5.14774400 | 0.49086700 |
| C | 2.96951800 | 1.53546200 | 1.37607300 |
| N | 0.62261100 | 1.00787800 | 1.54255900 |
| N | -0.23027800 | 0.22017200 | 1.59274700 |
| C | 3.43805600 | 0.28721500 | 1.20447800 |
| H | 2.73203700 | -0.54348800 | 1.14227100 |
| C | 4.84687300 | -0.08713100 | 1.06236200 |
| C | 5.89822800 | 0.81743100 | 1.28078100 |
| C | 5.15326100 | -1.40093300 | 0.67876000 |
| C | 7.21608900 | 0.41633600 | 1.10743400 |
| H | 5.68584100 | 1.83462400 | 1.59646500 |
| C | 6.47441300 | -1.79989400 | 0.50264300 |
| H | 4.34672800 | -2.11116900 | 0.51318200 |
| C | 7.50878600 | -0.89171900 | 0.71557700 |
| H | 8.02058100 | 1.12427100 | 1.28146600 |
| H | 6.69457100 | -2.81917100 | 0.20100800 |
| H | 3.61242000 | 2.40935600 | 1.40971500 |
| H | 8.54098500 | -1.20040100 | 0.58144200 |
| B | -0.92054400 | -0.33096300 | 0.15832500 |
| C | 0.41016100 | -0.65759300 | -0.71656700 |
| C | 1.12240600 | 0.34789200 | -1.36967400 |
| C | 1.04178200 | -1.90001200 | -0.70203400 |
| C | 2.35766600 | 0.15542900 | -1.97070600 |
| C | 2.27659900 | -2.13429200 | -1.29158800 |
| C | 2.94661300 | -1.09808900 | -1.92131700 |
| C | -1.84039100 | 0.89975000 | -0.37397500 |
| C | -2.52961300 | 1.75749100 | 0.47516600 |
| C | -2.08220000 | 1.08832900 | -1.73126100 |

| | | | |
|---|---|---|---|
| C | -3.37855800 | 2.75814400 | 0.02217300 |
| C | -2.92666300 | 2.07184000 | -2.22418700 |
| C | -3.57776300 | 2.91796900 | -1.33930000 |
| C | -1.88355900 | -1.57770400 | 0.52659000 |
| C | -2.22021900 | -2.49893700 | -0.46173300 |
| C | -2.54026200 | -1.75040200 | 1.74024800 |
| C | -3.11363200 | -3.54007600 | -0.26963300 |
| C | -3.43990600 | -2.78408100 | 1.97528400 |
| C | -3.72929000 | -3.68432700 | 0.96479000 |
| F | -2.38675500 | 1.65022400 | 1.80496000 |
| F | -2.33761600 | -0.90815200 | 2.76314500 |
| F | -3.99546800 | 3.56739400 | 0.88758500 |
| F | -4.38990800 | 3.86891400 | -1.79339500 |
| F | -3.11270000 | 2.21339100 | -3.53715000 |
| F | -1.47411000 | 0.31125900 | -2.63751700 |
| F | -4.03134600 | -2.90703600 | 3.16514100 |
| F | -1.65721800 | -2.40484000 | -1.67620500 |
| F | -3.38679900 | -4.39749900 | -1.25427800 |
| F | -4.59108300 | -4.67705900 | 1.17372200 |
| F | 0.65147900 | 1.60723700 | -1.40103000 |
| F | 2.99228600 | 1.16884000 | -2.55709700 |
| F | 4.14812800 | -1.30221800 | -2.44993600 |
| F | 2.85225000 | -3.33480000 | -1.20143500 |
| F | 0.49114800 | -2.94561200 | -0.07218700 |

**4′.B($C_6F_5$)$_3$**

E(SMD/M06-2X/6-31g(d)) = -2892.383541 au
H(SMD/M06-2X/6-31g(d)) = -2891.984314 au
G(SMD/M06-2X/6-31g(d)) = -2892.102642 au
E(SMD/M06-2X-D3/def2-TZVP//SMD/M06-2X/6-31g(d)) = -2893.616378 au

| | | | |
|---|---|---|---|
| C | -1.45835400 | 1.84441100 | 2.51290800 |
| C | -2.66064700 | 2.54475500 | 3.03298000 |
| O | -2.65906700 | 3.12978900 | 4.08929900 |
| O | -3.69243200 | 2.44401600 | 2.20661400 |
| C | -4.89249800 | 3.10030500 | 2.64452600 |
| H | -5.62448300 | 2.92346800 | 1.85893100 |
| H | -5.23072500 | 2.67085000 | 3.58946100 |
| H | -4.71074200 | 4.16971200 | 2.76767200 |
| C | -0.24244200 | 1.76004800 | 3.08015800 |
| N | -1.47586600 | 1.14681500 | 1.29530000 |
| N | -0.34272900 | 0.67057000 | 1.06428600 |
| C | 0.61744600 | 0.99197000 | 2.15059900 |
| H | 0.92080500 | 0.03527600 | 2.59724900 |
| C | 1.85245100 | 1.77287700 | 1.71877700 |
| C | 3.07422100 | 1.48387900 | 2.32406900 |
| C | 1.76005300 | 2.77318100 | 0.75150200 |
| C | 4.21640400 | 2.18317300 | 1.94036800 |

| | | | |
|---|---|---|---|
| H | 3.13671900 | 0.69869900 | 3.07204700 |
| C | 2.90774900 | 3.45660500 | 0.36017100 |
| H | 0.80362100 | 2.99774300 | 0.28765000 |
| C | 4.13626600 | 3.16174500 | 0.95202500 |
| H | 5.17004200 | 1.94870300 | 2.40280100 |
| H | 2.84088700 | 4.22020700 | -0.40858800 |
| H | 0.09395000 | 2.18927900 | 4.01523200 |
| H | 5.02966200 | 3.69443200 | 0.64107500 |
| B | -0.14194600 | -0.35943200 | -0.19763400 |
| C | -1.38677500 | -0.04572200 | -1.20643100 |
| C | -2.26594400 | -0.98389000 | -1.73452400 |
| C | -1.65346900 | 1.26745200 | -1.59386100 |
| C | -3.32908200 | -0.65148600 | -2.57143000 |
| C | -2.69779100 | 1.64481800 | -2.41591200 |
| C | -3.55056200 | 0.66831600 | -2.91246600 |
| C | -0.19406300 | -1.86963600 | 0.43213800 |
| C | -0.63483500 | -2.23560400 | 1.69622100 |
| C | 0.24590300 | -2.92986000 | -0.35778800 |
| C | -0.62267800 | -3.54492900 | 2.16550500 |
| C | 0.27163700 | -4.24765700 | 0.06435100 |
| C | -0.16343500 | -4.55882400 | 1.34506300 |
| C | 1.34374700 | -0.07479200 | -0.83176000 |
| C | 1.61234200 | 0.54926500 | -2.04885900 |
| C | 2.48640500 | -0.43506700 | -0.11893800 |
| C | 2.89596200 | 0.84971900 | -2.49066400 |
| C | 3.78185600 | -0.14008800 | -0.51116000 |
| C | 3.99053800 | 0.51559700 | -1.71311000 |
| F | -1.09662400 | -1.31633200 | 2.56989700 |
| F | 2.37312800 | -1.05079200 | 1.07413900 |
| F | -1.04938700 | -3.82449000 | 3.39840700 |
| F | -0.14561600 | -5.81903000 | 1.77338900 |
| F | 0.69805000 | -5.21756600 | -0.74599500 |
| F | 0.63137700 | -2.69459500 | -1.62118100 |
| F | 4.81562700 | -0.45934500 | 0.26813500 |
| F | 0.63440500 | 0.91042400 | -2.89086000 |
| F | 3.07706600 | 1.46217200 | -3.66214800 |
| F | 5.22254700 | 0.81800600 | -2.11258200 |
| F | -2.15001100 | -2.29357800 | -1.47092300 |
| F | -4.13615700 | -1.60530600 | -3.04093400 |
| F | -4.56347800 | 1.00181200 | -3.70863500 |
| F | -2.88823400 | 2.92505400 | -2.74121800 |
| F | -0.84716900 | 2.25615400 | -1.16799200 |

**2b**

E(SMD/M06-2X/6-31g(d)) = -1219.483091 au
H(SMD/M06-2X/6-31g(d)) = -1219.177488 au
G(SMD/M06-2X/6-31g(d)) = -1219.249571 au

E(SMD/M06-2X-D3/def2-TZVP//SMD/M06-2X/6-31g(d)) = -1219.974721 au

| | | | |
|---|---|---|---|
| C | -4.65543500 | 0.15062200 | -1.31524100 |
| C | -3.26748700 | 0.13648800 | -1.33159300 |
| C | -2.54721400 | 0.07546400 | -0.13506900 |
| C | -3.22177700 | 0.02866500 | 1.08895900 |
| C | -4.61090700 | 0.04232100 | 1.11970700 |
| C | -5.29688700 | 0.10303800 | -0.08503400 |
| H | -5.24115800 | 0.19703700 | -2.22668200 |
| H | -2.72765700 | 0.17249300 | -2.27201800 |
| H | -2.66124900 | -0.01882400 | 2.01569900 |
| H | -5.16382300 | 0.00686100 | 2.05190700 |
| F | -6.63826500 | 0.11665900 | -0.05944400 |
| C | -1.06042500 | 0.06655000 | -0.22219600 |
| O | -0.44422400 | 0.10234000 | -1.26256300 |
| O | -0.48631400 | 0.03044000 | 0.99185600 |
| C | 0.95646600 | -0.01449000 | 1.08737100 |
| H | 1.09551800 | -0.03803800 | 2.17290000 |
| C | 1.60155700 | 1.26289100 | 0.57542800 |
| C | 0.85268400 | 2.42996900 | 0.40693900 |
| C | 2.98407800 | 1.31408600 | 0.37536100 |
| C | 1.46054800 | 3.62080100 | 0.02094700 |
| H | -0.21868800 | 2.42139300 | 0.58165800 |
| C | 3.60982400 | 2.49743000 | -0.00669300 |
| H | 3.58701500 | 0.42226200 | 0.51805900 |
| C | 2.83101400 | 3.62946200 | -0.18154600 |
| H | 0.88854700 | 4.53145000 | -0.12119300 |
| H | 4.68098500 | 2.54785400 | -0.17004700 |
| F | 3.42589600 | 4.77744700 | -0.55712100 |
| C | 1.50076400 | -1.32395800 | 0.54305700 |
| C | 1.49230800 | -2.43436000 | 1.39067300 |
| C | 1.98012400 | -1.47046300 | -0.76269500 |
| C | 1.94533900 | -3.67677700 | 0.95575000 |
| H | 1.12568200 | -2.32758200 | 2.40854700 |
| C | 2.44459000 | -2.70109200 | -1.21366900 |
| H | 1.98412000 | -0.61797700 | -1.43271700 |
| C | 2.41437300 | -3.78107300 | -0.34332000 |
| H | 1.94556100 | -4.54702100 | 1.60315400 |
| H | 2.82147900 | -2.83390200 | -2.22214600 |
| F | 2.86200900 | -4.97318300 | -0.77724400 |

**$B(C_6F_5)_3$**

E(SMD/M06-2X/6-31g(d)) = -2207.510899 au

H(SMD/M06-2X/6-31g(d)) = -2207.324247 au

G(SMD/M06-2X/6-31g(d)) = -2207.412287 au

E(SMD/M06-2X-D3/def2-TZVP//SMD/M06-2X/6-31g(d)) = -2208.482703 au

| | | | |
|---|---|---|---|
| B | 0.00101400 | 0.00047300 | -0.00146200 |
| C | -1.01385200 | 1.19723700 | -0.00049400 |

| | | | |
|---|---|---|---|
| C | -0.73900500 | 2.41131000 | -0.63596100 |
| C | -1.62598400 | 3.47512700 | -0.65248400 |
| C | -2.84341500 | 3.35350100 | 0.00107900 |
| C | -3.16075200 | 2.17170200 | 0.65444400 |
| C | -2.25585400 | 1.12315700 | 0.63608700 |
| C | 1.54486400 | 0.28047700 | -0.00150100 |
| C | 2.45779200 | -0.56598900 | -0.63675600 |
| C | 3.82284200 | -0.33128900 | -0.65357900 |
| C | 4.32801600 | 0.78266700 | 0.00086600 |
| C | 3.46426400 | 1.65007900 | 0.65348400 |
| C | 2.10330000 | 1.39282000 | 0.63441200 |
| C | -0.52868300 | -1.47643400 | -0.00158800 |
| C | 0.15487100 | -2.51628400 | 0.63470100 |
| C | -0.30323000 | -3.82333800 | 0.65344300 |
| C | -1.48615900 | -4.13730300 | 0.00042200 |
| C | -2.19837000 | -3.14249400 | -0.65341800 |
| C | -1.71803500 | -1.84346000 | -0.63723700 |
| F | 0.41642000 | 2.59168200 | -1.28104500 |
| F | -1.32120800 | 4.60687600 | -1.28391500 |
| F | -3.70137800 | 4.36389900 | 0.00158400 |
| F | -4.32668800 | 2.05459200 | 1.28638500 |
| F | -2.62115900 | 0.01225400 | 1.28115800 |
| F | 2.03472900 | -1.65635600 | -1.28153400 |
| F | 4.64935800 | -1.16283300 | -1.28432900 |
| F | 5.63257600 | 1.01745800 | 0.00252900 |
| F | 3.94707700 | 2.71766100 | 1.28552100 |
| F | 1.32508900 | 2.26687000 | 1.27783500 |
| F | -2.45009400 | -0.93164200 | -1.28249800 |
| F | -3.33188700 | -3.44215700 | -1.28409000 |
| F | -1.93474500 | -5.38462700 | 0.00127100 |
| F | 0.37937400 | -4.77579900 | 1.28542700 |
| F | 1.30023600 | -2.27912900 | 1.27923200 |

**2b.B($C_6F_5$)$_3$**

E(SMD/M06-2X/6-31g(d)) = -3427.02968 au
H(SMD/M06-2X/6-31g(d)) = -3426.535599 au
G(SMD/M06-2X/6-31g(d)) = -3426.671342 au
E(SMD/M06-2X-D3/def2-TZVP//SMD/M06-2X/6-31g(d)) = -3428.489476 au

| | | | |
|---|---|---|---|
| C | -4.65543500 | 0.15062200 | -1.31524100 |
| C | -3.26748700 | 0.13648800 | -1.33159300 |
| C | -2.54721400 | 0.07546400 | -0.13506900 |
| C | -3.22177700 | 0.02866500 | 1.08895900 |
| C | -4.61090700 | 0.04232100 | 1.11970700 |
| C | -5.29688700 | 0.10303800 | -0.08503400 |
| H | -5.24115800 | 0.19703700 | -2.22668200 |
| H | -2.72765700 | 0.17249300 | -2.27201800 |
| H | -2.66124900 | -0.01882400 | 2.01569900 |

| | | | |
|---|---|---|---|
| H | -5.16382300 | 0.00686100 | 2.05190700 |
| F | -6.63826500 | 0.11665900 | -0.05944400 |
| C | -1.06042500 | 0.06655000 | -0.22219600 |
| O | -0.44422400 | 0.10234000 | -1.26256300 |
| O | -0.48631400 | 0.03044000 | 0.99185600 |
| C | 0.95646600 | -0.01449000 | 1.08737100 |
| H | 1.09551800 | -0.03803800 | 2.17290000 |
| C | 1.60155700 | 1.26289100 | 0.57542800 |
| C | 0.85268400 | 2.42996900 | 0.40693900 |
| C | 2.98407800 | 1.31408600 | 0.37536100 |
| C | 1.46054800 | 3.62080100 | 0.02094700 |
| H | -0.21868800 | 2.42139300 | 0.58165800 |
| C | 3.60982400 | 2.49743000 | -0.00669300 |
| H | 3.58701500 | 0.42226200 | 0.51805900 |
| C | 2.83101400 | 3.62946200 | -0.18154600 |
| H | 0.88854700 | 4.53145000 | -0.12119300 |
| H | 4.68098500 | 2.54785400 | -0.17004700 |
| F | 3.42589600 | 4.77744700 | -0.55712100 |
| C | 1.50076400 | -1.32395800 | 0.54305700 |
| C | 1.49230800 | -2.43436000 | 1.39067300 |
| C | 1.98012400 | -1.47046300 | -0.76269500 |
| C | 1.94533900 | -3.67677700 | 0.95575000 |
| H | 1.12568200 | -2.32758200 | 2.40854700 |
| C | 2.44459000 | -2.70109200 | -1.21366900 |
| H | 1.98412000 | -0.61797700 | -1.43271700 |
| C | 2.41437300 | -3.78107300 | -0.34332000 |
| H | 1.94556100 | -4.54702100 | 1.60315400 |
| H | 2.82147900 | -2.83390200 | -2.22214600 |
| F | 2.86200900 | -4.97318300 | -0.77724400 |

**$TS_1$**

E(SMD/M06-2X/6-31g(d)) = -3427.001605 au
H(SMD/M06-2X/6-31g(d)) = -3426.50999 au
G(SMD/M06-2X/6-31g(d)) = -3426.645082 au
E(SMD/M06-2X-D3/def2-TZVP//SMD/M06-2X/6-31g(d)) = -3428.463298 au

| | | | |
|---|---|---|---|
| C | -4.10294300 | 0.48248000 | -2.21437300 |
| C | -2.72114000 | 0.53432600 | -2.06701600 |
| C | -1.96464100 | -0.63682500 | -2.13012900 |
| C | -2.58811800 | -1.86506400 | -2.36962300 |
| C | -3.97033500 | -1.93382400 | -2.49326100 |
| C | -4.69898300 | -0.75472000 | -2.40626200 |
| H | -4.71557400 | 1.37639000 | -2.17350900 |
| H | -2.23076100 | 1.48917900 | -1.92228800 |
| H | -1.98836800 | -2.76800700 | -2.43176900 |
| H | -4.48595100 | -2.87485500 | -2.65206700 |
| F | -6.03474100 | -0.81562000 | -2.52530200 |
| C | -0.47899400 | -0.61519700 | -1.94940300 |

| | | | |
|---|---|---|---|
| O | 0.06916600 | 0.08889200 | -1.00700400 |
| O | 0.21376200 | -1.26791500 | -2.73737300 |
| C | 2.41206400 | -1.43227100 | -2.60845800 |
| H | 2.16560700 | -1.77279100 | -3.60857400 |
| C | 2.59006100 | -2.45965100 | -1.63299800 |
| C | 2.52741000 | -3.80123100 | -2.07326000 |
| C | 2.77113400 | -2.19054200 | -0.25738000 |
| C | 2.69588100 | -4.84377800 | -1.18346400 |
| H | 2.35748500 | -4.00651500 | -3.12620600 |
| C | 2.92942100 | -3.22557800 | 0.64434400 |
| H | 2.74641400 | -1.16881500 | 0.10265200 |
| C | 2.89540200 | -4.52991200 | 0.15886200 |
| H | 2.66570100 | -5.88179800 | -1.49402200 |
| H | 3.04639100 | -3.05053000 | 1.70874400 |
| F | 3.04456100 | -5.53036800 | 1.02432300 |
| B | -0.45961800 | 0.42321600 | 0.38705700 |
| C | -0.95554700 | 1.97852800 | 0.49882200 |
| C | -1.43147500 | 2.43628900 | 1.72343200 |
| C | -0.79174600 | 2.96369000 | -0.46487300 |
| C | -1.76574200 | 3.75502600 | 1.98142700 |
| C | -1.11597000 | 4.29974800 | -0.25468600 |
| C | -1.60224200 | 4.69948400 | 0.97783600 |
| C | 0.90843500 | 0.35342900 | 1.30297500 |
| C | 1.15231800 | -0.44204000 | 2.41554300 |
| C | 1.98032300 | 1.16049000 | 0.91666100 |
| C | 2.38266300 | -0.49256700 | 3.06305300 |
| C | 3.21049100 | 1.16907400 | 1.55918700 |
| C | 3.42010000 | 0.31623300 | 2.63314100 |
| C | -1.59403200 | -0.69457300 | 0.77781900 |
| C | -2.95406300 | -0.46287300 | 0.94893600 |
| C | -1.24064900 | -2.04114100 | 0.77068500 |
| C | -3.89268800 | -1.47772200 | 1.10190500 |
| C | -2.13505600 | -3.08575400 | 0.93551000 |
| C | -3.48129100 | -2.79900700 | 1.10330500 |
| F | -0.31959200 | 2.66492500 | -1.68871400 |
| F | -0.94931500 | 5.20058200 | -1.22763100 |
| F | -1.91407400 | 5.97672200 | 1.19861500 |
| F | -2.22934500 | 4.12784600 | 3.17723300 |
| F | -1.59098200 | 1.55936400 | 2.73008600 |
| F | 1.85180400 | 1.98988600 | -0.13049100 |
| F | 4.19195100 | 1.98467000 | 1.16492700 |
| F | 4.59874500 | 0.29138800 | 3.25365200 |
| F | 2.57384200 | -1.32589300 | 4.08973100 |
| F | 0.20201800 | -1.23511500 | 2.93240000 |
| F | -3.46121400 | 0.77878600 | 0.91494400 |
| F | -5.18983800 | -1.18523100 | 1.22363500 |

| | | | |
|---|---|---|---|
| F | -4.36807600 | -3.78309700 | 1.24360100 |
| F | -1.72065100 | -4.35540000 | 0.91293700 |
| F | 0.04007100 | -2.38771800 | 0.54474900 |
| C | 2.85475100 | -0.06115400 | -2.49689200 |
| C | 2.24788300 | 0.91984500 | -3.30795800 |
| C | 3.96284500 | 0.29128100 | -1.70173000 |
| C | 2.69224200 | 2.22720500 | -3.28543000 |
| H | 1.39825900 | 0.64484400 | -3.92500600 |
| C | 4.45023900 | 1.59005800 | -1.71400600 |
| H | 4.49137700 | -0.46495200 | -1.13174800 |
| C | 3.79144500 | 2.53362300 | -2.48749500 |
| H | 2.21986300 | 3.00613400 | -3.87338600 |
| H | 5.32067700 | 1.87836700 | -1.13706700 |
| F | 4.24226100 | 3.79062200 | -2.48364000 |

**6**

E(SMD/M06-2X/6-31g(d)) = -2726.980938 au
H(SMD/M06-2X/6-31g(d)) = -2726.687568 au
G(SMD/M06-2X/6-31g(d)) = -2726.797367 au
E(SMD/M06-2X-D3/def2-TZVP//SMD/M06-2X/6-31g(d)) = -2728.172605 au

| | | | |
|---|---|---|---|
| B | 0.20471700 | 0.08880100 | 0.04277400 |
| C | 0.91726100 | -0.72084400 | 1.28893100 |
| C | 2.12255900 | -0.40183000 | 1.90335600 |
| C | 0.33279600 | -1.90100900 | 1.74297700 |
| C | 2.69337900 | -1.16334100 | 2.91651000 |
| C | 0.86450300 | -2.68963400 | 2.75360500 |
| C | 2.05897200 | -2.31568100 | 3.34775100 |
| C | 0.73116200 | 1.63542800 | -0.12018500 |
| C | 1.06137600 | 2.27226500 | -1.30987000 |
| C | 0.73911300 | 2.46408700 | 0.99701100 |
| C | 1.40492500 | 3.61704800 | -1.38919100 |
| C | 1.07289900 | 3.80980100 | 0.96716100 |
| C | 1.40839100 | 4.39391500 | -0.24360600 |
| C | 0.47450000 | -0.87176900 | -1.26092700 |
| C | 1.76205100 | -0.97242000 | -1.77709600 |
| C | -0.44819100 | -1.73801500 | -1.83343900 |
| C | 2.12258500 | -1.82333400 | -2.80836500 |
| C | -0.13266900 | -2.61045900 | -2.87087300 |
| C | 1.15971400 | -2.65421300 | -3.36257400 |
| F | -0.80256300 | -2.35426200 | 1.18289400 |
| F | 0.24822500 | -3.80923200 | 3.14665000 |
| F | 2.59348300 | -3.05923100 | 4.31811400 |
| F | 3.85099300 | -0.79518200 | 3.47537700 |
| F | 2.81543000 | 0.69231300 | 1.54687300 |
| F | 0.42924800 | 1.95996100 | 2.20501400 |
| F | 1.07263900 | 4.54450800 | 2.08524900 |
| F | 1.73169700 | 5.68749600 | -0.30433900 |

| | | | |
|---|---|---|---|
| F | 1.71719200 | 4.17196600 | -2.56614600 |
| F | 1.06467100 | 1.61146300 | -2.47692500 |
| F | 2.73683200 | -0.20113500 | -1.26068800 |
| F | 3.37860700 | -1.86213500 | -3.26573900 |
| F | 1.47895800 | -3.48822600 | -4.35475700 |
| F | -1.06654100 | -3.41522400 | -3.38973800 |
| F | -1.72249200 | -1.79069800 | -1.40817300 |
| O | -1.24802900 | 0.15217400 | 0.39963400 |
| C | -2.12313200 | 0.64683400 | -0.44974500 |
| O | -1.84623200 | 1.18000600 | -1.50988500 |
| C | -3.53745300 | 0.48665900 | 0.01661800 |
| C | -3.85638900 | -0.37184600 | 1.07181300 |
| C | -4.54864900 | 1.18841100 | -0.64302600 |
| C | -5.17843100 | -0.52455400 | 1.47542000 |
| H | -3.06438600 | -0.92897400 | 1.56080400 |
| C | -5.87364700 | 1.05342500 | -0.24611100 |
| H | -4.28418200 | 1.84071900 | -1.46911700 |
| C | -6.15768700 | 0.19708800 | 0.80839300 |
| H | -5.45845700 | -1.18782800 | 2.28672700 |
| H | -6.67927700 | 1.59056500 | -0.73491600 |
| F | -7.43780300 | 0.05739700 | 1.19715700 |

**7**

E(SMD/M06-2X/6-31g(d)) = -700.0076624 au
H(SMD/M06-2X/6-31g(d)) = -699.809981 au
G(SMD/M06-2X/6-31g(d)) = -699.861286 au
E(SMD/M06-2X-D3/def2-TZVP//SMD/M06-2X/6-31g(d)) = -700.2822176 au

| | | | |
|---|---|---|---|
| C | 0.00003000 | 1.20311100 | -0.00002700 |
| H | 0.00001200 | 2.29322200 | -0.00012900 |
| C | -1.27886600 | 0.60444900 | -0.04411500 |
| C | -2.38693100 | 1.43155200 | 0.28396500 |
| C | -1.51187900 | -0.74473000 | -0.42649100 |
| C | -3.66509600 | 0.91804800 | 0.32036400 |
| H | -2.21163900 | 2.47304800 | 0.53688400 |
| C | -2.78968700 | -1.25780300 | -0.41756800 |
| H | -0.69620400 | -1.35885500 | -0.79062400 |
| C | -3.83696800 | -0.42139500 | -0.02508000 |
| H | -4.52602800 | 1.51847900 | 0.59081700 |
| H | -3.00561100 | -2.27467200 | -0.72457200 |
| F | -5.06395000 | -0.92169500 | -0.01040100 |
| C | 1.27889200 | 0.60452500 | 0.04421400 |
| C | 2.38690900 | 1.43159000 | -0.28397500 |
| C | 1.51189900 | -0.74460500 | 0.42666700 |
| C | 3.66508600 | 0.91802600 | -0.32036900 |
| H | 2.21169500 | 2.47307200 | -0.53695100 |
| C | 2.78968700 | -1.25776300 | 0.41764800 |
| H | 0.69616100 | -1.35865600 | 0.79078800 |

| | | | |
|---|---|---|---|
| C | 3.83695500 | -0.42139000 | 0.02508200 |
| H | 4.52598400 | 1.51845500 | -0.59089800 |
| H | 3.00552400 | -2.27464400 | 0.72463100 |
| F | 5.06394000 | -0.92176500 | 0.01019700 |

**$TS_2$**

E(SMD/M06-2X/6-31g(d)) = -1384.821706 au
H(SMD/M06-2X/6-31g(d)) = -1384.413678 au
G(SMD/M06-2X/6-31g(d)) = -1384.500202 au
E(SMD/M06-2X-D3/def2-TZVP//SMD/M06-2X/6-31g(d)) = -1385.364284 au

| | | | |
|---|---|---|---|
| C | 2.04756000 | 0.03198200 | 0.62257300 |
| C | -0.71418400 | 2.09345100 | -0.38165200 |
| C | -0.51705700 | 3.50733800 | 0.08909400 |
| O | -1.44403400 | 4.20246100 | 0.41439600 |
| O | 0.76364100 | 3.84497900 | 0.10111900 |
| C | 1.04252000 | 5.18961800 | 0.53644200 |
| H | 2.12423100 | 5.29216200 | 0.48332500 |
| H | 0.55354200 | 5.90270400 | -0.12916200 |
| H | 0.68980400 | 5.32839300 | 1.55980900 |
| C | -2.03912200 | 1.52272200 | -0.45527100 |
| N | 0.35732300 | 1.48055400 | -0.75984700 |
| N | 1.32821500 | 0.91694700 | -1.01428400 |
| C | -2.29824300 | 0.24155000 | -0.78417500 |
| H | -1.46297000 | -0.42115000 | -1.01488500 |
| C | -3.62227600 | -0.37405800 | -0.86713500 |
| C | -4.81196100 | 0.33604300 | -0.63465000 |
| C | -3.70096500 | -1.73848600 | -1.18457600 |
| C | -6.03943200 | -0.30740200 | -0.71530400 |
| H | -4.78095300 | 1.39411800 | -0.39278400 |
| C | -4.93179700 | -2.38074700 | -1.26645800 |
| H | -2.78310200 | -2.29209600 | -1.36855800 |
| C | -6.10423100 | -1.66645600 | -1.03041900 |
| H | -6.95200300 | 0.25172100 | -0.53345100 |
| H | -4.97559700 | -3.43695100 | -1.51307200 |
| H | -2.81583700 | 2.23484200 | -0.19618800 |
| C | 0.89176900 | -0.74829600 | 1.01330900 |
| C | 0.01189700 | -0.19916400 | 1.96569300 |
| C | 0.53368100 | -1.93916700 | 0.34385900 |
| C | -1.19025300 | -0.82230100 | 2.26305800 |
| H | 0.28614600 | 0.71844700 | 2.47776100 |
| C | -0.66115600 | -2.56982000 | 0.63392600 |
| H | 1.17139200 | -2.33901100 | -0.43799200 |
| C | -1.50579700 | -1.98944700 | 1.58014600 |
| H | -1.88545000 | -0.41819800 | 2.99038100 |
| H | -0.96994100 | -3.47752300 | 0.12710600 |
| F | -2.67490100 | -2.58009100 | 1.82610500 |
| C | 3.32177500 | -0.50536300 | 0.14830200 |

| | | | |
|---|---|---|---|
| C | 3.68330500 | -1.85168300 | 0.31763700 |
| C | 4.24472900 | 0.38709200 | -0.42744800 |
| C | 4.92903100 | -2.30276300 | -0.09795300 |
| H | 3.01391900 | -2.54301000 | 0.81713300 |
| C | 5.48176000 | -0.05386800 | -0.86279800 |
| H | 3.97492900 | 1.43354800 | -0.54562900 |
| C | 5.79715700 | -1.39769700 | -0.69019900 |
| H | 5.23567800 | -3.33378800 | 0.03803300 |
| H | 6.20198500 | 0.61459300 | -1.32098500 |
| F | 6.99397000 | -1.82989400 | -1.09961900 |
| H | 2.12177100 | 1.00471100 | 1.11188000 |
| H | -7.06704600 | -2.16414800 | -1.09229400 |

**8**

E(SMD/M06-2X/6-31g(d)) = -1384.829467 au
H(SMD/M06-2X/6-31g(d)) = -1384.419388 au
G(SMD/M06-2X/6-31g(d)) = -1384.50583 au
E(SMD/M06-2X-D3/def2-TZVP//SMD/M06-2X/6-31g(d)) = -1385.371556 au

| | | | |
|---|---|---|---|
| C | 1.63898600 | -0.29589300 | 0.46490300 |
| C | -0.58688500 | 2.27536900 | -0.14060400 |
| C | -0.10828100 | 3.69707000 | 0.12514000 |
| O | -0.88757900 | 4.58118600 | 0.34990200 |
| O | 1.20525400 | 3.77409700 | 0.06933100 |
| C | 1.76127600 | 5.08723500 | 0.29971800 |
| H | 2.83786000 | 4.96240300 | 0.20995200 |
| H | 1.38550200 | 5.78080800 | -0.45374600 |
| H | 1.49053200 | 5.42879100 | 1.29981800 |
| C | -1.99803300 | 1.96791600 | -0.10643500 |
| N | 0.31610200 | 1.41589700 | -0.37235900 |
| N | 1.10535900 | 0.58779400 | -0.67287500 |
| C | -2.47857100 | 0.72230300 | -0.30903000 |
| H | -1.77546300 | -0.08823300 | -0.50935400 |
| C | -3.87986500 | 0.32701100 | -0.28353200 |
| C | -4.92378300 | 1.24421800 | -0.07083700 |
| C | -4.18961600 | -1.02876600 | -0.47444200 |
| C | -6.24035200 | 0.80867300 | -0.05002500 |
| H | -4.70579600 | 2.29792700 | 0.07518800 |
| C | -5.51039900 | -1.46172600 | -0.45239400 |
| H | -3.38520700 | -1.74154300 | -0.63858600 |
| C | -6.53670800 | -0.54369400 | -0.24008300 |
| H | -7.04150400 | 1.52235700 | 0.11314200 |
| H | -5.73817500 | -2.51239200 | -0.60065200 |
| H | -2.62304800 | 2.82771500 | 0.10816200 |
| C | 0.68798600 | -1.46634500 | 0.43155800 |
| C | -0.25099200 | -1.60643000 | 1.45666400 |
| C | 0.64320700 | -2.31580000 | -0.68041000 |
| C | -1.22348400 | -2.59763200 | 1.38816900 |

| | | | |
|---|---|---|---|
| H | -0.22703500 | -0.93158900 | 2.30817100 |
| C | -0.32827500 | -3.30578300 | -0.76560700 |
| H | 1.36233500 | -2.19665600 | -1.48640400 |
| C | -1.24356000 | -3.42171600 | 0.27180300 |
| H | -1.96364800 | -2.72953300 | 2.16984000 |
| H | -0.39079000 | -3.97452000 | -1.61703700 |
| C | 3.10180400 | -0.53931700 | 0.18726000 |
| C | 3.67196000 | -1.80477300 | 0.32739500 |
| C | 3.90888100 | 0.54938700 | -0.15986200 |
| C | 5.03606600 | -1.98846200 | 0.11815400 |
| H | 3.05812600 | -2.65486200 | 0.60722900 |
| C | 5.26915800 | 0.37943800 | -0.38287100 |
| H | 3.47417700 | 1.54232500 | -0.25575100 |
| C | 5.80490700 | -0.89249200 | -0.23772600 |
| H | 5.50153300 | -2.96220900 | 0.22387200 |
| H | 5.91173000 | 1.20810000 | -0.65918300 |
| F | 7.11915700 | -1.06509200 | -0.44818300 |
| F | -2.20203500 | -4.35484300 | 0.18138200 |
| H | 1.49980800 | 0.27221600 | 1.39069500 |
| H | -7.56923400 | -0.87852500 | -0.22280100 |

**$TS_3$**

E(SMD/M06-2X/6-31g(d)) = -1384.826031 au
H(SMD/M06-2X/6-31g(d)) = -1384.419039 au
G(SMD/M06-2X/6-31g(d)) = -1384.50116 au
E(SMD/M06-2X-D3/def2-TZVP//SMD/M06-2X/6-31g(d)) = -1385.365176 au

| | | | |
|---|---|---|---|
| C | -0.17358500 | 1.28691300 | -1.05819200 |
| C | -2.49404700 | -1.34042600 | -0.39657200 |
| C | -3.98012100 | -1.53537200 | -0.44691900 |
| O | -4.52178800 | -2.46714400 | 0.09622700 |
| O | -4.58159900 | -0.57838000 | -1.13219600 |
| C | -6.01251500 | -0.69625200 | -1.22117000 |
| H | -6.33499300 | 0.14113900 | -1.83641100 |
| H | -6.27943600 | -1.64539900 | -1.68924800 |
| H | -6.45295700 | -0.63612900 | -0.22413000 |
| C | -1.68129600 | -2.27770400 | 0.15494000 |
| N | -1.94885700 | -0.17636700 | -0.93217600 |
| N | -0.90430600 | 0.19040700 | -0.37956200 |
| C | -0.26421200 | -2.10829300 | 0.13193000 |
| H | 0.17990500 | -1.76149500 | -0.80072300 |
| C | 0.64225800 | -2.51506500 | 1.13391400 |
| C | 0.21319000 | -2.98330100 | 2.40141900 |
| C | 2.02565400 | -2.41404800 | 0.84463600 |
| C | 1.15342000 | -3.35405300 | 3.34123100 |
| H | -0.84763600 | -3.04773900 | 2.62653000 |
| C | 2.95769700 | -2.78710200 | 1.79464500 |
| H | 2.33409900 | -2.04579900 | -0.13162400 |

| | | | |
|---|---|---|---|
| C | 2.51865700 | -3.25622600 | 3.03667900 |
| H | 0.84064300 | -3.71739700 | 4.31377800 |
| H | 4.01853000 | -2.71772700 | 1.58105100 |
| H | -2.12956500 | -3.13933800 | 0.64309900 |
| C | 1.17589900 | 0.70764300 | -1.47434400 |
| C | 1.20268400 | -0.14840900 | -2.58258300 |
| C | 2.35911800 | 0.96725600 | -0.78283900 |
| C | 2.38818700 | -0.74406200 | -2.99685400 |
| H | 0.28202300 | -0.34890900 | -3.12698000 |
| C | 3.55888700 | 0.38652900 | -1.19020400 |
| H | 2.36135100 | 1.63637200 | 0.07105600 |
| C | 3.54788500 | -0.45994300 | -2.28702700 |
| H | 2.42848100 | -1.40405300 | -3.85660700 |
| H | 4.49112800 | 0.58529000 | -0.67256300 |
| F | 4.70119000 | -1.01929700 | -2.68545900 |
| C | -0.11722200 | 2.45498600 | -0.09700100 |
| C | 0.14841500 | 2.26781700 | 1.26350000 |
| C | -0.32913000 | 3.74517200 | -0.58515400 |
| C | 0.21128500 | 3.35671700 | 2.12677200 |
| H | 0.30676700 | 1.26639800 | 1.65418700 |
| C | -0.26400100 | 4.84538900 | 0.26436400 |
| H | -0.54588800 | 3.89283800 | -1.63932300 |
| C | 0.00512200 | 4.62551700 | 1.60602400 |
| H | 0.41432200 | 3.23567200 | 3.18533300 |
| H | -0.42421400 | 5.85576800 | -0.09582900 |
| F | 0.06388500 | 5.68072700 | 2.43584600 |
| H | -0.73272100 | 1.57135800 | -1.95562300 |
| H | 3.25034600 | -3.54989700 | 3.78331300 |

**9**

E(SMD/M06-2X/6-31g(d)) = -1384.876163 au
H(SMD/M06-2X/6-31g(d)) = -1384.46628 au
G(SMD/M06-2X/6-31g(d)) = -1384.546778 au
E(SMD/M06-2X-D3/def2-TZVP//SMD/M06-2X/6-31g(d)) = -1385.414581 au

| | | | |
|---|---|---|---|
| C | 0.01954100 | 0.80544900 | -1.17939800 |
| C | -2.14558500 | -1.98833500 | -0.53750900 |
| C | -3.54998100 | -2.44958500 | -0.35999700 |
| O | -3.81349400 | -3.61984000 | -0.23531800 |
| O | -4.39592500 | -1.43187600 | -0.35826500 |
| C | -5.79626100 | -1.72115400 | -0.19032700 |
| H | -6.13233100 | -1.18630700 | 0.69834100 |
| H | -6.31304000 | -1.34572600 | -1.07399600 |
| H | -5.95567100 | -2.79227600 | -0.07754300 |
| C | -1.02750200 | -2.74145800 | -0.55680900 |
| N | -1.82726200 | -0.63727900 | -0.72076200 |
| N | -0.58696800 | -0.52888800 | -0.81749100 |
| C | 0.12425300 | -1.82770000 | -0.71809900 |

| | | | |
|---|---|---|---|
| H | 0.63498000 | -1.98529900 | -1.68061900 |
| C | 1.12249700 | -1.89183000 | 0.43029100 |
| C | 0.69863600 | -1.59002800 | 1.72522400 |
| C | 2.43920600 | -2.26527500 | 0.17759200 |
| C | 1.61572600 | -1.62867600 | 2.77048600 |
| H | -0.34127700 | -1.32577700 | 1.91220200 |
| C | 3.34908500 | -2.31397700 | 1.23150800 |
| H | 2.75269800 | -2.50010000 | -0.83548800 |
| C | 2.94109800 | -1.98731500 | 2.52286300 |
| H | 1.29473400 | -1.38573000 | 3.77833800 |
| H | 4.37953000 | -2.59538000 | 1.03808700 |
| H | -0.94341400 | -3.81450000 | -0.43882100 |
| C | 1.52533400 | 0.77724000 | -1.00972400 |
| C | 2.30706900 | 0.35875600 | -2.08936100 |
| C | 2.14602400 | 1.14595900 | 0.18801800 |
| C | 3.69158600 | 0.27571500 | -1.97718100 |
| H | 1.82950300 | 0.09462800 | -3.02974000 |
| C | 3.52610800 | 1.06612500 | 0.31825300 |
| H | 1.55653400 | 1.49409900 | 1.02981600 |
| C | 4.27019000 | 0.62362300 | -0.76729400 |
| H | 4.31586800 | -0.04489900 | -2.80404000 |
| H | 4.02882600 | 1.33705000 | 1.24040300 |
| F | 5.60324400 | 0.54004100 | -0.64164600 |
| C | -0.70540300 | 1.91680000 | -0.45088200 |
| C | -1.00694700 | 1.83995900 | 0.91215700 |
| C | -1.03616800 | 3.06774000 | -1.16595500 |
| C | -1.62667300 | 2.90314200 | 1.55786800 |
| H | -0.76116900 | 0.94565900 | 1.48042500 |
| C | -1.65174300 | 4.14320100 | -0.53282800 |
| H | -0.81223900 | 3.12758600 | -2.22719100 |
| C | -1.93474400 | 4.03647700 | 0.81903400 |
| H | -1.87201000 | 2.86423400 | 2.61351200 |
| H | -1.91860500 | 5.04593900 | -1.07131200 |
| F | -2.53609300 | 5.06492200 | 1.43791300 |
| H | -0.19937500 | 0.88622900 | -2.25001100 |
| H | 3.65501200 | -2.01632800 | 3.34006900 |

**$TS_4$**

E(SMD/M06-2X/6-31g(d)) = -4111.876787 au

H(SMD/M06-2X/6-31g(d)) = -4111.174695 au

G(SMD/M06-2X/6-31g(d)) = -4111.341109 au

E(SMD/M06-2X-D3/def2-TZVP//SMD/M06-2X/6-31g(d)) = -4113.602707 au

| | | | |
|---|---|---|---|
| C | 4.21072800 | 0.55575800 | -1.32610000 |
| C | 2.23797900 | 1.84710100 | 1.33211600 |
| C | 1.84618300 | 3.03667800 | 2.13171200 |
| O | 1.16601600 | 2.94558400 | 3.12434900 |
| O | 2.31846400 | 4.16476600 | 1.61845800 |

| | | | |
|---|---|---|---|
| C | 1.95866200 | 5.35565800 | 2.33658100 |
| H | 2.34932800 | 5.31230900 | 3.35508700 |
| H | 2.41406700 | 6.17683600 | 1.78636400 |
| H | 0.87291500 | 5.46296000 | 2.36107600 |
| C | 1.69883900 | 0.59572000 | 1.42087400 |
| N | 3.18699800 | 1.88775200 | 0.34517100 |
| N | 3.29411900 | 0.72115600 | -0.15074200 |
| C | 2.29582300 | -0.19403900 | 0.36854800 |
| H | 1.47176200 | -0.01565200 | -0.48942100 |
| C | 2.56812600 | -1.66074500 | 0.55058900 |
| C | 3.50315400 | -2.07130400 | 1.50583800 |
| C | 1.85676100 | -2.60211500 | -0.19024400 |
| C | 3.75179100 | -3.42757000 | 1.68771200 |
| H | 4.03107700 | -1.32900800 | 2.09974000 |
| C | 2.10103400 | -3.95987400 | 0.00516600 |
| H | 1.11257600 | -2.27548500 | -0.91127500 |
| C | 3.05421000 | -4.37196300 | 0.93320800 |
| H | 4.48306700 | -3.74823000 | 2.42305000 |
| H | 1.54823800 | -4.69323000 | -0.57409800 |
| H | 0.91318100 | 0.25732500 | 2.08247900 |
| C | 4.06641400 | -0.81115100 | -1.96402200 |
| C | 3.08340100 | -0.99684900 | -2.93811900 |
| C | 4.88628300 | -1.88240500 | -1.59625300 |
| C | 2.89172100 | -2.24526900 | -3.52335300 |
| H | 2.45111000 | -0.16399200 | -3.23138000 |
| C | 4.70607900 | -3.13513500 | -2.16738900 |
| H | 5.66491200 | -1.74502800 | -0.85204200 |
| C | 3.70382400 | -3.29077900 | -3.11554300 |
| H | 2.13217200 | -2.41277300 | -4.27920600 |
| H | 5.32065500 | -3.98439800 | -1.88882800 |
| F | 3.52234700 | -4.50288400 | -3.66481000 |
| C | 5.62773400 | 0.90412400 | -0.91216300 |
| C | 6.11232400 | 0.65298400 | 0.37218500 |
| C | 6.48114600 | 1.43893700 | -1.87892400 |
| C | 7.43732600 | 0.93229500 | 0.69461600 |
| H | 5.45715000 | 0.24052300 | 1.13493400 |
| C | 7.80798500 | 1.72123700 | -1.57381900 |
| H | 6.10595400 | 1.63585400 | -2.87960800 |
| C | 8.25852500 | 1.46092900 | -0.28841800 |
| H | 7.83327700 | 0.74875300 | 1.68749500 |
| H | 8.48602400 | 2.14168500 | -2.30850800 |
| F | 9.53823000 | 1.73598600 | 0.01666600 |
| H | 3.85605200 | 1.30687900 | -2.03876000 |
| H | 3.24807000 | -5.43040000 | 1.07814200 |
| O | 0.64978300 | 0.56847900 | -1.71224200 |
| O | -0.97816200 | -0.37565200 | -0.58446900 |

| | | | |
|---|---|---|---|
| C | -0.56662300 | 0.39506900 | -1.54362100 |
| C | -1.53275700 | 1.06330800 | -2.46079700 |
| C | -1.29579700 | 2.39182800 | -2.82678800 |
| C | -2.66271600 | 0.39259200 | -2.93282100 |
| C | -2.20894000 | 3.06693000 | -3.62708300 |
| H | -0.40846300 | 2.89982900 | -2.46167200 |
| C | -3.57075500 | 1.04946800 | -3.75471900 |
| H | -2.82262200 | -0.64747000 | -2.67421800 |
| C | -3.33158600 | 2.37918800 | -4.06934700 |
| H | -2.06849400 | 4.10510900 | -3.90783500 |
| H | -4.45487800 | 0.55401100 | -4.14038200 |
| F | -4.21608600 | 3.02659100 | -4.84258200 |
| B | -2.21894200 | -0.25910800 | 0.31425400 |
| C | -2.74125200 | 1.29123100 | 0.23356300 |
| C | -3.91721900 | 1.73618200 | -0.35841600 |
| C | -1.87578800 | 2.31007900 | 0.61775300 |
| C | -4.20954800 | 3.08024200 | -0.56493400 |
| C | -2.12536800 | 3.66115100 | 0.44793300 |
| C | -3.31176100 | 4.05155100 | -0.15442100 |
| C | -3.33502100 | -1.39364400 | -0.04475300 |
| C | -4.51047200 | -1.42083600 | 0.69918400 |
| C | -3.17080100 | -2.46222100 | -0.91474500 |
| C | -5.48099500 | -2.40051900 | 0.57690000 |
| C | -4.11801600 | -3.46855200 | -1.07364200 |
| C | -5.27845800 | -3.43998000 | -0.32032700 |
| C | -1.61686800 | -0.69171600 | 1.78276100 |
| C | -0.89626200 | -1.88370900 | 1.85341200 |
| C | -1.70718200 | 0.00804100 | 2.97984800 |
| C | -0.23053100 | -2.31759300 | 2.99076100 |
| C | -1.07341100 | -0.39978400 | 4.14980400 |
| C | -0.32213700 | -1.56430500 | 4.15272600 |
| F | -0.68158000 | 1.99362100 | 1.15374400 |
| F | -4.82841300 | 0.87221100 | -0.82945500 |
| F | -1.23261700 | 4.57706800 | 0.83525000 |
| F | -3.57691300 | 5.34157200 | -0.35066300 |
| F | -5.33964600 | 3.43863200 | -1.17850500 |
| F | -4.73930200 | -0.44125500 | 1.59076800 |
| F | -6.59514100 | -2.36389900 | 1.31210100 |
| F | -2.07451900 | -2.56598700 | -1.68689800 |
| F | -3.91061800 | -4.46579200 | -1.93816000 |
| F | -6.19511600 | -4.39815200 | -0.45645200 |
| F | -0.82044000 | -2.68216700 | 0.77759200 |
| F | -2.41466900 | 1.14385600 | 3.06720100 |
| F | -1.18669800 | 0.31727300 | 5.26976200 |
| F | 0.29267800 | -1.96550800 | 5.26448800 |
| F | 0.47474900 | -3.45071700 | 2.98324600 |

**6-H**
E(SMD/M06-2X/6-31g(d)) = -2727.409964 au
H(SMD/M06-2X/6-31g(d)) = -2727.103567 au
G(SMD/M06-2X/6-31g(d)) = -2727.212538 au
E(SMD/M06-2X-D3/def2-TZVP//SMD/M06-2X/6-31g(d)) = -2728.598586 au

| | | | |
|---|---|---|---|
| B | 0.28459800 | 0.07461200 | 0.03781100 |
| C | 0.87078100 | -0.79145000 | 1.28857300 |
| C | 2.08218200 | -0.56951900 | 1.93291500 |
| C | 0.18052900 | -1.92382900 | 1.71280700 |
| C | 2.56192300 | -1.38257700 | 2.95231600 |
| C | 0.61876700 | -2.75848200 | 2.72966700 |
| C | 1.82426600 | -2.48300400 | 3.35596700 |
| C | 0.87099500 | 1.58785100 | -0.09293900 |
| C | 1.24454400 | 2.21118900 | -1.27809400 |
| C | 0.94658500 | 2.39513400 | 1.03905100 |
| C | 1.69573700 | 3.52424300 | -1.34044400 |
| C | 1.38795500 | 3.70888300 | 1.02342500 |
| C | 1.76779600 | 4.27886400 | -0.18203200 |
| C | 0.44043700 | -0.87646500 | -1.27624700 |
| C | 1.72081200 | -1.07056500 | -1.78584900 |
| C | -0.54711300 | -1.65044800 | -1.86921500 |
| C | 2.01485600 | -1.93376500 | -2.82708100 |
| C | -0.30011200 | -2.52959100 | -2.91810000 |
| C | 0.98907800 | -2.67317700 | -3.39976000 |
| F | -0.97404700 | -2.26899700 | 1.11321800 |
| F | -0.09555100 | -3.82452300 | 3.09678800 |
| F | 2.27036600 | -3.27163100 | 4.33195200 |
| F | 3.72857500 | -1.11376000 | 3.54227200 |
| F | 2.86295500 | 0.46719400 | 1.59670400 |
| F | 0.59216400 | 1.89910000 | 2.23498000 |
| F | 1.44710900 | 4.42588500 | 2.14779700 |
| F | 2.19210500 | 5.54008300 | -0.22507700 |
| F | 2.04307900 | 4.06710200 | -2.50987700 |
| F | 1.17264000 | 1.56725700 | -2.45127300 |
| F | 2.74391300 | -0.38447400 | -1.25025600 |
| F | 3.26335300 | -2.06814200 | -3.27759900 |
| F | 1.24455000 | -3.51198700 | -4.40231300 |
| F | -1.29506600 | -3.23821100 | -3.45606400 |
| F | -1.82879600 | -1.58499300 | -1.45524400 |
| O | -1.22887900 | 0.25291800 | 0.39053800 |
| C | -2.09497200 | 0.73970400 | -0.37146200 |
| O | -1.70219800 | 1.26578400 | -1.49808000 |
| C | -3.50072300 | 0.69580900 | 0.02341900 |
| C | -3.89311600 | -0.29331900 | 0.93621700 |
| C | -4.43067100 | 1.60725100 | -0.49290000 |
| C | -5.21935200 | -0.38034500 | 1.32529800 |

| | | | |
|---|---|---|---|
| H | -3.15858400 | -0.99759900 | 1.31278200 |
| C | -5.75648700 | 1.53387300 | -0.09710200 |
| H | -4.12877200 | 2.39894900 | -1.17266000 |
| C | -6.12134400 | 0.53765200 | 0.80045300 |
| H | -5.56482500 | -1.13921100 | 2.01799400 |
| H | -6.50188500 | 2.23050400 | -0.46291900 |
| F | -7.39929300 | 0.46075400 | 1.17721000 |
| H | -2.45292600 | 1.51525100 | -2.07614700 |

**10**

E(SMD/M06-2X/6-31g(d)) = -1384.502355 au
H(SMD/M06-2X/6-31g(d)) = -1384.185106 au
G(SMD/M06-2X/6-31g(d)) = -1384.1031 au
E(SMD/M06-2X-D3/def2-TZVP//SMD/M06-2X/6-31g(d)) = -1385.045083 au

| | | | |
|---|---|---|---|
| C | -0.05438800 | 0.49586800 | -1.13497700 |
| C | -2.85724900 | -1.14120700 | 0.17811000 |
| C | -4.29866000 | -1.46161400 | 0.10938500 |
| O | -4.86031600 | -2.15262800 | 0.93093600 |
| O | -4.90533900 | -0.91155600 | -0.94407700 |
| C | -6.30434900 | -1.19243900 | -1.05294100 |
| H | -6.63886100 | -0.67799100 | -1.95259100 |
| H | -6.47151700 | -2.26794200 | -1.14498400 |
| H | -6.83835900 | -0.81514600 | -0.17791000 |
| C | -1.96198400 | -1.58952900 | 1.16721300 |
| N | -2.24723100 | -0.37616400 | -0.72400200 |
| N | -0.97310800 | -0.32802700 | -0.33421500 |
| C | -0.74631200 | -1.03944800 | 0.81526900 |
| C | 0.56875600 | -1.16209000 | 1.48736100 |
| C | 1.07582000 | -0.11821100 | 2.26614400 |
| C | 1.30276100 | -2.34520800 | 1.35498400 |
| C | 2.31649000 | -0.24799500 | 2.88587000 |
| H | 0.50112000 | 0.79605800 | 2.38189900 |
| C | 2.54319600 | -2.47155100 | 1.97410900 |
| H | 0.90415800 | -3.15607400 | 0.75178200 |
| C | 3.05408500 | -1.42067200 | 2.73550000 |
| H | 2.70582800 | 0.56789800 | 3.48735200 |
| H | 3.11261000 | -3.38888000 | 1.85834400 |
| H | -2.17149100 | -2.22036900 | 2.01837700 |
| C | 1.28169700 | -0.21514500 | -1.31121900 |
| C | 1.29212900 | -1.39030000 | -2.06783800 |
| C | 2.46420200 | 0.21918700 | -0.71166700 |
| C | 2.45536500 | -2.13756700 | -2.21426500 |
| H | 0.37233000 | -1.73335400 | -2.53602000 |
| C | 3.64070700 | -0.51255100 | -0.85004700 |
| H | 2.47270200 | 1.12388700 | -0.11237700 |
| C | 3.60900300 | -1.68082200 | -1.59449600 |
| H | 2.47991500 | -3.05347400 | -2.79479600 |

| | | | |
|---|---|---|---|
| H | 4.56625800 | -0.19657700 | -0.38063800 |
| F | 4.74047800 | -2.39703200 | -1.72488500 |
| C | 0.00910400 | 1.92689800 | -0.62198200 |
| C | -0.74757600 | 2.36098300 | 0.46646000 |
| C | 0.79544700 | 2.85505200 | -1.31455200 |
| C | -0.70464500 | 3.69168600 | 0.88053300 |
| H | -1.38675400 | 1.66592800 | 1.00329200 |
| C | 0.84873800 | 4.18558200 | -0.91748100 |
| H | 1.37590900 | 2.53345500 | -2.17532100 |
| C | 0.09631000 | 4.57670400 | 0.18081100 |
| H | -1.28539800 | 4.04233100 | 1.72689700 |
| H | 1.45570600 | 4.91468000 | -1.44342600 |
| F | 0.14400000 | 5.86277700 | 0.57383100 |
| H | -0.54179800 | 0.52192100 | -2.11513300 |
| H | 4.02307200 | -1.51890700 | 3.21567800 |

**$TS_5$**

E(SMD/M06-2X/6-31g(d)) = -2084.518207 au
H(SMD/M06-2X/6-31g(d)) = -2083.92036 au
G(SMD/M06-2X/6-31g(d)) = -2084.028626 au
E(SMD/M06-2X-D3/def2-TZVP//SMD/M06-2X/6-31g(d)) = -2085.333313 au

| | | | |
|---|---|---|---|
| C | 1.25340400 | 0.15380100 | -0.71471500 |
| C | -1.06520100 | -1.53557700 | 1.36716200 |
| C | -2.43370000 | -1.85310100 | 1.85664800 |
| O | -2.70920000 | -2.91822800 | 2.35441700 |
| O | -3.27201500 | -0.82132200 | 1.77198400 |
| C | -4.58371700 | -1.05743100 | 2.30823000 |
| H | -5.15856200 | -0.15799200 | 2.09215300 |
| H | -5.03824500 | -1.92843500 | 1.83314200 |
| H | -4.51696000 | -1.21788600 | 3.38650800 |
| C | 0.09590800 | -2.01897300 | 1.98024400 |
| N | -0.76349700 | -0.72563200 | 0.34982800 |
| N | 0.57933100 | -0.68452800 | 0.29843000 |
| C | 1.14020700 | -1.43363800 | 1.29088500 |
| C | 2.59158000 | -1.53904300 | 1.56284000 |
| C | 3.27343300 | -0.48167100 | 2.17079700 |
| C | 3.27101400 | -2.71882400 | 1.24642900 |
| C | 4.63528100 | -0.59619300 | 2.43750900 |
| H | 2.73854800 | 0.42811500 | 2.42867400 |
| C | 4.63329300 | -2.82677100 | 1.51122300 |
| H | 2.73374300 | -3.53916700 | 0.77925600 |
| C | 5.31700100 | -1.76454400 | 2.10206100 |
| H | 5.16276500 | 0.22737500 | 2.90875800 |
| H | 5.16159700 | -3.73972900 | 1.25384500 |
| H | 0.16343600 | -2.67246500 | 2.83739400 |
| C | 1.44334900 | 1.58060300 | -0.22221700 |
| C | 0.88545300 | 2.02419100 | 0.97777100 |

| | | | |
|---|---|---|---|
| C | 2.08741400 | 2.50391300 | -1.05499000 |
| C | 0.97646900 | 3.36330200 | 1.35325000 |
| H | 0.36229600 | 1.33310700 | 1.63319100 |
| C | 2.18879100 | 3.84217200 | -0.69426800 |
| H | 2.51006300 | 2.17666900 | -2.00121500 |
| C | 1.62236200 | 4.24743100 | 0.50589000 |
| H | 0.54289600 | 3.72374900 | 2.28011000 |
| H | 2.68478700 | 4.56794600 | -1.32982900 |
| F | 1.70199100 | 5.54220800 | 0.85616500 |
| C | 2.48226600 | -0.56392800 | -1.25988900 |
| C | 3.78937400 | -0.14196300 | -1.01621900 |
| C | 2.26596700 | -1.72579800 | -2.00557800 |
| C | 4.87032700 | -0.87709700 | -1.49371700 |
| H | 3.97650200 | 0.75256000 | -0.43158600 |
| C | 3.33232300 | -2.47727200 | -2.48559600 |
| H | 1.24737800 | -2.05672900 | -2.19880200 |
| C | 4.61837800 | -2.03455600 | -2.21332400 |
| H | 5.89383400 | -0.57205600 | -1.30401500 |
| H | 3.18307900 | -3.38477700 | -3.06045800 |
| F | 5.65878500 | -2.75289100 | -2.66850200 |
| H | 6.37986200 | -1.85049600 | 2.30669200 |
| H | 0.54302500 | 0.19463800 | -1.54443500 |
| C | -2.07360900 | 0.08741000 | -1.24404100 |
| H | -1.32353700 | -0.26769300 | -1.94622300 |
| C | -3.23553000 | -0.76053900 | -1.14208100 |
| C | -4.52799400 | -0.23295900 | -0.95350600 |
| C | -3.07460300 | -2.14826300 | -1.33724300 |
| C | -5.63083700 | -1.07135500 | -0.94528800 |
| H | -4.67589200 | 0.83741800 | -0.86273600 |
| C | -4.16295500 | -2.99824100 | -1.29066200 |
| H | -2.07774600 | -2.55119800 | -1.49525000 |
| C | -5.42343400 | -2.43695900 | -1.09731400 |
| H | -6.63897600 | -0.69085200 | -0.82503000 |
| H | -4.06446700 | -4.07075600 | -1.41445900 |
| C | -2.02719300 | 1.49332000 | -0.92742200 |
| C | -1.26382900 | 2.33812500 | -1.75450200 |
| C | -2.67047600 | 2.02517200 | 0.20898000 |
| C | -1.18037000 | 3.69503000 | -1.49095400 |
| H | -0.74955600 | 1.92229100 | -2.61701600 |
| C | -2.56300400 | 3.37212600 | 0.50222100 |
| H | -3.19744500 | 1.36155300 | 0.88735200 |
| C | -1.81945400 | 4.17834500 | -0.35672000 |
| H | -0.60830900 | 4.37015800 | -2.11711700 |
| H | -3.01811900 | 3.80676000 | 1.38510700 |
| F | -1.69191200 | 5.47230600 | -0.05993600 |
| F | -6.48033800 | -3.24785000 | -1.07265900 |

**11**

E(SMD/M06-2X/6-31g(d)) = -2084.548525 au
H(SMD/M06-2X/6-31g(d)) = -2083.947738 au
G(SMD/M06-2X/6-31g(d)) = -2084.056168 au
E(SMD/M06-2X-D3/def2-TZVP//SMD/M06-2X/6-31g(d)) = -2085.364535 au

| | | | |
|---|---|---|---|
| C | 1.23885900 | 0.38947800 | -0.67574300 |
| C | -1.05649900 | -1.56997800 | 1.26960800 |
| C | -2.43604500 | -1.84243100 | 1.78828300 |
| O | -2.82238200 | -2.95146700 | 2.04827900 |
| O | -3.08950700 | -0.71052100 | 2.00649500 |
| C | -4.41929200 | -0.85576900 | 2.53772400 |
| H | -4.84542500 | 0.14573900 | 2.52981900 |
| H | -5.00120900 | -1.53156100 | 1.90906000 |
| H | -4.36557800 | -1.24432000 | 3.55646900 |
| C | 0.10689300 | -2.08976500 | 1.79295700 |
| N | -0.74935600 | -0.70640800 | 0.28237400 |
| N | 0.61284800 | -0.65747300 | 0.17526000 |
| C | 1.15732200 | -1.48522900 | 1.09901700 |
| C | 2.58814000 | -1.75945900 | 1.34706900 |
| C | 3.53946500 | -0.76636600 | 1.59646300 |
| C | 2.96190600 | -3.10851400 | 1.39694100 |
| C | 4.85666800 | -1.12282000 | 1.86550600 |
| H | 3.25625200 | 0.28105200 | 1.58549500 |
| C | 4.28171200 | -3.45747700 | 1.66244400 |
| H | 2.22031400 | -3.87988300 | 1.21052900 |
| C | 5.23141600 | -2.46485800 | 1.89371400 |
| H | 5.59109000 | -0.34645700 | 2.05456400 |
| H | 4.56589400 | -4.50450000 | 1.68665600 |
| H | 0.19549600 | -2.78188100 | 2.61721900 |
| C | 1.24990700 | 1.73049800 | 0.04346400 |
| C | 1.17911500 | 1.86384200 | 1.43031300 |
| C | 1.28284900 | 2.88019000 | -0.75297600 |
| C | 1.16602400 | 3.12544700 | 2.02191500 |
| H | 1.11215400 | 0.99211200 | 2.07619800 |
| C | 1.27181600 | 4.14562100 | -0.17986900 |
| H | 1.31318200 | 2.78350400 | -1.83567300 |
| C | 1.21487800 | 4.24105200 | 1.20376200 |
| H | 1.10747100 | 3.24704400 | 3.09795100 |
| H | 1.29163000 | 5.04639900 | -0.78374300 |
| F | 1.19488100 | 5.45945800 | 1.76708100 |
| C | 2.56065500 | -0.09717200 | -1.25613300 |
| C | 3.71395600 | 0.68316600 | -1.21322700 |
| C | 2.59388500 | -1.34254400 | -1.89077400 |
| C | 4.90098000 | 0.22010200 | -1.77549800 |
| H | 3.70335700 | 1.65303700 | -0.72659600 |
| C | 3.77236100 | -1.82530000 | -2.44398300 |

| | | | |
|---|---|---|---|
| H | 1.69413900 | -1.95253400 | -1.93805900 |
| C | 4.90774000 | -1.02974500 | -2.37087700 |
| H | 5.81109500 | 0.80912900 | -1.74645100 |
| H | 3.82210200 | -2.79442400 | -2.92811500 |
| F | 6.05164300 | -1.48933100 | -2.90286100 |
| H | 6.26201100 | -2.73596800 | 2.10041700 |
| H | 0.56932100 | 0.50122300 | -1.53189900 |
| C | -1.62902000 | -0.22399800 | -0.83356200 |
| H | -1.04422800 | -0.42583300 | -1.73565600 |
| C | -2.86806900 | -1.10111900 | -0.96268800 |
| C | -4.15486700 | -0.59182600 | -0.79035500 |
| C | -2.69882700 | -2.44240700 | -1.32521200 |
| C | -5.26606000 | -1.41179700 | -0.96826100 |
| H | -4.29804400 | 0.44750600 | -0.51087100 |
| C | -3.79578100 | -3.27605400 | -1.49713100 |
| H | -1.69616400 | -2.84026600 | -1.46946300 |
| C | -5.06214800 | -2.73839800 | -1.31369900 |
| H | -6.27585800 | -1.03701500 | -0.84039800 |
| H | -3.68605100 | -4.31876600 | -1.77392900 |
| C | -1.88291700 | 1.26521100 | -0.74596900 |
| C | -1.99718900 | 1.98083500 | -1.93964700 |
| C | -2.01088900 | 1.92961400 | 0.47466500 |
| C | -2.22831100 | 3.35219500 | -1.92491600 |
| H | -1.89781500 | 1.46348900 | -2.89058800 |
| C | -2.23361900 | 3.30299600 | 0.50661900 |
| H | -1.91964700 | 1.38203700 | 1.40783500 |
| C | -2.33596900 | 3.98570200 | -0.69619800 |
| H | -2.31320000 | 3.92843400 | -2.83974400 |
| H | -2.32028600 | 3.84485100 | 1.44236900 |
| F | -2.53986800 | 5.31268000 | -0.66940900 |
| F | -6.12972400 | -3.53243000 | -1.48453400 |

**TS6**

E(SMD/M06-2X/6-31g(d)) = -2084.514934 au
H(SMD/M06-2X/6-31g(d)) = -2083.91729 au
G(SMD/M06-2X/6-31g(d)) = -2084.024907 au
E(SMD/M06-2X-D3/def2-TZVP//SMD/M06-2X/6-31g(d)) = -2085.330414 au

| | | | |
|---|---|---|---|
| C | -1.49373300 | 0.80663300 | 1.31138900 |
| C | 0.77962800 | -1.72799900 | -1.36947000 |
| C | 2.09608100 | -2.21702600 | -1.86334000 |
| O | 2.28267900 | -3.36198300 | -2.19365300 |
| O | 2.98444400 | -1.23403200 | -1.96285600 |
| C | 4.28391100 | -1.62006500 | -2.43356600 |
| H | 4.88498000 | -0.71240100 | -2.40466300 |
| H | 4.70860300 | -2.38682000 | -1.78156700 |
| H | 4.21229900 | -2.00072400 | -3.45434200 |
| C | -0.45177200 | -2.10723100 | -1.84578300 |

| | | | |
|---|---|---|---|
| N | 0.58240800 | -0.79369100 | -0.40343700 |
| N | -0.73830400 | -0.55799400 | -0.24250100 |
| C | -1.38013200 | -1.33739000 | -1.12292600 |
| C | -2.84566300 | -1.41095900 | -1.31702000 |
| C | -3.60544800 | -0.29475700 | -1.67302300 |
| C | -3.46472100 | -2.66230000 | -1.21474100 |
| C | -4.97412700 | -0.42340000 | -1.89312900 |
| H | -3.12098600 | 0.66995700 | -1.79122900 |
| C | -4.83322200 | -2.78791500 | -1.43431700 |
| H | -2.87038000 | -3.53252300 | -0.94957300 |
| C | -5.59188000 | -1.66698000 | -1.76785200 |
| H | -5.55887500 | 0.45068300 | -2.16433700 |
| H | -5.30687200 | -3.76051500 | -1.34328400 |
| H | -0.65834100 | -2.81036300 | -2.63946200 |
| C | -0.85829000 | 2.04457200 | 0.91014500 |
| C | -1.21054500 | 2.71800600 | -0.27518800 |
| C | 0.14584500 | 2.57450100 | 1.73741600 |
| C | -0.57490800 | 3.89463800 | -0.62588900 |
| H | -1.95108500 | 2.28628100 | -0.94094900 |
| C | 0.76238100 | 3.77421800 | 1.41700100 |
| H | 0.43234600 | 2.04457200 | 2.64229700 |
| C | 0.40257400 | 4.39592400 | 0.23007300 |
| H | -0.79868100 | 4.41687400 | -1.54949600 |
| H | 1.53502000 | 4.20873600 | 2.04093600 |
| F | 1.04397700 | 5.51188500 | -0.12392700 |
| C | -2.91393500 | 0.57401100 | 1.29083100 |
| C | -3.83757500 | 1.59688100 | 0.99299500 |
| C | -3.38924300 | -0.69904000 | 1.67206200 |
| C | -5.19743600 | 1.34564600 | 1.03974500 |
| H | -3.49379300 | 2.59966000 | 0.76757100 |
| C | -4.74281900 | -0.96938000 | 1.69463600 |
| H | -2.67429600 | -1.48147500 | 1.91358500 |
| C | -5.61931300 | 0.06119500 | 1.36353900 |
| H | -5.93092100 | 2.11536400 | 0.82836400 |
| H | -5.13481800 | -1.94650800 | 1.95329700 |
| F | -6.92634700 | -0.19519400 | 1.37136700 |
| H | -6.65998900 | -1.76255200 | -1.93794100 |
| H | -0.95201200 | 0.20657600 | 2.03900000 |
| C | 1.58064300 | -0.29660200 | 0.56957700 |
| H | 0.98091900 | 0.00858000 | 1.42934700 |
| C | 2.42921600 | -1.47347900 | 1.05271800 |
| C | 3.80521700 | -1.57063900 | 0.84773600 |
| C | 1.76496600 | -2.51232200 | 1.71535900 |
| C | 4.51274400 | -2.68494600 | 1.29483300 |
| H | 4.33861400 | -0.78018000 | 0.32989100 |
| C | 2.45192700 | -3.63224400 | 2.16455200 |

| | | | |
|---|---|---|---|
| H | 0.69006500 | -2.44761400 | 1.87386600 |
| C | 3.82057400 | -3.69399500 | 1.94263900 |
| H | 5.58272500 | -2.77672700 | 1.14173300 |
| H | 1.95017900 | -4.44571200 | 2.67699900 |
| C | 2.32314600 | 0.93726500 | 0.08405200 |
| C | 3.26979000 | 1.52472900 | 0.93176100 |
| C | 2.01691100 | 1.55934200 | -1.12505400 |
| C | 3.91462500 | 2.70358800 | 0.57513600 |
| H | 3.50335100 | 1.06211000 | 1.88717000 |
| C | 2.65034100 | 2.74345200 | -1.49635500 |
| H | 1.27772600 | 1.12532100 | -1.79168500 |
| C | 3.58386700 | 3.29512600 | -0.63520700 |
| H | 4.65003800 | 3.16995300 | 1.22192700 |
| H | 2.41872900 | 3.24334300 | -2.43115400 |
| F | 4.19030100 | 4.44325000 | -0.98387700 |
| F | 4.49673800 | -4.77109300 | 2.37369500 |

**3**

E(SMD/M06-2X/6-31g(d)) = -1384.503951 au
H(SMD/M06-2X/6-31g(d)) = -1384.104474 au
G(SMD/M06-2X/6-31g(d)) = -1384.190000 au
E(SMD/M06-2X-D3/def2-TZVP//SMD/M06-2X/6-31g(d)) = -1385.045494 au

| | | | |
|---|---|---|---|
| C | 1.09722100 | 0.39160300 | -0.71377500 |
| C | -0.16502400 | -1.80468400 | -1.10369100 |
| C | 0.88068600 | -2.63293300 | -1.74049700 |
| O | 0.68044600 | -3.77697900 | -2.07856200 |
| O | 2.04903500 | -2.00552500 | -1.88583600 |
| C | 3.10452900 | -2.78925600 | -2.45804800 |
| H | 3.97455300 | -2.13486000 | -2.47931700 |
| H | 3.30016100 | -3.66587500 | -1.83744000 |
| H | 2.83624000 | -3.10464500 | -3.46828800 |
| C | -1.43313900 | -2.24230200 | -0.77516800 |
| N | -0.07666100 | -0.50414300 | -0.69557500 |
| N | -1.20214900 | -0.10150400 | -0.11241700 |
| C | -2.04675100 | -1.13749200 | -0.15574000 |
| C | -3.40750400 | -1.01870400 | 0.39740100 |
| C | -3.85529100 | 0.19913800 | 0.92436300 |
| C | -4.26914200 | -2.12069500 | 0.40483100 |
| C | -5.13898700 | 0.30855200 | 1.44781300 |
| H | -3.18996800 | 1.05717500 | 0.91831400 |
| C | -5.55370400 | -2.00806100 | 0.92891800 |
| H | -3.93427700 | -3.07191500 | 0.00086200 |
| C | -5.99343300 | -0.79384200 | 1.45243100 |
| H | -5.47451300 | 1.25872000 | 1.85266400 |
| H | -6.21153400 | -2.87204900 | 0.92830800 |
| H | -1.83459600 | -3.22528300 | -0.97334800 |
| C | 0.64321400 | 1.84243200 | -0.70440500 |

| | | | |
|---|---|---|---|
| C | 0.60483900 | 2.60318500 | 0.46420300 |
| C | 0.23910400 | 2.42160700 | -1.90998200 |
| C | 0.16889600 | 3.92517200 | 0.43658400 |
| H | 0.91812900 | 2.16755200 | 1.40798200 |
| C | -0.20055200 | 3.73992800 | -1.95800300 |
| H | 0.26595700 | 1.83251400 | -2.82323200 |
| C | -0.22575100 | 4.46562200 | -0.77660900 |
| H | 0.13381500 | 4.53332600 | 1.33405600 |
| H | -0.51476400 | 4.20678300 | -2.88529000 |
| F | -0.64371000 | 5.74416900 | -0.81127200 |
| C | 2.05871400 | 0.03211900 | 0.40453900 |
| C | 3.42281200 | 0.27129600 | 0.22265600 |
| C | 1.61207800 | -0.50269500 | 1.61364500 |
| C | 4.33630300 | -0.01259300 | 1.23196100 |
| H | 3.77460900 | 0.68179000 | -0.72066900 |
| C | 2.51330600 | -0.79458000 | 2.63531900 |
| H | 0.55312400 | -0.69454100 | 1.76623800 |
| C | 3.85831400 | -0.54170300 | 2.42185500 |
| H | 5.39977500 | 0.16182600 | 1.10810200 |
| H | 2.18672400 | -1.21241100 | 3.58152800 |
| F | 4.73557300 | -0.82486700 | 3.40261400 |
| H | -6.99568200 | -0.70632300 | 1.86073400 |
| H | 1.59687800 | 0.20737400 | -1.66575500 |

**TS$_A$**

E(SMD/M06-2X/6-31g(d)) = -684.7670497 au
H(SMD/M06-2X/6-31g(d)) = -684.560363 au
G(SMD/M06-2X/6-31g(d)) = -684.614722 au
E(SMD/M06-2X-D3/def2-TZVP//SMD/M06-2X/6-31g(d)) = -685.0353071 au

| | | | |
|---|---|---|---|
| C | -1.69285900 | 0.58615700 | 0.03077400 |
| N | -1.48475900 | 1.87932200 | -0.38224200 |
| C | -0.49493800 | -0.02182700 | 0.39389200 |
| C | -3.05673400 | 0.05110400 | -0.01848100 |
| O | -4.03253900 | 0.69261100 | -0.33848900 |
| O | -3.07522600 | -1.24424000 | 0.31248800 |
| H | -0.50261900 | -1.08498300 | 0.61812900 |
| C | 0.68169700 | 0.72531000 | 0.35056600 |
| H | 0.67252500 | 1.69491000 | 0.85808000 |
| C | 2.01359000 | 0.14834800 | 0.16281800 |
| C | 2.19475500 | -1.08364900 | -0.48690500 |
| C | 3.14608200 | 0.84311500 | 0.61454200 |
| C | 3.46859200 | -1.61470200 | -0.65179300 |
| H | 1.33089000 | -1.61850000 | -0.87266100 |
| C | 4.42035300 | 0.31505400 | 0.43878400 |
| H | 3.01667800 | 1.80196300 | 1.11011500 |
| C | 4.58614800 | -0.91806900 | -0.19067100 |
| H | 3.59212200 | -2.57048300 | -1.15224000 |

| | | | |
|---|---|---|---|
| H | 5.28570500 | 0.86517100 | 0.79606800 |
| H | 5.58073800 | -1.33139200 | -0.32745100 |
| N | -0.44641400 | 2.37554000 | -0.60814200 |
| C | -4.36302400 | -1.86842300 | 0.25330300 |
| H | -4.20232000 | -2.90228300 | 0.55468200 |
| H | -5.05562700 | -1.37480000 | 0.93824800 |
| H | -4.75974800 | -1.82510500 | -0.76325000 |

**TS$_B$**

E(SMD/M06-2X/6-31g(d)) = -2892.306864 au
H(SMD/M06-2X/6-31g(d)) = -2891.911032 au
G(SMD/M06-2X/6-31g(d)) = -2892.031051 au
E(SMD/M06-2X-D3/def2-TZVP//SMD/M06-2X/6-31g(d)) = -2893.543835 au

| | | | |
|---|---|---|---|
| C | 2.69203900 | -0.46375300 | 0.50257400 |
| N | 2.69897200 | -0.44580000 | 1.89694900 |
| C | 3.97055400 | -0.74931400 | 0.01392600 |
| C | 1.42408600 | -0.21875200 | -0.12539900 |
| O | 0.38401300 | -0.35093400 | 0.58183600 |
| O | 1.26519400 | 0.15782400 | -1.35601100 |
| H | 4.17093300 | -0.73271500 | -1.05114600 |
| C | 4.99422500 | -1.00784700 | 0.92198800 |
| H | 4.79458100 | -1.76100400 | 1.69124600 |
| C | 6.41054400 | -0.78912700 | 0.62862800 |
| C | 6.81815700 | 0.18063600 | -0.30132200 |
| C | 7.38661700 | -1.53971300 | 1.30034700 |
| C | 8.16755700 | 0.37078500 | -0.57253700 |
| H | 6.07186500 | 0.79334800 | -0.80089200 |
| C | 8.73645400 | -1.34056900 | 1.03372100 |
| H | 7.07648400 | -2.28456100 | 2.02844600 |
| C | 9.13004600 | -0.38861900 | 0.09376700 |
| H | 8.47162600 | 1.12104300 | -1.29589900 |
| H | 9.48271000 | -1.92975100 | 1.55764900 |
| H | 10.18416500 | -0.23304100 | -0.11430400 |
| N | 3.65987400 | -0.43333900 | 2.55933500 |
| C | 2.32342100 | 0.61125900 | -2.22374300 |
| H | 3.03212900 | 1.22871100 | -1.66983200 |
| H | 2.80753900 | -0.25006800 | -2.68546500 |
| H | 1.81427500 | 1.20337400 | -2.98207200 |
| B | -1.03842800 | 0.05017000 | 0.08740200 |
| C | -1.48734000 | -0.86210700 | -1.18686700 |
| C | -0.86137300 | -2.01478200 | -1.64066800 |
| C | -2.69046300 | -0.56125300 | -1.81657300 |
| C | -1.36993400 | -2.80239800 | -2.66770600 |
| C | -3.23486500 | -1.31495700 | -2.84270800 |
| C | -2.56421800 | -2.45129100 | -3.27259300 |
| C | -0.93714300 | 1.65533700 | -0.18837500 |
| C | -0.78256600 | 2.53446800 | 0.88047500 |

| | | | |
|---|---|---|---|
| C | -0.86508600 | 2.25023500 | -1.44321800 |
| C | -0.60645200 | 3.90237800 | 0.73723300 |
| C | -0.68550400 | 3.61496300 | -1.63435700 |
| C | -0.55865400 | 4.44835500 | -0.53605900 |
| C | -2.02827900 | -0.39018400 | 1.30615700 |
| C | -1.90897800 | -1.67028100 | 1.84154200 |
| C | -3.09176000 | 0.35487700 | 1.80219700 |
| C | -2.74146800 | -2.17174200 | 2.83094200 |
| C | -3.95136100 | -0.10984300 | 2.78981700 |
| C | -3.77302800 | -1.38040800 | 3.31127900 |
| F | -0.93507600 | 1.51513600 | -2.56373400 |
| F | -0.79972500 | 2.06684100 | 2.13840500 |
| F | -0.47299800 | 4.69147300 | 1.80552700 |
| F | -0.38205500 | 5.75786300 | -0.70051500 |
| F | -0.61877000 | 4.12439700 | -2.86709000 |
| F | 0.29913600 | -2.43570900 | -1.10384000 |
| F | -3.37789200 | 0.52454600 | -1.42255500 |
| F | -4.39035700 | -0.96761100 | -3.41348600 |
| F | -3.06619600 | -3.19619700 | -4.25689900 |
| F | -0.71703800 | -3.89588000 | -3.06983100 |
| F | -0.96142700 | -2.50276800 | 1.37981900 |
| F | -3.34680500 | 1.58807700 | 1.34060600 |
| F | -4.94993600 | 0.65610700 | 3.23601300 |
| F | -4.58961900 | -1.84050500 | 4.25792100 |
| F | -2.57025200 | -3.40637500 | 3.30964300 |

**TS$_C$**

E(SMD/M06-2X/6-31g(d)) = -2892.314998 au

H(SMD/M06-2X/6-31g(d)) = -2891.918794 au

G(SMD/M06-2X/6-31g(d)) = -2892.038816 au

E(SMD/M06-2X-D3/def2-TZVP//SMD/M06-2X/6-31g(d)) = -2893.548708 au

| | | | |
|---|---|---|---|
| C | 0.29827100 | 3.10846400 | 0.93605600 |
| C | -0.54696900 | 4.32145300 | 1.16599600 |
| O | -0.06599100 | 5.41041300 | 1.36785900 |
| O | -1.83976600 | 4.04653200 | 1.09498500 |
| C | -2.71611300 | 5.17150300 | 1.27345100 |
| H | -3.72422300 | 4.76984800 | 1.19334200 |
| H | -2.55581000 | 5.61688500 | 2.25719100 |
| H | -2.53357800 | 5.91459000 | 0.49475700 |
| C | 1.67714500 | 3.16356100 | 1.00360100 |
| N | -0.30412600 | 1.93265300 | 0.64822500 |
| N | 0.22608800 | 1.06644700 | -0.03060600 |
| C | 2.45647400 | 2.02670300 | 0.78961000 |
| H | 2.07271500 | 1.07464700 | 1.14656600 |
| C | 3.80660900 | 2.02175000 | 0.29757600 |
| C | 4.40313900 | 3.16948500 | -0.26588600 |
| C | 4.54323200 | 0.82150300 | 0.36430700 |

| | | | |
|---|---|---|---|
| C | 5.70524700 | 3.11437200 | -0.73271300 |
| H | 3.83876700 | 4.09373600 | -0.34220200 |
| C | 5.84619700 | 0.77318100 | -0.10724200 |
| H | 4.08469000 | -0.06272900 | 0.79482600 |
| C | 6.42647800 | 1.91832400 | -0.65410000 |
| H | 6.16382600 | 3.99711400 | -1.16564500 |
| H | 6.40869600 | -0.15287500 | -0.05177300 |
| H | 2.12317100 | 4.14228900 | 1.16068700 |
| H | 7.44581600 | 1.88087400 | -1.02602400 |
| B | -0.46458200 | -0.37935200 | -0.10281200 |
| C | -1.65416700 | 0.02193800 | -1.15563000 |
| C | -2.99725300 | 0.17891800 | -0.82940600 |
| C | -1.32252300 | 0.39630700 | -2.45666700 |
| C | -3.95367100 | 0.61873100 | -1.73764700 |
| C | -2.24306600 | 0.83941500 | -3.39207400 |
| C | -3.57583900 | 0.95269700 | -3.02655000 |
| C | -0.95512400 | -0.94472400 | 1.34231000 |
| C | -0.49905000 | -0.53889400 | 2.58895800 |
| C | -1.80559300 | -2.04682700 | 1.36147700 |
| C | -0.88580900 | -1.14783700 | 3.77803500 |
| C | -2.21941700 | -2.68329500 | 2.51877000 |
| C | -1.75374000 | -2.22469700 | 3.74341000 |
| C | 0.62460100 | -1.45054700 | -0.68398800 |
| C | 0.47594100 | -2.27485700 | -1.79534300 |
| C | 1.80093700 | -1.65894000 | 0.02629000 |
| C | 1.44727800 | -3.17965000 | -2.20796400 |
| C | 2.80397800 | -2.53683200 | -0.35097400 |
| C | 2.62473900 | -3.30658100 | -1.48896700 |
| F | 0.37794200 | 0.47544900 | 2.71736500 |
| F | 2.00645100 | -0.98614800 | 1.17960200 |
| F | -0.41644500 | -0.70797000 | 4.94783000 |
| F | -2.13333800 | -2.82140900 | 4.87203900 |
| F | -3.04967200 | -3.72702400 | 2.47150600 |
| F | -2.26922900 | -2.53236900 | 0.19819400 |
| F | 3.92173900 | -2.64696900 | 0.37149500 |
| F | -0.63709400 | -2.24433300 | -2.54017900 |
| F | 1.25208300 | -3.93147500 | -3.29255100 |
| F | 3.56405200 | -4.16507600 | -1.87833900 |
| F | -3.45454500 | -0.08663700 | 0.40199200 |
| F | -5.23150600 | 0.73345400 | -1.36984800 |
| F | -4.48016200 | 1.38203800 | -3.90369400 |
| F | -1.85937800 | 1.17157400 | -4.62636200 |
| F | -0.04427700 | 0.33416000 | -2.86037800 |

# 6. References


[1] M. Santi, D. M. C. Ould, J. Wenz, Y. Soltani, R. L. Melen, T. Wirth, *Angew. Chem. Int. Ed*. **2019**, *58*, 7861–7865.

[2] R. K. Harris, E. D. Becker, S. M. Cabral De Menezes, R. Goodfellow, P. Granger, *Solid State Nucl Magn Reson*. **2002**, *4*, 458–483.

[3] D. Drikermann, R. S. Mößel, W. K. Al-Jammal, I. Vilotijevic, *Org. Lett.* **2020**, *22*, 1091−1095.

[4] A. Dasgupta, K. Stefkova, R. Babaahmadi, B.F. Yates, N. J. Buurma, A. Ariafard, E. Richards, R. L. Melen, *J. Am. Chem. Soc.* **2021**, *143*, 4451–4464.

[5] CrysAlisPro, Agilent Technoligies, Version 1.171.37.33 (release 27-03-2014 CrysAlis171 .NET).

[6] SHELXL-2013, G.M. SHeldrick, University of Göttingen, Germany (2013).

[7] Gaussian 16, Revision B.01, M. J. Frisch, G. W. Trucks, H. B. Schlegel, G. E. Scuseria, M. A. Robb, J. R. Cheeseman, G. Scalmani, V. Barone, G. A. Petersson, H. Nakatsuji, X. Li, M. Caricato, A. V. Marenich, J. Bloino, B. G. Janesko, R. Gomperts, B. Mennucci, H. P. Hratchian, J. V. Ortiz, A. F. Izmaylov, J. L. Sonnenberg, D. Williams-Young, F. Ding, F. Lipparini, F. Egidi, J. Goings, B. Peng, A. Petrone, T. Henderson, D. Ranasinghe, V. G. Zakrzewski, J. Gao, N. Rega, G. Zheng, W. Liang, M. Hada, M. Ehara, K. Toyota, R. Fukuda, J. Hasegawa, M. Ishida, T. Nakajima, Y. Honda, O. Kitao, H. Nakai, T. Vreven, K. Throssell, J. A. Montgomery, Jr., J. E. Peralta, F. Ogliaro, M. J. Bearpark, J. J. Heyd, E. N. Brothers, K. N. Kudin, V. N. Staroverov, T. A. Keith, R. Kobayashi, J. Normand, K. Raghavachari, A. P. Rendell, J. C. Burant, S. S. Iyengar, J. Tomasi, M. Cossi, J. M. Millam, M. Klene, C. Adamo, R. Cammi, J. W. Ochterski, R. L. Martin, K. Morokuma, O. Farkas, J. B. Foresman, and D. J. Fox, Gaussian, Inc., Wallingford CT, **2016**.

[8] Y. Zhao, D. G. Truhlar, *Acc. Chem. Res.* **2008**, *41*, 157−167.

[9] A. V. Marenich, C. J. Cramer, D. G. J. Truhlar, *Phys. Chem. B.* **2009**, *113*, 6378−6396.

[10] P. C. Hariharan, J. A. Pople, *Theor. Chim. Acta J. A.* **1973**, *28*, 213−222.

[11] (a) K. J. Fukui, *Phys. Chem.* **1970**, *74*, 4161−4163. (b) K. Fukui, *Acc. Chem. Res.* **1981**, *14*, 363−368.

[12] S. Grimme, J. Antony, S. Ehrlich, H. J. Krieg, *Chem. Phys.* **2010**, *132*, 154104.

[13] F. Weigend, F. Furche and R. J. Ahlrichs, *Chem. Phys.* **2003**, *119*, 12753–12762.

[14] J. W. Ochterski, Thermochemistry, Gaussian, Inc., Wallingford, CT, 2000.
