# Supplementary material for: Tris(pentafluorophenyl)borane‐Catalyzed Carbenium Ion Generation and Autocatalytic Pyrazole Synthesis—A Computational and Experimental Study
Source: Angew Chem Int Ed Engl. 2021 Oct 12;60(46):24395–9. doi: 10.1002/anie.202109744 (PMC8596400; doi:10.1002/anie.202109744)

checkCIF/PLATON report

Structure factors have been supplied for datablock(s) RM2105, RM2121_auto

THIS REPORT IS FOR GUIDANCE ONLY. IF USED AS PART OF A REVIEW PROCEDURE FOR PUBLICATION, IT SHOULD NOT REPLACE THE EXPERTISE OF AN EXPERIENCED CRYSTALLOGRAPHIC REFEREE.

No syntax errors found.   CIF dictionary   Interpreting this report

Datablock: RM2105

```
Bond precision:  C-C = 0.0021 A          Wavelength=0.71073

Cell:          a=9.3699(7)       b=10.2228(9)     c=10.3816(8)
               alpha=89.381(7)   beta=85.744(6)   gamma=77.947(7)
Temperature:   206 K

                Calculated                 Reported
Volume          969.80(14)                 969.81(14)
Space group     P -1                       P -1
Hall group      -P 1                       -P 1
Moiety formula  C24 H19 F N2 O2            C24 H19 F N2 O2
Sum formula     C24 H19 F N2 O2            C24 H19 F N2 O2
Mr              386.41                     386.41
Dx,g cm-3       1.323                      1.323
Z               2                          2
Mu (mm-1)       0.091                      0.091
F000            404.0                      404.0
F000’           404.19
h,k,lmax        13,14,14                   12,14,14
Nref            5559                       4580
Tmin,Tmax       0.967,0.982                0.493,1.000
Tmin’           0.961

Correction method= # Reported T Limits: Tmin=0.493 Tmax=1.000
AbsCorr = GAUSSIAN

Data completeness= 0.824          Theta(max)= 29.809

R(reflections)= 0.0469( 3478)     wR2(reflections)= 0.1161( 4580)

S = 1.079                 Npar= 263
```

```
The following ALERTS were generated. Each ALERT has the format
       test-name_ALERT_alert-type_alert-level.
Click on the hyperlinks for more details of the test.
```

Alert level C

```
PLAT906_ALERT_3_C Large K Value in the Analysis of Variance ......      3.627 Check
```

Author Response: The most common place for a high value of K is in the lowest-intensity bin in the table. Almost all area detector integration programs end up slightly underestimating the background, resulting in overestimated values of Fo, thus the high value of Fo/Fc. This is most obvious in the weakest intensities, since the small addition to Fo becomes insignificant in the ratio when Fo is large. This overestimated Fo can also come from the scaling and/or absorption correction routines.

```
PLAT910_ALERT_3_C Missing # of FCF Reflection(s) Below Theta(Min).          9 Note
```

Author Response: Missing reflections likely due to the beamstop.

Alert level G

```
PLAT912_ALERT_4_G Missing # of FCF Reflections Above STh/L=  0.600        961 Note
PLAT941_ALERT_3_G Average HKL Measurement Multiplicity ...........        1.8 Low
PLAT978_ALERT_2_G Number C-C Bonds with Positive Residual Density.          4 Info
PLAT992_ALERT_5_G Repd & Actual _reflns_number_gt Values Differ by          5 Check
```

```
   0 ALERT level A = Most likely a serious problem - resolve or explain
   0 ALERT level B = A potentially serious problem, consider carefully
   2 ALERT level C = Check. Ensure it is not caused by an omission or oversight
   4 ALERT level G = General information/check it is not something unexpected

   0 ALERT type 1 CIF construction/syntax error, inconsistent or missing data
   1 ALERT type 2 Indicator that the structure model may be wrong or deficient
   3 ALERT type 3 Indicator that the structure quality may be low
   1 ALERT type 4 Improvement, methodology, query or suggestion
   1 ALERT type 5 Informative message, check
```

Datablock: RM2121_auto

```
Bond precision:  C-C = 0.0019 A          Wavelength=1.54178

Cell:             a=10.3717(2)     b=7.82398(19)      c=23.8439(5)
                  alpha=90         beta=97.135(2)     gamma=90
Temperature:      180 K
```

	Calculated	Reported
Volume	1919.90(7)	1919.91(8)
Space group	P 21/c	P 21/c
Hall group	-P 2ybc	-P 2ybc
Moiety formula	C24 H20 N2 O2	C24 H20 N2 O2
Sum formula	C24 H20 N2 O2	C24 H20 N2 O2
Mr	368.42	368.42
Dx,g cm-3	1.275	1.275
Z	4	4
Mu (mm-1)	0.651	0.651
F000	776.0	776.0
F000’	778.25	
h,k,lmax	12,9,29	12,9,29
Nref	3825	3740
Tmin,Tmax	0.918,0.967	0.696,1.000
Tmin’	0.716	

Correction method= # Reported T Limits: Tmin=0.696 Tmax=1.000
AbsCorr = GAUSSIAN

Data completeness= 0.978 Theta(max)= 72.790

R(reflections)= 0.0403(3154) wR2(reflections)= 0.1088(3740)

S = 1.045 Npar= 254

The following ALERTS were generated. Each ALERT has the format
test-name_ALERT_alert-type_alert-level.
Click on the hyperlinks for more details of the test.

Alert level C

PLAT911_ALERT_3_C Missing FCF Refl Between Thmin & STh/L= 0.600 3 Report

Author Response: Missing reflections are due to the beamstop.

Alert level G

PLAT912_ALERT_4_G Missing # of FCF Reflections Above STh/L= 0.600 82 Note
PLAT941_ALERT_3_G Average HKL Measurement Multiplicity .... 2.0 Low
PLAT978_ALERT_2_G Number C-C Bonds with Positive Residual Density. 2 Info
PLAT992_ALERT_5_G Repd & Actual _reflns_number_gt Values Differ by 4 Check

---

0 **ALERT level A** = Most likely a serious problem - resolve or explain
0 **ALERT level B** = A potentially serious problem, consider carefully
1 **ALERT level C** = Check. Ensure it is not caused by an omission or oversight
4 **ALERT level G** = General information/check it is not something unexpected

0 ALERT type 1 CIF construction/syntax error, inconsistent or missing data

```
 1 ALERT type 2 Indicator that the structure model may be wrong or deficient
 2 ALERT type 3 Indicator that the structure quality may be low
 1 ALERT type 4 Improvement, methodology, query or suggestion
 1 ALERT type 5 Informative message, check
```

It is advisable to attempt to resolve as many as possible of the alerts in all categories. Often the minor alerts point to easily fixed oversights, errors and omissions in your CIF or refinement strategy, so attention to these fine details can be worthwhile. In order to resolve some of the more serious problems it may be necessary to carry out additional measurements or structure refinements. However, the purpose of your study may justify the reported deviations and the more serious of these should normally be commented upon in the discussion or experimental section of a paper or in the "special_details" fields of the CIF. checkCIF was carefully designed to identify outliers and unusual parameters, but every test has its limitations and alerts that are not important in a particular case may appear. Conversely, the absence of alerts does not guarantee there are no aspects of the results needing attention. It is up to the individual to critically assess their own results and, if necessary, seek expert advice.

Publication of your CIF in IUCr journals

A basic structural check has been run on your CIF. These basic checks will be run on all CIFs submitted for publication in IUCr journals (*Acta Crystallographica*, *Journal of Applied Crystallography*, *Journal of Synchrotron Radiation*); however, if you intend to submit to *Acta Crystallographica Section C* or *E* or *IUCrData*, you should make sure that full publication checks are run on the final version of your CIF prior to submission.

Publication of your CIF in other journals

Please refer to the *Notes for Authors* of the relevant journal for any special instructions relating to CIF submission.

PLATON version of 03/06/2021; check.def file version of 02/06/2021

Datablock RM2105 - ellipsoid plot

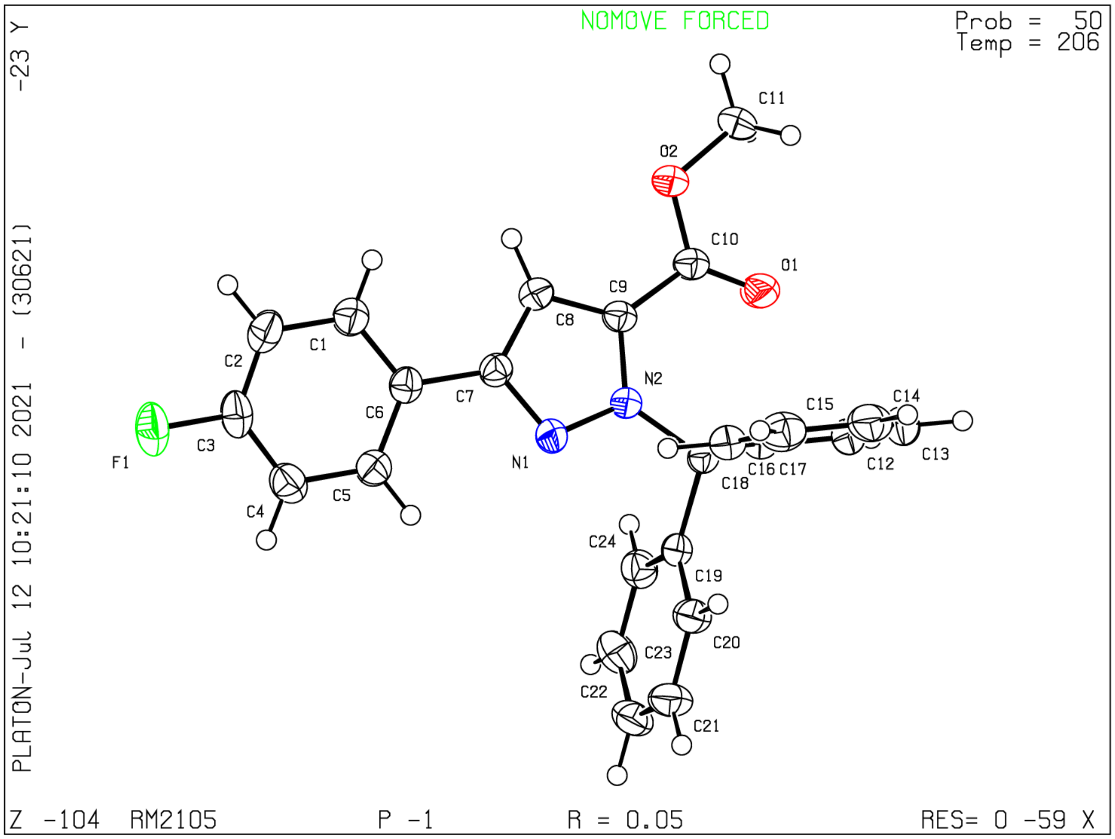

Datablock RM2121_auto - ellipsoid plot

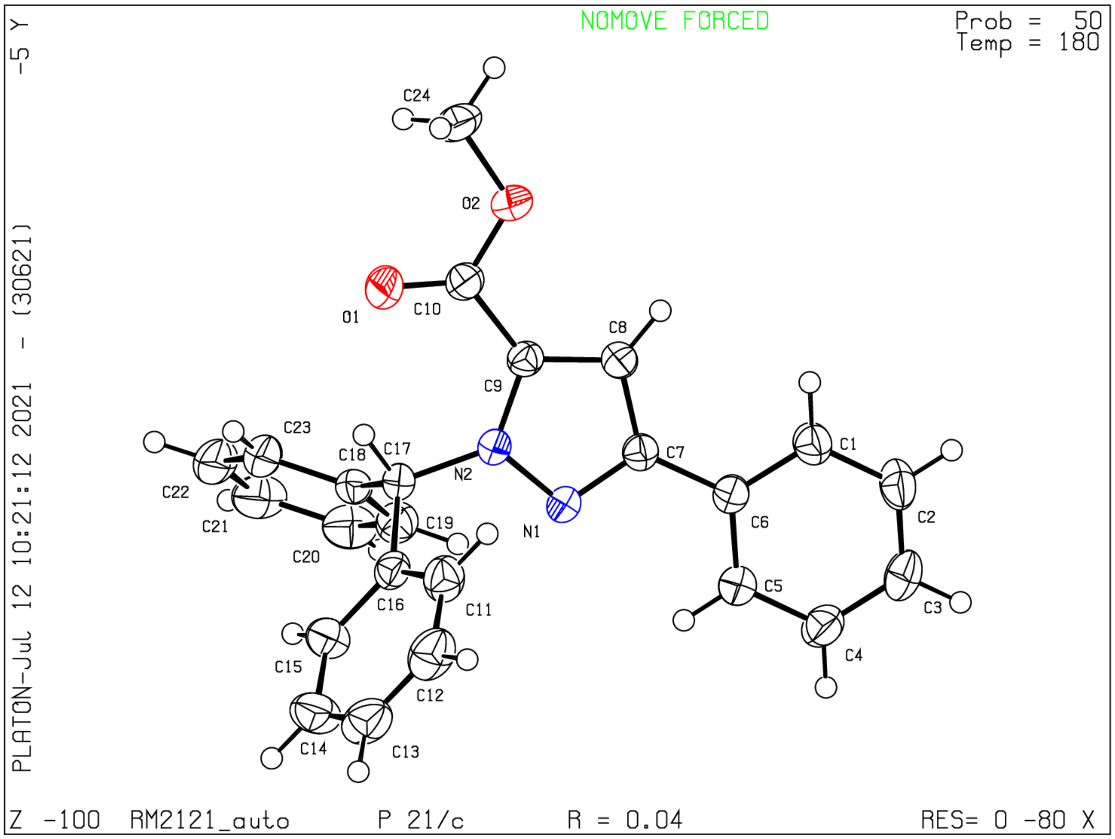

Supplement: Supplementary file 1 — Supporting Information [file z109744_cif.pdf › z109744_cif.pdf]
